# Spatial and temporal changes in cumulative human impacts on the world's ocean

**DOI:** 10.1038/ncomms8615

**Published:** 2015-07-14

**Authors:** Benjamin S. Halpern, Melanie Frazier, John Potapenko, Kenneth S. Casey, Kellee Koenig, Catherine Longo, Julia Stewart Lowndes, R. Cotton Rockwood, Elizabeth R. Selig, Kimberly A. Selkoe, Shaun Walbridge

**Affiliations:** 1 Bren School of Environmental Science and Management, University of California, Santa Barbara, California 93106, USA; 2 Imperial College London, Silwood Park Campus, Buckhurst Road, Ascot SL57PY, UK; 3 National Center for Ecological Analysis and Synthesis, 735 State St Suite 300, Santa Barbara, California 93101, USA; 4 Department of Geography, University of California, Santa Barbara, California 93106, USA; 5 NOAA National Centers for Environmental Information, Silver Spring, Maryland 20910, USA; 6 Betty and Gordon Moore Center for Science and Oceans, Conservation International, Arlington, Virginia 22202, USA; 7 Scripps Institution of Oceanography, University of California, San Diego, California 92093, USA; 8 Hawaii Institute of Marine Biology, University of Hawaii, Kaneohe, Hawaii HI 97644, USA; 9 ESRI, Boston Office, Middleton, Massachusetts 01949, USA

## Abstract

Human pressures on the ocean are thought to be increasing globally, yet we know little about their patterns of cumulative change, which pressures are most responsible for change, and which places are experiencing the greatest increases. Managers and policymakers require such information to make strategic decisions and monitor progress towards management objectives. Here we calculate and map recent change over 5 years in cumulative impacts to marine ecosystems globally from fishing, climate change, and ocean- and land-based stressors. Nearly 66% of the ocean and 77% of national jurisdictions show increased human impact, driven mostly by climate change pressures. Five percent of the ocean is heavily impacted with increasing pressures, requiring management attention. Ten percent has very low impact with decreasing pressures. Our results provide large-scale guidance about where to prioritize management efforts and affirm the importance of addressing climate change to maintain and improve the condition of marine ecosystems.

## Abstract (web summary)

Human pressure on the ocean is thought to be increasing globally, yet the magnitude and patterns of these changes are largely unknown. Here, the authors produce a global map of change in cumulative human pressures over the past 5 years, and show that ∼66% of the ocean has experienced elevated human impact.

## Graphical abstract

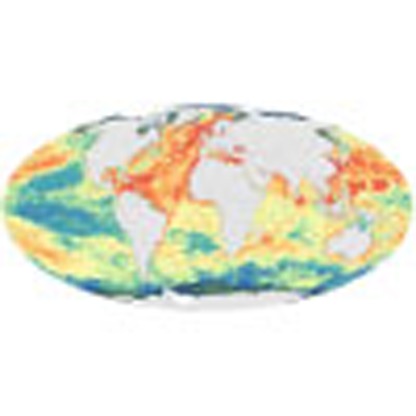

The ocean is crowded with human uses^1^. As human populations continue to grow and migrate to the coasts, demand for ocean space and resources is expanding, increasing the individual and cumulative pressures from a range of human activities. Marine species and habitats have long experienced detrimental impacts from human stressors^2^,^3^, and these stressors are generally increasing globally^4^. However, the spatial patterns of these stressors are varied and the amount of recent change is largely unknown. In many places, we know little about which stressors are having the biggest impact on ecosystem condition, their cumulative effect or how the composition of pressures is changing over time.

Quantifying and mapping local- and global-scale stressors in a standardized, comparable manner offers a powerful means to assess both the spatial pattern and temporal change of individual human pressures, as well as their total impact on natural systems across highly variable geographies^2^. Quantitative methods to map cumulative human impacts were recently developed and initially applied to marine ecosystems globally^2^. To date, these methods have been applied to marine and freshwater regions around the world to assess spatial patterns of cumulative impact^2^,^5^,^6^,^7^,^8^,^9^, and to explore how cumulative impacts affect or relate to other ecological processes or conservation needs (for example, refs 10, 11). These efforts have helped identify which areas and ecosystem types are relatively pristine or heavily impacted, where hotspots of biodiversity and impacts overlap, and which stressors dominate human impact^12^,^13^,^14^, in turn informing biodiversity conservation, threat mitigation and spatial planning decision processes (for example, ref. 15). Missing from these studies is an assessment of the location and intensity of change in cumulative impacts over time. Such temporal assessments would illuminate where and to what degree stressors are increasing or decreasing in intensity and impact, thus providing a means to assess management efficacy and adaptively respond to change. They can also support proactive management by informing our expectation of future states by tracking current trajectories.

Here we calculate and map the cumulative impact of 19 different types of anthropogenic stress on 20 global marine ecosystem types using best available global-scale data as of 2013 (Supplementary Tables 1–2). For 12 of these anthropogenic stressors, we used equivalent methods and data sources in the current and previous (5 years before) time periods, allowing assessment of the 5-year change in their individual and cumulative impacts (see Supplementary Methods). To help identify regions with different management and conservation needs, we identify areas experiencing the greatest and least cumulative impact and highest or lowest amount of change.

## Results

### Change in cumulative impact

**Figure 1:**
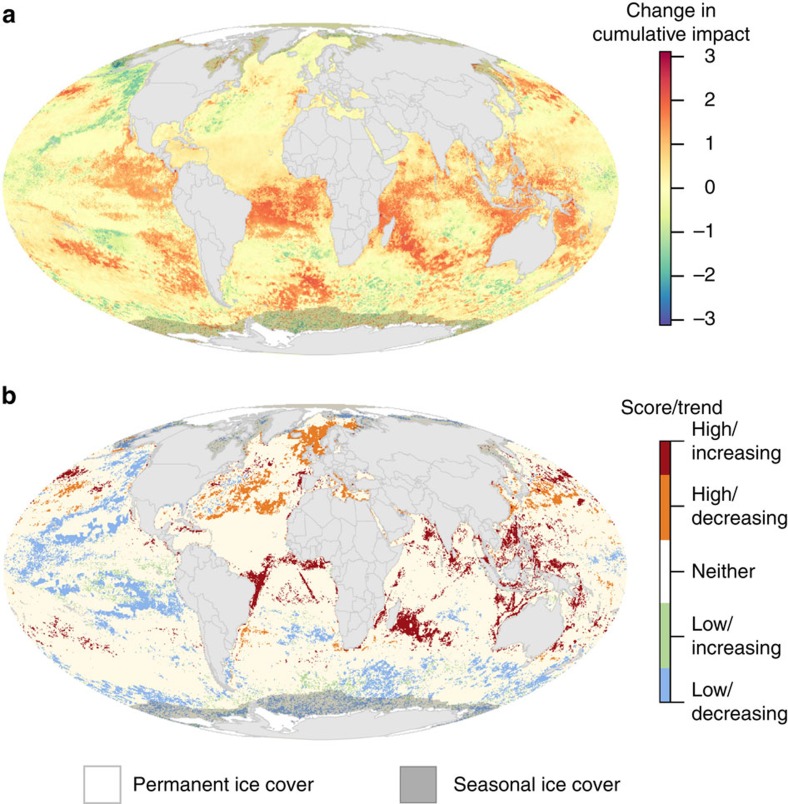
Change in cumulative human impact to marine ecosystems. (**a**) Absolute difference between current (as of 2013) and earlier (as of 2008) per-pixel cumulative impact scores based on 12 anthropogenic stressors that could be compared across time (max cumulative impact score for both periods=11.1). Positive scores represent an increase in cumulative impact. (**b**) Extreme combinations of cumulative impact and impact trend include areas with combinations of the highest (top quartile) and lowest (bottom quartile) impact and increasing (top quartile) and decreasing (bottom quartile) impact. In both panels, areas of permanent sea ice are shaded white and the area within maximum sea ice extent is shaded to indicate where scores are less certain because change in sea ice extent could not be included (see Supplementary Methods).

**Figure 2:**
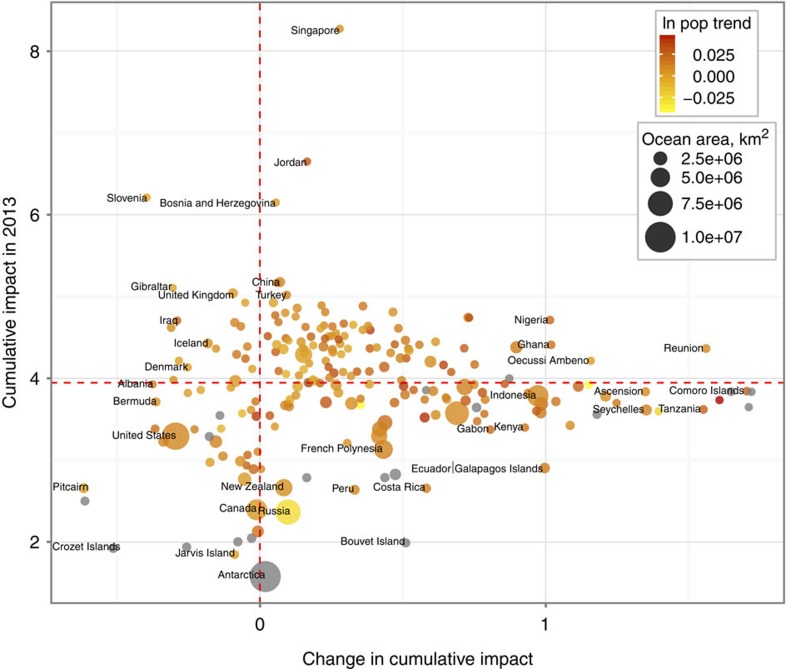
Current cumulative impact versus change in impact. The relationship between current cumulative impact (as of 2013) and 5-year change in impact from 5 years before for each country's EEZ (200 nm) is shown based on the 12 common stressors. Bubbles are scaled to the area (ln) of each country's EEZ and colour-coded by the change in the log of coastal population (25 miles inland) per year from 2008 to 2013; a subset of countries is labelled. Grey bubbles are nearly uninhabited. Horizontal dashed red line is the global median cumulative impact score in 2013; vertical line is no change over time. See Supplementary Tables 1 and 2 for data for all countries.

Nearly 66% of the ocean experienced increases in cumulative impact over the 5-year study span (Fig. 1a, Supplementary Fig. 1). Increases tended to be located in tropical, subtropical and coastal regions, with average increases in 77% of all exclusive economic zones (EEZs; Supplementary Data 1–2). In contrast, only 13% of the ocean experienced decreases over the study span (Supplementary Data 3), with these areas concentrated in the Northeast and Central Pacific and Eastern Atlantic (Fig. 1a). The Southern Ocean showed a patchy mix of increases and decreases. The largest average increases were in French territorial holdings in the Indian Ocean, Tanzania and the Seychelles. The greatest average decreases were within the EEZs of several remote South Pacific islands, the Alaskan coast and several European countries (Slovenia, Albania, Denmark and the Netherlands; Fig. 1a and 2, Supplementary Fig. 2; Supplementary Data 2 and 4). Change in cumulative impact was uncorrelated with current cumulative impact (Supplementary Table 3). Overall, countries with greater increases in coastal population had larger 5-year changes in cumulative impacts. Absolute coastal population size was unrelated to change in cumulative impact. Nevertheless, many places that are largely uninhabited or have relatively low population densities still experienced large increases in impacts (Fig. 2), suggesting that population size may not always drive decreases in ecological condition.

**Figure 3:**
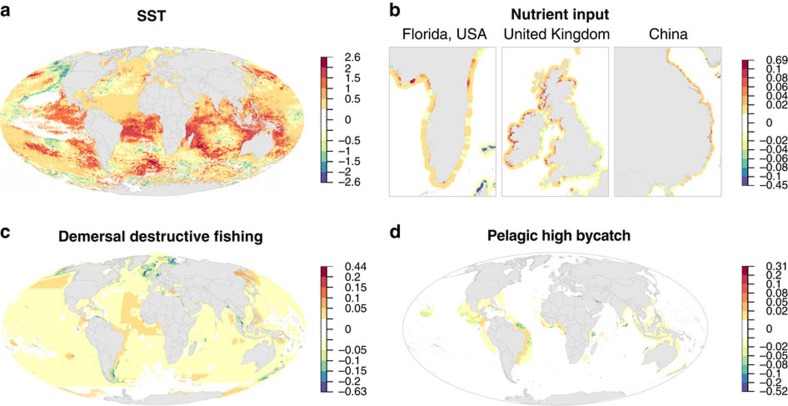
Absolute difference in 2013 versus 2008 per-pixel stressor intensities for four representative stressors. (**a**) Sea surface temperature anomalies, (**b**) nutrient input, (**c**) demersal destructive fishing and (**d**) pelagic high bycatch fishing. Positive scores represent an increase in stressor intensity. Note that colour scales differ among panels and are nonlinear.

Globally, increases in climate change stressors (sea surface temperature anomalies, ocean acidification and ultraviolet radiation) drove most of the increase in cumulative impact (Fig. 3a, Supplementary Fig. 3; Supplementary Table 4), confirming the need to address climate change to maintain and sustain marine ecosystems globally. Commercial fishing impacts increased in <10% of the ocean for any type of commercial fishing, and on average in only 40 (17%) of 239 EEZs (Fig. 3c,d, Supplementary Fig. 3). In fact, impacts from four of the five types of commercial fishing decreased in 70–80% of the ocean, consistent with results suggesting global catch has stabilized or is declining in most parts of the ocean^16^ and that well-managed fisheries are achieving sustainable yields^17^. However, we used fisheries catch as a measure of impact on ecosystems, which does not account for potential longer-lasting impacts of overfishing. We also had to assume that the proportion of catch per gear type remained constant within each EEZ, and so we may underestimate the impact of fishing. In addition, legacy effects of overfishing would not be captured by this analysis, and are likely greatest with habitat-modifying gear and long-lived species that primarily occur along continental shelves and pelagic waters, respectively. Such legacy effects may also be pronounced for invasive species, where current shipping intensity (and associated ballast water release and hull fouling) does not reflect past exposure and establishment of invasive species. Land-based stressors all increased globally (Fig. 3b, Supplementary Fig. 3; Supplementary Table 4), but these increases were concentrated in coastal areas of only 27–52% of all EEZs (depending on type of stressor; Supplementary Data 1).

### Current cumulative impact

**Figure 4:**
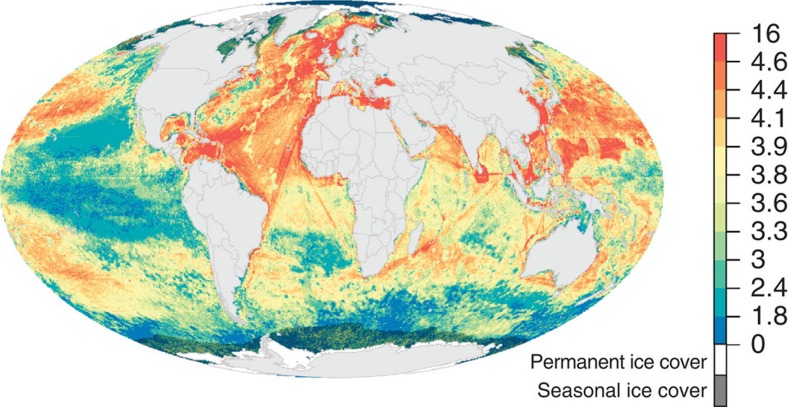
Cumulative human impact to marine ecosystems as of 2013. Impact scores are based on all 19 anthropogenic stressors. Colours are assigned to 10-quantiles in the data, except the highest scores which are the top 5% of scores. Areas of permanent sea ice are shaded white and the area within maximum sea ice extent is masked to indicate where scores are less certain because change in sea ice extent could not be included (see Supplementary Methods).

This updated assessment of cumulative impact confirms previous findings^2^ that no part of the global ocean is without human influence. Nearly the entire ocean (97.7%) is affected by multiple stressors. Several ‘hotspots' of cumulative impact exist where nearly all stressors overlap, most notably in the North Sea and South and East China Seas (Fig. 4, Supplementary Fig. 4). The many stressors associated with climate change (anomalously high sea surface temperatures, ocean acidification and increasing ultraviolet radiation) dominate humanity's footprint on the open ocean, but commercial fishing and shipping also cover large areas of the oceans and contribute significantly to overall impact (Supplementary Figs 5–7; Supplementary Data 5 and 6). In nearshore coastal waters, stressor combinations are more complex and varied (Supplementary Figs 4 and 8; Supplementary Data 7 and 8). National waters currently experiencing highest estimated impacts include those off Singapore, Jordan, Slovenia and Bosnia (Supplementary Fig. 5), while the most impacted coastal ecoregions^18^ include the Faroe Islands, Eastern Caribbean, Cape Verde and Azore islands (Supplementary Data 1 and 3). Least impacted geographic areas are primarily in the poles, but also include relatively large areas in the central Pacific like the waters surrounding Jarvis Island and Palmyra Atoll (USA) and the Line Group of Kiribati, as well as temperate ecoregions around Argentina and the northeast Pacific (Fig. 4; Supplementary Data 1 and 3).

## Discussion

These patterns of change in pressures over time offer guidance on where mitigation efforts are most needed (that is, where cumulative impacts are strongly increasing) and where past management actions may be paying dividends (impacts are high but strongly decreasing; Fig. 1a). Furthermore, overlaying the current (best available data as of 2013) cumulative impact map with 5-year changes in cumulative impact (Fig. 1b) reveals two scenarios of particular importance to management: areas of high and increasing impact, and areas of low and decreasing impact. The former scenario (5% of the ocean) merits immediate management action, focusing on pressure mitigation; the latter scenario (10% of the ocean) may be a lower priority, although controlling or decreasing pressures on already low impact areas could be strategic (Fig. 1b; Supplementary Table 5). Several areas of very high impact (North Sea, Mediterranean Sea and East China Sea; Fig. 4) experienced decreases in cumulative impact, while many offshore regions in the subtropical Atlantic and Indian Oceans that previously had relatively low impact scores saw large increases (Fig. 1b).

Decreases in individual stressors were generally relatively small on average and limited in area, but occurred for each stressor type and included areas of notable decrease (Supplementary Fig. 3). For example, demersal destructive (for example, trawl) fishing decreased significantly in many European countries, pelagic high bycatch (for example, longline) fishing decreased in several parts of the Middle East, sea surface temperature anomalies decreased in the Line Islands in the Pacific and around Alaska, USA and nutrient input decreased in the Adriatic Sea. Because of legacy effects of overfishing, decreases in catch may not translate into improved ecosystem condition or sustainable yields. Few stressors expanded their global footprint (Supplementary Table 4), primarily because their extent was already nearly global^2^. In short, even where some stressors show signs of decreasing, cumulative impact across all stressors is generally increasing, especially in coastal areas where human uses of the ocean are the greatest.

Our results do not account for potential losses in habitat which would likely occur with high intensity of multiple overlapping stressors, especially within intertidal and nearshore habitats. Habitat extent is poorly known for most marine habitats, and change in extent essentially unknown for all; our analyses used the same habitat extent for all habitats for both time periods. While change in sea ice extent is well mapped via satellites, we did not include sea ice as a habitat because it is naturally highly variable, or as a stressor because its impact on ecosystems remains poorly understood^19^ (see Supplementary Methods); we represent this uncertainty as shaded areas on mapped results of cumulative human impact. Trends in sea ice extent over the 5 years of our analyses varied spatially but many locations had already lost significant amounts of ice; where significant loss occurred during the time period of our analyses, our estimates of cumulative impact (and climate stressor impact in particular) would be significantly underestimated.

Cumulative impact assessment currently relies on assumptions of linear and additive responses of natural systems to stressors^20^. However, marine ecosystems may exhibit threshold responses to intense and cumulative stress^21^, creating nonlinear relationships of cumulative impact to ecological condition. Recent syntheses show that nonlinear responses of ecosystems to increases in single stressors are common but difficult to predict^22^. Emerging evidence also suggests that stressor interactions are more commonly synergistic and mitigative than additive^23^. Currently, insufficient information exists to allow incorporation of these relationships into the cumulative impact assessment, but once available they can be accommodated. Furthermore, several known stressors to marine systems could not be included because of insufficient global data, including offshore energy infrastructure (for example, wind farms, submerged pipes and cables, deep sea mining, marine debris). Nevertheless, cumulative impact assessments remain one of the few comprehensive quantitative tools to measure how humans are affecting natural systems, and how actions targeting specific stressors may be expected to improve or exacerbate overall impacts. Because the approach allows direct comparisons, it is possible to measure change through time, allowing for a detailed view of where individual and cumulative human impacts are increasing or decreasing and which stressors are most important for driving those changes. This analysis of change over 5 years cannot fully account for natural longer term climate variations (such as decadal oscillations), but it provides a strong indication of direction (and location) of human-caused change. This assessment is thus constructive both to set management priorities and assess effectiveness of past actions, and is particularly useful for marine spatial planning and ecosystem-based management that must address the cumulative impact of multiple human uses of the ocean^24^,^25^.

Empirically, measuring overall condition of natural systems remains difficult and resource intensive. Few, if any, approaches exist that allow direct comparison of condition globally and across scales. Our assessment of cumulative impact, although a prediction rather than measurement of condition, is highly valuable because of its global, scalable and quantitative comparability. Furthermore, previous global assessments^2^ and a regional comparison of cumulative impact scores and ecosystem condition in the Baltic Sea^26^ suggest modelled impact scores describe actual condition reasonably well. Our approach also moves beyond assessing change in intensity of stressors (that is, ‘ecological footprints') by accounting for vulnerability or resilience of ecosystems to different stressor types^27^. In other words, our approach accounts for the reality that increases in stressor intensity may not lead to changes in ecosystem condition, while in other cases (that is, more sensitive ecosystems) small increases in intensity could cause large changes in condition.

Our results offer guidance for most management and conservation strategies, both proactive and reactive. For example, results can support prioritization of regions or stressors of concern globally (for example, as is done by the Global Environment Facility branch of the World Bank) and nationally (for example, through US's National Ocean Policy), track progress towards meeting management and policy objectives (for example, as mandated by the European Union's Marine Strategy Framework Directive) and potentially even set targets for total acceptable cumulative human impact on ecosystems in support of broader ecosystem-based management goals. If the ocean is going to continue to support and sustain human values and needs, addressing and mitigating cumulative impacts must become standard. Our finding that the majority of global waters are currently experiencing large and increasing cumulative impact of human activities brings urgency to addressing this need.

## Methods

### General model

Calculation of cumulative impacts followed and built on the approach developed previously^2^,^6^. Cumulative impact (*I*_C_) is the per-pixel average of the habitat vulnerability-weighted stressor intensities (see Supplementary Table 1 for list of stressors and habitats), where weights (*μ*_*i,j*_) are determined by the vulnerability of each *i... m* habitat (*E*) to each *j... n* stressor (*D*), such that:


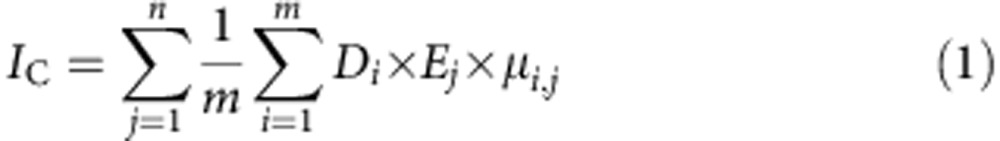


In the previous global analyses^2^, the sum of weighted intensities was used to account for the three-dimensionality of the ocean; here we calculate the average (following ref. 6) to produce a single two-dimensional map. The previous approach (and results) was not used for temporal comparisons (see ‘temporal comparisons' below). We used ecosystem vulnerability weights (*μ*_i,j_) developed previously^27^ for all stressor–habitat combinations, including new ones added here, as all stressor–habitat combinations were assessed in that study.

### Habitat and stressor data

For nearshore areas, we assumed benthic habitats are well-mixed with the water column above and so treat them as a single depth layer, as done elsewhere^6^. At depths >60 m, we treat the surface waters as a separate pelagic habitat, and at depths >200 m we assume three distinct depth layers (benthic, deep pelagic and surface pelagic). For offshore waters (>60-m depth), fully overlapping habitats from benthic and pelagic systems lead to imperfect representations of three-dimensional impact in a two-dimensional representation; in nearshore coastal areas there is only a single depth layer, removing this issue.

We updated most stressor data layers used previously^2^, and used newly developed or significantly improved data sources for four layers (nutrient and organic land-based pollution, commercial shipping and port volume, which is used for invasive species, and ocean-based pollution), as well as data for two stressors new to this analysis (light pollution and sea level rise; see Supplementary Table 1 for full list of data). The only data layers that could not be updated were inorganic pollution from land-based sources, artisanal fishing and ocean acidification, and thus in those cases we used the exact same data as used in the previous analyses. Habitat data are infrequently updated and improved, and so all of the habitat data remain the same as those used previously^2^. As such, changes in cumulative impact scores are entirely due to changes in stressor intensities.

### Normalization of stressor data

We first log[*X*+1] transformed each stressor data layer, except benthic structures. Benthic structures were treated as binary data since an oil rig either exists or does not. The transformation of data appropriately reduces the effect of extreme outliers when rescaling the data to assign the relative impact of different levels of the anthropogenic stressors considered here^28^. All data layers were then rescaled between 0 and 1, with the highest per-pixel transformed value for each stressor across either time period set=1. We rescaled data in this way to ensure comparability across time periods (that is, using the same reference point across time). If stressor intensities increase in the future beyond this reference value, then analyses across all years of analyses would need to be redone. Rescaling allows for direct comparison among drivers with dramatically different units of measurement.

### Temporal comparisons

We recalculated previous (2008) scores using updated methods to allow direct comparison with current (best available data as of 2013) results. Because some data sources were new or were developed using new methods that could not be applied to past data, we restricted temporal analyses to only those data layers that could be directly compared across time. This left 12 stressor layers and all habitat data (see Supplementary Table 1).

To help address potential management priorities, we classified each pixel as high, medium or low current (2013) cumulative impact and as increasing, no change and decreasing impact across the 5-year time frame of the study. High and low impact categories were classified as the top and bottom 25% of values, respectively, with all other values categorized as medium. This led to cutoff values of >4.02 (high impact) and <2.739 (low impact). Increasing and decreasing impact were similarly classified as the top and bottom 25% of values, respectively, with cutoff values of >0.602 (increasing) and <−0.045 (decreasing).

### Data projection and representation

We used the same land-sea mask (and derived coastline) as we developed previously^2^. As was also done for that study, all data were represented at ∼1 km^2^ resolution, even though several layers had native resolutions at coarser scales. In doing so, we assumed the coarse-scale value was evenly distributed across all 1 km^2^ cells within that region. For climate change drivers (sea surface temperature (SST) and ultraviolet anomalies and changes in ocean acidification), this assumption is reasonable given the scale at which those drivers act. The land-based drivers, human population data and benthic structures data were all available or produced at ∼1 km^2^ resolution, and spread of the impact of these stressors into the ocean at the same resolution is reasonable. Regardless, when coarse-scale data are distributed equally to all 1 km^2^ cells within the larger area, the coarser scale pattern is essentially recreated while the finer resolution information is preserved where and when it is appropriate. Finally, before all analyses, we converted all data to the Mollweide projection with a WGS84 datum as it is an accurate single global projection that preserves area and allows data transfer and analysis among operating systems and software.

### Summarizing results

To help aid decision making at regional, national and sub-national scales, we summarized individual and cumulative impact of stressors, and recent change in impact, by EEZs (using international standards for boundary delineation; ref. 29), marine ecoregions^18^, large marine ecosystems and Food and Agriculture Organization (FAO) high seas regions. In each case, we averaged per-pixel values (current impact and change in impact), allowing direct comparison among regions despite large differences in size.

### Input data

Methods for preparing stressor data that were unchanged from the previous analyses (Supplementary Table 1) are described in detail elsewhere^2^. Stressors with updated data were prepared using more recent years from the same data source. In these cases, we describe the new data but do not elaborate methods. We primarily focus on describing those layers where updating required new methods. Data for all habitats were unchanged from previous analyses^2^. The 20 different habitats included are listed in Supplementary Table 1.

### Vulnerability weights

We used nearly identical vulnerability weights as developed and used before^27^. Because global data layers used here do not perfectly match the categories used in these vulnerability studies, we made the following adjustments. Commercial activity was equated with our shipping layer, non-point source weights were used for our pollution layers, nutrient input was the average of oligotrophic and eutrophic weights, and demersal nondestructive low bycatch commercial fishing weights were determined by multiplying high bycatch values by 0.75. The non-zero weights for sea level rise in deeper habitats and pelagic waters did not make sense and so were set equal to zero (*N*=5). Light pollution weights had to be derived new for this study; peer-reviewed literature and our own expert judgment were used to set these values. Supplementary Table 2 provides the full set of vulnerability weights.

### Methodological comparisons

To compare results from current methods and updated data sources to those from past methods^2^, we correlated per-pixel output for 2008 from past and current approaches (results shown in Supplementary Fig. 9). Differences are expected for at least two reasons. First, the past approach summed rather than averaged impact scores across habitats within a pixel. This method was changed, following previous methods^6^, to account for imperfect habitat extent data. For pixels with only a single habitat, the two methods produce identical results. In coastal areas, and in particular intertidal areas, multiple habitats typically exist within a single pixel, and so differences in scores would be expected to be concentrated in these areas. This is in fact what we found (see Supplementary Fig. 9).

Second, differences in how stressor data were normalized should lead to very minor differences when maximum stressor intensity has increased over time, which it has for many stressors. Previously^2^, stressors were normalized to the maximum value for that time period, whereas in the current analysis comparing current to previous time periods, stressors were normalized to the maximum across both time periods. We controlled for this when doing the temporal analyses in this study, but did not (and could not) control for this when comparing previously published results to our current results.

## Additional information

**How to cite this article**: Halpern, B. S. *et al.* Spatial and temporal changes in cumulative human impacts on the world's ocean. *Nat. Commun.* 6:7615 doi: 10.1038/ncomms8615 (2015).

## Supplementary Material

Supplementary Figures, Supplementary Tables, Supplementary Methods and Supplementary References Supplementary Figures 1-9, Supplementary Tables 1-6, Supplementary Methods and Supplementary References

Supplementary Data 1 Average impact scores for each stressor and for cumulative impact in 2013 for each EEZ, in decreasing order of average cumulative impact. True zero values are indicated by zeros with no trailing decimals; very small values are zeros with several zero decimal values.

Supplementary Data 2 Average difference in impact scores for each stressor and for cumulative impact between 2013 and 2008 for each EEZ. Differences could only be calculated for the 12 (of 19) stressor layers that had data for both time. True zero values are indicated by zeros with no trailing decimals; very small values are zeros with several zero decimal values.

Supplementary Data 3 Average impact scores for each stressor and for cumulative impact in 2013 for each marine ecoregion of the world (MEOW; Spalding et al. 2007), in decreasing order of average cumulative impact. True zero values are indicated by zeros with no trailing decimals; very small values are zeros with several zero decimal values.

Supplementary Data 4 Average difference in impact scores for each stressor and for cumulative impact between 2013 and 2008 for each marine ecoregion of the world (MEOW; Spalding et al. 2007). Differences could only be calculated for the 12 (of 19) stressor layers that had data for both time. True zero values are indicated by zeros with no trailing decimals; very small values are zeros with several zero decimal values.

Supplementary Data 5 Average impact scores for each stressor and for cumulative impact in 2013 for each FAO high seas region, in decreasing order of average cumulative impact. True zero values are indicated by zeros with no trailing decimals; very small values are zeros with several zero decimal values.

Supplementary Data 6 Average difference in impact scores for each stressor and for cumulative impact between 2013 and 2008 for each FAO high seas region. Differences could only be calculated for the 12 (of 19) stressor layers that had data for both time. True zero values are indicated by zeros with no trailing decimals; very small values are zeros with several zero decimal values.

Supplementary Data 7 Average impact scores for each stressor and for cumulative impact in 2013 for each large marine ecosystem (LME), in decreasing order of average cumulative impact. True zero values are indicated by zeros with no trailing decimals; very small values are zeros with several zero decimal values.

Supplementary Data 8 Average difference in impact scores for each stressor and for cumulative impact between 2013 and 2008 for each large marine ecosystem (LME). Differences could only be calculated for the 12 (of 19) stressor layers that had data for both time. True zero values are indicated by zeros with no trailing decimals; very small values are zeros with several zero decimal values.
