# Supplementary material for: Spatial and temporal changes in cumulative human impacts on the world's ocean
Source: Nat Commun. 2015 Jul 14;6:7615. doi: 10.1038/ncomms8615 (PMC4510691; doi:10.1038/ncomms8615)
Supplement: Supplementary Figures, Supplementary Tables, Supplementary Methods and Supplementary References — Supplementary Figures 1-9, Supplementary Tables 1-6, Supplementary Methods and Supplementary References [file ncomms8615-s1.pdf]

Supplementary Figures

Supplementary Figure 1

Histograms for per-pixel scores of A) difference in cumulative impact from 2008 to 2013, B) difference in individual stressors from 2008 to 2013, and C) cumulative impact score in 2013. For A, the skew in distribution to positive values indicates that more places experienced an increase in cumulative impact.

A.

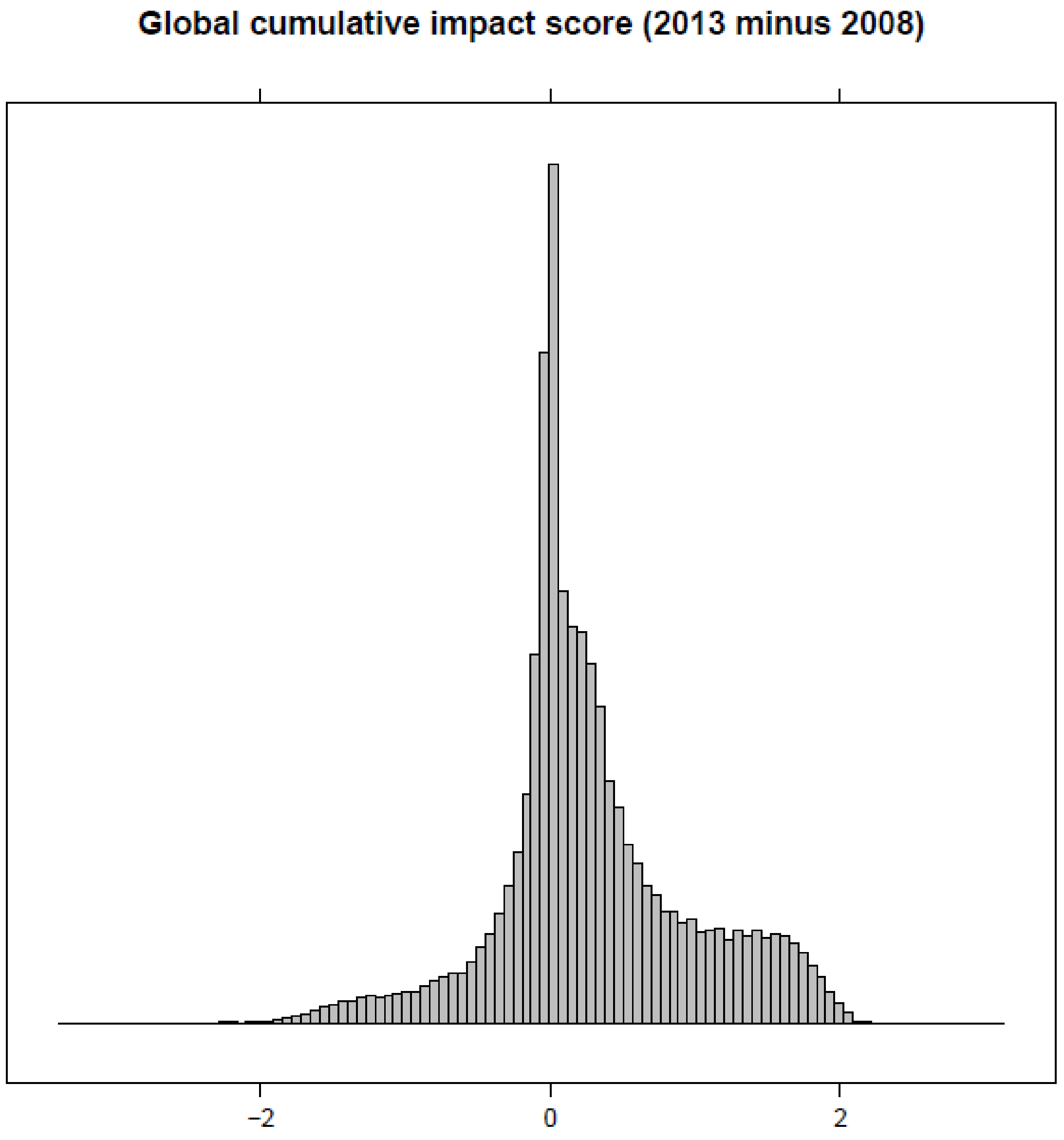

B.

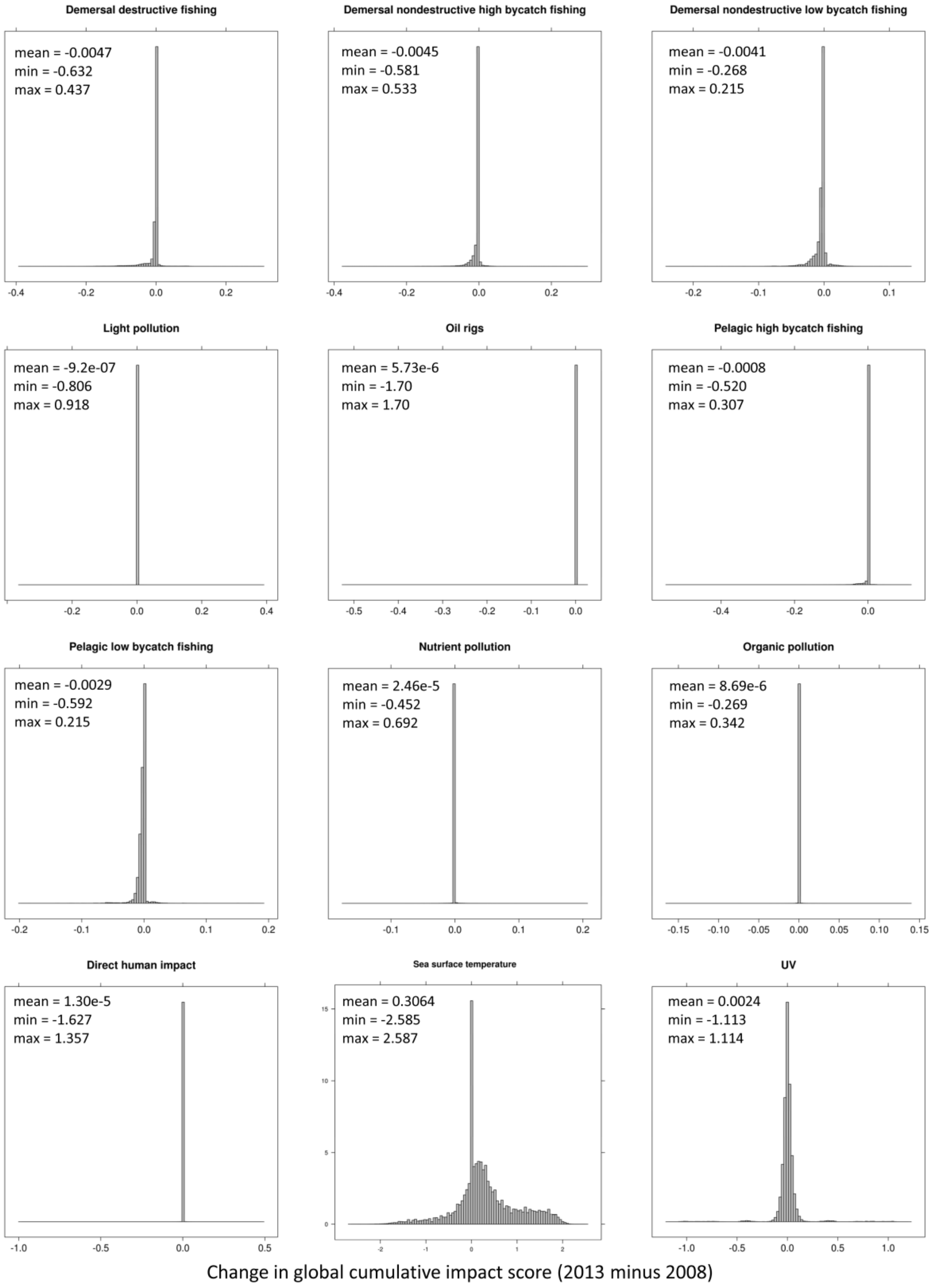

Demersal destructive fishing
mean = -0.0047
min = -0.632
max = 0.437
Demersal nondestructive high bycatch fishing
mean = -0.0045
min = -0.581
max = 0.533
Demersal nondestructive low bycatch fishing
mean = -0.0041
min = -0.268
max = 0.215
Light pollution
mean = -9.2e-07
min = -0.806
max = 0.918
Oil rigs
mean = 5.73e-6
min = -1.70
max = 1.70
Pelagic high bycatch fishing
mean = -0.0008
min = -0.520
max = 0.307
Pelagic low bycatch fishing
mean = -0.0029
min = -0.592
max = 0.215
Nutrient pollution
mean = 2.46e-5
min = -0.452
max = 0.692
Organic pollution
mean = 8.69e-6
min = -0.269
max = 0.342
Direct human impact
mean = 1.30e-5
min = -1.627
max = 1.357
Sea surface temperature
mean = 0.3064
min = -2.585
max = 2.587
UV
mean = 0.0024
min = -1.113
max = 1.114
Change in global cumulative impact score (2013 minus 2008)

C.

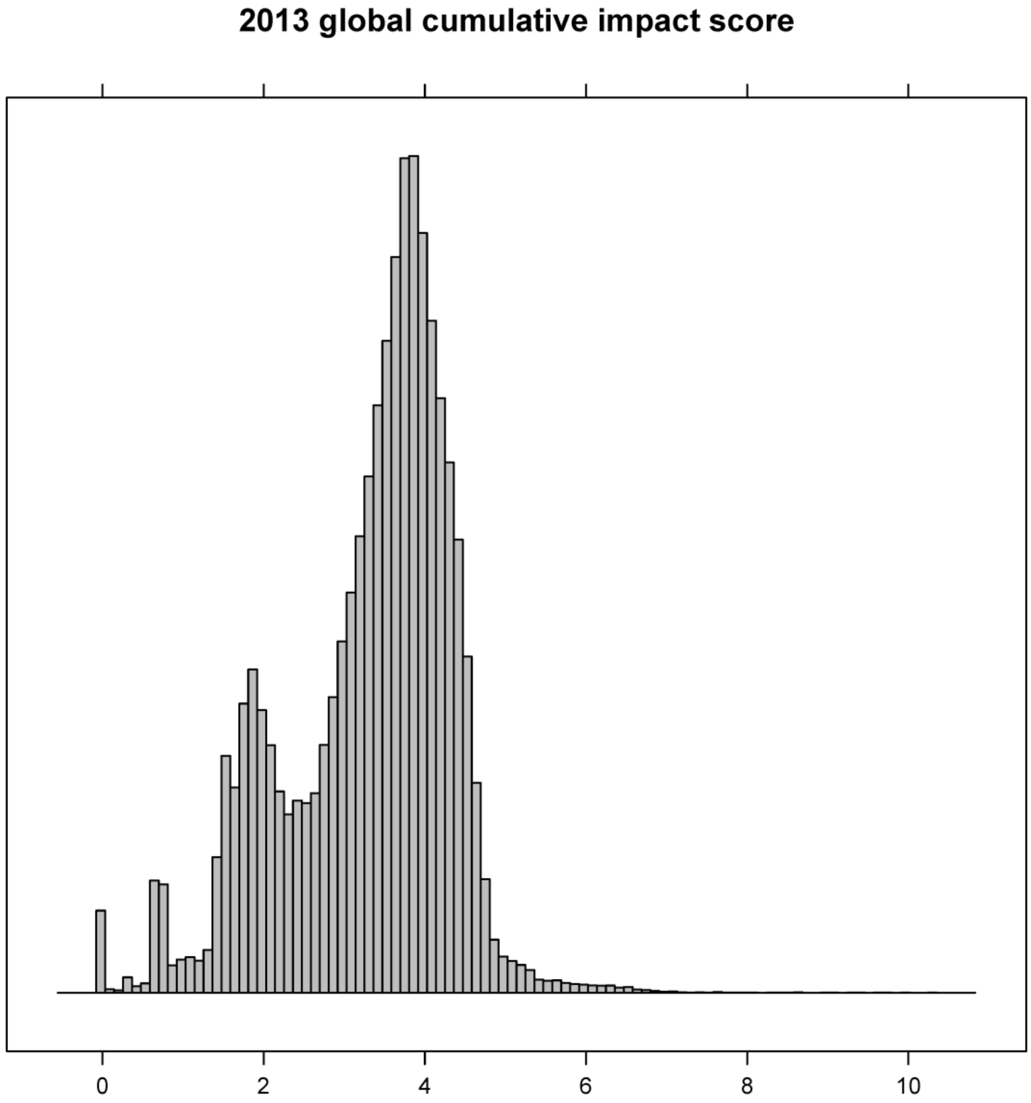

Cumulative Impact score

Supplementary Figure 2

Comparison of average per-pixel cumulative impact scores for each A) EEZ (N=221) and B) marine ecoregion (N=232), based on 12 stressors for which temporal comparisons could be made. Ecoregions increase more primarily because they are coastal (to 200m depth contour) and thus experience most stressors, in particular land-based ones, whereas EEZs include large areas of pelagic ocean that experience little land-based impact. Dash lines along axes show distribution of points within the plot. The dashed yellow line is the line of no change. Outlier points are labeled for ease of reference; full results provided in Supplementary Data 2, 4.

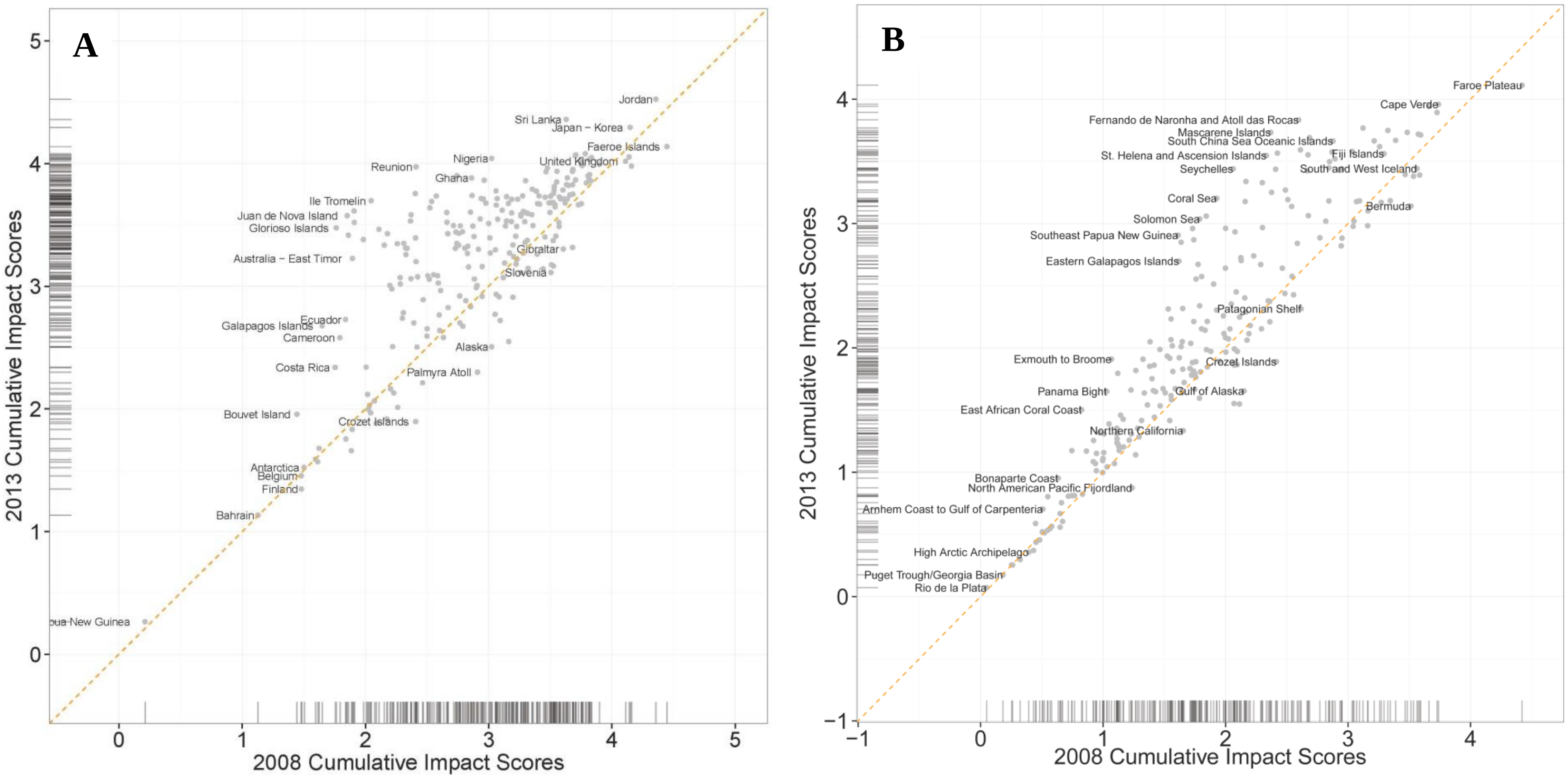

Supplementary Figure 3

Global maps of difference in impact scores for each of 12 stressors with data for 2008 and 2013. The first set of 7 maps is for stressors that affect most parts of the ocean; the second set of 5 maps includes zoomed in views to 4 representative areas for stressors that affect coastal areas. Note that color ramping changes among all figures.

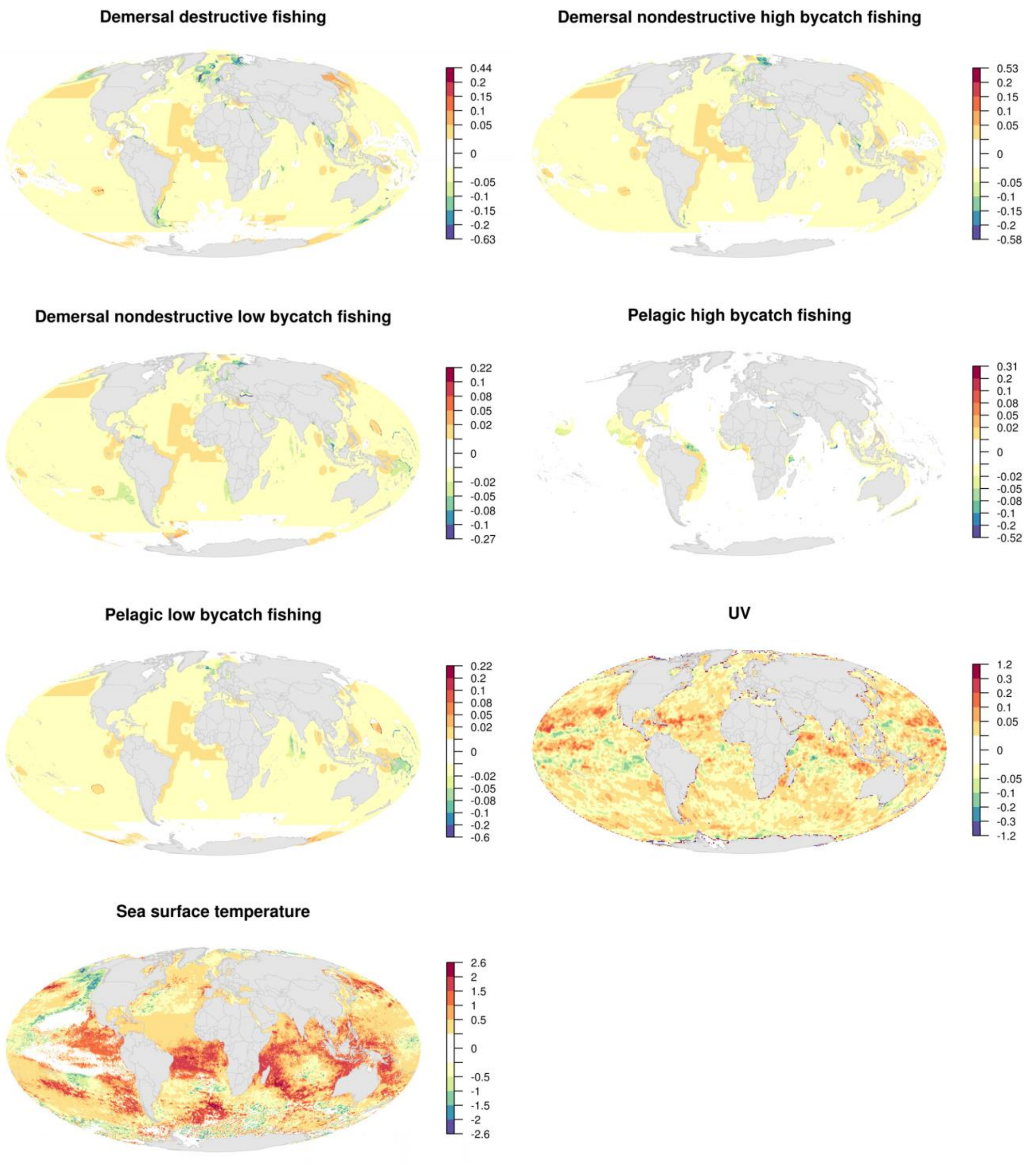

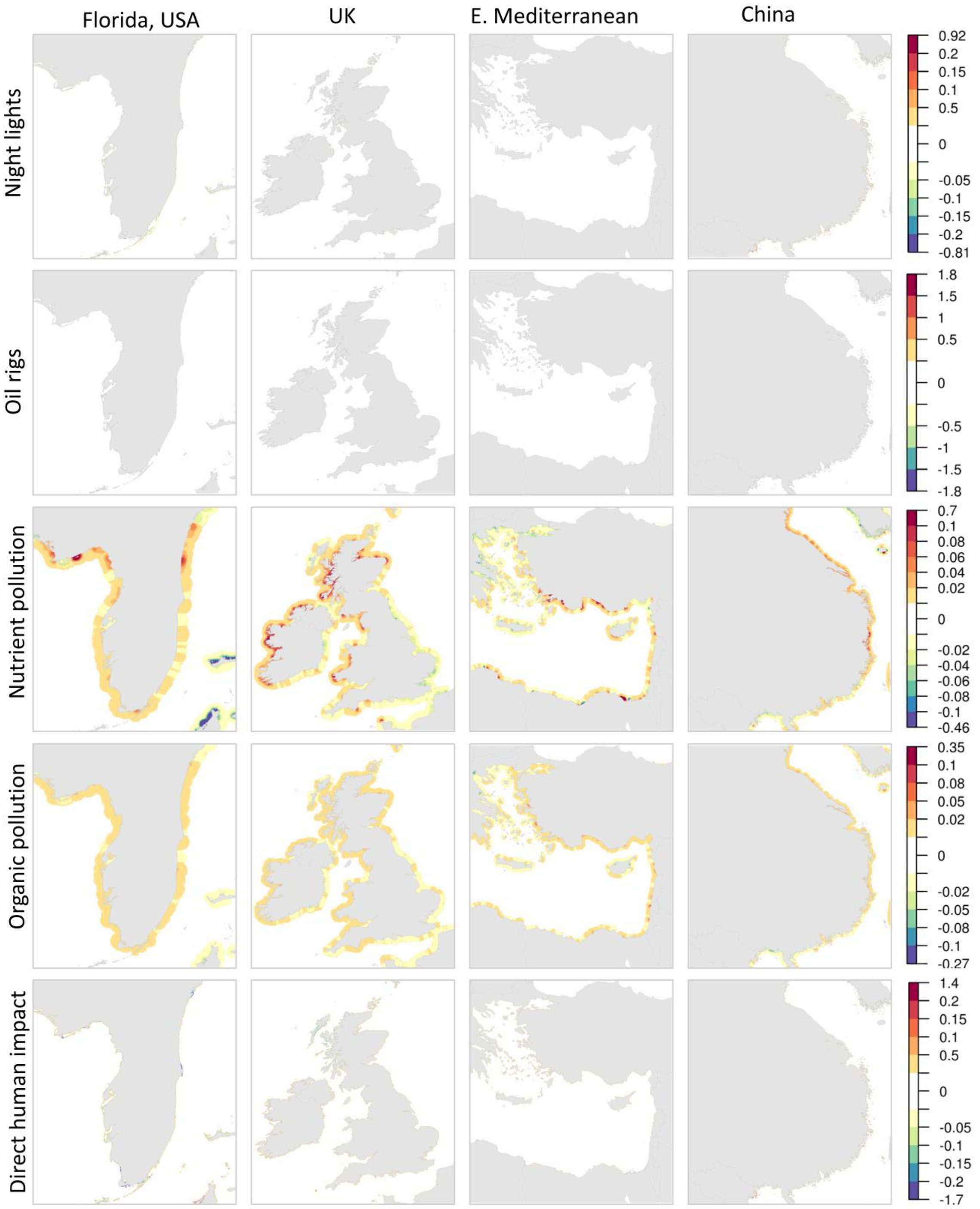

Florida, USA
UK
E. Mediterranean
China
Night lights
Oil rigs
Nutrient pollution
Organic pollution
Direct human impact
0.92
0.2
0.15
0.1
0.5
0
-0.05
-0.1
-0.15
-0.2
-0.81
1.8
1.5
1
0.5
0
-0.5
-1
-1.5
-1.8
0.7
0.1
0.08
0.06
0.04
0.02
0
-0.02
-0.04
-0.06
-0.08
-0.1
-0.46
0.35
0.1
0.08
0.05
0.02
0
-0.02
-0.05
-0.08
-0.1
-0.27
1.4
0.2
0.15
0.1
0.5
0
-0.05
-0.1
-0.15
-0.2
-1.7

Supplementary Figure 4

Map of count of stressors per pixel. Four zoomed panels (left to right) are Florida, USA; United Kingdom, Eastern Mediterranean, and China. Areas of permanent sea ice are shaded white and the area of maximum sea ice extent is masked to indicate where scores are less certain because change in sea ice extent could not be included (see Supplemental Methods).

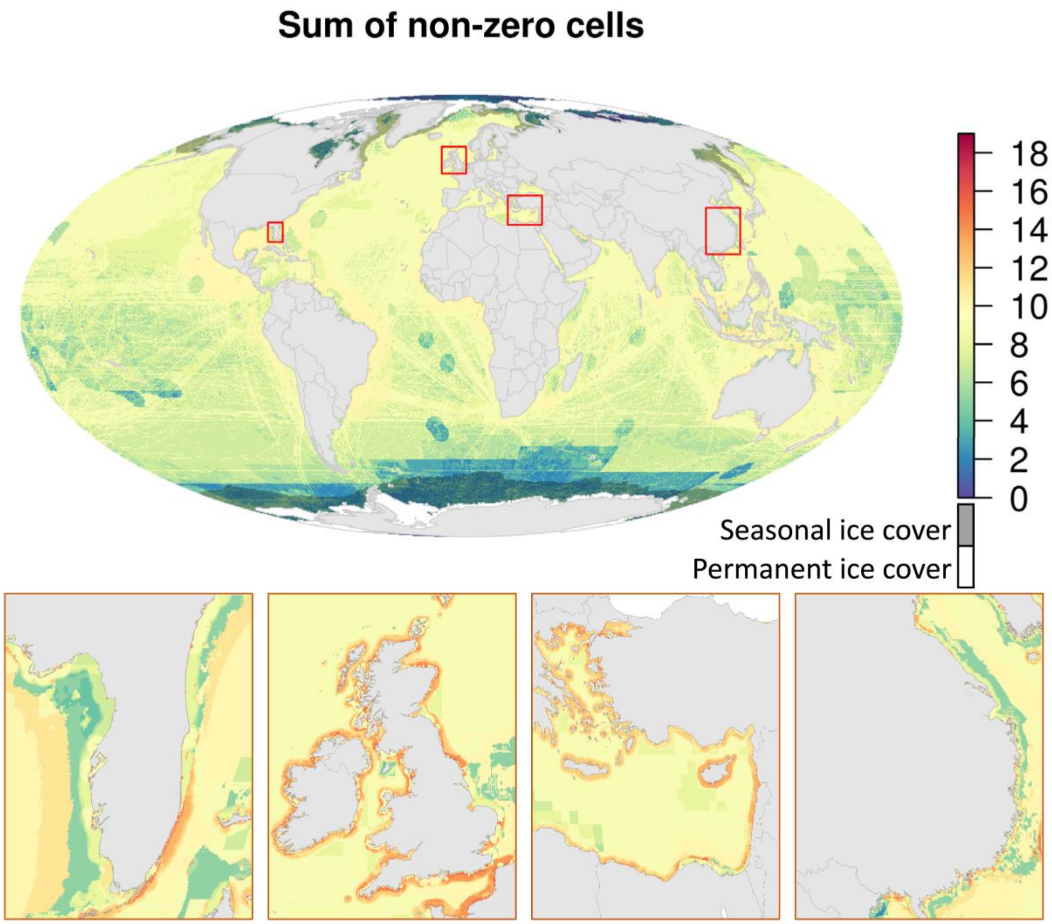

Supplementary Figure 5

Stacked bar charts for each EEZ (rows) showing contribution of each stressor type to the overall cumulative impact score. EEZs are ordered from highest to lowest cumulative impact score; number following EEZ name is our internal numbering system).

(see next page)

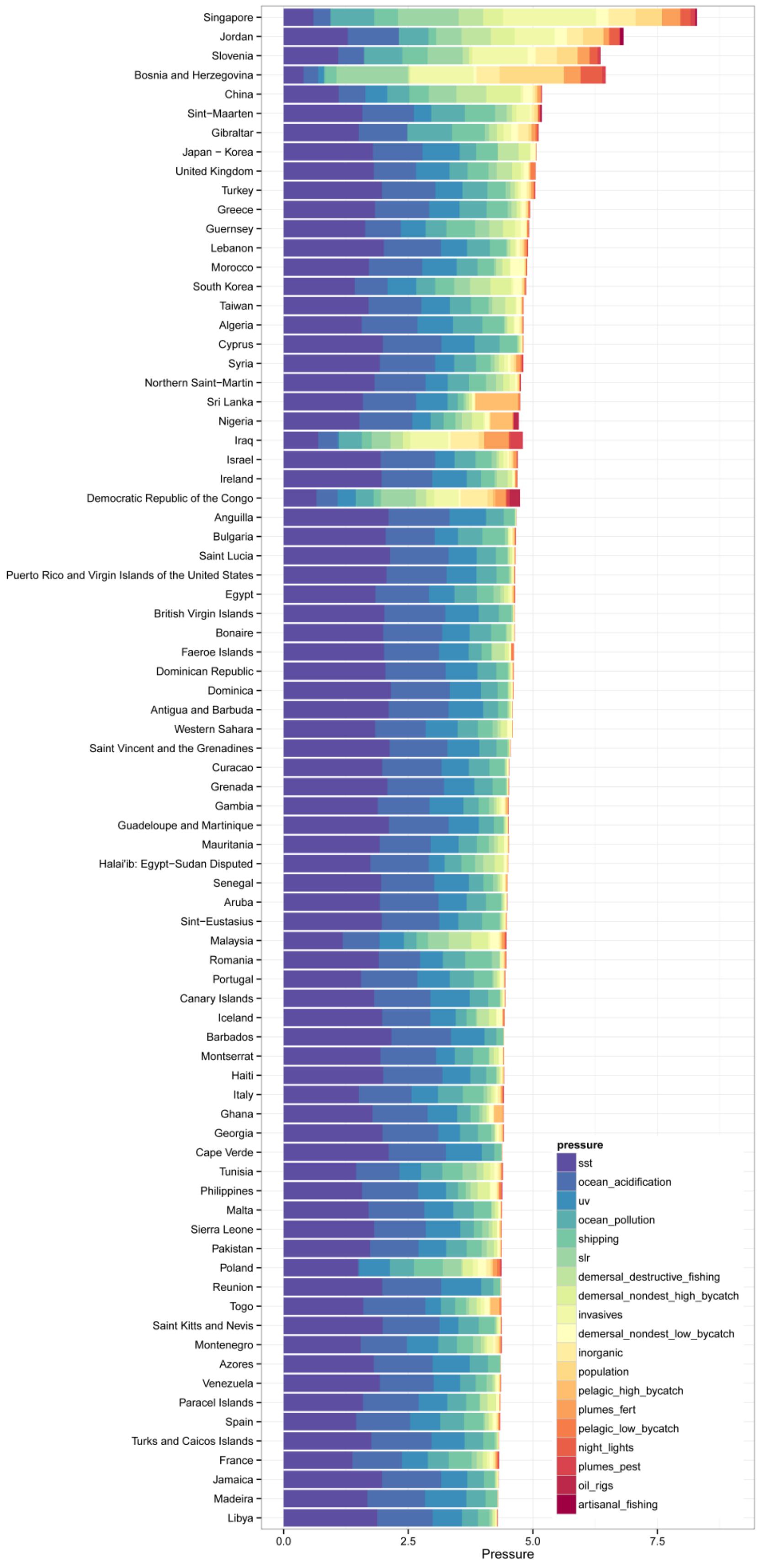
Singapore
Jordan
Slovenia
Bosnia and Herzegovina
China
Sint−Maarten
Gibraltar
Japan − Korea
United Kingdom
Turkey
Greece
Guernsey
Lebanon
Morocco
South Korea
Taiwan
Algeria
Cyprus
Syria
Northern Saint−Martin
Sri Lanka
Nigeria
Iraq
Israel
Ireland
Democratic Republic of the Congo
Anguilla
Bulgaria
Saint Lucia
Puerto Rico and Virgin Islands of the United States
Egypt
British Virgin Islands
Bonaire
Faeroe Islands
Dominican Republic
Dominica
Antigua and Barbuda
Western Sahara
Saint Vincent and the Grenadines
Curacao
Grenada
Gambia
Guadeloupe and Martinique
Mauritania
Halai'ib: Egypt−Sudan Disputed
Senegal
Aruba
Sint−Eustasius
Malaysia
Romania
Portugal
Canary Islands
Iceland
Barbados
Montserrat
Haiti
Italy
Ghana
Georgia
Cape Verde
Tunisia
Philippines
Malta
Sierra Leone
Pakistan
Poland
Reunion
Togo
Saint Kitts and Nevis
Montenegro
Azores
Venezuela
Paracel Islands
Spain
Turks and Caicos Islands
France
Jamaica
Madeira
Libya
0.0
2.5
5.0
7.5
Pressure
pressure
sst
ocean_acidification
uv
ocean_pollution
shipping
slr
demersal_destructive_fishing
demersal_nondest_high_bycatch
invasives
demersal_nondest_low_bycatch
inorganic
population
pelagic_high_bycatch
plumes_fert
pelagic_low_bycatch
night_lights
plumes_pest
oil_rigs
artisanal_fishing

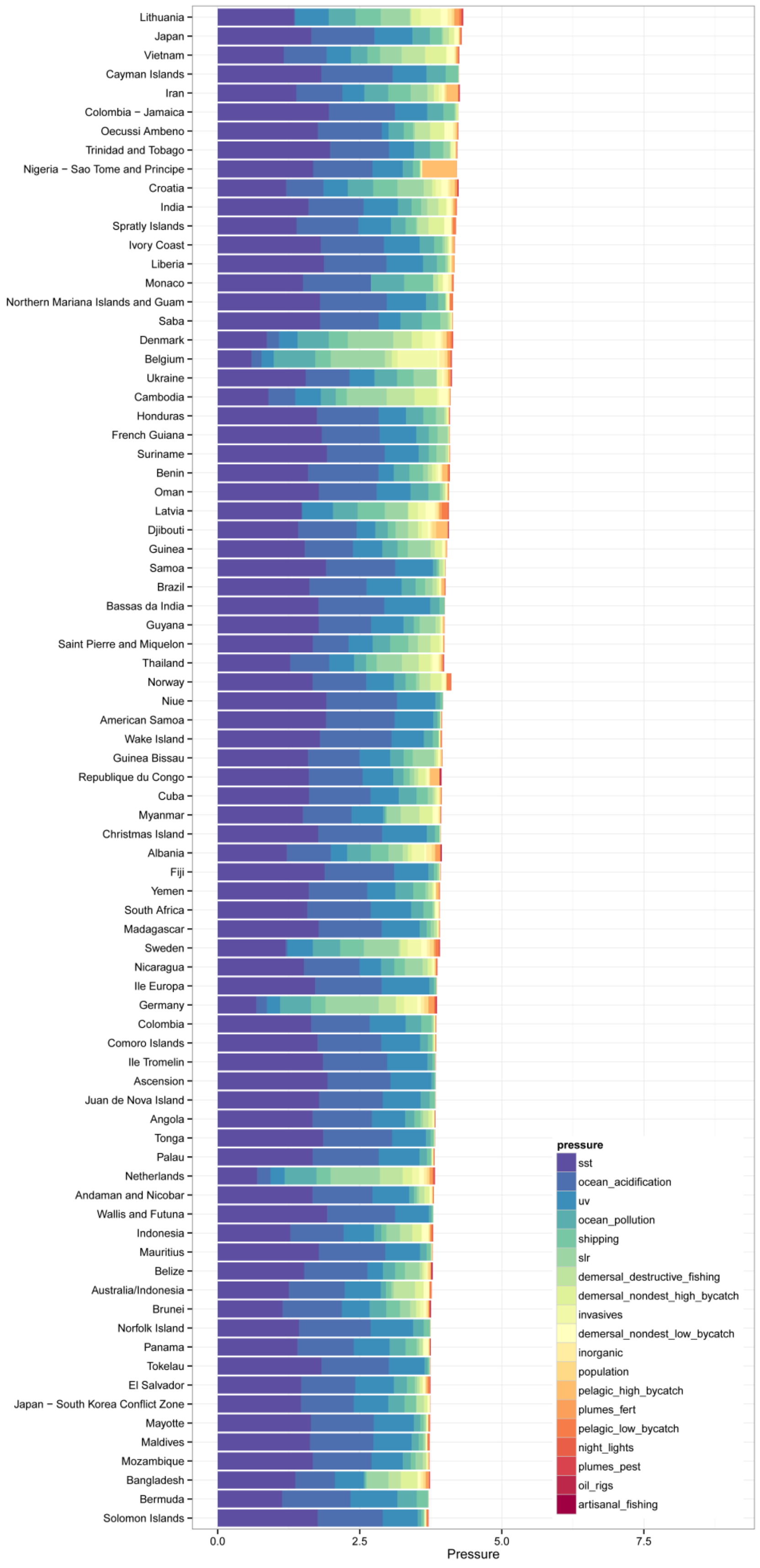

Lithuania
Japan
Vietnam
Cayman Islands
Iran
Colombia − Jamaica
Oecussi Ambeno
Trinidad and Tobago
Nigeria − Sao Tome and Principe
Croatia
India
Spratly Islands
Ivory Coast
Liberia
Monaco
Northern Mariana Islands and Guam
Saba
Denmark
Belgium
Ukraine
Cambodia
Honduras
French Guiana
Suriname
Benin
Oman
Latvia
Djibouti
Guinea
Samoa
Brazil
Bassas da India
Guyana
Saint Pierre and Miquelon
Thailand
Norway
Niue
American Samoa
Wake Island
Guinea Bissau
Republique du Congo
Cuba
Myanmar
Christmas Island
Albania
Fiji
Yemen
South Africa
Madagascar
Sweden
Nicaragua
Ile Europa
Germany
Colombia
Comoro Islands
Ile Tromelin
Ascension
Juan de Nova Island
Angola
Tonga
Palau
Netherlands
Andaman and Nicobar
Wallis and Futuna
Indonesia
Mauritius
Belize
Australia/Indonesia
Brunei
Norfolk Island
Panama
Tokelau
El Salvador
Japan − South Korea Conflict Zone
Mayotte
Maldives
Mozambique
Bangladesh
Bermuda
Solomon Islands
0.0
2.5
5.0
7.5
Pressure
pressure
sst
ocean_acidification
uv
ocean_pollution
shipping
slr
demersal_destructive_fishing
demersal_nondest_high_bycatch
invasives
demersal_nondest_low_bycatch
inorganic
population
pelagic_high_bycatch
plumes_fert
pelagic_low_bycatch
night_lights
plumes_pest
oil_rigs
artisanal_fishing

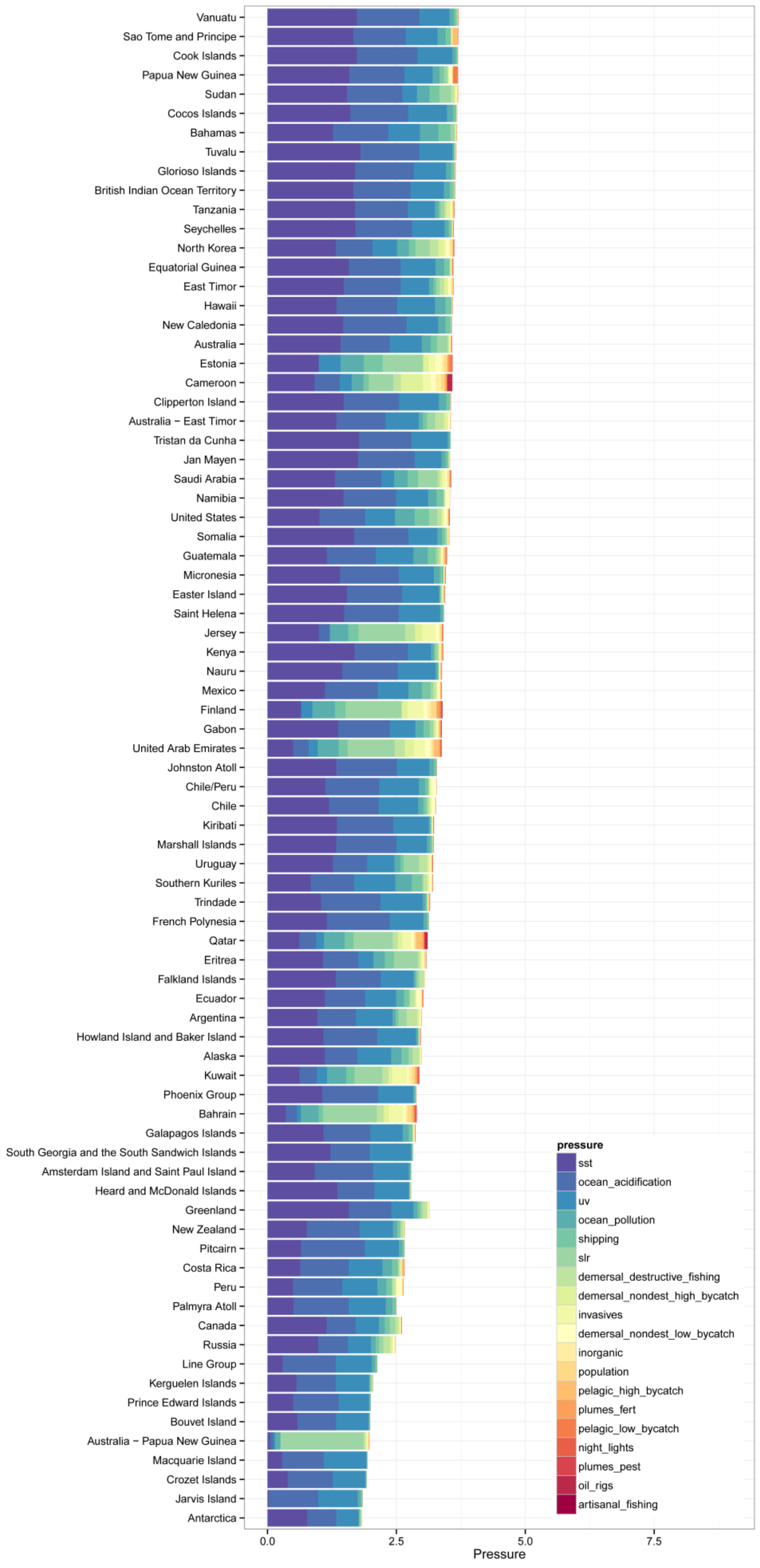
Vanuatu
Sao Tome and Principe
Cook Islands
Papua New Guinea
Sudan
Cocos Islands
Bahamas
Tuvalu
Glorioso Islands
British Indian Ocean Territory
Tanzania
Seychelles
North Korea
Equatorial Guinea
East Timor
Hawaii
New Caledonia
Australia
Estonia
Cameroon
Clipperton Island
Australia − East Timor
Tristan da Cunha
Jan Mayen
Saudi Arabia
Namibia
United States
Somalia
Guatemala
Micronesia
Easter Island
Saint Helena
Jersey
Kenya
Nauru
Mexico
Finland
Gabon
United Arab Emirates
Johnston Atoll
Chile/Peru
Chile
Kiribati
Marshall Islands
Uruguay
Southern Kuriles
Trindade
French Polynesia
Qatar
Eritrea
Falkland Islands
Ecuador
Argentina
Howland Island and Baker Island
Alaska
Kuwait
Phoenix Group
Bahrain
Galapagos Islands
South Georgia and the South Sandwich Islands
Amsterdam Island and Saint Paul Island
Heard and McDonald Islands
Greenland
New Zealand
Pitcairn
Costa Rica
Peru
Palmyra Atoll
Canada
Russia
Line Group
Kerguelen Islands
Prince Edward Islands
Bouvet Island
Australia − Papua New Guinea
Macquarie Island
Crozet Islands
Jarvis Island
Antarctica
0.0
2.5
5.0
7.5
Pressure
pressure
sst
ocean_acidification
uv
ocean_pollution
shipping
slr
demersal_destructive_fishing
demersal_nondest_high_bycatch
invasives
demersal_nondest_low_bycatch
inorganic
population
pelagic_high_bycatch
plumes_fert
pelagic_low_bycatch
night_lights
plumes_pest
oil_rigs
artisanal_fishing

Supplementary Figure 6

Global map of the impact of each of 19 stressors across all 20 potential habitats in 2013. For stressors that primarily occur in coastal areas, we show each for four example zoomed regions.

(see next page)

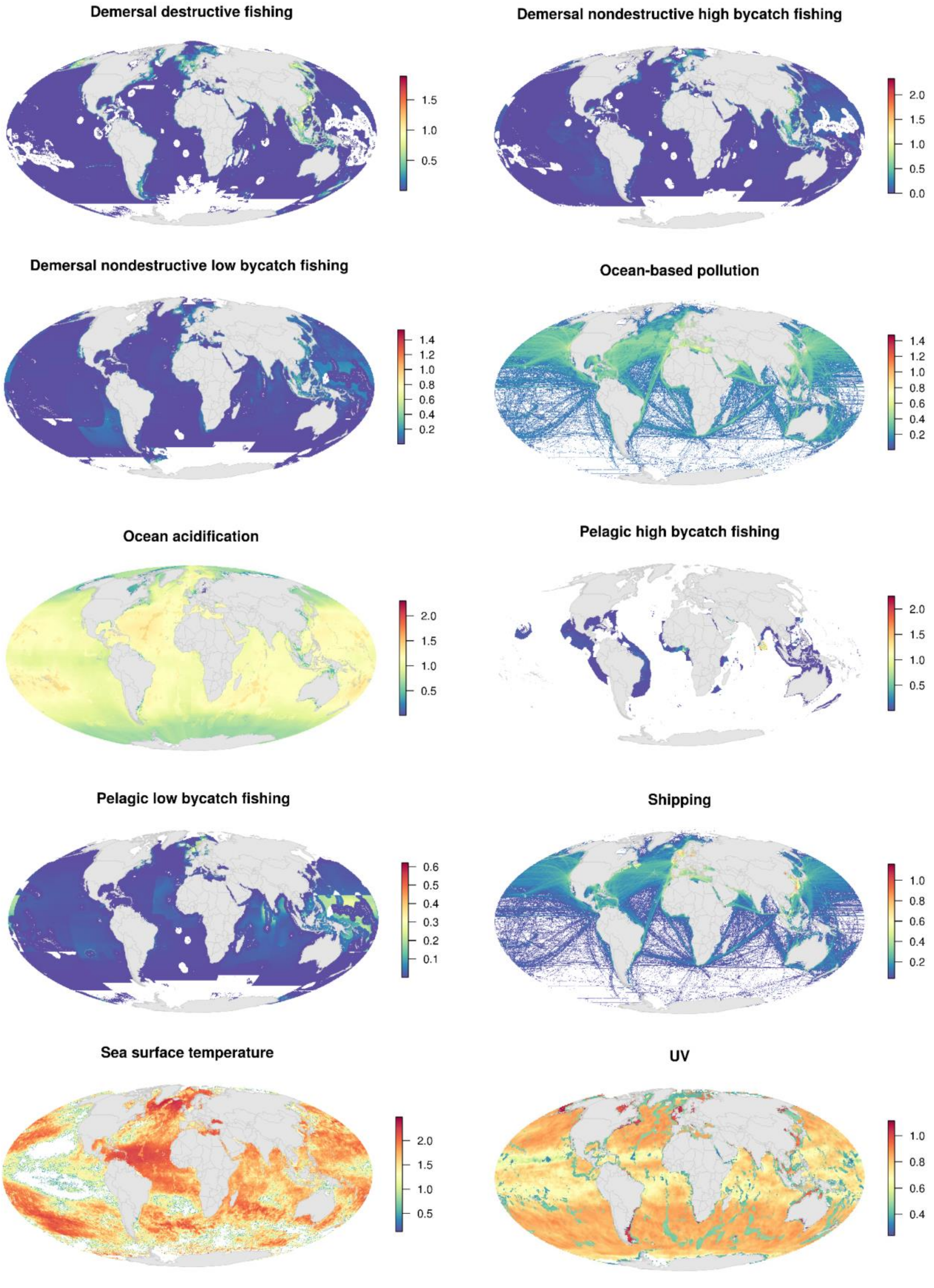
Demersal destructive fishing
0.5
1.0
1.5
Demersal nondestructive high bycatch fishing
0.0
0.5
1.0
1.5
2.0
Demersal nondestructive low bycatch fishing
0.2
0.4
0.6
0.8
1.0
1.2
1.4
Ocean-based pollution
0.2
0.4
0.6
0.8
1.0
1.2
1.4
Ocean acidification
0.5
1.0
1.5
2.0
Pelagic high bycatch fishing
0.5
1.0
1.5
2.0
Pelagic low bycatch fishing
0.1
0.2
0.3
0.4
0.5
0.6
Shipping
0.2
0.4
0.6
0.8
1.0
Sea surface temperature
0.5
1.0
1.5
2.0
UV
0.4
0.6
0.8
1.0

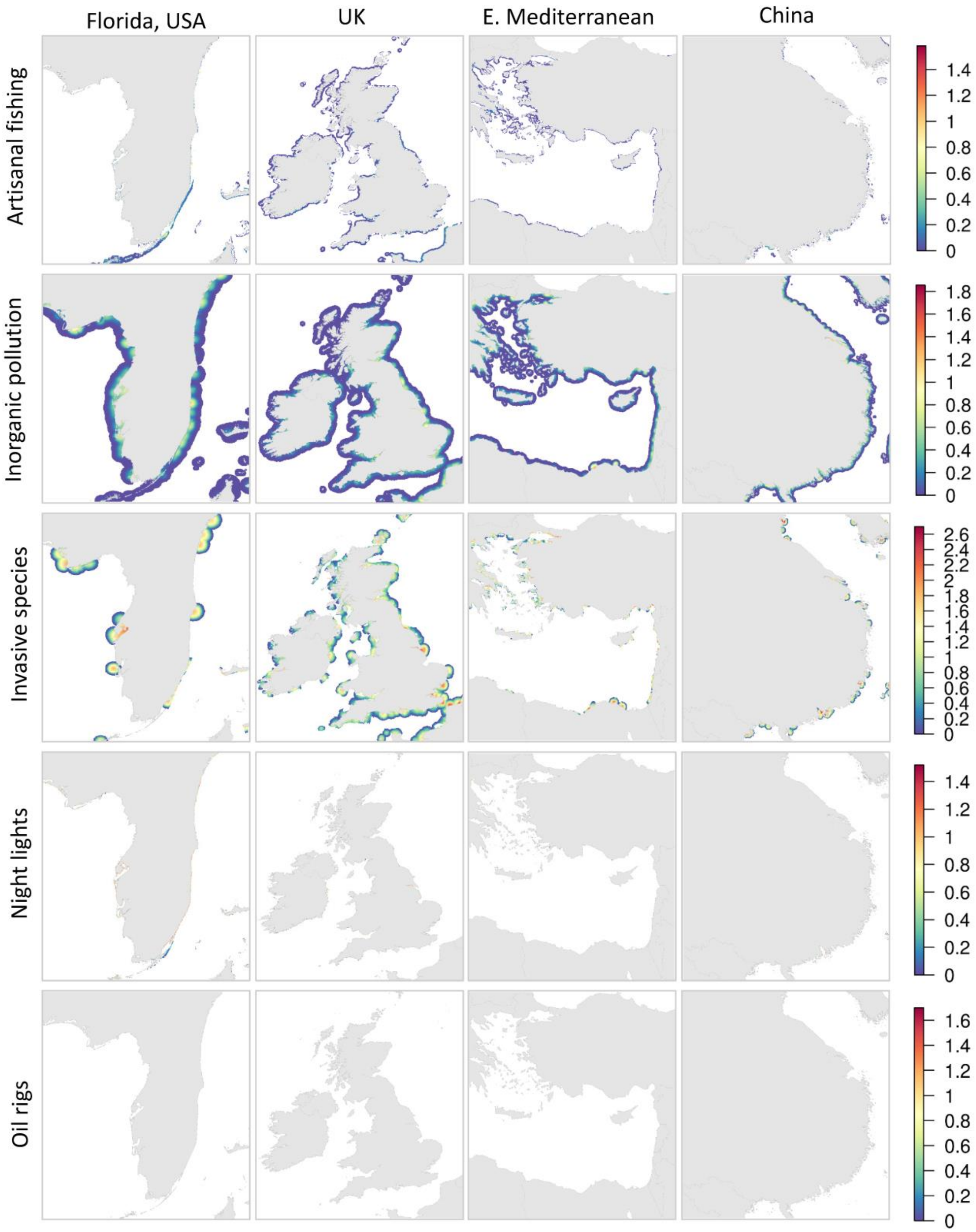
Florida, USA
UK
E. Mediterranean
China
Artisanal fishing
Inorganic pollution
Invasive species
Night lights
Oil rigs
0
0.2
0.4
0.6
0.8
1
1.2
1.4
1.6
1.8
2
2.2
2.4
2.6

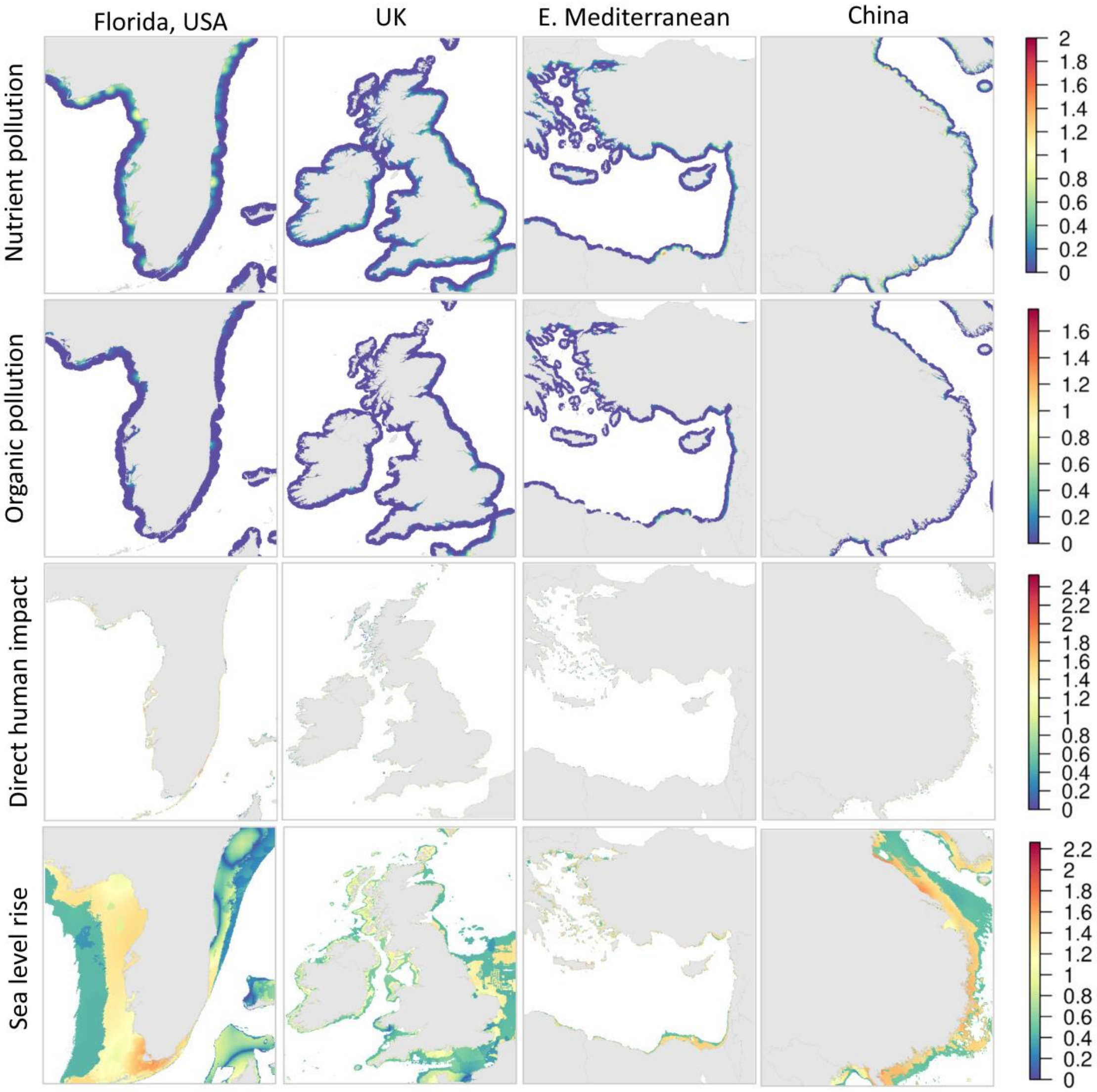

Florida, USA
UK
E. Mediterranean
China
Nutrient pollution
Organic pollution
Direct human impact
Sea level rise
2
1.8
1.6
1.4
1.2
1
0.8
0.6
0.4
0.2
0
1.6
1.4
1.2
1
0.8
0.6
0.4
0.2
0
2.4
2.2
2
1.8
1.6
1.4
1.2
1
0.8
0.6
0.4
0.2
0
2.2
2
1.8
1.6
1.4
1.2
1
0.8
0.6
0.4
0.2
0

Supplementary Figure 7

Global maps of the % contribution of each stressor to overall cumulative impact in 2013. For stressors that primarily occur in coastal areas, we show each for four example zoomed regions (Florida USA; United Kingdom, Eastern Mediterranean, and China).

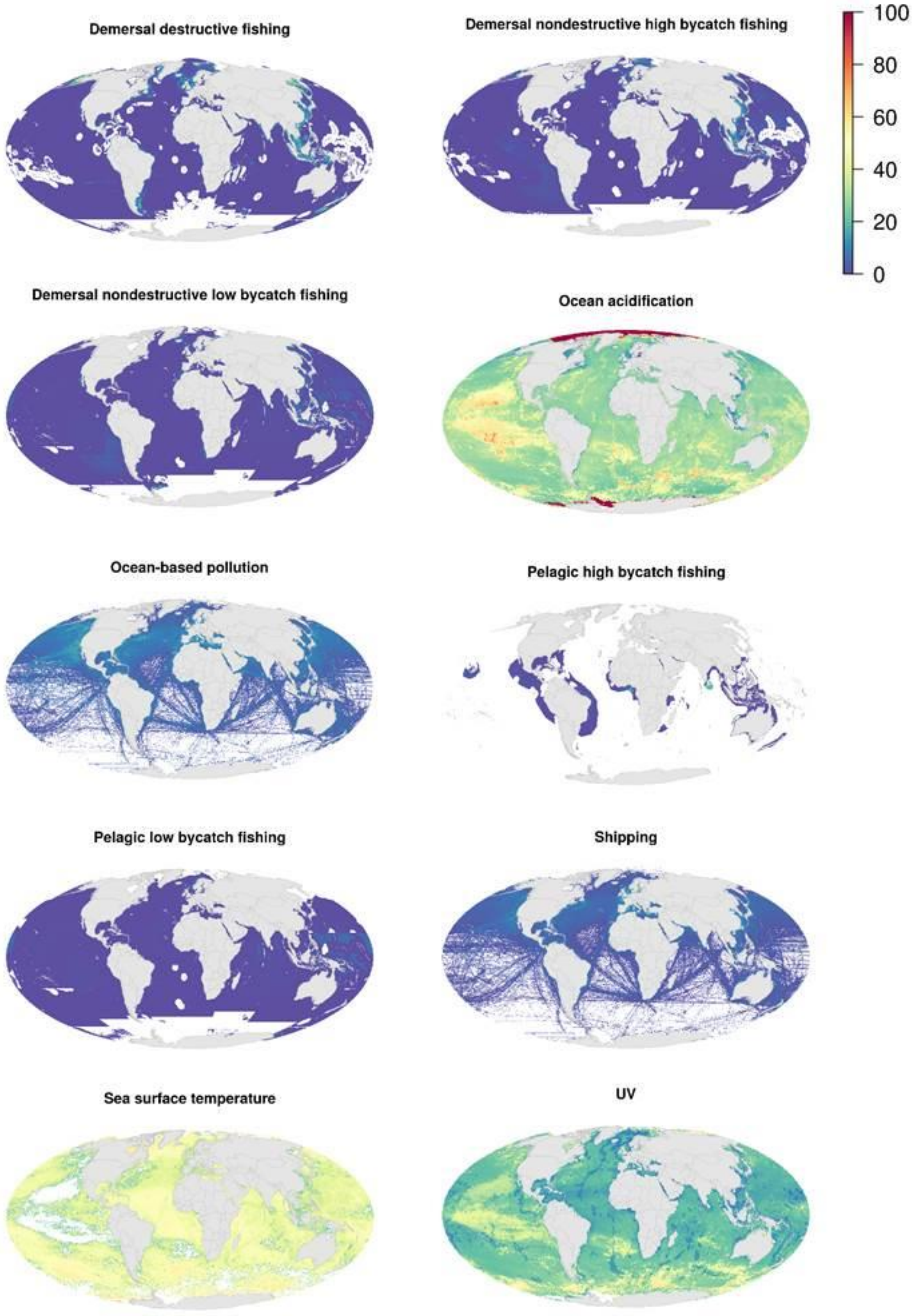

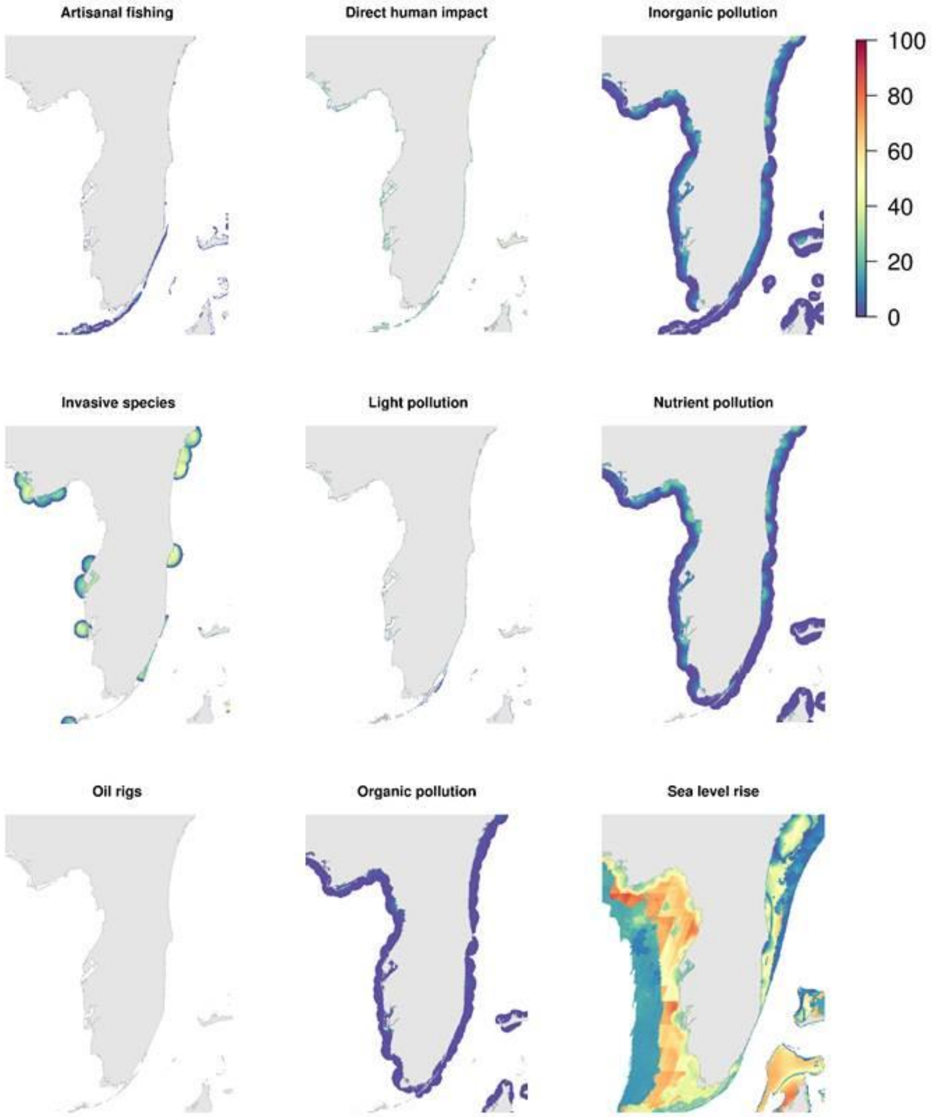

Artisanal fishing
Direct human impact
Inorganic pollution
100
80
60
40
20
0
Invasive species
Light pollution
Nutrient pollution
Oil rigs
Organic pollution
Sea level rise

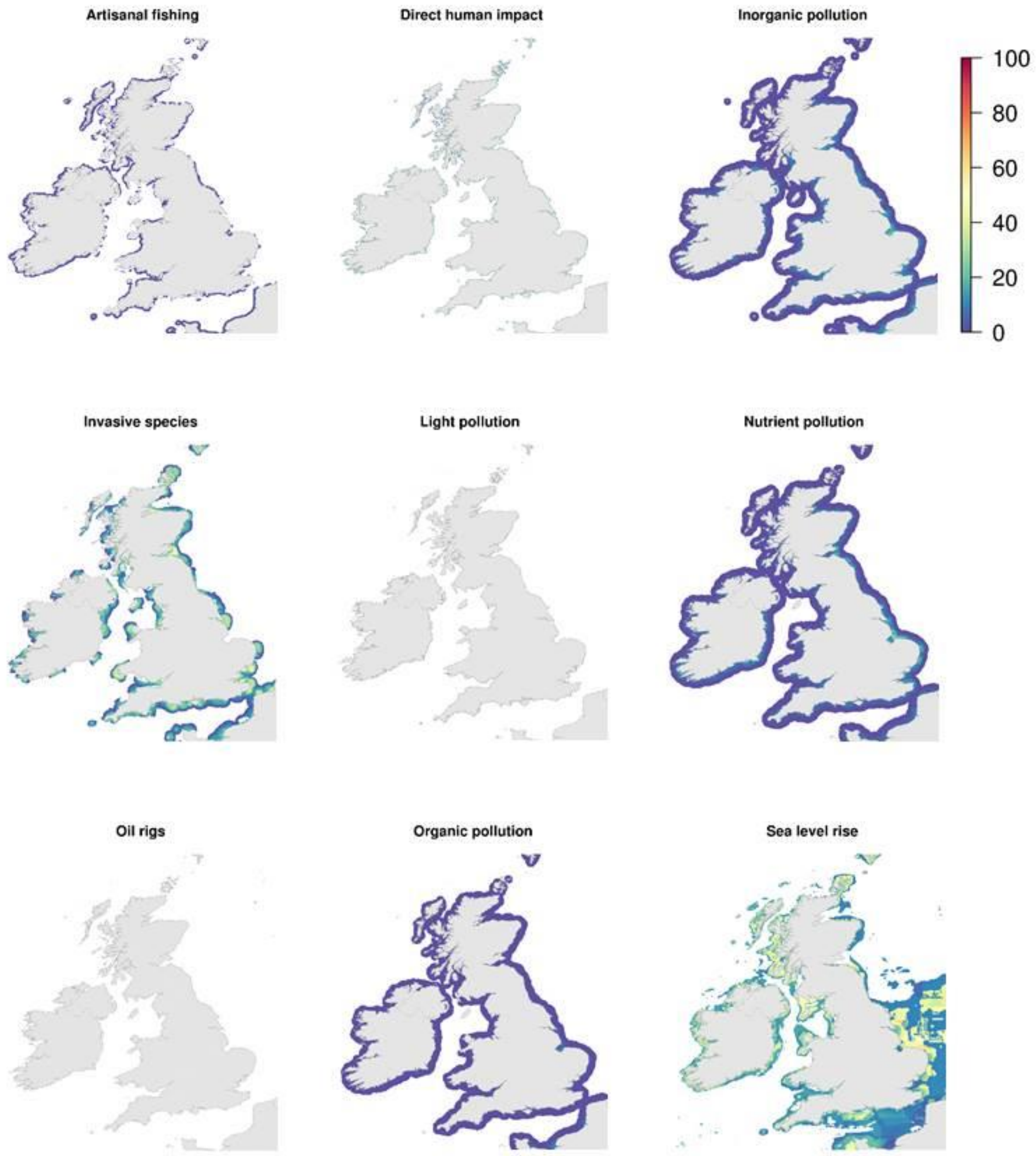
Artisanal fishing
Direct human impact
Inorganic pollution
100
80
60
40
20
0
Invasive species
Light pollution
Nutrient pollution
Oil rigs
Organic pollution
Sea level rise

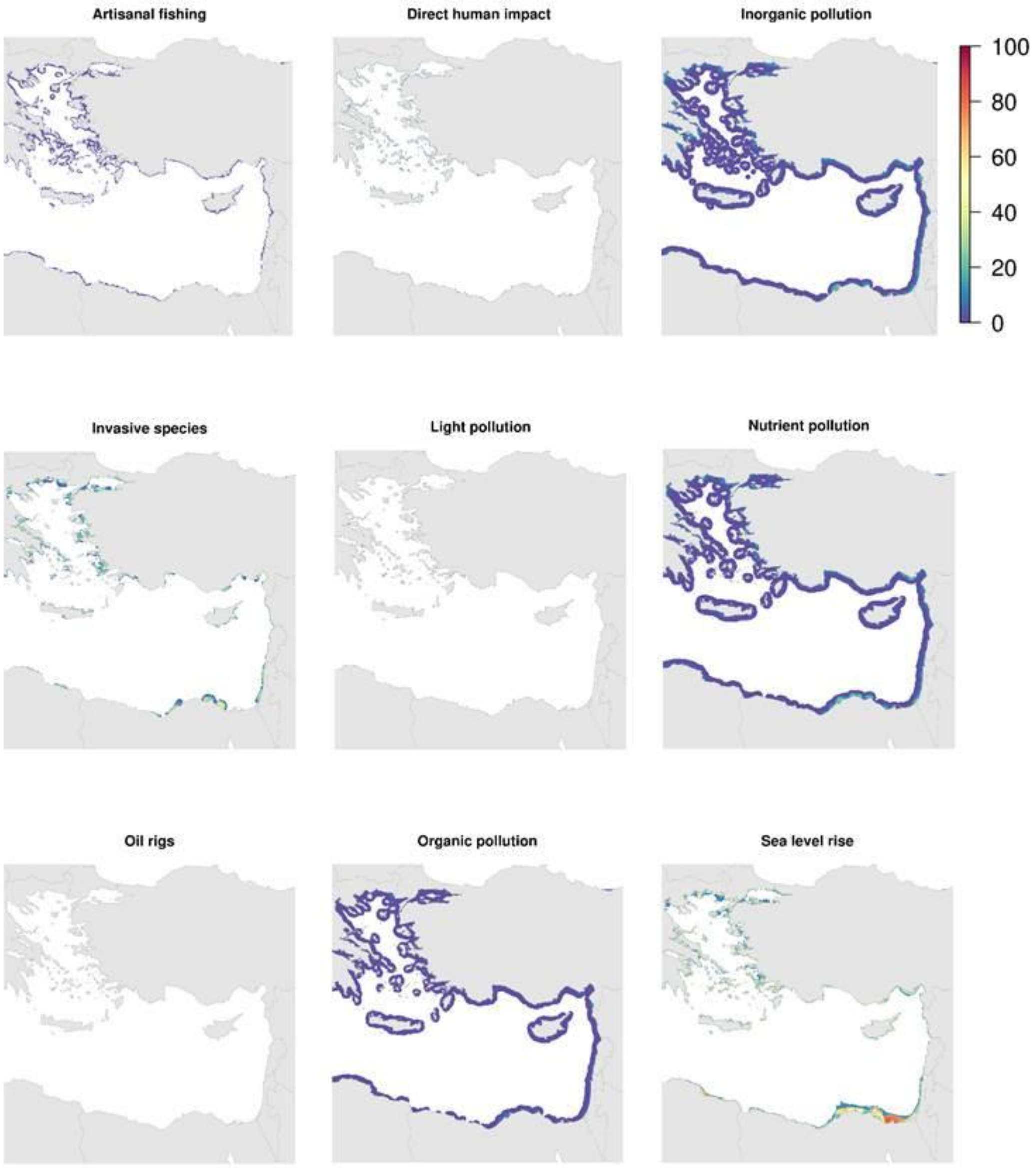
Artisanal fishing
Direct human impact
Inorganic pollution
100
80
60
40
20
0
Invasive species
Light pollution
Nutrient pollution
Oil rigs
Organic pollution
Sea level rise

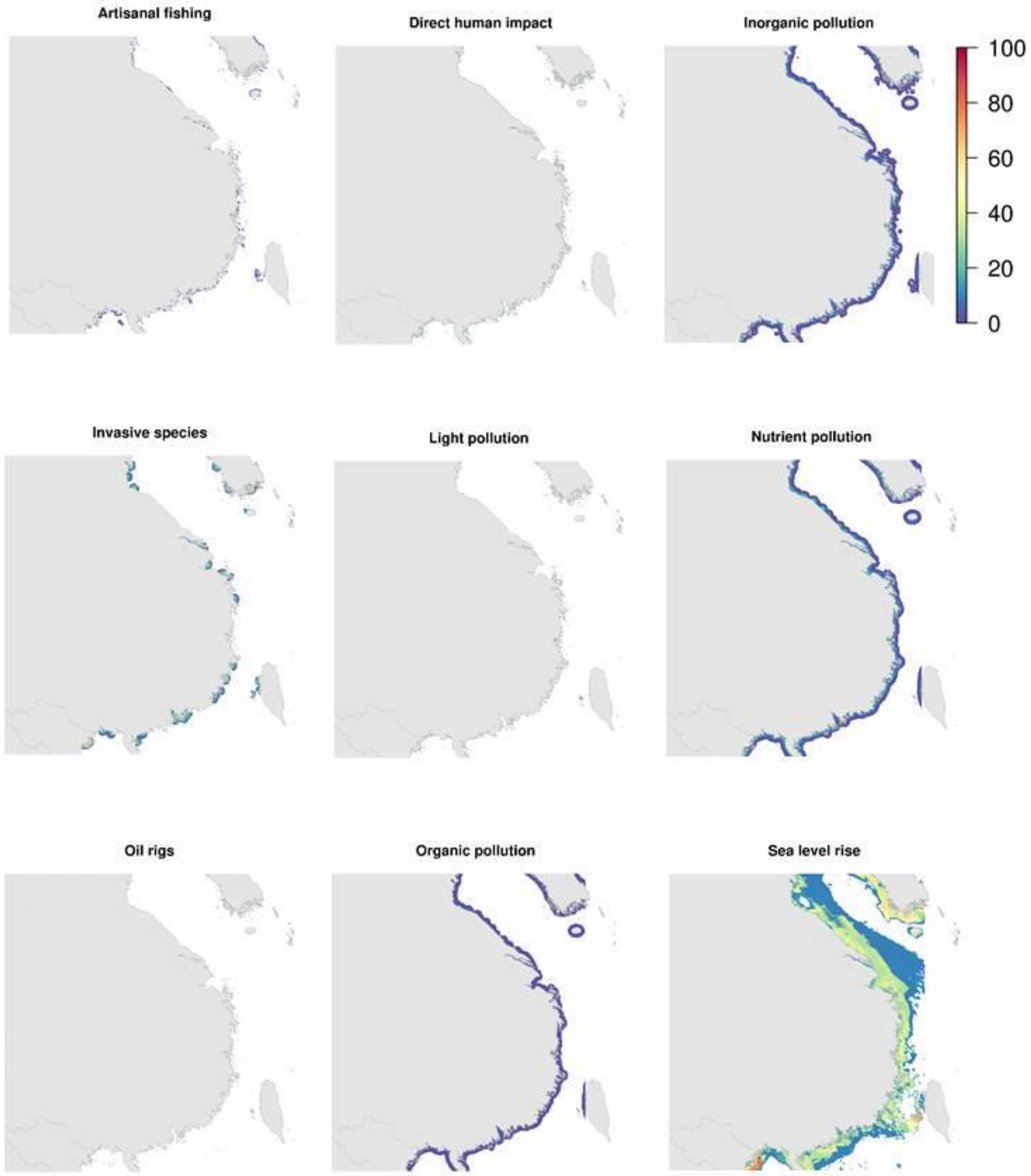
Artisanal fishing
Direct human impact
Inorganic pollution
100
80
60
40
20
0
Invasive species
Light pollution
Nutrient pollution
Oil rigs
Organic pollution
Sea level rise

Supplementary Figure 8

Global map of the cumulative stressor intensity of all 19 stressors in 2013. Cumulative stressor intensity is the sum of the 0-1 normalized stressor magnitude values prior to being multiplied by habitat vulnerability scores to produce cumulative impact scores. Areas of permanent sea ice are shaded white and the area of maximum sea ice extent is masked to indicate where scores are less certain because change in sea ice extent could not be included (see Supplemental Methods).

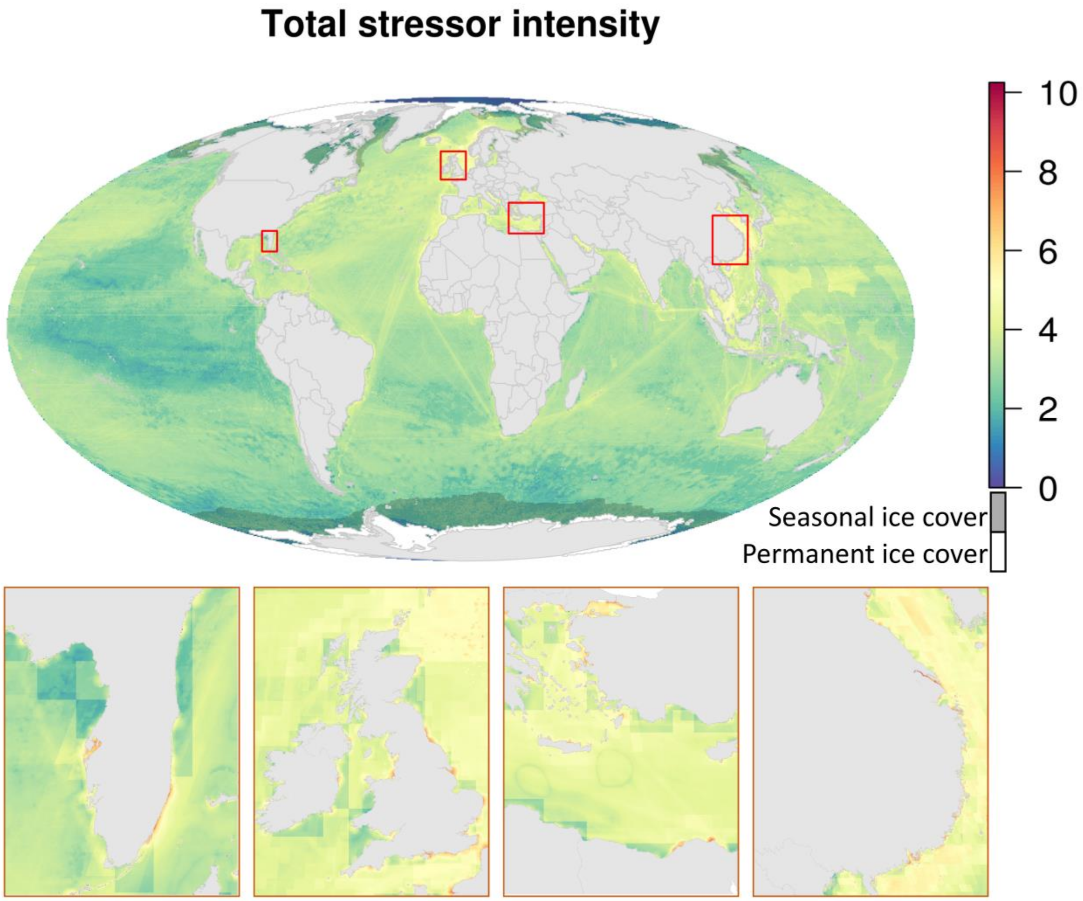

Supplementary Figure 9

Correlation of per-pixel cumulative impact scores from the previous global assessment[1] and the same time period recalculated using the current methods and data sources (r = 0.79). Plot includes 10 million randomly-selected points (roughly 2% of all pixels). Points with much higher 'old' scores compared to new scores are coastal pixels where intertidal habitats are assumed to be everywhere and thus create much higher cumulative impact scores when summed per pixel (old method) rather than averaged per pixel (new method). See 'Methodological Comparisons' above for further details

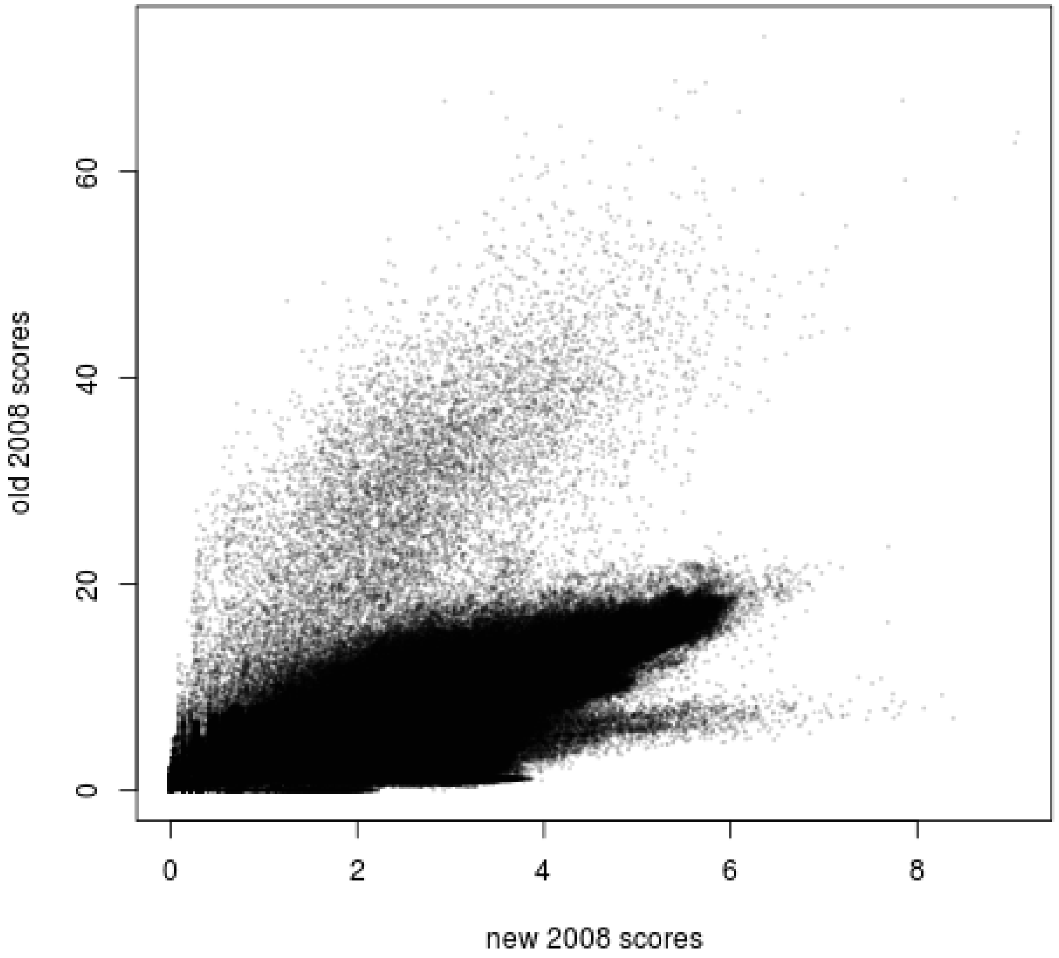

Supplementary Tables

Supplementary Table 1

Full list of data layers used in the analysis. Years used for 2008 analysis are listed for only those layers where temporal comparisons could be made (i.e., data and methods were comparable between time periods).

	Layer	native resolution	2013 Year(s) used	2008 Year(s) used
**STRESSORS**				
Land-based	nutrient pollution	modeled 1km^2	2007-2010	2003-2006
	organic pollution	modeled 1km^2	2007-2010	2003-2006
	inorganic pollution	modeled 1km^2	2000-2001	
	direct human	modeled 1km^2	2011	2006
	light pollution	1km^2	2007-2010	2004-2006
Fishing	demersal, destructive	half-degree	2011	2006
	demersal, non-destructive, high bycatch	half-degree	2011	2006
	demersal, non-destructive, low bycatch	half-degree	2011	2006
	pelagic, high bycatch	half-degree	2011	2006
	pelagic, low bycatch	half-degree	2011	2006
	artisanal	modeled 1km^2	2006	
Climate change	SST anomalies	~21km^2	1985-1990 vs. 2005-2010	1985-1990 vs. 2000-2005
	UV radiation	1 degree	2008-2012	1997-2001
	ocean acidification	1 degree	1870 vs. 2000-2009	

	sea level rise	modeled 0.25 degree	1992-2012	
Ocean-based	commercial shipping	0.1 degree	2003-2011	
	invasive species	modeled 1km^2	2011	
	ocean-based pollution	modeled 1km^2		
	benthic structures	1km^2	2007-2010	2004-2006

HABITATS			Year assessed
Intertidal	rocky intertidal	modeled 1km^2	modeled
	beach	modeled 1km^2	modeled
	mud flats	modeled 1km^2	modeled
	salt marsh	modeled 1km^2	modeled
	mangroves	1km^2	2006
Coastal (<60m depth)	seagrass beds	1km^2	2006
	shellfish reefs	modeled 1km^2	modeled
	coral reefs	1km^2	2006
	rocky reefs	1km^2	2005
	kelp forests	modeled 1km^2	modeled
	shallow soft bottom	1km^2	2005
Offshore (>60m depth)	continental shelf, hard bottom (60-200m)	1km^2	2005
	continental shelf, soft bottom (60-200m)	1km^2	2005
	continental slope hard bottom (200-2000m)	1km^2	2005
	continental slope soft bottom (200-2000m)	1km^2	2005
	deep benthic, hard bottom (>2000m)	1km^2	2005
	deep benthic, soft bottom (>2000m)	1km^2	2005
	shallow pelagic (<60m)	1km^2	2013
	deep pelagic (>60m)	1km^2	2013
	seamounts	1km^2	2004

Supplementary Table 2

Ecosystem vulnerability weights for each stressor. Values are modified from previous values[2]

	Rocky Intertidal	Intertidal Mud	Beach	mangroves	Salt Marsh	coral	seagrass	Kelp Forest	rocky reef	Susp.-Feeder [illegible]	shallow soft	soft shelf	hard shelf	soft slope	hard slope	soft deep	hard deep	seamounts	pelagic	pelagic_deep
nutrients	1.6	1.6	0.4	1.8	1.9	1.8	2.1	0.4	1.6	1.4	2.0	1.4	1.7	2.0	0.6	1.3	0.0	0.0	1.2	0.0
org_pollution	2.1	2.8	0.1	1.4	1.7	1.2	1.0	1.0	2.2	2.8	1.2	1.4	0.0	2.0	0.2	1.7	0.0	0.0	1.9	1.6
inorg_pollution	2.1	1.6	0.6	0.5	2.0	0.7	0.8	0.0	2.2	2.7	1.5	2.1	0.2	2.1	0.2	1.8	0.0	0.0	2.3	1.6
light_pollution	1.4	1.4	2.0	0.9	1.8	1.0	0.5	0.5	0.7	1.0	0.5	0.0	0.0	0.0	0.0	0.0	0.0	0.0	0.4	0.0
direct human	2.8	2.2	2.7	3.3	1.6	2.3	2.5	1.6	2.5	3.0	2.0	1.1	2.9	0.0	0.0	1.6	0.0	0.0	0.9	0.0
dem_dest	1.2	1.4	0.2	0.0	1.0	1.2	0.2	1.5	2.7	3.1	2.1	3.0	3.1	3.2	2.8	2.3	3.0	3.5	2.1	0.8
dem_nd_hb	0.8	1.9	0.9	0.9	1.0	1.6	1.1	2.1	2.9	0.7	2.1	2.0	3.2	2.3	2.4	2.0	0.0	0.0	1.6	0.0
dem_nd_lb	0.6	1.4	0.7	0.7	0.8	1.2	0.8	1.6	2.2	0.5	1.6	1.5	2.4	1.8	1.8	1.5	0.0	0.0	1.2	0.0
pel_hb	0.9	0.0	0.1	0.0	0.5	0.5	0.0	0.0	2.6	0.0	0.0	1.1	2.8	0.2	0.0	1.6	0.0	0.0	3.0	2.2
pel_lb	0.0	0.0	0.0	0.0	0.4	0.7	0.0	0.0	2.6	0.0	0.6	0.8	2.8	0.2	0.0	0.5	0.0	0.0	2.2	0.6
artisanal	1.3	0.4	0.7	1.7	0.6	2.3	0.3	0.8	2.2	1.0	0.0	0.9	1.9	0.0	0.4	0.3	0.0	0.9	1.0	0.0
sst	2.8	1.4	0.6	2.4	1.4	2.8	2.1	2.0	1.9	0.8	0.5	2.5	2.9	2.3	0.9	2.5	1.5	1.8	3.3	2.3
uv	0.9	1.3	0.0	0.2	1.1	0.8	0.5	0.1	0.7	0.0	0.3	1.9	1.8	0.0	0.0	1.3	0.0	0.0	1.5	0.0
acid	0.9	1.0	0.0	1.2	1.3	1.1	1.4	0.0	1.1	0.7	0.1	1.7	2.5	2.1	1.6	2.2	2.7	2.7	1.8	0.0
sea level rise	2.5	1.9	2.1	3.0	3.1	2.4	2.6	1.6	1.5	1.8	2.2	0.0	0.0	0.0	0.0	0.0	0.0	0.0	0.0	0.0
invasives	2.8	2.9	0.9	1.0	2.8	1.5	1.2	1.3	2.5	2.6	2.7	1.6	1.5	0.2	0.5	1.5	0.0	0.0	2.3	0.0
ocean_pollution	1.3	0.8	0.5	1.2	1.2	1.2	0.5	0.1	1.7	0.0	1.1	1.2	0.3	1.4	1.7	2.3	1.2	1.2	1.7	0.4
shipping	0.3	1.9	1.9	2.0	1.4	1.5	1.9	0.0	1.4	0.0	0.3	1.7	0.9	0.1	1.0	0.9	0.0	0.0	1.9	0.0
oil rigs	1.0	0.9	0.8	1.3	0.9	0.5	1.6	0.0	1.7	0.4	0.1	0.5	2.1	1.6	2.2	1.9	1.6	1.4	1.5	0.0

Supplementary Table 3

Relationships between current cumulative impact (2013) and change in cumulative impact (2013 minus 2008) and ocean area (EEZ area, ln km^2), coastal population (ln, 25 miles inland), and coastal population trend based on linear models.

		Estimate	Std. Error	t-value	P-value
2013 Cumulative impact[¥], $R^2=0.38$, $F_{2,198}=59.92$, N=201					
	Intercept	5.32			
	Ocean area (ln km^2)	-0.192	0.0187	-10.29	<0.001
	Coastal population (ln)	0.075	0.0132	5.66	<0.001
Change in cumulative impact (2013 minus 2008) [±], $R^2=0.08$, $F_{2,198}=9.16$, N=201					
	Intercept	-0.190			
	Ocean area (ln km^2)	0.036	0.0131	2.76	0.006
	Coastal population trend	7.300	2.2227	3.28	0.001

Supplementary Table 4

Percentage of ocean area with decreased/increased/no change from 2008 to 2013.Area (in assessment pixels, which are roughly 1km^2) previously unaffected by each stressor but now experiencing some level of it (‘new area’), areas previously affected but no longer experiencing the stressor (‘area reduced’), and the global average change in impact score of each stressor between 2008 to 2013 assessments. Results for cumulative impact are also provided.

Category	Stressor	% decrease	% increase	% No Change	New area	% new area	Area reduced	% area reduced	global average change
Fishing	Demersal destructive	70.946	9.600	19.454	0	0	0	0	-0.00473
	Demersal, non-destructive, high bycatch	73.683	8.489	17.828	0	0	2095	0.001	-0.00452
	Demersal, non-destructive, low bycatch	77.493	9.685	12.822	0	0	0	0	-0.00413
	Pelagic, high bycatch	0.036	0.029	99.935	0	0	0	0	-0.0008
	Pelagic, low bycatch	0.001	0.002	99.997	0	0	0	0	-0.00295
Ocean-based	Light pollution	7.976	1.412	90.611	16,143	0.004	19,168	0.005	-9E-07
	Benthic structures	74.007	8.939	17.055	7,616	0.002	5,938	0.001	0.000006
Land-based	Nutrient pollution	1.308	1.360	97.332	490,860	0.118	394,028	0.095	0.00025
	Organic pollution	0.885	1.002	98.112	424,784	0.102	358,552	0.086	0.000009
	Direct human impact	0.112	0.159	99.729	25,301	0.006	25,762	0.006	0.00001
Climate change	SST anomalies	22.222	62.269	15.509	69,712,874	16.752	17,936,214	4.310	0.29813
	UV anomalies	46.177	48.942	4.881	5,431,251	1.305	4,954,971	1.191	0.00236
	Cumulative impact	32.058	65.771	2.171	21,984	0.005	15,578	0.004	0.28338

Supplementary Table 5

Summary of the number of 1km^2 pixels in each category of cumulative pressure (high, low, neither) and trend in impact (increasing, decreasing, neither).

**Pressure**	**Trend**	**# cells**
High	Increasing	20784803
High	Decreasing	13755296
Neither	Neither	331329142
Low	Increasing	8073845
Low	Decreasing	42215891

Supplementary Table 6

Online sources of port throughput data and the number of port records (N) reported on each site. The total number of records is greater than the resulting dataset because of the removal of replicate ports.

**Organization**	**Website**	**N**
American Association of Port Authorities	http://www.aapa-ports.org/	324
Comisión Económica para América Latina y el Caribe	http://www.eclac.org	100
Containerization International (CI) Yearbook	http://europe.nxtbook.com/nxteu/informa/ci_yearbook2011	100
Dynamar	https://www.dynamar.com/	114
Eurostat	http://appsso.eurostat.ec.europa.eu/	1,103
Ports Australia	http://www.portsaustralia.com.au/	46
UK Department of Transportation	https://www.gov.uk/government/publications/	159
**Total**		**1,946**

Supplementary Methods

Stressor Data

Methods for preparing stressor data that were unchanged from the previous analyses (Supplementary Table 1) are described in detail elsewhere[1]. Stressors with updated data were prepared using more recent years from the same data source. In these cases we describe the new data but do not elaborate methods. We primarily focus on describing those layers where updating required new methods.

We were unable include change in sea ice extent as a stressor because there remains large uncertainty on if or how changes in sea ice extent affect marine ecosystems[3]. Our framework for assessing cumulative impacts to habitats requires a vulnerability assessment for how each habitat uniquely responds to each stressor. We do not have these vulnerability scores for change in sea ice extent as a stressor to habitats. To acknowledge the uncertainty created by not including sea ice as a stressor, we mask areas within maximum sea ice extent in all figures where multiple stressors are assessed.

LAND-BASED STRESSORS

Nutrient pollution

To allow for direct comparison between past[1] and current assessments (for 2013), we used input data from 2003-2006 for the 2008 assessment, as was done previously[1] and input data from 2007-2010, the most recent available, for the 2013 assessment. In particular, we used FAO data on annual country-level fertilizer use, averaged over the time periods, with missing values filled using a linear regression model of fertilizers as a function of pesticides (gaps: N=4; regression: $R^2 = 0.72$) when pesticide data were available or agricultural GDP (gaps: N=22; regression: $R^2 = 0.62$) when not. These country-level average fertilizer values were then dasymetrically distributed over a country's landscape using global land cover data from the years 2005 (for the 2003-2006 time period) and 2009 (for the 2007-2010 time period), derived from the Moderate Resolution imaging Spectroradiometer (MODIS) instrument at ~500m resolution (following methods described elsewhere[1] but with updated MODIS data not available previously). These values were then aggregated by ~140,000 global basins (as described elsewhere[1]), and diffusive plumes were modeled from each basin's pourpoint. The final non-zero plumes (about ~76,000) were aggregated into ~1km^2 rasters in the Mollweide projection (WGS1984 datum) to produce a single plume aggregated pollution raster. These raw values were then log[X+1] transformed and normalized to 0-1. A simple visual and pixel-count comparison of the agriculture land cover classes shows that, globally, these classes have not changed much during the two time periods (at the 500m resolution of the data). However, global fertilizer consumption has shown a small, but notable increase (roughly 4-8% globally over the study time period).

To assess how well our modeled nutrient pollution values matched estimates from more complex watershed process models, we correlated dissolved organic nitrogen and phosphorous (DON+DOP) values from the ~6300 modeled values from the GlobalNEWS project[4] with our nitrogen pollution values for the corresponding watersheds. Values were

highly significantly correlated for the largest 4% of watersheds (R^2=0.55, p<0.0001; N=145) and the largest 20% of watersheds (R^2 = 0.45, p<0.001; N=729), and significant but with weaker correlation for all watersheds (R^2=0.23, p<0.001; N=3645). At the scale of the U.S. west coast the correlation between empirical river output and our modeled output was remarkably high (R^2 = 0.97)[5], further supporting our approach as a means to capture relative nutrient pollution for small watersheds as well as large ones.

Organic pollution

We use pesticides as a proxy measure for organic pollution. These methods are nearly identical to the ones described above for nutrient pollution. Missing pesticide values were filled using a linear regression model of pesticides as a function of fertilizers (gaps: N=69; regression: R^2 = 0.72) when fertilizer data were available or agricultural GDP (gaps: N=22; regression: R^2 = 0.82) when not. Global pesticide consumption has shown a small, but notable increase (roughly 4-8% globally over the study time period).

Inorganic pollution

All data and methods remain the same as previously described[1], such that this data layer is identical in both the 2008 and 2013 assessments.

Direct human impact

As was done previously[1], we modeled direct human impact on the coast as the sum of the coastal human population, defined as the number of people within a moving circular window around an arbitrary focal coastal cell of radius 10 km on the basis of the LandScan 30 arc-second population data (http://www.ornl.gov/sci/landscan/). We used 2006 LandScan data for the previous (2008) assessment and 2011 data for the current (2013) assessment. This value was then assigned to the adjacent ocean cell since this driver primarily affects intertidal and very nearshore ecosystems.

Light pollution

We used the DMSP stable night lights data layers obtained from NOAA NGDC (http://ngdc.noaa.gov/eog/dmsp/downloadV4composites.html). The original layers contained non-calibrated radiance values. We applied a calibration technique developed elsewhere[6]. The original raster digital number (DN_o) values of 0-63 were calibrated to produce adjusted DN (DN_a) values using the following intercalibration formula:

$$DN_a = C_0 + C_1 * DN_o + C_2 * DN_o^2 \quad (1)$$

with the three calibration coefficients (C_0, C_1, and C_2) set from recent analyses[7]. This formula is based on the assumption that a reference area (around Sicily) has not changed much in light output in recent years. Lowest values in DN_a (non-lit areas) were replaced with zeros. This technique, while not perfect, does allow for better comparison between different satellites and time periods.

To minimize error, we chose to use only one satellite, F16, for the period spanning 2004-2009 (we also tried including more satellites and expanding the time period, but the results were similar). We took the mean of calibrated values from 2004-2006 for the previous assessment time period (2008) and values from 2007-2009 for the new assessment (2013). Calibration coefficients from 2010 were not available, so this year was excluded.

FISHING STRESSORS

Commercial fishing

These layers were initially developed previously[1] to provide information on spatially-explicit catch by 5 broad categories of fisheries and associated gear types: demersal destructive (e.g., bottom trawl), demersal non-destructive high bycatch (e.g., pots, traps), demersal non-destructive low bycatch (e.g., hook and line), pelagic high bycatch (e.g., long-line), and pelagic low bycatch (e.g., hook and line; see [1] for gear classifications). The layers were recently revised for the annual update of the Ocean Health Index and are described elsewhere[8]. We used those same methods and briefly describe them below.

Catch (landings) data are only a proxy for the potential impact of fishing on marine ecosystems because it is the effort to land the catch that determines impact. For example, an area with low fish densities would have to be trawled many more times to harvest an equal catch to an area with higher fish densities. Consequently, fishing stressors may be underestimated in areas with lower densities and overestimated in areas with higher densities. Annual wild caught fisheries statistics were updated by the FAO for each of its spatial reporting units, i.e. the FAO major fishing area, but finer-scale allocation of catch to half-degree grids and assignments to one of the five gear types had not been updated since the original publication[1]. Thus, updating these data layers required a two-step process. First, the difference was calculated between total annual FAO wild caught fisheries catch for recent years and past years, in each FAO major fishing area. Second, these differences were used to update the values assigned to half-degree grids and gear type categories. The assumption was that the relative proportion of biomass caught by each gear type had not changed within each FAO fishing area since the previous estimate, so any increase/decline in total harvested biomass was translated into an equal increase/decline across all gear categories in that fishing area.

Updating annual catch data: We used catch data per FAO fishing area to estimate the percent change in total annual catch per region, calculated as the difference between the most recent reporting period (2009 to 2011) and the period (1999 to 2003) previously used[1], divided by the original period (1999 to 2003). Each of the five gear type 1km^2 raster layers[1] was then multiplied by the percent change of the corresponding FAO fishing area to create estimates of current spatially-explicit catch by gear type. Although this calculation requires the unlikely assumption that the proportional amount of catch per gear type per has remained the same over the past 10 years across the entire FAO area, we felt it better to make this assumption than to assume fishing pressure has not changed at all.

Artisanal fishing

All data and methods remain the same as originally developed[1], such that this data layer is identical in both the 2008 and 2013 assessments.

CLIMATE CHANGE STRESSORS

Sea surface temperature (SST) anomalies

Sea surface temperature (SST) data were obtained from version 4 of the Coral Reef Temperature Anomaly Database (CoRTAD; Refs [9,10], which was produced by the NOAA National Oceanographic Data Center using 4.6 km (nominally 21 km^2 at the equator) Advanced Very High Resolution Radiometer (AVHRR) Pathfinder Version 5.2 SST data[11] from 1982-2010 (www.nodc.noaa.gov/SatelliteData/CoRTAD). Because SST measurements are less reliable where there is persistent ice, an ice mask was created to identify places near the poles that were almost always covered by significant sea ice. The ice mask was generated primarily from the OSI/SAF Global Daily Sea Ice Concentration Reprocessing Data Set (accession.nodc.noaa.gov/0068294), which was regridded and made available in the Pathfinder V5.2 dataset. In Pathfinder, when the OSI/SAF data are unavailable, the sea ice concentrations from the NCDC Daily OI SST data[12] were included. For each day of the climatological year (1 through 366), the daily sea ice fraction from all of the years was read in and averaged to create a daily, sea-ice fraction climatology. Grid cells that contained a sea ice fraction greater than 0.15 for all of the averaged days of the year were masked out of the analysis.

An anomaly was defined as exceeding the standard deviation (SD) of SSTs from the climatology for that location (i.e., grid cell) and week of the year. The climatology was defined as a weekly average for each ~4 km grid cell from 1982-2010 (see Ref [1] for original methods description). The frequency of anomalies was calculated for each year in the dataset. The difference was then quantified between the number of anomalies in the 5 most recent years (2005-2010) and the 5 years that were defined as the earliest period in the previous analysis (1985-1990). The metric of change in SST anomalies from previous work[1] was also recreated, which measured the difference in anomaly frequency from 2000-2005 compared to 1985-1990.

There are three main differences between the dataset used previously[1] and the version 4 CoRTAD data used for this analysis. Since that publication, the Pathfinder data used to generate the version 4 CoRTAD has been updated to use a different and higher resolution reference[12] dataset to help identify bad data (data are removed if they are +5/-2°C from the reference dataset). The new version now more accurately resolves strong temperature gradients like those that occur along major boundary currents or near the coast. By adding in new years to the dataset, a new climatology is also created, so the standard deviation thresholds have changed. The result could be a decrease in the overall frequency of anomalies compared to the older dataset. Anomalies were calculated in only the positive (warming) direction, as was done previously[1].

Temporal dynamics: Changes in regional ocean climates, such as occur with the El Niño Southern Oscillation (ENSO) or other major oscillations (e.g., Pacific Decadal Oscillation, North Atlantic Oscillation), have a direct effect on local and regional patterns of SST anomalies. By averaging anomalies over a five year period, we minimize the potential effect of annual variability. Longer-term climatological patterns are much harder to address. Ideally, we would be able to fully factor out changes in local SST due to regional climatic oscillations, but to do this we would need many decades of data and more robust models to predict local and regional changes due to such oscillations (so that human driven change could be isolated for each patch of ocean). These data and models are currently not available.

Some of the local-scale changes in SST anomalies could therefore be due to natural variation and not a consequence of human activities. The most notable potential location for such an effect in our results is the Northeast and Eastern Tropical Pacific (see Supplementary Figure 9), where there were large decreases in anomalies between the two assessment periods. However, the Pacific Decadal Oscillation was in a cool phase over the period of our two assessment windows (2000-2005 and 2005-2010)[13], and so for this current analyses the observed changes in SST anomalies do not appear directly driven by long-period climate oscillations such as PDO.

UV radiation anomalies

We followed methods developed previously[1]. We used Aura/OMI satellite data (disc.sci.gsfc.nasa.gov/data-holdings/PIP/erythemal_uv_irradiance.shtml) to obtain daily Local Noon Erythemal UV Irradiance (mW/m^2). The Aura/OMI satellite provides data at 1x1 degree resolution from September 2004 through present, spanning 180 degrees latitude and 360 degrees longitude.

We calculated the number of positive monthly anomalies during a 5-year time period (2008-2012). Daily irradiance values were averaged into 60 monthly mean UV values for the five year period. Mean monthly UV values exceeding the baseline mean plus one standard deviation were labeled as anomalous pixels. The number of positive anomalies ranged from 0-9. These were then transformed and normalized from 0-1 for each raster cell:

$$\frac{log10(x+1)}{log10(\max(x+1))} \quad (2)$$

Ocean acidification

All data and methods remain the same as previously reported[1], such that this data layer is identical in both the 2008 and 2013 assessments.

Sea level rise

The sea level rise data are based on Nicholls and Cazenave[14], which can be viewed at: http://www.aviso.altimetry.fr/en/data/products/ocean-indicators-products/mean-sea-level/products-images.html. The original data

(MSL_Map_MERGED_Global_IB_RWT_NoGIA_Adjust.nc) were reprojected into Geographic WGS84, 0.25x0.25 degree resolution pixels, and then the rate of sea level rise (mm/yr) per pixel was calculated across the time span of the dataset (Oct 1992 through Dec 2012). This calculation produced a minimum value of -34 mm/yr and maximum value of 30 mm/yr. To produce a value of net change in sea level, these rates were multiplied by the duration of the time series (20.167 yrs), resulting in a minimum value of -685.63 mm and maximum value of 607.01 mm. For comparison to other pressure layers, these values were then rescaled to the largest absolute value, in this case preserving negative values such that the new values ranged -1.0 to 0.885. Note that this global data set does not take into account Glacial Isostatic Adjustment (GIA) of ~-0.3 mm/yr, although this does affect the normalized values (addressed in website noted above). For the purposes of calculating pressures, negative values were set to zero (i.e. no negative pressure), such that only positive sea level rise values mattered.

OCEAN-BASED STRESSORS

Benthic structures (oil rigs)

To capture the presence of oil rigs, we applied a 0/1 binary threshold to our night pollution layers. After masking out land areas, most ocean stable lights are assumed to be oil rigs. However, this method still leaves some erroneous non-flare lights. We used NGDC DMSP gas flare data from 2006/2007 to mask out non-flare areas from our oil rigs layers for both time periods (http://www.ngdc.noaa.gov/eog/data/web_data/gasflares_v2/). This leads to a smaller set of oil rig pixels, but improves on accuracy over the previous method[1].

Shipping

Vessel identity and location information was obtained using two approaches. (1) Over the past 20 years, 10-20% of the vessel fleet has voluntarily participated in collecting meteorological data for the open ocean, which includes location at the time of measurement, as part of the Volunteer Observing System (VOS). (2) In order to improve maritime safety, in 2002 the International Maritime Organization SOLAS agreement required all vessels over 300 gross tonnage (GT) and vessels carrying passengers to equip Automatic Identification System (AIS) transceivers, which use the Global Positioning System (GPS) to precisely locate vessels.

In the previously developed shipping layer[1], a single year sample of the VOS data was used for analysis. These data ignored vessel type, and included observations from only 12% of the vessel fleet. The ships included are a spatially- and statistically-biased sample of the population, making the modeled results somewhat misleading. Here, we have instead adopted the model outputs developed elsewhere[15]. We collapsed down the detailed analysis from that study into eight broad classes of vessels: authority, cargo, fishing, high-speed, passenger, pleasure, support, tanker and an ‘other’ class. The vessel classes which move globally (cargo, tanker, and passenger) are required to carry AIS transceivers, and in these three classes 60-70% of the total vessel fleet was observed using AIS. The resulting data layer is primarily composed of these vessel classes in both

the AIS and VOS data sources, and is almost exclusively these ship types in the open ocean. We used a simple linear average of the two data sources, producing a final model resolved for the whole ocean at a resolution of 0.1 decimal degrees (~11km).

Both the AIS and VOS data have limited observation frequency, leading to gaps that when directly interpolated with geodesic paths, create invalid routes which cross land masses. We used a routing model to create a visibility graph of the oceans, creating valid potential movement paths. These movement paths are based on the assumption that mariners will prefer great circle distances when possible. Each ocean location was treated as a node in the visibility graph, and shortest distance paths were computed using the A* search algorithm[15]. The resulting graph contains 6.5M vertices (valid ship locations) and 17M edges (connections between locations). These methods are different from the ones developed previously [1], thus precluding temporal comparisons of this data layer.

Invasive species

As was done previously[1], we used the volume (measured in tonnes) of goods transported through commercial ports as a proxy measure of potential introduction of invasive species. Two main pathways account for ship-associated invasions: ballast water exchange and biofouling. Ballast water is used to achieve proper buoyancy and stability for ships contingent on the mass and distribution of cargo. Water is taken on and released proportional to the mass of goods transferred at a port. When ballast taken on at one location is transported and released at another, marine life in the water and sediment become potential non-native invaders. Biofouling introductions occur when organisms colonize the hull, other ship surfaces or equipment and are thereby transported to the host environment. Each mechanism has been associated with numerous invasions worldwide[16,17].

The number of viable propagules released is termed 'propagule pressure' and is one of the stronger determinants of invasion[18-20]. As a proxy for propagule pressure, some authors have noted a strong relationship (albeit sometimes non-linear) between cargo tonnage or related shipping measures (e.g., ship arrivals) and invasion[21-24]. Successful colonization also depends on source-destination habitat similarity, travel distance, and destination habitat degradation. In addition, ports have different regulations regarding if/how ballast water is treated, so this proxy measure may overestimate invasive species in locations where regulations are well-enforced. However, data are simply not available on a global scale to quantify these additional factors. We therefore make the simplifying assumption that risk of invasion is proportional to tonnes of goods transferred because this metric scales positively with the volume of ballast released, the surface area for fouling organisms and the number of vessel visits, all of which are established predictors of invasion.

Besides gathering new data on the current volume of goods transported through ports, we implemented a number of important improvements for how missing port volume values were approximated. We were not able to repeat these methods for previous port volume data[1], and so we used these new data for both time periods (i.e., we could not do temporal comparisons for this layer). In brief, we used total cargo volume data (in metric tonnes) reported for the 2011 calendar year for 868 ports, matched with entries in the World Port Index database (WPI; available from the National Geospatial-

Intelligence Agency). From these data, we modeled cargo volume estimates (see details below) for an additional 2,854 ports to provide a total data set of 3,722 ports with cargo throughput information.

Data Availability: Proprietary (and expensive) data exist for cargo volume for most ports in the world (e.g., from Lloyds of London). We instead relied on freely available data to produce more repeatable methods without barriers. Often, ports and port authorities report cargo throughput for the total weight of all types of goods, including bulk and containerized cargo. An alternate type of data reporting is for Twenty-foot Equivalent Units (TEUs), a measure of cargo shipped in containers. While reporting for this type of cargo is more complete, containerized shipping represents only 15-30% of trade because it excludes bulk cargoes (e.g., petroleum, ore, grain, autos, and livestock). Total cargo throughput data in both metric tonnes and TEUs are reasonably accessible for many ports, particularly in Europe, the Americas and Australia. Cargo data are less accessible or underrepresented in Asia, Africa and the Middle East with the exception of the largest ports in those regions. We collected data on cargo throughput in TEUs for 580 ports and in metric tonnes for 1,498 ports. World-wide data is available from the WPI on 3,709 ports and lists the name, location, size category and services available along with additional characteristics of each port.

Data collection: Total cargo volume data by port was collected from regional and national statistical organizations, and from published port rankings. We used online data sources (Supplementary Table 6) and statistics provided directly by individual organizations for cargo throughput measures in TEUs (N=580) and in metric tonnes (N=1,498) for 2011. Metric tonnes is a preferable measure of port throughput because it includes all classes of goods including containerized trade. Because the majority of ports with TEU data also had data in metric tonnes (432/580) and the relationship between TEUs and tonnes was moderate (R^2=0.734) overall and especially poor for smaller ports, we chose not to use the TEU data to predict tonnes for the remaining 148 ports or for modelling exercises. Only the metric tonnes data was used for the remainder of the analyses.

Preparing cargo volume data: All data were imported into a database and duplicate ports were eliminated. When metric tonnes values differed for duplicate port records, the more precise record was used (some datasets were rounded). If neither record had greater precision, the more authoritative resource was preferred in the following sequence: National > Regional > Global/Industry summary. Once all duplicates were eliminated, data were combined with the World Port Index dataset by matching port name and country to allow for gap-filling (see *Gap-filling cargo volume*, below).

To resolve inconsistencies between port identities in the port volume data versus the WPI database, we implemented several steps. First, we matched records that referred to the same port location across the datasets but did not match because of disparities in spelling, official and unofficial name use, or variation in grouping and reporting of ports. Second, for cases where large ports had a single value in the port volume datasets but were divided into subsidiary ports in the WPI data, we simply combined the WPI records. To do this, we compared all data fields among the subsidiary WPI records and chose the

most ‘conservative’ value for each field. For example, if there were a variety of channel depths among the records, the shallowest depth was entered for the newly created combined record. Subsidiary records were then deleted. Finally, in some cases, entries for ports existed in the port volume data but not the WPI data. For 23 of the largest of these ports, we were able to construct a WPI entry from various online sources. Once all possible matches and edits were made to the data sets, 868 ports were matched between WPI data and cargo volume data (estimated 9% of global ports, but representing >80% of cargo tonnage).

Preparing WPI data: Not all ports had values included for each of the port attribute fields of the WPI dataset. For missing channel (n=459), anchorage (n=361) and cargo pier (n=461) depth values in the WPI database, depth was assigned: ‘very small’ ports were given depth category 0-5 ft, ‘small’ ports were assigned category 6-10 ft, ‘medium’ given 11-15 ft and ‘large’ ports were given a value of 16-20 ft. Since all of these depths were well below the mean depth across all ports or the mean depth for any port size, these designations were considered conservative. Greater depths were associated with higher cargo volume, so conservative depth assignment leads to conservative predictions of cargo volume for modeled ports. Similarly, for binary variables in the WPI dataset, the value related to lower cargo volume was assigned where values were missing. Finally, oil terminal depth was converted to presence/absence where missing values for depth corresponded with a lack of an oil terminal. Depths in this category provide little predictive power for cargo volume since more than 90% of all ports were deeper than necessary for the largest class of oil tanker vessel. Presence/absence data on four port services (electrical, longshore, navigation equipment and electrical repair) was tallied to give a single variable quantifying the number of services offered at a port. The same was done for six categories of supplies (deck, diesel, engine, fuel oil, provisions, and water).

Gap-filling cargo volume: Using these two variables and a selection of 26 ordered and binary predictor variables from the WPI database, we constructed a set of likely linear models based on an initial analysis of predictor significance. Akaike information criterion (AIC) was calculated to choose among the models. The selected model was: Log(Tonnes +1) ~ (cargo pier depth) + (oil terminal presence) + (turning area) + (wharf presence) + (tug assistance) + (channel depth) + (garbage disposal) + (max vessel size) + (floating cranes) + (ballast treatment) + (lifts 25-49 tonnes) + (first port of entry) + (harbor size). Model fit in this case was reasonable (adjusted R^2=0.473, $F_{(43, 824)}$ = 19.1, p<<0.001), given the binary nature of most of the predictor variables. This best-fit model was then used to predict metric tons of cargo values for the unmatched records in the WPI dataset. Validation of predicted values using online searches for individual port websites and cargo data reported therein showed that of 22 selected ports, all were within an order of magnitude of the true value and most were within 10%. The modeling and prediction process resulted in 3,722 ports (~37% of global ports and 100% of the largest 100+ ports) with location and cargo volume information. Models and analysis were performed in R statistical software[25].

Pluming port volume data: To approximate the range and potential impact of invasive species introduced via ballast water, we plumed port volume data along coasts, as was

done previously[1]. In brief, the intensity of port volume was assumed to decline exponentially with (per-pixel) distance from the port source. This decay function assumes diffusive (rather than advective) spread of invasive species. We further assumed that once diffused volume data fell below a value of 5mt, no additional spread occurred. This set a maximum distance of spread (from the largest port) at roughly 1000km along a coastline. These areas were then clipped to the shallow habitats (<60 m depth), since most known invasive species transported by ballast water are intertidal or shallow subtidal.

As noted previously[1], our approach to modeling invasive species does not account for species that arrive through other transport mechanisms (such as aquaculture), and it assumes a linear relationship between invasive species occurrence and port volume, and between spatial extent of the invasion and port volume. The former relationship may instead have a threshold value or non-linear relationship, but such relationships are probably taxa-specific and are not currently known for most species. The latter relationship is also not likely to be linear, as the spread of species along coastlines is more likely a function of time since introduction and dispersal capabilities, variables which are available for only a select few marine invasive species, and the relative ocean climatology of the origin and destination of the ship. For example, models that account for the environmental niche of potential invaders perform better than models created solely on the basis of port volume [26], but such niches are not known for most invasive species, and tracking the origin and destination of ships is computationally prohibitive.

Ocean-based pollution

This data layer combines estimates of pollution coming from commercial shipping and from ports. As such, it is a combination of the shipping and port volume data layers (described above under 'shipping' and 'invasive species', respectively), with the port volume data plumed to estimate pollution from commercial ports (with exponential decline in intensity from the port). Specifically, we reduced the plume distance compared to that used for invasive species, following methods developed previously[1].

Supplementary References

1 Halpern, B. S. *et al.* A global map of human impact on marine ecosystems. *Science* **319**, 948-952 (2008).

2 Halpern, B. S., Selkoe, K. A., Micheli, F. & Kappel, C. V. Evaluating and ranking the vulnerability of global marine ecosystems to anthropogenic threats. *Conservation Biology* **21**, 1301-1315 (2007).

3 Post, E. *et al.* Ecological consequences of sea-ice decline. *Science* **341**, 519-524 (2013).

4 Moyorga, E. *et al.* Global nutrient export from watersheds 2 (NEWS 2): model development and implementation. *Environmental Modelling & Software* **25**, 837-853 (2010).

5 Halpern, B. S. *et al.* Mapping cumulative human impacts to California Current marine ecosystems *Conservation Letters* **2**, 138-148 (2009).
6 Elvidge, C. D. *et al.* A fifteen year record of global natural gas flaring derived from satellite data. *Energies* **2**, 595-622 (2009).
7 Elvidge, C. D. *et al.* National trends in satellite observed lighting: 1992-2009. *AGU Fall Meeting* **1**, 3 (2011).
8 Halpern, B. S. *et al.* Patterns and emerging trends in global ocean health. *Plos One* DOI:10.1371/journal.pone.0117863 (2015).
9 Selig, E. R. & Bruno, J. F. A Global Analysis of the Effectiveness of Marine Protected Areas in Preventing Coral Loss. *Plos One* **5**, doi:10.1371/journal.pone.0009278 (2010).
10 Casey, K. S., Selig, E. R. & Foti, G. (ed National Oceanographic Data Center) (NOAA, 2012).
11 Casey, K. S. *et al.* (ed National Oceanographic Data Center) (NOAA, 2011).
12 Reynolds, R. W. *et al.* Daily high-resolution-blended analyses for sea surface temperature. *Journal of Climate* **20**, 5473-5496, doi:10.1175/2007jcli1824.1 (2007).
13 Johnstone, J. A. & Mantua, N. J. Atmospheric controls on northeast Pacific temperature variability and change, 1900-2012. *Proceedings of the National Academy of Sciences* **111**, 14360-14365 (2014).
14 Nicholls, R. J. & Cazenave, A. Sea-Level Rise and Its Impact on Coastal Zones. *Science* **328**, 1517-1520, doi:10.1126/science.1185782 (2010).
15 Walbridge, S. *Assessing ship movements using volunteered geographic information* Master of Arts thesis, University of California, (2013).
16 Ruiz, G. M., Carlton, J. T., Grosholz, E. D. & Hines, A. H. Global invasions of marine and estuarine habitats by non-indigenous species: mechanisms, extent, and consequences. *American Zoologist* **37**, 621-632 (1997).
17 Ruiz, G. M., Fofonoff, P. W., Carlton, J. T., Wonham, M. J. & Hines, A. H. Invasion of coastal marine communities in North America: apparent patterns, processes, and biases. *Annual Review Of Ecology And Systematics* **31**, 481-531 (2000).
18 Kolar, C. S. & Lodge, D. M. Progress in invasion biology: predicting invaders. *Trends In Ecology & Evolution* **16**, 199-204 (2001).
19 Lockwood, J. L., Cassey, P. & Blackburn, T. M. The more you introduce the more you get: the role of colonization pressure and propagule pressure in invasion ecology. *Divers. Distrib.* **15**, 904-910 (2009).
20 Briski, E. & al., E. Relationship between propagule pressure and colonization pressure in invasion ecology: a test with ships' ballast. *Proceedings of the Royal Society B: Biological Sciences* **279**, 2990-2997 (2012).
21 Costello, C., Springborn, M., McAusland, C. & Solow, A. Unintended biological invasions: does risk vary by trading partner? *Journal of Environmental Economics and Management* **54**, 262-276 (2007).
22 Lo, V. B., Levings, C. D. & Chan, K. M. A. Quantifying potential propagule pressure of aquatic invasive species from the commercial shipping industry in Canada. *Marine Pollution Bulletin* **64**, 295-302 (2012).

23 Seebens, H., Gastner, M. T. & Blasius, B. The risk of marine bioinvasion caused by global shipping. *Ecology Letters* **16**, 782-790 (2013).

24 Drake, J. M. & Lodge, D. M. Global hot spots of biological invasions: evaluating options for ballast-water management. *Proceedings Of The Royal Society Of London Series B-Biological Sciences* **271**, 575-580 (2004).

25 R: a language and environment for statistical computing (R Development Core Team, Vienna, Austria, 2013).

26 Herborg, L. M., Jerde, C. L., Lodge, D. M., Ruiz, G. M. & MacIsaac, H. J. Predicting invasion risk using measures of introduction effort and environmental niche models. *Ecological Applications* **17**, 663-674 (2007).
