# Supplementary material for: Spatial and temporal changes in cumulative human impacts on the world's ocean
Source: Nat Commun. 2015 Jul 14;6:7615. doi: 10.1038/ncomms8615 (PMC4510691; doi:10.1038/ncomms8615)
Supplement: Supplementary Data 1 — Average impact scores for each stressor and for cumulative impact in 2013 for each EEZ, in decreasing order of average cumulative impact. True zero values are indicated by zeros with no trailing decimals; very small values are zeros with several zero decimal values. [file ncomms8615-s2.doc]

## *Supplementary Data 1*

Average impact scores for each stressor and for cumulative impact in 2013 for each EEZ, in decreasing order of average cumulative impact. True zero values are indicated by zeros with no trailing decimals; very small values are zeros with several zero decimal values.

| **Suppl. Data 1:**  **2013 EEZ**  **EEZ name** | **Country** | **Average cumulative impact score** | **Artisanal fishing** | **Demersal destructive fishing** | **Demersal nondestructive high bycatch fishing** | **Demersal nondestructive low bycatch fishing** | **Direct human impact** | **Inorganic pollution** | **Invasive species** | **Light pollution** | **Nutrient pollution** | **Oil rigs** | **Ocean acidification** | **Ocean-based pollution** | **Organic pollution** | **Pelagic high bycatch fishing** | **Pelagic low bycatch fishing** | **Sea level rise** | **Sea surface temperature** | **Shipping** | **UV** |
| --- | --- | --- | --- | --- | --- | --- | --- | --- | --- | --- | --- | --- | --- | --- | --- | --- | --- | --- | --- | --- | --- |
| **Singapore** | Singapore | 8.2254 | 0.0296 | 0.4915 | 0.3978 | 0.2487 | 0.5273 | 0.5466 | 1.8671 | 0.2009 | 0.3650 | 0 | 0.3407 | 0.8788 | 0.0988 | 0.0005 | 0.0044 | 1.2156 | 0.599 | 0.4778 | 0 |
| **Jordan** | Jordan | 6.5864 | 0.0696 | 0.5888 | 0.4762 | 0.2478 | 0.4039 | 0.3231 | 0.8042 | 0.205 | 0.1061 | 0 | 1.0233 | 0.592 | 0.0109 | 0.0106 | 0.0041 | 0.5078 | 1.2901 | 0.1551 | 0 |
| **Slovenia** | Slovenia | 6.1910 | 0.0090 | 0.1248 | 0.0701 | 0.1599 | 0.4133 | 0.4296 | 1.1069 | 0.1553 | 0.2401 | 0 | 0.5180 | 0.7687 | 0.0500 | 0 | 0.0043 | 0.7055 | 1.0961 | 0.5045 | 0 |
| **Bosnia and Herzegovina** | Bosnia and Herzegovina | 6.1407 | 0 | 0.0230 | 0.0249 | 0.0576 | 1.2881 | 0.4652 | 1.2695 | 0.4316 | 0.3350 | 0 | 0.2936 | 0.0239 | 0.0721 | 0 | 0 | 1.4309 | 0.4001 | 0.2322 | 0.1131 |
| **China** | China | 5.1714 | 0.0020 | 0.6008 | 0.6856 | 0.1910 | 0.0425 | 0.0429 | 0.0529 | 0.0083 | 0.0651 | 0.0004 | 0.5352 | 0.4438 | 0.0105 | 0.0103 | 0.0031 | 0.5545 | 1.1052 | 0.3890 | 0.4388 |
| **Sint-Maarten** | Netherlands | 5.1454 | 0.0268 | 0.1032 | 0.0886 | 0.0278 | 0.0674 | 0.0586 | 0.2756 | 0.04 | 0 | 0 | 1.0342 | 0.6728 | 0 | 0 | 0.0123 | 0.2363 | 1.5807 | 0.6081 | 0.3462 |
| **Gibraltar** | United Kingdom | 5.1048 | 0.0018 | 0.1565 | 0.1311 | 0.1406 | 0.0606 | 0.2074 | 0.1538 | 0.0288 | 0.0785 | 0 | 0.9756 | 0.8951 | 0.0070 | 0 | 0.0249 | 0.0873 | 1.5071 | 0.6580 | 0 |
| **Japan - Korea** | Joint Regime | 5.0725 | 0 | 0.4158 | 0.2378 | 0.1099 | 0 | 0 | 0 | 0 | 0 | 0 | 0.9951 | 0.3301 | 0 | 0 | 0.0121 | 0.0006 | 1.7935 | 0.4351 | 0.7424 |
| **United Kingdom** | United Kingdom | 5.0395 | 0.0012 | 0.3069 | 0.1742 | 0.0858 | 0.0208 | 0.0163 | 0.0625 | 0.0039 | 0.0163 | 0.0000 | 0.8469 | 0.3627 | 0.0022 | 0 | 0.0876 | 0.1655 | 1.8098 | 0.4192 | 0.6719 |
| **Turkey** | Turkey | 5.0148 | 0.0017 | 0.0902 | 0.0642 | 0.1213 | 0.0379 | 0.0469 | 0.0528 | 0.0078 | 0.0480 | 0 | 1.0722 | 0.4973 | 0.0116 | 0.0002 | 0.0160 | 0.0964 | 1.9729 | 0.3666 | 0.5436 |
| **Greece** | Greece | 4.9238 | 0.0020 | 0.1029 | 0.0665 | 0.0772 | 0.0286 | 0.0224 | 0.0310 | 0.0054 | 0.0131 | 0 | 1.0881 | 0.5454 | 0.0020 | 0 | 0.0214 | 0.0743 | 1.8336 | 0.4224 | 0.6076 |
| **Guernsey** | United Kingdom | 4.9228 | 0.0022 | 0.2760 | 0.2393 | 0.1090 | 0.0193 | 0.0052 | 0.1142 | 0.0028 | 0.0002 | 0 | 0.7174 | 0.417 | 0.0000 | 0 | 0.0302 | 0.2823 | 1.6340 | 0.5776 | 0.4972 |
| **Lebanon** | Lebanon | 4.8873 | 0.0011 | 0.0547 | 0.0937 | 0.0651 | 0.0255 | 0.0549 | 0.0300 | 0.0091 | 0.0300 | 0 | 1.1458 | 0.4579 | 0.0079 | 0.0126 | 0.0225 | 0.0294 | 2.0102 | 0.3290 | 0.5241 |
| **Morocco** | Morocco | 4.8818 | 0.0006 | 0.1251 | 0.1532 | 0.2689 | 0.0115 | 0.0211 | 0.0119 | 0.0016 | 0.0144 | 0 | 1.0656 | 0.4173 | 0.0031 | 0.0000 | 0.0120 | 0.0417 | 1.7134 | 0.3306 | 0.6935 |
| **South Korea** | South Korea | 4.8542 | 0.0009 | 0.4070 | 0.4148 | 0.1665 | 0.0347 | 0.0181 | 0.0424 | 0.0106 | 0.0218 | 0 | 0.6582 | 0.3798 | 0.0044 | 0 | 0.0041 | 0.3171 | 1.4274 | 0.3836 | 0.5745 |
| **Taiwan** | Taiwan | 4.8087 | 0.0006 | 0.2618 | 0.2094 | 0.0883 | 0.0085 | 0.0132 | 0.0132 | 0.0025 | 0.0110 | 0.0001 | 1.0613 | 0.4246 | 0.0011 | 0.0026 | 0.0155 | 0.0723 | 1.7016 | 0.3547 | 0.5702 |
| **Algeria** | Algeria | 4.8073 | 0.0013 | 0.0427 | 0.1254 | 0.0996 | 0.0166 | 0.0334 | 0.0207 | 0.0036 | 0.0114 | 0 | 1.1179 | 0.5887 | 0.0023 | 0 | 0.0101 | 0.0229 | 1.5665 | 0.4346 | 0.7142 |
| **Cyprus** | Cyprus | 4.8000 | 0.0005 | 0.0268 | 0.0222 | 0.0146 | 0.0096 | 0.0079 | 0.0102 | 0.0025 | 0.0056 | 0 | 1.1730 | 0.5011 | 0.0016 | 0.0008 | 0.0043 | 0.0222 | 1.9934 | 0.3498 | 0.6649 |
| **Syria** | Syria | 4.7674 | 0.0028 | 0.0881 | 0.1041 | 0.0530 | 0.0429 | 0.0646 | 0.0787 | 0.0121 | 0.0815 | 0 | 1.1077 | 0.4368 | 0.0212 | 0.0049 | 0.0220 | 0.0799 | 1.9313 | 0.2933 | 0.3831 |
| **Northern Saint-Martin** | France | 4.7519 | 0.0100 | 0.1451 | 0.1280 | 0.0410 | 0.0321 | 0.0084 | 0.1115 | 0.0091 | 0 | 0 | 1.0239 | 0.4255 | 0 | 0.0005 | 0.0135 | 0.1996 | 1.8244 | 0.3424 | 0.4449 |
| **Sri Lanka** | Sri Lanka | 4.7437 | 0.0005 | 0.0616 | 0.0589 | 0.0490 | 0.0075 | 0.0094 | 0.0054 | 0.0009 | 0.0104 | 0 | 1.0676 | 0.2030 | 0.0004 | 0.8517 | 0.0336 | 0.0467 | 1.5905 | 0.1195 | 0.6304 |
| **Nigeria** | Nigeria | 4.7110 | 0.0059 | 0.2017 | 0.2314 | 0.0816 | 0.0099 | 0.0186 | 0.0315 | 0.0017 | 0.0073 | 0.0967 | 1.0607 | 0.2598 | 0.0020 | 0.4404 | 0.0136 | 0.1297 | 1.5205 | 0.2361 | 0.3683 |
| **Iraq** | Iraq | 4.7103 | 0.0053 | 0.2464 | 0.1487 | 0.0489 | 0.1065 | 0.5666 | 0.7593 | 0.0277 | 0.4939 | 0.005 | 0.3891 | 0.4559 | 0.2422 | 0.0002 | 0.0004 | 0.3806 | 0.6951 | 0.1966 | 0.0282 |
| **Israel** | Israel | 4.6853 | 0.0006 | 0.0490 | 0.0845 | 0.0282 | 0.0229 | 0.0571 | 0.0856 | 0.0081 | 0.0575 | 0 | 1.0896 | 0.4231 | 0.0148 | 0.0149 | 0.0032 | 0.0966 | 1.9491 | 0.3219 | 0.3910 |
| **Ireland** | Ireland | 4.6814 | 0.0010 | 0.2206 | 0.0823 | 0.0336 | 0.0118 | 0.0059 | 0.0177 | 0.0018 | 0.0089 | 0 | 1.0160 | 0.2859 | 0.0008 | 0 | 0.0294 | 0.0456 | 1.9666 | 0.2695 | 0.6924 |
| **Democratic Republic of the Congo** | Democratic Republic of the Congo | 4.6789 | 0.0049 | 0.2092 | 0.1584 | 0.0423 | 0.1080 | 0.5366 | 0.4930 | 0.0218 | 0.2058 | 0.2025 | 0.4253 | 0.3591 | 0.0555 | 0.0517 | 0.0006 | 0.6983 | 0.6572 | 0.1474 | 0.3640 |
| **Anguilla** | United Kingdom | 4.6682 | 0.0000 | 0.0093 | 0.0073 | 0.0040 | 0.0014 | 0.0002 | 0.0009 | 0.0004 | 0 | 0 | 1.2201 | 0.3548 | 0 | 0.0008 | 0.0018 | 0.0102 | 2.1065 | 0.2164 | 0.7349 |
| **Bulgaria** | Bulgaria | 4.6524 | 0.0015 | 0.0105 | 0.0058 | 0.0325 | 0.0148 | 0.0236 | 0.0395 | 0.0034 | 0.0263 | 0 | 0.9847 | 0.4874 | 0.0049 | 0 | 0.0016 | 0.0590 | 2.0472 | 0.4504 | 0.4672 |
| **Saint Lucia** | Saint Lucia | 4.6443 | 0.0005 | 0.0313 | 0.0491 | 0.0179 | 0.0105 | 0.0134 | 0.0119 | 0.0035 | 0.0016 | 0 | 1.1733 | 0.3852 | 0.0003 | 0 | 0.0080 | 0.0152 | 2.1358 | 0.2319 | 0.5630 |
| **Puerto Rico and Virgin Islands of the United States** | United States | 4.6386 | 0.0043 | 0.0194 | 0.0121 | 0.0108 | 0.0078 | 0.0103 | 0.0200 | 0.0032 | 0.0000 | 0 | 1.2043 | 0.4034 | 0 | 0.0102 | 0.0016 | 0.0281 | 2.0639 | 0.2452 | 0.5997 |
| **Egypt** | Egypt | 4.6348 | 0.0008 | 0.0735 | 0.0876 | 0.0201 | 0.0163 | 0.0137 | 0.0427 | 0.0053 | 0.0164 | 0.0008 | 1.0760 | 0.4487 | 0.0038 | 0.0166 | 0.0012 | 0.1387 | 1.8414 | 0.3326 | 0.5096 |
| **British Virgin Islands** | United Kingdom | 4.6336 | 0.0002 | 0.0058 | 0.0054 | 0.0047 | 0.0056 | 0.0003 | 0.0085 | 0.0010 | 0 | 0 | 1.2212 | 0.4041 | 0 | 0.0069 | 0.0016 | 0.0277 | 2.023 | 0.2508 | 0.6682 |
| **Bonaire** | Netherlands | 4.6268 | 0.0011 | 0.0860 | 0.0122 | 0.0362 | 0.0116 | 0.0020 | 0.0070 | 0.0011 | 0 | 0 | 1.1855 | 0.4276 | 0 | 0 | 0.0018 | 0.0205 | 1.9976 | 0.2987 | 0.5492 |
| **Faeroe Islands** | Denmark | 4.6195 | 0.0002 | 0.2576 | 0.0802 | 0.0398 | 0.0031 | 0.0004 | 0.0070 | 0.0003 | 0 | 0 | 1.1004 | 0.2584 | 0 | 0 | 0.0537 | 0.0102 | 2.0144 | 0.1978 | 0.5972 |
| **Dominican Republic** | Dominican Republic | 4.6105 | 0.0007 | 0.0228 | 0.0162 | 0.0109 | 0.0081 | 0.0107 | 0.0124 | 0.0011 | 0.0059 | 0 | 1.2080 | 0.3793 | 0.0023 | 0.0069 | 0.0030 | 0.0182 | 2.0420 | 0.2308 | 0.6373 |
| **Dominica** | Dominica | 4.6078 | 0.0003 | 0.0210 | 0.0381 | 0.0207 | 0.0074 | 0.0033 | 0.0026 | 0.0012 | 0.0015 | 0 | 1.1799 | 0.3345 | 0.0002 | 0.0003 | 0.0083 | 0.0129 | 2.1534 | 0.2016 | 0.6253 |
| **Antigua and Barbuda** | Antigua and Barbuda | 4.5920 | 0.0005 | 0.0177 | 0.0272 | 0.0141 | 0.0029 | 0.0009 | 0.0124 | 0.0007 | 0.0000 | 0 | 1.2014 | 0.3004 | 0.0000 | 0.0003 | 0.0051 | 0.0247 | 2.1076 | 0.1837 | 0.6936 |
| **Western Sahara** | Morocco | 4.5909 | 0.0001 | 0.0629 | 0.1265 | 0.0967 | 0.0029 | 0.0005 | 0.0073 | 0.0002 | 0.0000 | 0 | 1.0140 | 0.4046 | 0.0000 | 0.0011 | 0.0068 | 0.0988 | 1.8366 | 0.2953 | 0.6413 |
| **Saint Vincent and the Grenadines** | Saint Vincent and the Grenadines | 4.5492 | 0.0007 | 0.0086 | 0.0076 | 0.0049 | 0.0077 | 0.0026 | 0.0017 | 0.0016 | 0.0007 | 0 | 1.1582 | 0.3437 | 0.0001 | 0.0001 | 0.0025 | 0.0277 | 2.1268 | 0.2171 | 0.6407 |
| **Curacao** | Netherlands | 4.5179 | 0.0014 | 0.0338 | 0.0087 | 0.0231 | 0.0068 | 0.0034 | 0.0016 | 0.0017 | 0 | 0 | 1.1888 | 0.4123 | 0 | 0 | 0.0021 | 0.0087 | 1.9790 | 0.3043 | 0.5478 |
| **Grenada** | Grenada | 4.5165 | 0.0024 | 0.0028 | 0.0033 | 0.0082 | 0.0108 | 0.0030 | 0.0059 | 0.0022 | 0.0006 | 0 | 1.1371 | 0.3658 | 0.0001 | 0.0001 | 0.0014 | 0.0258 | 2.0810 | 0.2627 | 0.6079 |
| **Gambia** | Gambia | 4.5141 | 0.0006 | 0.0549 | 0.0580 | 0.0655 | 0.0083 | 0.0172 | 0.0378 | 0.0010 | 0.0073 | 0 | 1.0364 | 0.3061 | 0.0030 | 0.0399 | 0.0045 | 0.0957 | 1.8900 | 0.2091 | 0.6799 |
| **Guadeloupe and Martinique** | France | 4.5112 | 0.0012 | 0.0197 | 0.0290 | 0.0143 | 0.0080 | 0.0048 | 0.0047 | 0.0025 | 0.0000 | 0 | 1.1972 | 0.3012 | 0.0000 | 0.0019 | 0.0065 | 0.0136 | 2.1126 | 0.1926 | 0.6054 |
| **Mauritania** | Mauritania | 4.5095 | 0.0003 | 0.0793 | 0.1209 | 0.0686 | 0.0078 | 0.0047 | 0.0061 | 0.0002 | 0.0010 | 0.0004 | 1.0239 | 0.3648 | 0.0001 | 0.0073 | 0.0040 | 0.1012 | 1.9272 | 0.2430 | 0.5591 |
| **Hala’ib Triangle** | Egypt/Sudan Disputed | 4.4997 | 0.0008 | 0.2303 | 0.1817 | 0.0561 | 0.0157 | 0.0117 | 0 | 0.0004 | 0.0013 | 0 | 1.1709 | 0.3360 | 0.0000 | 0.0030 | 0.0001 | 0.1634 | 1.7408 | 0.2769 | 0.3171 |
| **Senegal** | Senegal | 4.4877 | 0.0003 | 0.0465 | 0.0445 | 0.0540 | 0.0071 | 0.0103 | 0.0079 | 0.0007 | 0.0037 | 0 | 1.0658 | 0.2921 | 0.0004 | 0.0210 | 0.0051 | 0.0874 | 1.9567 | 0.1932 | 0.6945 |
| **Aruba** | Netherlands | 4.4854 | 0.0025 | 0.0341 | 0.0119 | 0.0308 | 0.0073 | 0.0032 | 0.0193 | 0.0019 | 0.0000 | 0 | 1.1735 | 0.3896 | 0.0000 | 0 | 0.0024 | 0.0130 | 1.9298 | 0.3022 | 0.5680 |
| **Sint-Eustasius** | Netherlands | 4.4726 | 0.0003 | 0.0292 | 0.0270 | 0.0148 | 0.0143 | 0.0019 | 0.0249 | 0.0036 | 0.0000 | 0 | 1.1504 | 0.4748 | 0.0000 | 0.0008 | 0.0074 | 0.0184 | 1.9691 | 0.3543 | 0.3860 |
| **Malaysia** | Malaysia | 4.4624 | 0.0022 | 0.4493 | 0.3396 | 0.1813 | 0.0228 | 0.0240 | 0.0384 | 0.0033 | 0.0477 | 0.0049 | 0.7384 | 0.2524 | 0.0099 | 0.0122 | 0.0200 | 0.4171 | 1.1871 | 0.2307 | 0.4883 |
| **Romania** | Romania | 4.4608 | 0.0012 | 0.0009 | 0.0017 | 0.0097 | 0.0095 | 0.0301 | 0.0430 | 0.0019 | 0.0259 | 0.001 | 0.8292 | 0.4463 | 0.0068 | 0 | 0.0000 | 0.1604 | 1.9104 | 0.5346 | 0.4563 |
| **Portugal** | Portugal | 4.4455 | 0.0006 | 0.0734 | 0.0428 | 0.0699 | 0.0060 | 0.0112 | 0.0112 | 0.0018 | 0.0058 | 0 | 1.1291 | 0.4849 | 0.0030 | 0 | 0.0097 | 0.0249 | 1.5539 | 0.3713 | 0.6487 |
| **Canary Islands** | Spain | 4.4450 | 0.0001 | 0.0208 | 0.0297 | 0.0332 | 0.0038 | 0.0027 | 0.0010 | 0.0011 | 0.0011 | 0 | 1.1285 | 0.3692 | 0.0001 | 0.0005 | 0.0098 | 0.0077 | 1.8166 | 0.2339 | 0.7889 |
| **Iceland** | Iceland | 4.4267 | 0.0005 | 0.2400 | 0.1428 | 0.1196 | 0.0036 | 0.0009 | 0.0074 | 0.0004 | 0.0011 | 0 | 0.9664 | 0.2169 | 0.0000 | 0 | 0.0349 | 0.0253 | 1.9775 | 0.1875 | 0.5078 |
| **Barbados** | Barbados | 4.4202 | 0.0000 | 0.0008 | 0.0013 | 0.0026 | 0.0008 | 0.0010 | 0.0002 | 0.0003 | 0.0004 | 0 | 1.1950 | 0.2366 | 0.0001 | 0.0044 | 0.0012 | 0.0008 | 2.1638 | 0.1403 | 0.6708 |
| **Montserrat** | United Kingdom | 4.4144 | 0.0004 | 0.0858 | 0.1001 | 0.0811 | 0.0075 | 0.0012 | 0 | 0.0008 | 0 | 0 | 1.1109 | 0.3665 | 0 | 0.0010 | 0.0119 | 0.0128 | 1.9454 | 0.3170 | 0.3777 |
| **Haiti** | Haiti | 4.4101 | 0.0032 | 0.0448 | 0.0261 | 0.0126 | 0.0220 | 0.0218 | 0.0101 | 0.0006 | 0.0011 | 0 | 1.1882 | 0.3184 | 0.0006 | 0.0035 | 0.0006 | 0.0175 | 1.9978 | 0.1972 | 0.5594 |
| **Italy** | Italy | 4.4097 | 0.0018 | 0.0756 | 0.0485 | 0.0473 | 0.0221 | 0.042 | 0.0439 | 0.0064 | 0.0264 | 0 | 1.0572 | 0.4976 | 0.0106 | 0 | 0.0094 | 0.0735 | 1.5088 | 0.4155 | 0.5317 |
| **Ghana** | Ghana | 4.4088 | 0.0001 | 0.0821 | 0.0422 | 0.0835 | 0.0046 | 0.0089 | 0.0124 | 0.0006 | 0.0044 | 0 | 1.1038 | 0.2668 | 0.0020 | 0.1751 | 0.0094 | 0.0669 | 1.7837 | 0.1735 | 0.5934 |
| **Georgia** | Georgia | 4.4018 | 0.0013 | 0.0124 | 0.0113 | 0.0455 | 0.0249 | 0.0431 | 0.0256 | 0.0049 | 0.0140 | 0 | 1.1170 | 0.3606 | 0.0037 | 0.0006 | 0.0027 | 0.0606 | 1.9845 | 0.2692 | 0.4358 |
| **Cape Verde** | Cape Verde | 4.3874 | 0.0001 | 0.0020 | 0.0008 | 0.0045 | 0.0015 | 0.0005 | 0.0006 | 0.0001 | 0.0001 | 0 | 1.1504 | 0.2500 | 0.0000 | 0.0000 | 0.0050 | 0.0042 | 2.1068 | 0.1435 | 0.7188 |
| **Tunisia** | Tunisia | 4.3871 | 0.0025 | 0.1357 | 0.1354 | 0.0715 | 0.0268 | 0.0302 | 0.0848 | 0.0049 | 0.0170 | 0.0010 | 0.8696 | 0.4246 | 0.0025 | 0 | 0.0127 | 0.2841 | 1.4545 | 0.4052 | 0.4339 |
| **Philippines** | Philippines | 4.3768 | 0.0094 | 0.1410 | 0.2430 | 0.1292 | 0.0264 | 0.0137 | 0.0126 | 0.0009 | 0.0073 | 0.0000 | 1.1254 | 0.2393 | 0.0009 | 0.0020 | 0.0493 | 0.0963 | 1.5735 | 0.1542 | 0.5609 |
| **Malta** | Malta | 4.3767 | 0.0003 | 0.0650 | 0.0664 | 0.0393 | 0.0050 | 0.0056 | 0.0055 | 0.0013 | 0.0007 | 0 | 1.1185 | 0.4087 | 0.0004 | 0 | 0.0164 | 0.0072 | 1.7065 | 0.3511 | 0.5801 |
| **Sierra Leone** | Sierra Leone | 4.3664 | 0.0002 | 0.0700 | 0.0917 | 0.0399 | 0.0080 | 0.0068 | 0.0083 | 0.0001 | 0.0043 | 0 | 1.0366 | 0.2734 | 0.0005 | 0.0193 | 0.0075 | 0.1349 | 1.8168 | 0.1647 | 0.6900 |
| **Pakistan** | Pakistan | 4.3651 | 0.0002 | 0.1404 | 0.0631 | 0.0434 | 0.0079 | 0.0140 | 0.0122 | 0.0007 | 0.0109 | 0 | 0.9693 | 0.4109 | 0.0028 | 0 | 0.0025 | 0.1030 | 1.7366 | 0.3003 | 0.5556 |
| **Poland** | Poland | 4.3641 | 0.0018 | 0.0326 | 0.2019 | 0.1678 | 0.0430 | 0.0855 | 0.1020 | 0.0092 | 0.0832 | 0 | 0.0249 | 0.4840 | 0.0224 | 0 | 0.0584 | 0.3680 | 1.4920 | 0.5788 | 0.6150 |
| **R**é**union** | France | 4.3640 | 0.0001 | 0.0008 | 0.0010 | 0.0042 | 0.0009 | 0.0012 | 0.0005 | 0.0004 | 0 | 0 | 1.1815 | 0.2424 | 0 | 0 | 0.0058 | 0.0014 | 1.9803 | 0.1408 | 0.8032 |
| **Togo** | Togo | 4.3613 | 0.0008 | 0.1518 | 0.0762 | 0.1006 | 0.0072 | 0.0136 | 0.0773 | 0.0014 | 0.003 | 0 | 1.2434 | 0.2863 | 0.0005 | 0.1798 | 0.0331 | 0.0614 | 1.5991 | 0.2208 | 0.3111 |
| **Saint Kitts and Nevis** | Saint Kitts and Nevis | 4.3601 | 0.0032 | 0.0175 | 0.0198 | 0.0116 | 0.0200 | 0.0050 | 0.0264 | 0.0054 | 0.0002 | 0 | 1.1390 | 0.4083 | 0.0002 | 0.0004 | 0.0027 | 0.0299 | 1.9883 | 0.3171 | 0.3793 |
| **Montenegro** | Montenegro | 4.3582 | 0.0011 | 0.0718 | 0.0501 | 0.0405 | 0.0387 | 0.0479 | 0.1239 | 0.0100 | 0.0247 | 0 | 0.9261 | 0.3763 | 0.0044 | 0 | 0.0055 | 0.1552 | 1.5474 | 0.3251 | 0.6275 |
| **Azores** | Portugal | 4.3568 | 0.0000 | 0.0082 | 0.0010 | 0.0027 | 0.0008 | 0.0003 | 0.0004 | 0.0002 | 0.0005 | 0 | 1.1784 | 0.3653 | 0.0001 | 0 | 0.0020 | 0.0011 | 1.8092 | 0.2414 | 0.7459 |
| **Venezuela** | Venezuela | 4.3503 | 0.0012 | 0.0440 | 0.0152 | 0.0404 | 0.0132 | 0.0140 | 0.0241 | 0.0023 | 0.0132 | 0.0000 | 1.0794 | 0.3145 | 0.0023 | 0.0002 | 0.0017 | 0.1165 | 1.9314 | 0.2185 | 0.5286 |
| **Paracel Islands** | Disputed | 4.3474 | 0 | 0.1450 | 0.1731 | 0.0683 | 0 | 0 | 0 | 0.0000 | 0 | 0 | 1.1174 | 0.3781 | 0 | 0.0010 | 0.0152 | 0.0132 | 1.5918 | 0.2699 | 0.5744 |
| **Spain** | Spain | 4.3393 | 0.0008 | 0.0725 | 0.0650 | 0.0600 | 0.013 | 0.0251 | 0.0266 | 0.0041 | 0.0143 | 0 | 1.0817 | 0.4807 | 0.0026 | 0.0000 | 0.0176 | 0.0326 | 1.4571 | 0.3893 | 0.6015 |
| **Turks and Caicos Islands** | United Kingdom | 4.3223 | 0.0008 | 0.0209 | 0.0124 | 0.0107 | 0.0036 | 0.0004 | 0.0025 | 0.0006 | 0 | 0 | 1.2096 | 0.3714 | 0 | 0.0031 | 0.0014 | 0.0353 | 1.7610 | 0.2297 | 0.6605 |
| **France** | France | 4.3159 | 0.0043 | 0.1200 | 0.0829 | 0.0635 | 0.0233 | 0.0324 | 0.0570 | 0.0060 | 0.0247 | 0 | 0.9975 | 0.4218 | 0.0102 | 0 | 0.0246 | 0.1021 | 1.3806 | 0.4593 | 0.5140 |
| **Jamaica** | Jamaica | 4.3107 | 0.0006 | 0.0228 | 0.0146 | 0.0072 | 0.0052 | 0.0058 | 0.0100 | 0.0013 | 0.0015 | 0 | 1.1845 | 0.3326 | 0.0002 | 0.0007 | 0.0003 | 0.0091 | 1.9755 | 0.2174 | 0.5242 |
| **Madeira** | Portugal | 4.3083 | 0.0000 | 0.0011 | 0.0020 | 0.0115 | 0.0006 | 0.0004 | 0.0001 | 0.0002 | 0.0002 | 0 | 1.1544 | 0.3850 | 0.0000 | 0 | 0.0033 | 0.0011 | 1.6815 | 0.2364 | 0.8308 |
| **Libya** | Libya | 4.2921 | 0.0009 | 0.0194 | 0.0443 | 0.0123 | 0.0076 | 0.0078 | 0.0159 | 0.0016 | 0.0059 | 0.0016 | 1.1084 | 0.3196 | 0.0009 | 0.0002 | 0.0025 | 0.0482 | 1.8792 | 0.2284 | 0.5906 |
| **Lithuania** | Lithuania | 4.2901 | 0.0012 | 0.0211 | 0.1708 | 0.1262 | 0.0469 | 0.0686 | 0.3480 | 0.0093 | 0.0868 | 0 | 0.0169 | 0.4710 | 0.0248 | 0 | 0.0351 | 0.5124 | 1.3443 | 0.4447 | 0.5926 |
| **Japan** | Japan | 4.2891 | 0.0008 | 0.1040 | 0.0866 | 0.0696 | 0.0081 | 0.0057 | 0.0139 | 0.0019 | 0.0041 | 0 | 1.1081 | 0.3085 | 0.0014 | 0.0081 | 0.0235 | 0.0246 | 1.6470 | 0.2069 | 0.6699 |
| **Vietnam** | Vietnam | 4.2458 | 0.0009 | 0.4119 | 0.3687 | 0.1334 | 0.0156 | 0.0161 | 0.0152 | 0.0018 | 0.0244 | 0.001 | 0.7513 | 0.2897 | 0.0040 | 0.0064 | 0.0145 | 0.3795 | 1.1645 | 0.2230 | 0.4284 |
| **Cayman Islands** | United Kingdom | 4.2440 | 0.0002 | 0.0010 | 0.0003 | 0.0007 | 0.0021 | 0.0005 | 0.0009 | 0.0007 | 0 | 0 | 1.2560 | 0.3381 | 0 | 0.0011 | 0.0001 | 0.0016 | 1.8225 | 0.2241 | 0.5947 |
| **Iran** | Iran | 4.2398 | 0.0020 | 0.1150 | 0.0858 | 0.0415 | 0.0245 | 0.0194 | 0.0535 | 0.0062 | 0.0087 | 0.01 | 0.8120 | 0.4188 | 0.0012 | 0.1968 | 0.0064 | 0.2931 | 1.3830 | 0.3960 | 0.3856 |
| **Colombia - Jamaica** | Joint Regime | 4.2375 | 0 | 0.0214 | 0.0275 | 0.0062 | 0 | 0 | 0 | 0 | 0 | 0 | 1.1624 | 0.2832 | 0 | 0 | 0.0001 | 0.0174 | 1.9546 | 0.1952 | 0.5695 |
| **Oecussi Ambeno** | East Timor | 4.2144 | 0.0008 | 0.2675 | 0.2509 | 0.1517 | 0.0315 | 0.0343 | 0 | 0.0005 | 0.0144 | 0 | 1.1275 | 0.2664 | 0.0007 | 0.0028 | 0.0077 | 0.0339 | 1.7619 | 0.1623 | 0.1187 |
| **Trinidad and Tobago** | Trinidad and Tobago | 4.2124 | 0.0005 | 0.0178 | 0.0064 | 0.0154 | 0.012 | 0.0074 | 0.0537 | 0.0032 | 0.0108 | 0.0016 | 1.0359 | 0.2826 | 0.0013 | 0.0036 | 0.0011 | 0.1120 | 1.9763 | 0.2328 | 0.4427 |
| **Nigeria - Sao Tome and Principe** | Joint Regime | 4.2089 | 0 | 0.0117 | 0.0134 | 0.0166 | 0 | 0 | 0 | 0 | 0 | 0 | 1.0434 | 0.1791 | 0 | 0.6083 | 0.0028 | 0 | 1.6807 | 0.1213 | 0.5316 |
| **Croatia** | Croatia | 4.2087 | 0.005 | 0.1496 | 0.0635 | 0.1194 | 0.0843 | 0.036 | 0.0978 | 0.0141 | 0.0363 | 0 | 0.6562 | 0.4453 | 0.0043 | 0 | 0.0049 | 0.4637 | 1.2037 | 0.4260 | 0.4298 |
| **India** | India | 4.2033 | 0.0008 | 0.1970 | 0.1342 | 0.0750 | 0.0095 | 0.0224 | 0.0182 | 0.0014 | 0.0256 | 0.0004 | 0.9706 | 0.2422 | 0.0013 | 0.0194 | 0.0158 | 0.1061 | 1.5961 | 0.1682 | 0.6032 |
| **Spratly Islands** | Disputed | 4.1927 | 0 | 0.1944 | 0.2773 | 0.1297 | 0 | 0 | 0 | 0.0000 | 0 | 0 | 1.0887 | 0.2584 | 0 | 0.0218 | 0.0550 | 0.0230 | 1.3865 | 0.1851 | 0.5729 |
| **Ivory Coast** | Ivory Coast | 4.1686 | 0.0002 | 0.0471 | 0.0218 | 0.0334 | 0.0042 | 0.013 | 0.0092 | 0.0004 | 0.0061 | 0.0005 | 1.1104 | 0.2542 | 0.0013 | 0.0333 | 0.0057 | 0.0428 | 1.8152 | 0.1463 | 0.6280 |
| **Liberia** | Liberia | 4.1633 | 0.0002 | 0.0179 | 0.0234 | 0.0114 | 0.0036 | 0.0069 | 0.0135 | 0.0001 | 0.0023 | 0 | 1.0986 | 0.2447 | 0.0002 | 0.0419 | 0.0044 | 0.0383 | 1.8709 | 0.1468 | 0.6431 |
| **Monaco** | Monaco | 4.1515 | 0.0003 | 0.0869 | 0.0823 | 0.0920 | 0.0072 | 0.0509 | 0.0046 | 0 | 0.0090 | 0 | 1.1964 | 0.5830 | 0.0015 | 0 | 0.0241 | 0.0026 | 1.4997 | 0.5110 | 0 |
| **Northern Mariana Islands and Guam** | United States | 4.1394 | 0.0003 | 0.0065 | 0.0234 | 0.0416 | 0.0005 | 0.0003 | 0.0003 | 0.0001 | 0 | 0 | 1.1749 | 0.2146 | 0 | 0 | 0.0589 | 0.0013 | 1.7994 | 0.1271 | 0.6907 |
| **Saba** | Netherlands | 4.1376 | 0.0000 | 0.0329 | 0.0260 | 0.0141 | 0.0013 | 0.0002 | 0 | 0.0006 | 0 | 0 | 1.0316 | 0.3755 | 0 | 0.0097 | 0.0032 | 0.1317 | 1.8013 | 0.3278 | 0.3817 |
| **Denmark** | Denmark | 4.1263 | 0.0021 | 0.3248 | 0.1904 | 0.0980 | 0.0617 | 0.0384 | 0.2263 | 0.0083 | 0.0719 | 0 | 0.2148 | 0.5489 | 0.0047 | 0 | 0.0283 | 0.7967 | 0.8624 | 0.3370 | 0.3282 |
| **Belgium** | Belgium | 4.1066 | 0.0004 | 0.1232 | 0.1010 | 0.0372 | 0.0493 | 0.0951 | 0.6987 | 0.0166 | 0.0491 | 0 | 0.1756 | 0.7291 | 0.0053 | 0 | 0.0029 | 0.9523 | 0.5948 | 0.2736 | 0.2158 |
| **Ukraine** | Ukraine | 4.1058 | 0.0015 | 0.0035 | 0.0034 | 0.0364 | 0.0298 | 0.0454 | 0.0868 | 0.0032 | 0.0443 | 0 | 0.7723 | 0.3964 | 0.0142 | 0.0002 | 0.0002 | 0.4041 | 1.5476 | 0.2936 | 0.4381 |
| **Cambodia** | Cambodia | 4.0888 | 0.0010 | 0.4911 | 0.3951 | 0.1606 | 0.0228 | 0.0119 | 0.0346 | 0.0004 | 0.0029 | 0 | 0.4730 | 0.2631 | 0.0002 | 0.0000 | 0.0040 | 0.7013 | 0.8918 | 0.1935 | 0.4499 |
| **Honduras** | Honduras | 4.0838 | 0.0029 | 0.0318 | 0.0190 | 0.0048 | 0.0093 | 0.0093 | 0.0085 | 0.0007 | 0.0076 | 0 | 1.0863 | 0.3060 | 0.0031 | 0.0022 | 0.0004 | 0.1467 | 1.7455 | 0.2228 | 0.4800 |
| **French Guiana** | France | 4.0821 | 0.0003 | 0.0220 | 0.0038 | 0.0028 | 0.0043 | 0.0021 | 0 | 0.0003 | 0.0009 | 0 | 1.0189 | 0.2216 | 0.0001 | 0.0047 | 0.0007 | 0.1744 | 1.8295 | 0.1541 | 0.6449 |
| **Suriname** | Suriname | 4.0768 | 0.0001 | 0.0448 | 0.0066 | 0.0072 | 0.0037 | 0.0033 | 0 | 0.0002 | 0.0044 | 0 | 1.0175 | 0.1786 | 0.0007 | 0.0075 | 0.0005 | 0.1592 | 1.9212 | 0.1354 | 0.5967 |
| **Benin** | Benin | 4.0759 | 0.0019 | 0.0719 | 0.0679 | 0.0470 | 0.0075 | 0.0225 | 0.0386 | 0.0010 | 0.0072 | 0 | 1.2404 | 0.2758 | 0.0010 | 0.0893 | 0.0301 | 0.0843 | 1.5890 | 0.2402 | 0.2686 |
| **Oman** | Oman | 4.0649 | 0.0005 | 0.0356 | 0.0169 | 0.0191 | 0.0054 | 0.0033 | 0.0031 | 0.0011 | 0.0014 | 0.0001 | 1.0163 | 0.3164 | 0.0001 | 0.0180 | 0.0070 | 0.0475 | 1.7788 | 0.2038 | 0.5964 |
| **Latvia** | Latvia | 4.0519 | 0.0005 | 0.0134 | 0.1670 | 0.1673 | 0.0273 | 0.0448 | 0.1333 | 0.0031 | 0.0487 | 0 | 0.0176 | 0.4348 | 0.0096 | 0 | 0.1074 | 0.4039 | 1.4719 | 0.4784 | 0.5380 |
| **Djibouti** | Djibouti | 4.0346 | 0.0077 | 0.1734 | 0.0667 | 0.0425 | 0.0750 | 0.0303 | 0.1087 | 0.0051 | 0.0021 | 0 | 1.0298 | 0.2208 | 0.0003 | 0.1996 | 0.0049 | 0.2229 | 1.4153 | 0.1348 | 0.3270 |
| **Guinea** | Guinea | 4.0288 | 0.0003 | 0.0730 | 0.1258 | 0.0422 | 0.0099 | 0.0053 | 0.0140 | 0.0003 | 0.0013 | 0 | 0.8487 | 0.2698 | 0.0002 | 0.0122 | 0.0043 | 0.4005 | 1.5296 | 0.1792 | 0.5172 |
| **Samoa** | Samoa | 4.0065 | 0.0012 | 0.0577 | 0.0341 | 0.0050 | 0.0051 | 0 | 0.0023 | 0.0003 | 0.0000 | 0 | 1.2178 | 0.0646 | 0.0000 | 0 | 0.0023 | 0.0121 | 1.9048 | 0.0379 | 0.6641 |
| **Brazil** | Brazil | 4.0029 | 0.0007 | 0.0740 | 0.0396 | 0.0205 | 0.0073 | 0.0081 | 0.0112 | 0.0011 | 0.0100 | 0.0006 | 1.0042 | 0.2475 | 0.0012 | 0.0443 | 0.0097 | 0.1298 | 1.6133 | 0.1666 | 0.6177 |
| **Bassas da India** | France | 3.9980 | 0 | 0.0000 | 0.0001 | 0.0020 | 0 | 0 | 0 | 0 | 0 | 0 | 1.1586 | 0.1620 | 0 | 0 | 0.0028 | 0.0015 | 1.7715 | 0.0900 | 0.8096 |
| **Guyana** | Guyana | 3.9844 | 0.0002 | 0.0740 | 0.0206 | 0.0165 | 0.0082 | 0.0065 | 0.0072 | 0.0007 | 0.0065 | 0 | 0.9208 | 0.1803 | 0.0005 | 0.0156 | 0.0007 | 0.2782 | 1.7772 | 0.1059 | 0.5711 |
| **Saint Pierre and Miquelon** | France | 3.9807 | 0.0004 | 0.2212 | 0.1592 | 0.0269 | 0.0129 | 0.0012 | 0.0308 | 0.0015 | 0.0000 | 0 | 0.6281 | 0.3089 | 0.0000 | 0 | 0.0118 | 0.1693 | 1.6738 | 0.3190 | 0.4239 |
| **Thailand** | Thailand | 3.9723 | 0.0034 | 0.2958 | 0.2057 | 0.1064 | 0.0260 | 0.0234 | 0.0364 | 0.0048 | 0.0268 | 0.0006 | 0.6886 | 0.2131 | 0.0053 | 0.0014 | 0.0041 | 0.4415 | 1.2750 | 0.1884 | 0.4346 |
| **Norway** | Norway | 3.9671 | 0.0008 | 0.1924 | 0.1890 | 0.0607 | 0.0135 | 0.0038 | 0.0189 | 0.0032 | 0.0016 | 0.0002 | 0.9427 | 0.2053 | 0.0001 | 0 | 0.0774 | 0.0582 | 1.6694 | 0.1860 | 0.4885 |
| **Niue** | New Zealand | 3.9653 | 0.0000 | 0.0006 | 0.0002 | 0.0011 | 0.0002 | 0 | 0.0000 | 0.0000 | 0 | 0 | 1.2421 | 0.0838 | 0 | 0 | 0.0016 | 0.0004 | 1.9123 | 0.0474 | 0.6757 |
| **American Samoa** | United States | 3.9472 | 0.0000 | 0.0072 | 0.0036 | 0.0100 | 0.0006 | 0 | 0.0003 | 0.0001 | 0 | 0 | 1.2090 | 0.0751 | 0 | 0 | 0.0140 | 0.0014 | 1.9044 | 0.0430 | 0.6787 |
| **Wake Island** | United States | 3.9462 | 0 | 0.0036 | 0.0133 | 0.0170 | 0 | 0 | 0.0001 | 0.0000 | 0 | 0 | 1.2610 | 0.1574 | 0 | 0 | 0.0252 | 0.0000 | 1.7973 | 0.1030 | 0.5684 |
| **Guinea Bissau** | Guinea Bissau | 3.9411 | 0.0005 | 0.0329 | 0.0379 | 0.0340 | 0.0222 | 0.0042 | 0 | 0.0001 | 0.0004 | 0 | 0.9073 | 0.2374 | 0.0000 | 0.0074 | 0.0056 | 0.3762 | 1.5889 | 0.1603 | 0.5379 |
| **R**é**publique du Congo** | RŽpublique du Congo | 3.9318 | 0.0003 | 0.0699 | 0.1262 | 0.0289 | 0.0077 | 0.0148 | 0.0328 | 0.0012 | 0.0005 | 0.0312 | 0.9469 | 0.1795 | 0.0001 | 0.1652 | 0.0022 | 0.0788 | 1.6016 | 0.1111 | 0.5398 |
| **Cuba** | Cuba | 3.9297 | 0.0014 | 0.0377 | 0.0281 | 0.0124 | 0.0189 | 0.0155 | 0.0236 | 0.0011 | 0.0117 | 0 | 1.0814 | 0.3122 | 0.0011 | 0.0078 | 0.0003 | 0.0869 | 1.6061 | 0.1983 | 0.4989 |
| **Myanmar** | Myanmar | 3.9251 | 0.0028 | 0.3325 | 0.2296 | 0.1021 | 0.0182 | 0.0121 | 0.0029 | 0.0002 | 0.0055 | 0.0000 | 0.8583 | 0.0386 | 0.0013 | 0.0022 | 0.0044 | 0.2445 | 1.4977 | 0.0241 | 0.5557 |
| **Christmas Island** | Australia | 3.9222 | 0.0001 | 0.0020 | 0.0051 | 0.0049 | 0.0002 | 0.0000 | 0.0001 | 0.0000 | 0 | 0 | 1.1248 | 0.1444 | 0 | 0.0000 | 0.0043 | 0.0009 | 1.7686 | 0.0812 | 0.7857 |
| **Albania** | Albania | 3.9211 | 0.0093 | 0.0927 | 0.0679 | 0.0349 | 0.0607 | 0.0974 | 0.2190 | 0.0057 | 0.0850 | 0 | 0.7749 | 0.4153 | 0.0127 | 0 | 0.0033 | 0.2500 | 1.2138 | 0.3125 | 0.2888 |
| **Fiji** | Fiji | 3.9198 | 0.0016 | 0.0057 | 0.0055 | 0.0052 | 0.0046 | 0.0007 | 0.0019 | 0.0001 | 0.0002 | 0 | 1.2226 | 0.0967 | 0.0000 | 0.0001 | 0.0040 | 0.0304 | 1.8808 | 0.0588 | 0.6039 |
| **Yemen** | Yemen | 3.9047 | 0.0004 | 0.0671 | 0.0225 | 0.0279 | 0.0069 | 0.0080 | 0.009 | 0.0005 | 0.0021 | 0 | 1.0284 | 0.3139 | 0.0000 | 0.0459 | 0.0136 | 0.0510 | 1.6027 | 0.2146 | 0.4947 |
| **South Africa** | South Africa | 3.9031 | 0.0002 | 0.0172 | 0.0045 | 0.0597 | 0.0040 | 0.0055 | 0.0040 | 0.0005 | 0.0038 | 0.0000 | 1.1162 | 0.2155 | 0.0006 | 0.0009 | 0.0016 | 0.0256 | 1.5746 | 0.1592 | 0.7123 |
| **Madagascar** | Madagascar | 3.9021 | 0.0010 | 0.0481 | 0.0108 | 0.0120 | 0.0082 | 0.0078 | 0.0043 | 0.0001 | 0.0007 | 0 | 1.1090 | 0.1226 | 0.0000 | 0.0006 | 0.0076 | 0.0589 | 1.7754 | 0.0711 | 0.6697 |
| **Sweden** | Sweden | 3.8806 | 0.0046 | 0.0293 | 0.1325 | 0.1020 | 0.0686 | 0.0511 | 0.2373 | 0.0149 | 0.0309 | 0 | 0.0332 | 0.4795 | 0.0017 | 0 | 0.0537 | 0.6115 | 1.1937 | 0.4171 | 0.4481 |
| **Nicaragua** | Nicaragua | 3.8575 | 0.0024 | 0.0892 | 0.0678 | 0.0196 | 0.0107 | 0.0141 | 0.0255 | 0.0003 | 0.0111 | 0 | 0.9775 | 0.2339 | 0.0054 | 0.0058 | 0.0078 | 0.3112 | 1.5174 | 0.1870 | 0.3778 |
| **Ile Europa** | France | 3.8556 | 0 | 0.0003 | 0.0004 | 0.0024 | 0 | 0 | 0 | 0 | 0 | 0 | 1.1715 | 0.0781 | 0 | 0.0033 | 0.0033 | 0.0004 | 1.7127 | 0.0431 | 0.8404 |
| **Germany** | Germany | 3.8475 | 0.0022 | 0.3023 | 0.1428 | 0.0657 | 0.076 | 0.058 | 0.2308 | 0.0133 | 0.0996 | 0 | 0.1870 | 0.5437 | 0.0210 | 0 | 0.0134 | 0.9366 | 0.6781 | 0.2547 | 0.2330 |
| **Colombia** | Colombia | 3.8447 | 0.0012 | 0.0142 | 0.0049 | 0.0078 | 0.0051 | 0.0056 | 0.0076 | 0.0004 | 0.0062 | 0 | 1.0282 | 0.2751 | 0.0030 | 0.0002 | 0.0008 | 0.0263 | 1.6458 | 0.1817 | 0.6327 |
| **Comoro Islands** | Comoro Islands | 3.8429 | 0.0005 | 0.0146 | 0.0121 | 0.0129 | 0.004 | 0.0016 | 0.0010 | 0.0001 | 0.0008 | 0 | 1.1210 | 0.1376 | 0.0001 | 0.0035 | 0.0105 | 0.0087 | 1.7556 | 0.0773 | 0.6841 |
| **Ile Tromelin** | France | 3.8369 | 0 | 0.0001 | 0.0004 | 0.0049 | 0 | 0 | 0 | 0 | 0 | 0 | 1.1316 | 0.0876 | 0 | 0 | 0.0069 | 0.0000 | 1.8508 | 0.0472 | 0.7071 |
| **Ascension** | United Kingdom | 3.8358 | 0.0000 | 0.0001 | 0.0000 | 0.0010 | 0.0001 | 0.0000 | 0.0003 | 0.0000 | 0 | 0 | 1.1090 | 0.0481 | 0 | 0 | 0.0014 | 0.0003 | 1.9314 | 0.0265 | 0.7179 |
| **Juan de Nova Island** | France | 3.8348 | 0 | 0.0011 | 0.0006 | 0.0030 | 0 | 0 | 0 | 0 | 0 | 0 | 1.1217 | 0.1590 | 0 | 0 | 0.0041 | 0.0015 | 1.7821 | 0.0948 | 0.6669 |
| **Angola** | Angola | 3.8260 | 0.0004 | 0.0955 | 0.0643 | 0.0221 | 0.0048 | 0.0098 | 0.0104 | 0.0006 | 0.0013 | 0.005 | 1.0461 | 0.1609 | 0.0002 | 0.0014 | 0.0014 | 0.0461 | 1.6670 | 0.1075 | 0.5856 |
| **Tonga** | Tonga | 3.8245 | 0.0003 | 0.0044 | 0.0047 | 0.0025 | 0.0018 | 0.0000 | 0.0012 | 0.0001 | 0.0002 | 0 | 1.2151 | 0.0817 | 0.0000 | 0 | 0.0016 | 0.0090 | 1.8544 | 0.0542 | 0.5937 |
| **Palau** | Palau | 3.8156 | 0.0006 | 0.0038 | 0.0146 | 0.0167 | 0.0009 | 0.0000 | 0.0007 | 0.0000 | 0.0000 | 0 | 1.1641 | 0.1305 | 0.0000 | 0.0005 | 0.0180 | 0.0057 | 1.6702 | 0.0728 | 0.7164 |
| **Netherlands** | Netherlands | 3.8149 | 0.0008 | 0.4001 | 0.168 | 0.0853 | 0.0451 | 0.0517 | 0.1225 | 0.0114 | 0.0546 | 0.0000 | 0.2367 | 0.5624 | 0.0166 | 0 | 0.0120 | 0.8685 | 0.6925 | 0.2473 | 0.2471 |
| **Andaman and Nicobar** | India | 3.8005 | 0.0003 | 0.0966 | 0.0886 | 0.0434 | 0.0054 | 0.0009 | 0.0004 | 0.0001 | 0.0016 | 0 | 1.0565 | 0.0842 | 0.0000 | 0.0127 | 0.0105 | 0.0379 | 1.6673 | 0.0510 | 0.6453 |
| **Wallis and Futuna** | France | 3.7936 | 0.0002 | 0.0002 | 0.0002 | 0.0003 | 0.0010 | 0 | 0.0005 | 0.0001 | 0 | 0 | 1.1969 | 0.0462 | 0 | 0 | 0.0002 | 0.0028 | 1.9238 | 0.0267 | 0.5954 |
| **Indonesia** | Indonesia | 3.7858 | 0.0036 | 0.2152 | 0.1609 | 0.1152 | 0.0169 | 0.0119 | 0.0088 | 0.0005 | 0.0113 | 0.0002 | 0.9395 | 0.1292 | 0.0012 | 0.0030 | 0.0348 | 0.2402 | 1.2759 | 0.0888 | 0.5344 |
| **Mauritius** | Mauritius | 3.7824 | 0.0001 | 0.0053 | 0.0034 | 0.0074 | 0.0004 | 0.0004 | 0.0002 | 0.0001 | 0.0004 | 0 | 1.1676 | 0.1153 | 0.0002 | 0.0002 | 0.0093 | 0.0100 | 1.7803 | 0.0695 | 0.6121 |
| **Belize** | Belize | 3.7673 | 0.0153 | 0.0493 | 0.0332 | 0.0126 | 0.0336 | 0.0155 | 0.0402 | 0.0028 | 0.0144 | 0 | 1.1119 | 0.2124 | 0.0061 | 0.0063 | 0.0002 | 0.2563 | 1.5217 | 0.1756 | 0.2745 |
| **Australia/Indonesia** | Australia/Indonesia | 3.7650 | 0 | 0.3767 | 0.1533 | 0.0979 | 0 | 0 | 0 | 0 | 0 | 0.0006 | 0.9818 | 0.0922 | 0 | 0.0068 | 0.0344 | 0.0410 | 1.2522 | 0.0930 | 0.6350 |
| **Brunei** | Brunei | 3.7482 | 0.0076 | 0.1054 | 0.0671 | 0.0433 | 0.0184 | 0.0229 | 0.0654 | 0.0052 | 0.0049 | 0.0071 | 1.0425 | 0.2928 | 0.0007 | 0.0053 | 0.0121 | 0.1784 | 1.1414 | 0.2427 | 0.4886 |
| **Norfolk Island** | Australia | 3.7458 | 0 | 0.0059 | 0.0028 | 0.0007 | 0.0001 | 0 | 0.0006 | 0.0000 | 0 | 0 | 1.2602 | 0.1871 | 0 | 0 | 0.0002 | 0.0022 | 1.4302 | 0.1080 | 0.7477 |
| **Panama** | Panama | 3.7408 | 0.0062 | 0.0600 | 0.0063 | 0.0792 | 0.0140 | 0.0088 | 0.0126 | 0.0006 | 0.0041 | 0 | 0.9927 | 0.2805 | 0.0024 | 0.0020 | 0.0044 | 0.0459 | 1.4018 | 0.1964 | 0.6302 |
| **Tokelau** | New Zealand | 3.7407 | 0 | 0.0009 | 0.0022 | 0.0039 | 0.0005 | 0 | 0 | 0 | 0 | 0 | 1.1840 | 0.0571 | 0 | 0 | 0.0046 | 0.0008 | 1.8255 | 0.0310 | 0.6302 |
| **El Salvador** | El Salvador | 3.7396 | 0.0015 | 0.0428 | 0.0482 | 0.0297 | 0.0061 | 0.0138 | 0.0147 | 0.0007 | 0.0127 | 0 | 0.9517 | 0.2285 | 0.0048 | 0.0250 | 0.0315 | 0.0412 | 1.4686 | 0.1415 | 0.6795 |
| **Japan - South Korea Conflict Zone** | Disputed | 3.7386 | 0 | 0.1372 | 0.0629 | 0.0412 | 0 | 0 | 0 | 0.0000 | 0 | 0 | 0.9291 | 0.2855 | 0 | 0 | 0.0038 | 0.0001 | 1.4643 | 0.2056 | 0.6089 |
| **Mayotte** | France | 3.7337 | 0.0004 | 0.0135 | 0.0043 | 0.0201 | 0.0060 | 0.0009 | 0.0038 | 0.0012 | 0 | 0 | 1.1045 | 0.1126 | 0 | 0.0024 | 0.0261 | 0.0315 | 1.6410 | 0.0616 | 0.7067 |
| **Maldives** | Maldives | 3.7283 | 0.0016 | 0.0165 | 0.0032 | 0.0228 | 0.0057 | 0.0000 | 0.0004 | 0.0006 | 0 | 0 | 1.1175 | 0.1228 | 0 | 0.0001 | 0.0337 | 0.0322 | 1.6228 | 0.0763 | 0.6721 |
| **Mozambique** | Mozambique | 3.7165 | 0.0010 | 0.0567 | 0.0069 | 0.0061 | 0.0114 | 0.0132 | 0.0086 | 0.0002 | 0.0057 | 0 | 1.0350 | 0.1394 | 0.0011 | 0.0019 | 0.0025 | 0.1290 | 1.6729 | 0.0846 | 0.5489 |
| **Bangladesh** | Bangladesh | 3.7143 | 0.0022 | 0.2149 | 0.2952 | 0.0536 | 0.0424 | 0.0477 | 0 | 0.0006 | 0.0600 | 0 | 0.6968 | 0.0250 | 0.0109 | 0.0014 | 0.0006 | 0.3861 | 1.3665 | 0.0235 | 0.5087 |
| **Bermuda** | United Kingdom | 3.7121 | 0.0002 | 0.0004 | 0.0005 | 0.0020 | 0.0004 | 0.0001 | 0.0021 | 0.0002 | 0 | 0 | 1.2053 | 0.3415 | 0 | 0.0004 | 0.0007 | 0.0016 | 1.1325 | 0.2000 | 0.8242 |
| **Solomon Islands** | Solomon Islands | 3.7068 | 0.0018 | 0.0045 | 0.0036 | 0.0232 | 0.0055 | 0.0006 | 0.0006 | 0.0000 | 0.0001 | 0 | 1.1436 | 0.0632 | 0.0000 | 0.0005 | 0.0327 | 0.0176 | 1.7594 | 0.0369 | 0.6154 |
| **Vanuatu** | Vanuatu | 3.7031 | 0.0016 | 0.0028 | 0.0024 | 0.0048 | 0.0059 | 0.0002 | 0.0010 | 0.0000 | 0.0001 | 0 | 1.2170 | 0.0860 | 0.0000 | 0 | 0.0068 | 0.0107 | 1.7305 | 0.0539 | 0.5830 |
| **Sao Tome and Principe** | Sao Tome and Principe | 3.6973 | 0.0002 | 0.0086 | 0.0059 | 0.0100 | 0.0026 | 0.0009 | 0.0014 | 0.0002 | 0 | 0 | 1.0157 | 0.1579 | 0 | 0.1032 | 0.0067 | 0.0089 | 1.6678 | 0.0952 | 0.6138 |
| **Cook Islands** | New Zealand | 3.6935 | 0 | 0.0003 | 0.0002 | 0.0010 | 0.0002 | 0 | 0.0000 | 0.0000 | 0.0000 | 0 | 1.1771 | 0.0670 | 0.0000 | 0 | 0.0015 | 0.0006 | 1.7336 | 0.0378 | 0.6743 |
| **Papua New Guinea** | Papua New Guinea | 3.6899 | 0.0018 | 0.0137 | 0.0125 | 0.0661 | 0.0085 | 0.0029 | 0.0025 | 0.0001 | 0.0005 | 0 | 1.0712 | 0.1345 | 0.0001 | 0.0001 | 0.0949 | 0.0702 | 1.5883 | 0.0837 | 0.5427 |
| **Sudan** | Sudan | 3.6878 | 0.0011 | 0.0647 | 0.0209 | 0.0089 | 0.0190 | 0.0096 | 0.0163 | 0.0006 | 0.0006 | 0 | 1.0752 | 0.2426 | 0.0000 | 0.0005 | 0.0002 | 0.2218 | 1.5418 | 0.1951 | 0.2820 |
| **Cocos Islands** | Australia | 3.6777 | 0 | 0.0018 | 0.0040 | 0.0041 | 0.0001 | 0.0000 | 0 | 0.0000 | 0 | 0 | 1.1229 | 0.1168 | 0 | 0 | 0.0045 | 0.0009 | 1.6055 | 0.0646 | 0.7526 |
| **Bahamas** | Bahamas | 3.6710 | 0.0013 | 0.0066 | 0.0192 | 0.0037 | 0.0075 | 0.0008 | 0.0019 | 0.0008 | 0.0003 | 0 | 1.0732 | 0.3601 | 0.0000 | 0.0014 | 0.0004 | 0.0832 | 1.2731 | 0.2323 | 0.6089 |
| **Tuvalu** | Tuvalu | 3.6591 | 0.0000 | 0.0012 | 0.0043 | 0.0083 | 0.0004 | 0 | 0.0004 | 0.0000 | 0 | 0 | 1.1484 | 0.0294 | 0 | 0 | 0.0099 | 0.0052 | 1.8013 | 0.0163 | 0.6363 |
| **Glorioso Islands** | France | 3.6481 | 0 | 0.0023 | 0.0004 | 0.0071 | 0 | 0 | 0 | 0 | 0 | 0 | 1.1356 | 0.1020 | 0 | 0 | 0.0098 | 0.0065 | 1.7014 | 0.0592 | 0.6238 |
| **British Indian Ocean Territory** | United Kingdom | 3.6427 | 0 | 0.0009 | 0.0008 | 0.0040 | 0 | 0 | 0.0003 | 0.0001 | 0 | 0 | 1.1079 | 0.1125 | 0 | 0.0000 | 0.0060 | 0.0341 | 1.6685 | 0.0627 | 0.6450 |
| **Tanzania** | Tanzania | 3.6188 | 0.0020 | 0.0385 | 0.0743 | 0.0183 | 0.0156 | 0.0151 | 0.0121 | 0.0006 | 0.0047 | 0 | 1.0282 | 0.0713 | 0.0004 | 0.0134 | 0.0024 | 0.0748 | 1.6954 | 0.0379 | 0.5211 |
| **Seychelles** | Seychelles | 3.6130 | 0.0001 | 0.0026 | 0.0012 | 0.0095 | 0.0004 | 0.0000 | 0.0006 | 0.0001 | 0.0000 | 0 | 1.0964 | 0.0799 | 0.0000 | 0.0068 | 0.0132 | 0.0172 | 1.7073 | 0.0470 | 0.6310 |
| **North Korea** | North Korea | 3.6102 | 0.0011 | 0.1660 | 0.1091 | 0.0514 | 0.0410 | 0.0340 | 0.0496 | 0.0007 | 0.0064 | 0 | 0.7130 | 0.2324 | 0.0018 | 0 | 0.0146 | 0.2773 | 1.3234 | 0.1269 | 0.4738 |
| **Equatorial Guinea** | Equatorial Guinea | 3.6003 | 0.0001 | 0.0054 | 0.0122 | 0.0079 | 0.0018 | 0.0013 | 0.0038 | 0.0002 | 0.0008 | 0.0062 | 1.0027 | 0.1674 | 0.0001 | 0.0142 | 0.0058 | 0.0160 | 1.5785 | 0.0999 | 0.6774 |
| **East Timor** | East Timor | 3.5951 | 0.0020 | 0.0912 | 0.0734 | 0.0496 | 0.0230 | 0.0216 | 0.0013 | 0.0006 | 0.0011 | 0 | 1.1059 | 0.0874 | 0.0001 | 0.0010 | 0.0036 | 0.0635 | 1.4789 | 0.0551 | 0.5464 |
| **Hawaii** | United States | 3.5918 | 0.0002 | 0.0017 | 0.0026 | 0.0059 | 0.0006 | 0.0005 | 0.0006 | 0.0001 | 0.0006 | 0 | 1.1750 | 0.2042 | 0.0001 | 0.0025 | 0.0030 | 0.0024 | 1.3385 | 0.1195 | 0.7347 |
| **New Caledonia** | France | 3.5796 | 0.0012 | 0.0005 | 0.0003 | 0.0004 | 0.0023 | 0.0001 | 0.0014 | 0.0001 | 0.0001 | 0 | 1.2312 | 0.1420 | 0.0000 | 0 | 0.0004 | 0.0313 | 1.4653 | 0.0900 | 0.6139 |
| **Australia** | Australia | 3.5743 | 0.0004 | 0.0243 | 0.0148 | 0.0123 | 0.0037 | 0.0014 | 0.0066 | 0.0003 | 0.0038 | 0.0000 | 0.9542 | 0.1767 | 0.0003 | 0.0068 | 0.0109 | 0.2016 | 1.4167 | 0.1229 | 0.6207 |
| **Estonia** | Estonia | 3.5663 | 0.0021 | 0.0083 | 0.1041 | 0.1260 | 0.0652 | 0.0405 | 0.1317 | 0.0065 | 0.0378 | 0 | 0.0095 | 0.4533 | 0.0018 | 0 | 0.0503 | 0.7789 | 0.9901 | 0.3657 | 0.4163 |
| **Cameroon** | Cameroon | 3.5602 | 0.0031 | 0.1479 | 0.4213 | 0.0941 | 0.0657 | 0.1039 | 0.1615 | 0.0038 | 0.0262 | 0.0879 | 0.4879 | 0.2285 | 0.0099 | 0.0201 | 0.0017 | 0.4740 | 0.9125 | 0.1028 | 0.2331 |
| **Clipperton Island** | France | 3.5589 | 0 | 0.0002 | 0.0023 | 0.0013 | 0 | 0 | 0 | 0 | 0 | 0 | 1.0729 | 0.1422 | 0 | 0.0068 | 0.0020 | 0.0000 | 1.4795 | 0.0784 | 0.7733 |
| **Australia - East Timor** | Joint Development | 3.5540 | 0 | 0.1685 | 0.0794 | 0.0438 | 0 | 0 | 0 | 0 | 0 | 0.0009 | 0.9525 | 0.0819 | 0 | 0.0044 | 0.0033 | 0.1578 | 1.3373 | 0.0822 | 0.6418 |
| **Tristan da Cunha** | United Kingdom | 3.5528 | 0.0000 | 0.0000 | 0.0007 | 0.0002 | 0.0000 | 0.0000 | 0.0000 | 0 | 0 | 0 | 1.0106 | 0.0359 | 0 | 0 | 0.0003 | 0.0012 | 1.7800 | 0.0211 | 0.7029 |
| **Jan Mayen** | Norway | 3.5458 | 0 | 0.0243 | 0.0016 | 0.0101 | 0 | 0 | 0 | 0 | 0 | 0 | 1.0962 | 0.0803 | 0 | 0 | 0.0022 | 0.0008 | 1.7574 | 0.0521 | 0.5213 |
| **Saudi Arabia** | Saudi Arabia | 3.5435 | 0.0020 | 0.0552 | 0.0435 | 0.0263 | 0.0245 | 0.0181 | 0.0566 | 0.0069 | 0.0084 | 0.0033 | 0.8989 | 0.2622 | 0.0004 | 0.0138 | 0.0015 | 0.3808 | 1.3092 | 0.1990 | 0.2492 |
| **Namibia** | Namibia | 3.5365 | 0.0001 | 0.0177 | 0.0070 | 0.0838 | 0.0012 | 0.0023 | 0.0016 | 0.0001 | 0.0005 | 0 | 1.0173 | 0.1637 | 0.0003 | 0 | 0.0003 | 0.0192 | 1.4758 | 0.1261 | 0.6222 |
| **United States** | United States | 3.5298 | 0.0012 | 0.0815 | 0.0319 | 0.0424 | 0.0154 | 0.0147 | 0.0303 | 0.0038 | 0.0127 | 0.0001 | 0.8843 | 0.3833 | 0.0025 | 0.0021 | 0.0023 | 0.1637 | 1.0090 | 0.2763 | 0.5779 |
| **Somalia** | Somalia | 3.5216 | 0.0002 | 0.0172 | 0.0063 | 0.0049 | 0.0052 | 0.0057 | 0.0026 | 0.0000 | 0.0007 | 0 | 1.0522 | 0.0916 | 0.0002 | 0.0044 | 0.0036 | 0.0326 | 1.6812 | 0.0543 | 0.5630 |
| **Guatemala** | Guatemala | 3.4755 | 0.0005 | 0.0405 | 0.0187 | 0.0123 | 0.0056 | 0.0172 | 0.0150 | 0.0006 | 0.0135 | 0 | 0.9507 | 0.2751 | 0.0058 | 0.0281 | 0.0105 | 0.0459 | 1.1498 | 0.1590 | 0.7309 |
| **Micronesia** | Micronesia | 3.4594 | 0.0002 | 0.0023 | 0.0082 | 0.0141 | 0.0005 | 0.0000 | 0.0003 | 0.0000 | 0 | 0 | 1.1391 | 0.1152 | 0 | 0 | 0.0184 | 0.0069 | 1.4086 | 0.0656 | 0.6801 |
| **Easter Island** | Chile | 3.4398 | 0.0000 | 0.0268 | 0.0158 | 0.0168 | 0.0001 | 0 | 0 | 0.0000 | 0.0001 | 0 | 1.0703 | 0.0214 | 0.0000 | 0 | 0.0198 | 0.0001 | 1.5429 | 0.0124 | 0.7134 |
| **Saint Helena** | United Kingdom | 3.4250 | 0.0000 | 0.0003 | 0.0001 | 0.0009 | 0.0001 | 0.0000 | 0.0000 | 0.0000 | 0 | 0 | 1.0647 | 0.0482 | 0 | 0.0001 | 0.0005 | 0.0007 | 1.4841 | 0.0255 | 0.7999 |
| **Jersey** | United Kingdom | 3.4014 | 0.0029 | 0.1898 | 0.1418 | 0.0622 | 0.0354 | 0.0217 | 0.2614 | 0.0088 | 0.0079 | 0 | 0.2124 | 0.3562 | 0.0009 | 0 | 0.0019 | 0.9035 | 0.9976 | 0.2011 | 0 |
| **Kenya** | Kenya | 3.3974 | 0.0021 | 0.0214 | 0.0104 | 0.0061 | 0.0127 | 0.0174 | 0.0028 | 0.0009 | 0.0122 | 0 | 1.0367 | 0.0562 | 0.0020 | 0.0123 | 0.0027 | 0.0460 | 1.6875 | 0.0294 | 0.4481 |
| **Nauru** | Nauru | 3.3827 | 0.0000 | 0.0019 | 0.0130 | 0.0243 | 0.0001 | 0 | 0.0001 | 0.0000 | 0 | 0 | 1.0745 | 0.0411 | 0 | 0 | 0.0219 | 0.0001 | 1.4508 | 0.0212 | 0.7338 |
| **Mexico** | Mexico | 3.3791 | 0.0006 | 0.0514 | 0.0278 | 0.0378 | 0.0048 | 0.0065 | 0.0052 | 0.0005 | 0.0059 | 0.0004 | 1.0231 | 0.2587 | 0.0023 | 0.0171 | 0.0051 | 0.0550 | 1.1138 | 0.1666 | 0.5992 |
| **Finland** | Finland | 3.3747 | 0.0041 | 0.0137 | 0.1081 | 0.0851 | 0.1110 | 0.0613 | 0.3042 | 0.0211 | 0.0605 | 0 | 0.0045 | 0.4378 | 0.0027 | 0 | 0.0267 | 1.0808 | 0.6480 | 0.2072 | 0.2183 |
| **Gabon** | Gabon | 3.3730 | 0.0036 | 0.0287 | 0.0357 | 0.0167 | 0.0065 | 0.0060 | 0.0159 | 0.0008 | 0.0010 | 0.0103 | 1.0007 | 0.1589 | 0.0002 | 0.0379 | 0.0026 | 0.0634 | 1.3738 | 0.1231 | 0.4926 |
| **United Arab Emirates** | United Arab Emirates | 3.3717 | 0.0016 | 0.1933 | 0.1814 | 0.1076 | 0.0371 | 0.0287 | 0.2162 | 0.0143 | 0.0242 | 0.0020 | 0.2994 | 0.4057 | 0.0030 | 0.0963 | 0.0035 | 0.9134 | 0.4984 | 0.1747 | 0.1762 |
| **Johnston Atoll** | United States | 3.2887 | 0 | 0.0008 | 0.0007 | 0.0032 | 0.0000 | 0 | 0.0004 | 0 | 0 | 0 | 1.1756 | 0.0892 | 0 | 0 | 0.0046 | 0.0006 | 1.3358 | 0.0496 | 0.6282 |
| **Chile/Peru** | Disputed | 3.2773 | 0.0000 | 0.0137 | 0.0317 | 0.1023 | 0.0000 | 0.0008 | 0.0004 | 0 | 0.0001 | 0 | 1.0452 | 0.1222 | 0.0000 | 0.0008 | 0.0025 | 0.0000 | 1.1243 | 0.0662 | 0.7670 |
| **Chile** | Chile | 3.2570 | 0.0006 | 0.0308 | 0.0314 | 0.0747 | 0.0045 | 0.0027 | 0.0022 | 0.0002 | 0.0021 | 0.0000 | 0.9617 | 0.0996 | 0.0007 | 0.0000 | 0.0008 | 0.0316 | 1.1927 | 0.0595 | 0.7679 |
| **Kiribati** | Kiribati | 3.2297 | 0.0007 | 0.0114 | 0.0075 | 0.0143 | 0.0014 | 0 | 0.0002 | 0.0000 | 0 | 0 | 1.0932 | 0.0297 | 0 | 0.0000 | 0.0162 | 0.0045 | 1.3458 | 0.0164 | 0.6887 |
| **Marshall Islands** | Marshall Islands | 3.2273 | 0.0005 | 0.0010 | 0.0033 | 0.0073 | 0.0014 | 0 | 0.0002 | 0.0000 | 0 | 0 | 1.1646 | 0.0646 | 0 | 0 | 0.0096 | 0.0098 | 1.3354 | 0.0385 | 0.5910 |
| **Uruguay** | Uruguay | 3.2080 | 0.0001 | 0.1570 | 0.0377 | 0.0154 | 0.0074 | 0.0069 | 0.0218 | 0.0009 | 0.0123 | 0 | 0.6673 | 0.1152 | 0.0057 | 0.0004 | 0.0013 | 0.3014 | 1.2671 | 0.0652 | 0.5267 |
| **Southern Kuriles** | Disputed | 3.2035 | 0.0003 | 0.0715 | 0.0394 | 0.0525 | 0.0045 | 0.0004 | 0.0010 | 0.0001 | 0.0007 | 0 | 0.8422 | 0.3170 | 0.0001 | 0 | 0.0094 | 0.0278 | 0.8391 | 0.2010 | 0.7988 |
| **Trindade** | Brazil | 3.1524 | 0 | 0.0104 | 0.0112 | 0.0085 | 0 | 0 | 0 | 0 | 0 | 0 | 1.1533 | 0.0518 | 0 | 0.0159 | 0.0126 | 0.0001 | 1.0378 | 0.0277 | 0.8233 |
| **French Polynesia** | France | 3.1313 | 0.0002 | 0.0006 | 0.0002 | 0.0005 | 0.0011 | 0.0000 | 0.0001 | 0.0001 | 0.0000 | 0 | 1.2183 | 0.0620 | 0.0000 | 0 | 0.0007 | 0.0040 | 1.1518 | 0.0361 | 0.6557 |
| **Qatar** | Qatar | 3.1004 | 0.0015 | 0.0952 | 0.0893 | 0.0517 | 0.0295 | 0.0151 | 0.1751 | 0.0112 | 0.0281 | 0.0519 | 0.3261 | 0.4014 | 0.0037 | 0.1222 | 0.0015 | 0.7600 | 0.618 | 0.1733 | 0.1508 |
| **Eritrea** | Eritrea | 3.0630 | 0.0039 | 0.0432 | 0.0245 | 0.0115 | 0.0338 | 0.0123 | 0.0321 | 0.0003 | 0.0001 | 0 | 0.6843 | 0.2215 | 0.0000 | 0.0000 | 0.0013 | 0.4657 | 1.0792 | 0.1764 | 0.2900 |
| **Falkland Islands** | United Kingdom | 3.0502 | 0.0001 | 0.0924 | 0.0100 | 0.0034 | 0.0013 | 0 | 0.0010 | 0.0000 | 0 | 0 | 0.8711 | 0.0360 | 0 | 0.0000 | 0.0000 | 0.0446 | 1.3247 | 0.0296 | 0.6377 |
| **Ecuador** | Ecuador | 3.0184 | 0.0006 | 0.0883 | 0.0212 | 0.0919 | 0.0087 | 0.0064 | 0.0096 | 0.0009 | 0.0071 | 0 | 0.7810 | 0.1519 | 0.0034 | 0.0023 | 0.0079 | 0.0265 | 1.1145 | 0.0987 | 0.6001 |
| **Argentina** | Argentina | 2.9874 | 0.0001 | 0.2080 | 0.0412 | 0.0218 | 0.0044 | 0.0027 | 0.0093 | 0.0005 | 0.0037 | 0.0000 | 0.7499 | 0.0601 | 0.0011 | 0.0000 | 0.0002 | 0.1572 | 0.9689 | 0.0556 | 0.7065 |
| **Howland Island and Baker Island** | United States | 2.9731 | 0 | 0.0008 | 0.0043 | 0.0143 | 0 | 0 | 0 | 0 | 0 | 0 | 1.0399 | 0.0372 | 0 | 0 | 0.0178 | 0.0001 | 1.0863 | 0.0200 | 0.7522 |
| **Alaska** | United States | 2.9369 | 0.0000 | 0.1291 | 0.0201 | 0.0171 | 0.0017 | 0.0013 | 0.0077 | 0.0003 | 0.0014 | 0.0000 | 0.6325 | 0.2014 | 0.0001 | 0 | 0.0007 | 0.0782 | 1.1135 | 0.1374 | 0.6513 |
| **Kuwait** | Kuwait | 2.9273 | 0.0027 | 0.1147 | 0.0752 | 0.0346 | 0.0508 | 0.0648 | 0.2871 | 0.0214 | 0.0329 | 0.0005 | 0.3363 | 0.3736 | 0.0043 | 0.0221 | 0.0020 | 0.5409 | 0.6203 | 0.1590 | 0.1985 |
| **Phoenix Group** | Kiribati | 2.8903 | 0.0000 | 0.0003 | 0.0011 | 0.0045 | 0.0001 | 0 | 0.0001 | 0 | 0 | 0 | 1.0769 | 0.0345 | 0 | 0.0001 | 0.0062 | 0.0003 | 1.0646 | 0.0188 | 0.6827 |
| **Bahrain** | Bahrain | 2.8858 | 0.0031 | 0.1317 | 0.1018 | 0.0496 | 0.0764 | 0.0346 | 0.2767 | 0.0380 | 0.0202 | 0.0002 | 0.2177 | 0.3478 | 0.0005 | 0.0380 | 0.0017 | 1.0425 | 0.3548 | 0.0864 | 0.0719 |
| **Galapagos Islands** | Ecuador | 2.8711 | 0.0000 | 0.0129 | 0.0093 | 0.0195 | 0.0006 | 0.0001 | 0 | 0.0000 | 0.0004 | 0 | 0.9078 | 0.1164 | 0.0001 | 0.0022 | 0.0124 | 0.0028 | 1.0892 | 0.0712 | 0.6268 |
| **South Georgia and the South Sandwich Islands** | United Kingdom | 2.8262 | 0 | 0.0021 | 0.0000 | 0.0038 | 0 | 0 | 0.0002 | 0.0000 | 0 | 0 | 0.7649 | 0.0222 | 0 | 0 | 0.0001 | 0.0042 | 1.2203 | 0.0123 | 0.7964 |
| **Amsterdam Island and Saint Paul Island** | France | 2.7868 | 0 | 0.0004 | 0.0005 | 0.0003 | 0 | 0 | 0 | 0 | 0 | 0 | 1.1346 | 0.0281 | 0 | 0 | 0.0004 | 0.0001 | 0.9164 | 0.0172 | 0.6889 |
| **Heard and McDonald Islands** | Australia | 2.7863 | 0 | 0.0223 | 0 | 0.0000 | 0 | 0 | 0 | 0 | 0 | 0 | 0.7147 | 0.0088 | 0 | 0 | 0.0022 | 0.0036 | 1.3600 | 0.0053 | 0.6696 |
| **Greenland** | Denmark | 2.7702 | 0.0005 | 0.0892 | 0.0193 | 0.0203 | 0.0015 | 0 | 0.0046 | 0.0000 | 0.0000 | 0 | 0.8223 | 0.0799 | 0.0000 | 0 | 0.0007 | 0.0428 | 1.5779 | 0.0540 | 0.4297 |
| **New Zealand** | New Zealand | 2.6644 | 0.0001 | 0.0537 | 0.0090 | 0.0035 | 0.0025 | 0.0029 | 0.0023 | 0.0002 | 0.0022 | 0 | 1.0244 | 0.0819 | 0.0002 | 0.0001 | 0.0003 | 0.0182 | 0.7621 | 0.0554 | 0.6471 |
| **Pitcairn** | United Kingdom | 2.6587 | 0 | 0.0001 | 0.0003 | 0.0008 | 0.0000 | 0 | 0 | 0 | 0 | 0 | 1.2365 | 0.0676 | 0 | 0 | 0.0012 | 0.0001 | 0.6521 | 0.0407 | 0.6593 |
| **Costa Rica** | Costa Rica | 2.6558 | 0.0011 | 0.032 | 0.0139 | 0.0081 | 0.0036 | 0.0038 | 0.0049 | 0.0004 | 0.0031 | 0 | 0.9429 | 0.1851 | 0.0019 | 0.0392 | 0.0043 | 0.0065 | 0.6341 | 0.1193 | 0.6525 |
| **Peru** | Peru | 2.6384 | 0.0002 | 0.0537 | 0.0198 | 0.1134 | 0.0038 | 0.0070 | 0.0047 | 0.0005 | 0.0038 | 0 | 0.9550 | 0.1736 | 0.0011 | 0.0034 | 0.0039 | 0.0125 | 0.4958 | 0.1067 | 0.6800 |
| **Palmyra Atoll** | United States | 2.5006 | 0 | 0.0010 | 0.0007 | 0.0021 | 0 | 0 | 0 | 0 | 0 | 0 | 1.0717 | 0.1289 | 0 | 0 | 0.0022 | 0.0008 | 0.5051 | 0.0705 | 0.7177 |
| **Canada** | Canada | 2.3959 | 0.0007 | 0.0610 | 0.0294 | 0.0131 | 0.0047 | 0.0020 | 0.0087 | 0.0008 | 0.0014 | 0.0000 | 0.5641 | 0.1114 | 0.0002 | 0 | 0.0073 | 0.1061 | 1.1448 | 0.0982 | 0.4535 |
| **Russia** | Russia | 2.3668 | 0.0003 | 0.1347 | 0.0461 | 0.0437 | 0.0036 | 0.0021 | 0.0036 | 0.0003 | 0.0011 | 0.0000 | 0.5760 | 0.0919 | 0.0002 | 0.0000 | 0.0006 | 0.0821 | 0.9835 | 0.0664 | 0.4476 |
| **Line Group** | Kiribati | 2.1291 | 0.0000 | 0.0002 | 0.0002 | 0.0014 | 0.0003 | 0 | 0.0001 | 0.0000 | 0.0000 | 0 | 1.0322 | 0.0655 | 0.0000 | 0.0001 | 0.0020 | 0.0002 | 0.2936 | 0.0355 | 0.6979 |
| **Kerguelen Islands** | France | 2.0476 | 0 | 0.0289 | 0.0002 | 0.0000 | 0 | 0 | 0 | 0.0000 | 0 | 0 | 0.7634 | 0.0179 | 0 | 0 | 0.0002 | 0.0188 | 0.5622 | 0.0126 | 0.6440 |
| **Prince Edward Islands** | South Africa | 2.0034 | 0 | 0.0015 | 0.0000 | 0.0000 | 0 | 0 | 0 | 0 | 0.0003 | 0 | 0.8806 | 0.0201 | 0.0000 | 0 | 0.0000 | 0.0005 | 0.5004 | 0.0142 | 0.5857 |
| **Bouvet Island** | Norway | 1.9900 | 0 | 0 | 0 | 0 | 0 | 0 | 0 | 0 | 0 | 0 | 0.7520 | 0.0196 | 0 | 0 | 0 | 0.0002 | 0.5778 | 0.0129 | 0.6276 |
| **Australia - Papua New Guinea** | Australia - Papua New Guinea | 1.9753 | 0.0008 | 0.0349 | 0.0243 | 0.0178 | 0.0264 | 0.0059 | 0 | 0.0001 | 0.0017 | 0 | 0.0570 | 0.099 | 0.0001 | 0.0000 | 0.0006 | 1.6078 | 0.0527 | 0.009 | 0.0376 |
| **Macquarie Island** | Australia | 1.9389 | 0.0000 | 0.0004 | 0.0000 | 0.0000 | 0.0001 | 0.0000 | 0 | 0 | 0.0000 | 0 | 0.8106 | 0.0131 | 0.0000 | 0 | 0.0000 | 0.0004 | 0.2834 | 0.0071 | 0.8238 |
| **Crozet Islands** | France | 1.9247 | 0 | 0.0024 | 0.0002 | 0.0001 | 0 | 0 | 0 | 0 | 0 | 0 | 0.8749 | 0.0149 | 0 | 0 | 0.0001 | 0.0026 | 0.3919 | 0.0099 | 0.6280 |
| **Jarvis Island** | United States | 1.8469 | 0 | 0.0003 | 0.0004 | 0.0022 | 0.0000 | 0 | 0 | 0 | 0 | 0 | 0.9592 | 0.0601 | 0 | 0 | 0.0023 | 0.0000 | 0.0244 | 0.0320 | 0.7660 |
| **Antarctica** | Antarctica | 1.5787 | 0 | 0.0025 | 0.0000 | 0.0114 | 0.0000 | 0 | 0.0001 | 0 | 0 | 0 | 0.5644 | 0.0166 | 0 | 0 | 0.0027 | 0.0271 | 0.7725 | 0.0096 | 0.4280 |
