# Supplementary material for: Spatial and temporal changes in cumulative human impacts on the world's ocean
Source: Nat Commun. 2015 Jul 14;6:7615. doi: 10.1038/ncomms8615 (PMC4510691; doi:10.1038/ncomms8615)
Supplement: Supplementary Data 2 — Average difference in impact scores for each stressor and for cumulative impact between 2013 and 2008 for each EEZ. Differences could only be calculated for the 12 (of 19) stressor layers that had data for both time. True zero values are indicated by zeros with no trailing decimals; very small values are zeros with several zero decimal values. [file ncomms8615-s3.doc]

## *Supplementary Data 2*

Average difference in impact scores for each stressor and for cumulative impact between 2013 and 2008 for each EEZ. Differences could only be calculated for the 12 (of 19) stressor layers that had data for both time. True zero values are indicated by zeros with no trailing decimals; very small values are zeros with several zero decimal values.

| **Suppl. Data 2:**  **2013 minus 2008 EEZ**  **EEZ name** | **Country** | **Average cumulative impact score** | **Demersal destructive fishing** | **Demersal nondestructive high bycatch fishing** | **Demersal nondestructive low bycatch fishing** | **Direct human impact** | **Light pollution** | **Nutrient pollution** | **Oil rigs** | **Organic pollution** | **Pelagic high bycatch fishing** | **Pelagic low bycatch fishing** | **Sea surface temperature** | **UV** |
| --- | --- | --- | --- | --- | --- | --- | --- | --- | --- | --- | --- | --- | --- | --- |
| **Juan de Nova Island** | France | 1.71970 | -0.00105 | -0.00059 | -0.00313 | 0 | 0 | 0 | 0 | 0 | 0 | -0.00435 | 1.67818 | 0.05064 |
| **Glorioso Islands** | France | 1.71014 | -0.00235 | -0.00037 | -0.00690 | 0 | 0 | 0 | 0 | 0 | 0 | -0.00993 | 1.69309 | 0.03661 |
| **Comoro Islands** | Comoro Islands | 1.70342 | -0.00432 | -0.00379 | -0.00485 | 0.00002 | -0.00008 | -0.00004 | 0 | -0.00002 | -0.00158 | -0.00466 | 1.70709 | 0.01858 |
| **Ile Tromelin** | France | 1.64815 | -0.00016 | -0.00050 | -0.00563 | 0 | 0 | 0 | 0 | 0 | 0 | -0.00812 | 1.68794 | -0.02537 |
| **Mayotte** | France | 1.60795 | -0.00512 | -0.00231 | -0.01121 | 0.00001 | 0.00011 | 0 | 0 | 0 | -0.00151 | -0.01562 | 1.63424 | 0.01226 |
| **Réunion** | France | 1.56058 | -0.00098 | -0.00114 | -0.00483 | 0.00001 | -0.00001 | 0 | 0 | 0 | 0 | -0.00683 | 1.55026 | 0.02454 |
| **Tanzania** | Tanzania | 1.55299 | -0.02147 | -0.03233 | -0.01047 | 0.00031 | -0.00006 | 0.00069 | 0 | 0.00005 | -0.01075 | -0.00193 | 1.62697 | 0.00918 |
| **East Timor** | East Timor | 1.39727 | -0.01607 | -0.01354 | -0.00781 | 0.00001 | 0.00005 | 0.00043 | 0 | 0.00003 | -0.00030 | -0.00107 | 1.39785 | 0.04786 |
| **Seychelles** | Seychelles | 1.35155 | -0.00195 | -0.00090 | -0.00694 | 0.00000 | -0.00001 | 0.00000 | 0 | 0.00000 | -0.00533 | -0.00999 | 1.35801 | 0.01878 |
| **Ascension** | United Kingdom | 1.34847 | -0.00001 | -0.00001 | -0.00017 | 0.00000 | 0.00000 | 0 | 0 | 0 | 0 | -0.00023 | 1.36646 | -0.01744 |
| **Australia - East Timor** | Joint Development | 1.32882 | -0.00368 | -0.00197 | -0.00108 | 0 | 0 | 0 | -0.00011 | 0 | -0.00015 | -0.00012 | 1.29477 | 0.04116 |
| **Sao Tome and Principe** | Sao Tome and Principe | 1.24798 | 0.00051 | 0.00037 | 0.00064 | -0.00009 | -0.00001 | -0.00011 | 0 | -0.00002 | 0.00690 | 0.00046 | 1.25530 | -0.01480 |
| **Mauritius** | Mauritius | 1.20930 | -0.00555 | -0.00373 | -0.00947 | 0.00000 | 0.00000 | -0.00008 | 0 | 0.00000 | -0.00034 | -0.01322 | 1.25952 | -0.01772 |
| **Clipperton Island** | France | 1.17905 | -0.00019 | -0.00169 | -0.00103 | 0 | 0 | 0 | 0 | 0 | -0.00536 | -0.00156 | 1.18076 | 0.00812 |
| **Australia/Indonesia** | Australia/Indonesia | 1.16252 | -0.01256 | -0.00624 | -0.00378 | 0 | 0 | 0 | 0.00010 | 0 | -0.00039 | -0.00182 | 1.18637 | 0.00084 |
| **Oecussi Ambeno** | East Timor | 1.16124 | -0.04767 | -0.04735 | -0.02527 | 0.00145 | 0.00002 | 0.00562 | 0 | 0.00031 | -0.00087 | -0.00233 | 1.69163 | -0.39593 |
| **Nigeria - Sao Tome and Principe** | Joint Regime | 1.15442 | 0.00032 | 0.00035 | 0.00042 | 0 | 0 | 0 | 0 | 0 | 0.01669 | 0.00008 | 1.15580 | -0.01924 |
| **Christmas Island** | Australia | 1.15058 | 0.00003 | 0.00003 | -0.00003 | 0.00010 | 0.00002 | 0 | 0 | 0 | 0.00000 | 0.00003 | 1.05730 | 0.09314 |
| **Madagascar** | Madagascar | 1.11435 | -0.02511 | -0.00760 | -0.00860 | 0.00014 | -0.00001 | 0.00004 | 0 | 0.00001 | -0.00049 | -0.00618 | 1.15383 | 0.01212 |
| **Saint Helena** | United Kingdom | 1.08527 | -0.00013 | -0.00003 | -0.00040 | 0.00000 | 0.00000 | 0 | 0 | 0 | -0.00003 | -0.00024 | 1.09503 | -0.00892 |
| **Mozambique** | Mozambique | 1.03612 | -0.01237 | -0.00163 | -0.00158 | 0.00002 | 0.00001 | 0.00146 | 0 | -0.00002 | -0.00056 | -0.00072 | 1.00938 | 0.04729 |
| **Galapagos Islands** | Ecuador | 1.02670 | 0.00151 | 0.00142 | 0.00257 | -0.0005 | 0.00000 | 0.00027 | 0 | 0.00004 | 0.00037 | 0.00207 | 1.02935 | -0.00971 |
| **Ghana** | Ghana | 1.01879 | -0.00672 | -0.00429 | -0.00554 | 0.00009 | 0.00001 | 0.00101 | 0 | 0.00015 | -0.02558 | -0.00135 | 1.06379 | -0.00006 |
| **Nigeria** | Nigeria | 1.01521 | 0.00311 | 0.00327 | 0.00142 | 0.00059 | -0.00026 | -0.00137 | 0.00614 | -0.00018 | 0.01191 | 0.00037 | 1.00880 | -0.01454 |
| **Papua New Guinea** | Papua New Guinea | 0.98480 | 0.00231 | 0.00208 | 0.01041 | 0.00019 | 0.00000 | -0.00017 | 0 | -0.00001 | 0.00002 | 0.01641 | 0.98247 | -0.02630 |
| **New Caledonia** | France | 0.98269 | -0.00327 | -0.00193 | -0.00354 | 0.00002 | 0.00001 | -0.00004 | 0 | -0.00001 | 0 | -0.00410 | 0.97913 | 0.01707 |
| **Indonesia** | Indonesia | 0.97312 | -0.01219 | -0.01081 | -0.00658 | 0.00030 | -0.00003 | 0.00114 | 0.00000 | 0.00017 | -0.00030 | -0.00265 | 0.99755 | 0.01104 |
| **Equatorial Guinea** | Equatorial Guinea | 0.96758 | -0.00022 | -0.00036 | -0.00033 | 0.00008 | 0.00005 | 0.00013 | 0.00099 | 0.00001 | -0.00065 | -0.00026 | 0.97478 | -0.00581 |
| **Palau** | Palau | 0.93910 | 0.00004 | 0.00013 | 0.00011 | -0.00005 | -0.00002 | 0.00000 | 0 | 0.00000 | 0.00000 | 0.00015 | 0.94983 | -0.01103 |
| **Kenya** | Kenya | 0.92766 | -0.01442 | -0.00662 | -0.00459 | 0.00016 | -0.00009 | 0.00025 | 0 | 0.00016 | -0.01051 | -0.00224 | 1.00549 | -0.03440 |
| **Philippines** | Philippines | 0.89797 | 0.00032 | -0.00009 | -0.00041 | 0.00058 | -0.00007 | -0.00100 | 0.00000 | -0.00009 | -0.00009 | -0.00056 | 0.93712 | -0.03265 |
| **Ecuador** | Ecuador | 0.88810 | 0.00135 | 0.00050 | 0.00133 | 0.00023 | -0.00003 | -0.00024 | 0 | -0.00083 | 0.00006 | 0.00021 | 0.90679 | -0.01928 |
| **Bassas da India** | France | 0.87239 | 0.00000 | -0.00010 | -0.00207 | 0 | 0 | 0 | 0 | 0 | 0 | -0.00293 | 0.79327 | 0.08422 |
| **République du Congo** | République du Congo | 0.85660 | 0.00559 | 0.00818 | 0.00248 | 0.00015 | 0.00002 | 0.00016 | -0.00061 | 0.00003 | 0.01807 | 0.00025 | 0.83612 | -0.01027 |
| **Gabon** | Gabon | 0.80600 | -0.00582 | -0.00679 | -0.00344 | 0.00036 | -0.00003 | 0.00019 | -0.00077 | 0.00005 | -0.00936 | -0.00065 | 0.80167 | 0.03357 |
| **Cameroon** | Cameroon | 0.78889 | -0.00937 | -0.02044 | -0.00577 | 0.00008 | -0.00021 | -0.00224 | 0.00202 | 0.00190 | -0.00169 | -0.00014 | 0.90552 | -0.05521 |
| **Panama** | Panama | 0.78837 | 0.01242 | 0.00173 | 0.01000 | 0.00007 | -0.00001 | 0.00027 | 0 | -0.00046 | 0.00058 | 0.00128 | 0.66696 | 0.09903 |
| **Trindade** | Brazil | 0.78569 | -0.00017 | -0.00025 | -0.00020 | 0 | 0 | 0 | 0 | 0 | -0.00038 | -0.00030 | 0.79078 | -0.00379 |
| **Angola** | Angola | 0.77834 | -0.00774 | -0.00600 | -0.00234 | 0.00050 | 0.00004 | 0.00016 | 0.00117 | 0.00004 | -0.00020 | -0.00019 | 0.81485 | -0.01984 |
| **Spratly Islands** | Disputed | 0.77263 | -0.01213 | -0.02007 | -0.00983 | 0 | 0.00000 | 0 | 0 | 0 | -0.00298 | -0.00695 | 0.81818 | 0.00640 |
| **Guatemala** | Guatemala | 0.75994 | 0.01400 | 0.00679 | 0.00485 | 0.00007 | -0.00001 | -0.00151 | 0 | 0.00043 | 0.01210 | 0.00437 | 0.72349 | -0.00206 |
| **British Indian Ocean Territory** | United Kingdom | 0.75783 | -0.00109 | -0.00096 | -0.00472 | 0 | -0.00002 | 0 | 0 | 0 | -0.00001 | -0.00731 | 0.79689 | -0.02496 |
| **Ivory Coast** | Ivory Coast | 0.75118 | -0.02235 | -0.01269 | -0.01668 | 0.00019 | 0.00005 | -0.00030 | 0.00029 | 0.00001 | -0.02689 | -0.00460 | 0.82237 | 0.01376 |
| **Myanmar** | Myanmar | 0.74908 | -0.02205 | -0.01918 | -0.00856 | 0.00045 | 0.00004 | -0.00024 | -0.00003 | 0.00041 | -0.00034 | -0.00062 | 0.80728 | -0.00392 |
| **Democratic Republic of the Congo** | Democratic Republic of the Congo | 0.74257 | 0.03898 | 0.04390 | 0.01185 | 0.00184 | -0.00205 | 0.01710 | -0.00212 | 0.00729 | 0.01738 | 0.00021 | 0.65501 | 0.01575 |
| **Sri Lanka** | Sri Lanka | 0.72819 | -0.00018 | -0.00020 | -0.00017 | 0.00001 | -0.00004 | 0.00064 | 0 | -0.00012 | -0.00416 | -0.00015 | 0.75953 | -0.02544 |
| **Maldives** | Maldives | 0.71963 | -0.00952 | -0.00227 | -0.01409 | 0.00000 | 0.00007 | 0 | 0 | 0 | -0.00010 | -0.02237 | 0.74117 | 0.02674 |
| **Sierra Leone** | Sierra Leone | 0.71510 | 0.02387 | 0.02560 | 0.01364 | -0.00004 | 0.00000 | 0.00044 | 0 | 0.00008 | 0.00876 | 0.00333 | 0.59573 | 0.04589 |
| **Brazil** | Brazil | 0.70610 | 0.00213 | 0.00135 | 0.00071 | -0.00004 | 0.00004 | 0.00054 | 0.00013 | 0.00005 | 0.00181 | 0.00039 | 0.68549 | 0.01547 |
| **Paracel Islands** | Disputed | 0.69730 | -0.01035 | -0.01499 | -0.00668 | 0 | 0.00000 | 0 | 0 | 0 | -0.00014 | -0.00194 | 0.73381 | -0.00241 |
| **Australia** | Australia | 0.68997 | -0.00069 | -0.00044 | -0.00038 | 0.00013 | 0.00001 | -0.00004 | 0.00000 | 0.00000 | -0.00023 | -0.00035 | 0.69353 | 0.00052 |
| **Benin** | Benin | 0.66937 | 0.00279 | 0.00233 | 0.00137 | 0.00011 | 0.00027 | 0.00026 | 0 | 0.00027 | 0.00444 | 0.00142 | 0.72387 | -0.06392 |
| **Norfolk Island** | Australia | 0.65373 | -0.00765 | -0.00447 | -0.00131 | -0.00001 | 0.00000 | 0 | 0 | 0 | 0 | -0.00048 | 0.69635 | -0.02867 |
| **Belize** | Belize | 0.65112 | -0.01870 | -0.01453 | -0.00553 | -0.00032 | -0.00013 | -0.00480 | 0 | 0.00130 | -0.00361 | -0.00014 | 0.62843 | 0.07512 |
| **Liberia** | Liberia | 0.64258 | -0.00345 | -0.00419 | -0.00231 | 0.00015 | 0.00003 | 0.00048 | 0 | 0.00005 | -0.01058 | -0.00111 | 0.68961 | -0.02425 |
| **Tristan da Cunha** | United Kingdom | 0.61497 | 0.00000 | -0.00002 | -0.00001 | -0.00002 | 0.00000 | 0 | 0 | 0 | 0 | -0.00001 | 0.62394 | -0.00888 |
| **Portugal** | Portugal | 0.61484 | -0.00158 | -0.00103 | -0.00146 | -0.00005 | 0.00001 | -0.00036 | 0 | -0.00033 | 0 | -0.00029 | 0.65900 | -0.03796 |
| **Colombia** | Colombia | 0.60623 | -0.00874 | -0.00348 | -0.00470 | 0.00015 | -0.00002 | 0.00078 | 0 | -0.00068 | -0.00014 | -0.00066 | 0.61246 | 0.012 |
| **India** | India | 0.59678 | -0.01094 | -0.00931 | -0.00522 | -0.00001 | 0.00006 | 0.00213 | 0.00002 | -0.00017 | -0.00232 | -0.00172 | 0.61708 | 0.00882 |
| **Costa Rica** | Costa Rica | 0.58211 | 0.00947 | 0.00471 | 0.00277 | 0.00004 | 0.00000 | -0.00063 | 0 | 0.00095 | 0.01467 | 0.00159 | 0.54703 | 0.00236 |
| **Ile Europa** | France | 0.58166 | -0.00027 | -0.00044 | -0.00246 | 0 | 0 | 0 | 0 | 0 | -0.00346 | -0.00347 | 0.50196 | 0.08982 |
| **Chile/Peru** | Disputed | 0.57700 | -0.00219 | -0.00735 | -0.01609 | 0.00000 | 0 | 0.00007 | 0 | 0.00001 | -0.00021 | -0.00066 | 0.62061 | -0.01721 |
| **El Salvador** | El Salvador | 0.57686 | -0.00158 | -0.00174 | -0.00127 | -0.00013 | -0.00008 | 0.00046 | 0 | 0.00151 | -0.00119 | -0.00144 | 0.58202 | 0.00150 |
| **Somalia** | Somalia | 0.57652 | -0.00744 | -0.00290 | -0.00231 | 0.00036 | 0.00000 | 0.00026 | 0 | 0.00006 | -0.00223 | -0.00181 | 0.59997 | -0.00579 |
| **Haiti** | Haiti | 0.55848 | 0.00096 | 0.00052 | 0.00025 | 0.00005 | 0.00008 | 0.00011 | 0 | 0.00023 | -0.00010 | 0.00001 | 0.51090 | 0.04935 |
| **Nicaragua** | Nicaragua | 0.53786 | 0.00887 | 0.00702 | 0.00215 | -0.00001 | -0.00001 | -0.00178 | 0 | 0.00132 | 0.00109 | 0.00143 | 0.53317 | -0.01289 |
| **Chile** | Chile | 0.53772 | -0.00646 | -0.01385 | -0.02016 | -0.00078 | 0.00000 | 0.00006 | 0.00000 | 0.00007 | -0.00001 | -0.00050 | 0.57168 | 0.01081 |
| **Spain** | Spain | 0.52200 | -0.01442 | -0.01301 | -0.01064 | 0.00017 | -0.00004 | -0.00120 | 0 | -0.00033 | 0.00000 | -0.00429 | 0.54130 | 0.02638 |
| **Togo** | Togo | 0.51942 | -0.00122 | -0.00076 | -0.00058 | 0.00015 | 0.00027 | -0.00168 | 0 | -0.00008 | -0.00287 | -0.00049 | 0.57977 | -0.05093 |
| **Cayman Islands** | United Kingdom | 0.51200 | -0.00185 | -0.00059 | -0.00130 | 0.00002 | -0.00001 | 0 | 0 | 0 | -0.00212 | -0.00025 | 0.43137 | 0.08691 |
| **Bouvet Island** | Norway | 0.51017 | 0 | 0 | 0 | 0 | 0 | 0 | 0 | 0 | 0 | 0 | 0.52185 | -0.01167 |
| **Malaysia** | Malaysia | 0.50970 | -0.09133 | -0.08223 | -0.04132 | 0.00072 | -0.00004 | 0.00437 | 0.00074 | 0.00169 | -0.00213 | -0.00610 | 0.73977 | -0.00997 |
| **Andaman and Nicobar** | India | 0.50775 | 0.00201 | 0.00222 | 0.00115 | -0.00012 | 0.00000 | 0.00028 | 0 | 0.00000 | 0.00055 | 0.00041 | 0.50024 | 0.00174 |
| **Yemen** | Yemen | 0.50285 | -0.00197 | -0.00082 | -0.00101 | 0.00026 | 0.00005 | 0.00081 | 0 | -0.00016 | -0.00188 | -0.00055 | 0.55581 | -0.04613 |
| **Thailand** | Thailand | 0.49637 | -0.09726 | -0.08025 | -0.04007 | 0.00063 | -0.00032 | -0.00156 | -0.00006 | -0.00068 | -0.00092 | -0.00271 | 0.72211 | 0.00257 |
| **Vietnam** | Vietnam | 0.49211 | -0.03181 | -0.03396 | -0.01344 | 0.00062 | -0.00004 | -0.00021 | -0.00003 | 0.00003 | -0.00107 | -0.00228 | 0.58186 | -0.00536 |
| **Dominican Republic** | Dominican Republic | 0.48478 | -0.01760 | -0.01290 | -0.00883 | 0.00007 | -0.00008 | 0.00090 | 0 | 0.00091 | -0.00670 | -0.00292 | 0.39926 | 0.13386 |
| **South Georgia and the South Sandwich Islands** | United Kingdom | 0.47388 | -0.00190 | 0.00000 | -0.00254 | 0 | 0.00000 | 0 | 0 | 0 | 0 | -0.00010 | 0.46143 | 0.01709 |
| **Montserrat** | United Kingdom | 0.47204 | 0.03436 | 0.03984 | 0.03317 | -0.00048 | -0.00020 | 0 | 0 | 0 | 0.00047 | 0.00573 | 0.34588 | 0.01432 |
| **Vanuatu** | Vanuatu | 0.47091 | -0.00272 | -0.00218 | -0.00393 | 0.00005 | 0.00000 | 0.00001 | 0 | 0.00000 | 0 | -0.00610 | 0.48921 | -0.00242 |
| **Taiwan** | Taiwan | 0.46573 | -0.01702 | -0.01560 | -0.00798 | 0.00000 | -0.00009 | 0.00046 | 0.00001 | 0.00003 | -0.00037 | -0.00203 | 0.50047 | 0.00892 |
| **Jamaica** | Jamaica | 0.45760 | -0.00598 | -0.00400 | -0.00232 | 0.00006 | -0.00013 | -0.00103 | 0 | -0.00011 | -0.00023 | -0.00009 | 0.43495 | 0.03705 |
| **Micronesia** | Micronesia | 0.43730 | -0.00015 | -0.00047 | -0.00067 | 0.00002 | 0.00000 | 0 | 0 | 0 | 0 | -0.00105 | 0.43115 | 0.00849 |
| **Amsterdam Island and Saint Paul Island** | France | 0.43712 | -0.00030 | -0.00035 | -0.00021 | 0 | 0.00000 | 0 | 0 | 0 | 0 | -0.00033 | 0.43264 | 0.00568 |
| **Northern Mariana Islands and Guam** | United States | 0.43487 | 0.00305 | 0.00919 | 0.01474 | -0.00002 | -0.00002 | 0 | 0 | 0 | 0 | 0.02293 | 0.37898 | 0.00611 |
| **French Polynesia** | France | 0.43192 | -0.00092 | -0.00029 | -0.00076 | 0.00002 | 0.00000 | 0.00000 | 0 | 0.00000 | 0 | -0.00113 | 0.44575 | -0.01071 |
| **Mexico** | Mexico | 0.41936 | -0.00159 | -0.00099 | -0.00110 | 0.00023 | 0.00000 | -0.00027 | 0.00000 | 0.00060 | -0.00076 | -0.00023 | 0.41843 | 0.00608 |
| **Malta** | Malta | 0.40877 | 0.01367 | 0.01413 | 0.00818 | 0.00004 | 0.00000 | -0.00027 | 0 | 0.00021 | 0 | 0.00420 | 0.37786 | -0.00901 |
| **Western Sahara** | Morocco | 0.38570 | 0.00511 | 0.00805 | 0.00626 | 0.00000 | 0.00001 | 0.00000 | 0 | 0.00000 | 0.00012 | 0.00071 | 0.32640 | 0.03985 |
| **Pakistan** | Pakistan | 0.38502 | -0.03026 | -0.01838 | -0.01143 | 0.00022 | 0.00001 | 0.00161 | 0 | 0.00035 | 0 | -0.00108 | 0.43213 | 0.01400 |
| **Cuba** | Cuba | 0.38331 | -0.01971 | -0.01555 | -0.00736 | -0.00036 | -0.00005 | 0.00168 | 0 | 0.00027 | -0.00542 | -0.00020 | 0.40485 | 0.02862 |
| **Honduras** | Honduras | 0.38278 | -0.00988 | -0.00606 | -0.00168 | 0.00013 | -0.00001 | -0.00092 | 0 | 0.00034 | -0.00083 | -0.00014 | 0.37793 | 0.02454 |
| **Puerto Rico and Virgin Islands of the United States** | United States | 0.37889 | -0.00423 | -0.00281 | -0.00254 | -0.00016 | -0.00026 | 0.00000 | 0 | 0 | -0.00279 | -0.00043 | 0.34476 | 0.04830 |
| **Morocco** | Morocco | 0.36072 | -0.00447 | -0.00546 | -0.00625 | -0.00006 | -0.00002 | -0.00119 | 0 | -0.00169 | 0.00000 | -0.00063 | 0.38756 | -0.0062 |
| **Antigua and Barbuda** | Antigua and Barbuda | 0.36003 | 0.00761 | 0.01140 | 0.00605 | 0.00007 | -0.00003 | -0.00002 | 0 | 0.00001 | 0.00014 | 0.00240 | 0.23894 | 0.09362 |
| **Brunei** | Brunei | 0.35250 | -0.06028 | -0.04357 | -0.02783 | -0.00019 | -0.00007 | 0.00050 | 0.00217 | 0.00012 | -0.00441 | -0.00993 | 0.50426 | -0.00669 |
| **Cocos Islands** | Australia | 0.35195 | 0.00020 | 0.00043 | 0.00043 | 0.00000 | 0.00000 | 0 | 0 | 0 | 0 | 0.00049 | 0.34056 | 0.00984 |
| **French Guiana** | France | 0.33982 | 0.00088 | 0.00017 | 0.00013 | -0.00004 | -0.00005 | 0.00016 | 0 | 0.00002 | 0.00022 | 0.00003 | 0.32926 | 0.00962 |
| **Peru** | Peru | 0.33139 | -0.01402 | -0.00721 | -0.01763 | 0.00006 | 0.00005 | 0.00045 | 0 | 0.00019 | -0.00139 | -0.00158 | 0.39732 | -0.02448 |
| **Bulgaria** | Bulgaria | 0.32664 | -0.01642 | -0.00897 | -0.03417 | -0.00005 | 0.00011 | 0.00222 | 0 | 0.00094 | 0 | -0.00288 | 0.29361 | 0.09339 |
| **Colombia - Jamaica** | Joint Regime | 0.32470 | -0.01557 | -0.01864 | -0.00493 | 0 | 0 | 0 | 0 | 0 | 0 | -0.00010 | 0.37163 | -0.00769 |
| **Cook Islands** | New Zealand | 0.32010 | -0.00021 | -0.00017 | -0.00074 | -0.00005 | 0.00000 | 0.00000 | 0 | 0.00000 | 0 | -0.00113 | 0.32537 | -0.00296 |
| **Guinea** | Guinea | 0.31873 | -0.00747 | -0.01102 | -0.00434 | -0.00009 | -0.00001 | 0.00009 | 0 | 0.00014 | -0.00164 | -0.00057 | 0.34162 | 0.00309 |
| **Saint Kitts and Nevis** | Saint Kitts and Nevis | 0.30834 | -0.02027 | -0.02138 | -0.01386 | 0.00080 | -0.00017 | -0.00070 | 0 | 0.00001 | -0.00055 | -0.00352 | 0.34383 | 0.02660 |
| **Uruguay** | Uruguay | 0.30525 | 0.02253 | 0.00599 | 0.00281 | -0.00002 | 0.00006 | 0.00048 | 0 | 0.00083 | 0.00007 | 0.00027 | 0.26572 | 0.00686 |
| **Grenada** | Grenada | 0.29614 | -0.00444 | -0.00511 | -0.00917 | 0.00027 | -0.00001 | -0.00015 | 0 | -0.00006 | -0.00010 | -0.00229 | 0.30348 | 0.01437 |
| **Anguilla** | United Kingdom | 0.29334 | -0.00429 | -0.00398 | -0.00240 | 0.00002 | 0.00001 | 0 | 0 | 0 | -0.00053 | -0.00125 | 0.18708 | 0.11874 |
| **Suriname** | Suriname | 0.29109 | -0.01330 | -0.00227 | -0.00251 | 0.00000 | -0.00001 | 0.00135 | 0 | 0.00008 | -0.00281 | -0.00018 | 0.31016 | 0.00232 |
| **Lithuania** | Lithuania | 0.28495 | -0.00129 | -0.00799 | -0.00511 | 0.00035 | -0.00028 | -0.00143 | 0 | 0.00452 | 0 | -0.00206 | 0.30731 | -0.00206 |
| **Singapore** | Singapore | 0.28274 | -0.06855 | -0.06733 | -0.03523 | 0.00954 | 0.00280 | -0.01511 | 0 | 0.00631 | -0.00014 | -0.00128 | 0.50635 | 0 |
| **Barbados** | Barbados | 0.28058 | -0.00059 | -0.00091 | -0.00174 | 0.00000 | 0.00000 | -0.00005 | 0 | 0.00000 | -0.00315 | -0.00087 | 0.28250 | 0.00541 |
| **Sint-Eustasius** | Netherlands | 0.27570 | -0.02837 | -0.02491 | -0.01600 | -0.00039 | -0.00037 | -0.00003 | 0 | 0.00002 | -0.00101 | -0.00906 | 0.32628 | 0.03031 |
| **Tonga** | Tonga | 0.27423 | -0.00231 | -0.00252 | -0.00134 | -0.00006 | -0.00001 | 0.00001 | 0 | 0.00000 | 0 | -0.00092 | 0.30190 | -0.02049 |
| **Saint Vincent and the Grenadines** | Saint Vincent and the Grenadines | 0.26626 | -0.00399 | -0.00359 | -0.00241 | -0.00015 | -0.00012 | 0.00005 | 0 | -0.00002 | -0.00005 | -0.00124 | 0.28162 | -0.00332 |
| **Fiji** | Fiji | 0.26622 | -0.00766 | -0.00835 | -0.00705 | -0.00007 | -0.00002 | -0.00007 | 0 | 0.00000 | -0.00019 | -0.00612 | 0.31669 | -0.02045 |
| **Oman** | Oman | 0.25859 | -0.00211 | -0.00112 | -0.00126 | 0.00031 | 0.00010 | -0.00040 | -0.00001 | -0.00010 | -0.00132 | -0.00052 | 0.27823 | -0.01228 |
| **France** | France | 0.25562 | -0.02024 | -0.01530 | -0.01051 | 0.00004 | -0.00012 | -0.00156 | 0 | -0.00009 | 0 | -0.00539 | 0.35296 | -0.04207 |
| **British Virgin Islands** | United Kingdom | 0.25361 | -0.00075 | -0.00068 | -0.00062 | 0.00021 | -0.00010 | 0 | 0 | 0 | -0.00098 | -0.00023 | 0.18880 | 0.06810 |
| **Venezuela** | Venezuela | 0.25340 | -0.03427 | -0.01534 | -0.02885 | 0.00023 | -0.00009 | 0.00167 | 0.00000 | 0.00012 | -0.00028 | -0.00206 | 0.32826 | 0.00583 |
| **Curacao** | Netherlands | 0.25265 | -0.02842 | -0.01059 | -0.01635 | -0.00002 | -0.00007 | 0 | 0 | 0 | 0 | -0.00323 | 0.28760 | 0.02455 |
| **Samoa** | Samoa | 0.24307 | 0.01792 | 0.01174 | 0.00217 | 0.00002 | -0.00010 | 0.00000 | 0 | 0.00000 | 0 | 0.00107 | 0.17620 | 0.03430 |
| **Guadeloupe and Martinique** | France | 0.24034 | -0.01147 | -0.01709 | -0.01006 | 0.00029 | -0.00013 | 0.00000 | 0 | 0.00000 | -0.00164 | -0.00535 | 0.26650 | 0.01980 |
| **Saba** | Netherlands | 0.24010 | -0.03625 | -0.02878 | -0.01604 | -0.00003 | -0.00007 | 0 | 0 | 0 | -0.01215 | -0.00395 | 0.29374 | 0.04363 |
| **Solomon Islands** | Solomon Islands | 0.23094 | -0.00755 | -0.00605 | -0.03504 | 0.00008 | 0.00000 | 0.00002 | 0 | 0.00000 | -0.00090 | -0.05542 | 0.34383 | -0.00759 |
| **Trinidad and Tobago** | Trinidad and Tobago | 0.23036 | -0.01392 | -0.00519 | -0.01021 | 0.00030 | 0.00032 | 0.00487 | 0.00016 | 0.00074 | -0.00329 | -0.00096 | 0.29327 | -0.03507 |
| **Algeria** | Algeria | 0.22864 | -0.01407 | -0.03479 | -0.02501 | 0.00028 | 0.00015 | -0.00196 | 0 | -0.00022 | 0 | -0.00412 | 0.30457 | 0.00471 |
| **Aruba** | Netherlands | 0.22755 | -0.03165 | -0.01479 | -0.02285 | 0.00029 | -0.00011 | 0.00000 | 0 | 0.00000 | 0 | -0.00362 | 0.28730 | 0.01355 |
| **Saint Lucia** | Saint Lucia | 0.22429 | -0.01945 | -0.02657 | -0.01139 | -0.00001 | 0.00000 | -0.00027 | 0 | -0.00010 | 0 | -0.00567 | 0.29398 | -0.00511 |
| **Cape Verde** | Cape Verde | 0.22208 | -0.00122 | -0.00049 | -0.00283 | 0.00001 | -0.00001 | 0.00001 | 0 | 0.00000 | 0.00000 | -0.00322 | 0.21521 | 0.01476 |
| **Lebanon** | Lebanon | 0.21562 | 0.00563 | 0.00937 | 0.00622 | 0.00015 | -0.00027 | 0.00052 | 0 | 0.00084 | 0.00156 | 0.00270 | 0.08837 | 0.10124 |
| **Dominica** | Dominica | 0.21217 | -0.01404 | -0.02275 | -0.01593 | 0.00008 | -0.00020 | -0.00055 | 0 | -0.00004 | -0.00033 | -0.00760 | 0.27712 | -0.00299 |
| **Guinea Bissau** | Guinea Bissau | 0.21085 | -0.01271 | -0.01506 | -0.01089 | 0.00029 | 0.00000 | 0.00002 | 0 | 0.00003 | -0.00378 | -0.00268 | 0.25268 | 0.00489 |
| **Niue** | New Zealand | 0.19431 | -0.00055 | -0.00022 | -0.00095 | -0.00002 | 0.00000 | 0 | 0 | 0 | 0 | -0.00144 | 0.20192 | -0.00441 |
| **Georgia** | Georgia | 0.18642 | -0.01491 | -0.01312 | -0.03435 | 0.00010 | 0.00091 | -0.00074 | 0 | 0.00006 | -0.00088 | -0.00367 | 0.19621 | 0.05836 |
| **Guyana** | Guyana | 0.18529 | -0.03504 | -0.01188 | -0.00997 | 0.00011 | -0.00005 | 0.00051 | 0 | -0.00001 | -0.01131 | -0.00054 | 0.25549 | -0.00112 |
| **Mauritania** | Mauritania | 0.17889 | -0.00522 | -0.00619 | -0.00333 | 0.00016 | 0.00000 | -0.00014 | 0.00036 | 0.00000 | -0.00073 | -0.00040 | 0.17938 | 0.01597 |
| **Jordan** | Jordan | 0.17274 | 0.04423 | 0.04176 | 0.02050 | 0.00175 | 0.00619 | 0.00703 | 0 | 0.00279 | 0.00150 | 0.00058 | 0.05660 | 0 |
| **American Samoa** | United States | 0.17213 | 0.00575 | 0.00275 | 0.00829 | 0.00001 | -0.00001 | 0 | 0 | 0 | 0 | 0.01162 | 0.12728 | 0.01646 |
| **Monaco** | Monaco | 0.17118 | 0.04495 | 0.03959 | 0.03494 | 0.00002 | 0 | -0.00102 | 0 | 0.00005 | 0 | 0.01364 | 0.14223 | -0.10322 |
| **Wake Island** | United States | 0.16831 | -0.00127 | -0.00396 | -0.00454 | 0 | 0.00000 | 0 | 0 | 0 | 0 | -0.00786 | 0.13631 | 0.04963 |
| **Heard and McDonald Islands** | Australia | 0.16381 | -0.00953 | 0 | 0.00000 | 0 | 0.00000 | 0 | 0 | 0 | 0 | -0.00099 | 0.15476 | 0.01960 |
| **Bonaire** | Netherlands | 0.15971 | -0.06762 | -0.01487 | -0.02629 | -0.00028 | -0.00010 | 0 | 0 | 0 | 0 | -0.00273 | 0.27971 | -0.00652 |
| **Madeira** | Portugal | 0.15443 | -0.00015 | -0.00027 | -0.00117 | 0.00000 | 0.00000 | -0.00010 | 0 | -0.00003 | 0 | -0.00047 | 0.13557 | 0.02108 |
| **Ukraine** | Ukraine | 0.15337 | -0.00557 | -0.00545 | -0.04166 | -0.00097 | -0.00015 | 0.00847 | 0 | 0.00420 | -0.00039 | -0.00035 | 0.20876 | -0.01146 |
| **Japan** | Japan | 0.15299 | -0.00945 | -0.00954 | -0.00764 | -0.00020 | -0.00012 | -0.00029 | 0 | -0.00010 | -0.00100 | -0.00301 | 0.18266 | 0.00207 |
| **Poland** | Poland | 0.15216 | -0.01935 | -0.08151 | -0.05254 | -0.00012 | -0.00062 | 0.00123 | 0 | 0.00407 | 0 | -0.03237 | 0.33517 | -0.00035 |
| **Southern Kuriles** | Disputed | 0.15003 | -0.00943 | -0.00692 | -0.00765 | -0.00001 | 0.00001 | 0.00022 | 0 | 0.00003 | 0 | -0.00187 | 0.12015 | 0.05586 |
| **Japan - Korea** | Joint Regime | 0.14451 | -0.02528 | -0.01788 | -0.00826 | 0 | 0 | 0 | 0 | 0 | 0 | -0.00146 | 0.09696 | 0.10042 |
| **Canary Islands** | Spain | 0.14391 | -0.00065 | -0.00087 | -0.00086 | 0.00009 | -0.00003 | -0.00065 | 0 | -0.00004 | -0.00002 | -0.00039 | 0.12348 | 0.02410 |
| **Djibouti** | Djibouti | 0.13546 | 0.00386 | 0.00174 | 0.00110 | 0.00697 | 0.00106 | 0.00053 | 0 | 0.00000 | 0.00613 | 0.00015 | 0.02696 | 0.08756 |
| **South Africa** | South Africa | 0.13220 | -0.00546 | -0.00144 | -0.01160 | 0.00007 | -0.00001 | 0.00005 | -0.00003 | 0.00005 | -0.00031 | -0.00057 | 0.10360 | 0.04805 |
| **South Korea** | South Korea | 0.13060 | -0.01969 | -0.01922 | -0.00868 | -0.00031 | -0.00022 | -0.00254 | 0 | -0.00006 | 0 | -0.00039 | 0.13577 | 0.04706 |
| **Wallis and Futuna** | France | 0.12602 | -0.00202 | -0.00179 | -0.00167 | 0.00000 | -0.00001 | 0 | 0 | 0 | 0 | -0.00182 | 0.16061 | -0.02723 |
| **Northern Saint-Martin** | France | 0.12485 | -0.07886 | -0.07397 | -0.02672 | 0.00049 | -0.00015 | 0 | 0 | 0 | -0.00039 | -0.01072 | 0.29381 | 0.02265 |
| **Tokelau** | New Zealand | 0.10527 | -0.00069 | -0.00140 | -0.00201 | 0.00002 | 0.00000 | 0 | 0 | 0 | 0 | -0.00295 | 0.19070 | -0.07840 |
| **Hala’ib Triangle** | Egypt/Sudan Disputed | 0.10208 | -0.10600 | -0.09591 | -0.03470 | 0.00161 | -0.00002 | 0.00109 | 0 | 0.00003 | -0.00290 | -0.00006 | 0.24706 | 0.09282 |
| **Tuvalu** | Tuvalu | 0.10177 | -0.00064 | -0.00199 | -0.00299 | -0.00004 | 0.00000 | 0 | 0 | 0 | 0 | -0.00438 | 0.12868 | -0.01672 |
| **Romania** | Romania | 0.09977 | -0.00112 | -0.00231 | -0.01056 | -0.00013 | -0.00009 | 0.00002 | -0.00168 | 0.00013 | 0 | -0.00004 | 0.25527 | -0.13861 |
| **Russia** | Russia | 0.09861 | 0.00051 | -0.00845 | 0.00219 | -0.00009 | 0.00002 | 0.00015 | 0.00000 | 0.00003 | 0.00000 | -0.00023 | 0.10138 | 0.01516 |
| **Sudan** | Sudan | 0.09830 | -0.03705 | -0.01205 | -0.00578 | 0.00117 | 0.00004 | 0.00038 | 0 | 0.00002 | -0.00041 | -0.00021 | 0.06526 | 0.08748 |
| **Turkey** | Turkey | 0.09345 | -0.01804 | -0.01751 | -0.04852 | 0.00047 | 0.00021 | -0.00048 | 0 | 0.00132 | 0.00004 | -0.00655 | 0.23841 | -0.05192 |
| **Latvia** | Latvia | 0.09151 | -0.00220 | -0.02061 | -0.01573 | -0.00029 | -0.00015 | 0.00126 | 0 | 0.00262 | 0 | -0.01447 | 0.22684 | -0.08341 |
| **Senegal** | Senegal | 0.09109 | -0.01555 | -0.01417 | -0.01277 | 0.00007 | 0.00001 | -0.00090 | 0 | -0.00011 | -0.00963 | -0.00231 | 0.16631 | -0.01957 |
| **Cyprus** | Cyprus | 0.08843 | -0.00104 | -0.00082 | -0.00066 | 0.00061 | 0.00005 | -0.00002 | 0 | -0.00045 | -0.00004 | -0.00026 | 0.11929 | -0.02756 |
| **Bahamas** | Bahamas | 0.08770 | -0.00284 | -0.00870 | -0.00179 | 0.00013 | -0.00008 | -0.00224 | 0 | -0.00018 | -0.00076 | -0.00024 | 0.04970 | 0.05484 |
| **Bosnia and Herzegovina** | Bosnia and Herzegovina | 0.08365 | -0.00296 | -0.00360 | -0.00723 | -0.02643 | 0.04317 | -0.04118 | 0 | -0.01230 | 0 | 0 | 0.10566 | 0.11306 |
| **New Zealand** | New Zealand | 0.08355 | -0.02588 | -0.00582 | -0.00235 | -0.00005 | -0.00001 | -0.00022 | 0 | -0.00030 | -0.00008 | -0.00023 | 0.10244 | 0.01626 |
| **Azores** | Portugal | 0.08225 | -0.00152 | -0.00020 | -0.00054 | -0.00002 | 0.00000 | -0.00002 | 0 | -0.00001 | 0 | -0.00042 | 0.08200 | 0.00302 |
| **China** | China | 0.07045 | -0.03532 | -0.03792 | -0.01389 | -0.00003 | 0.00029 | 0.00177 | -0.00016 | 0.00014 | -0.00143 | -0.00043 | 0.10922 | 0.04926 |
| **Cambodia** | Cambodia | 0.06553 | -0.10920 | -0.10290 | -0.04437 | -0.00037 | -0.00006 | 0.00081 | 0 | 0.00006 | -0.00001 | -0.00198 | 0.30066 | 0.02573 |
| **Israel** | Israel | 0.06501 | -0.02336 | -0.03310 | -0.01280 | 0.00021 | -0.00007 | 0.00020 | 0 | 0.00120 | -0.01121 | -0.00168 | 0.20394 | -0.05704 |
| **Sint-Maarten** | Netherlands | 0.05916 | -0.09400 | -0.08235 | -0.02876 | -0.00009 | -0.00092 | 0 | 0 | 0 | 0 | -0.01488 | 0.25918 | 0.02640 |
| **Italy** | Italy | 0.05895 | -0.01929 | -0.01354 | -0.01173 | -0.00008 | -0.00010 | -0.00283 | 0 | -0.00096 | 0 | -0.00313 | 0.13057 | -0.01926 |
| **Turks and Caicos Islands** | United Kingdom | 0.05495 | -0.00944 | -0.00614 | -0.00510 | 0.00009 | 0.00005 | 0 | 0 | 0 | -0.00184 | -0.00081 | 0.03586 | 0.04233 |
| **Syria** | Syria | 0.05382 | -0.00177 | -0.00207 | -0.00101 | 0.00033 | -0.00047 | 0.00165 | 0 | 0.00170 | -0.00012 | -0.00053 | 0.06017 | -0.00278 |
| **Jersey** | United Kingdom | 0.05286 | -0.04888 | -0.04113 | -0.01849 | 0.00004 | -0.00015 | -0.00115 | 0 | 0.00000 | 0 | -0.00065 | 0.19611 | -0.03203 |
| **Australia - Papua New Guinea** | Australia - Papua New Guinea | 0.05133 | -0.00089 | -0.00062 | -0.00044 | -0.00050 | -0.00010 | -0.00288 | 0 | -0.00010 | 0.00000 | -0.00002 | 0.05251 | 0.00481 |
| **Greece** | Greece | 0.04721 | 0.03259 | 0.02135 | 0.02278 | -0.00017 | -0.00035 | -0.00242 | 0 | -0.00035 | 0 | 0.00781 | -0.01191 | -0.02227 |
| **Japan - South Korea Conflict Zone** | Disputed | 0.03735 | -0.01708 | -0.01092 | -0.00699 | 0 | -0.00001 | 0 | 0 | 0 | 0 | -0.00077 | 0.07129 | 0.00183 |
| **Saudi Arabia** | Saudi Arabia | 0.03470 | -0.01811 | -0.01641 | -0.01005 | -0.00013 | 0.00033 | -0.00358 | -0.00028 | -0.00051 | -0.00297 | -0.00047 | 0.08394 | 0.00398 |
| **Gambia** | Gambia | 0.02772 | -0.01687 | -0.01515 | -0.01395 | 0.00007 | 0.00020 | -0.00178 | 0 | -0.00117 | -0.01545 | -0.00173 | 0.13791 | -0.04429 |
| **Antarctica** | Antarctica | 0.01886 | 0.00146 | -0.00001 | 0.00216 | 0 | 0 | 0 | 0 | 0 | 0 | 0.00173 | 0.04348 | -0.01554 |
| **Bahrain** | Bahrain | 0.00414 | -0.00294 | -0.00235 | -0.00117 | 0.00499 | 0.00005 | -0.00985 | -0.00046 | 0.00009 | -0.00102 | -0.00005 | 0.01494 | 0.00224 |
| **Line Group** | Kiribati | -0.00695 | -0.00019 | -0.00019 | -0.00158 | 0.00001 | 0.00000 | 0.00000 | 0 | 0.00000 | -0.00010 | -0.00230 | 0.00272 | -0.00533 |
| **Qatar** | Qatar | -0.00804 | -0.01910 | -0.01826 | -0.01035 | 0.00335 | 0.00045 | 0.00745 | -0.00101 | 0.00155 | -0.03048 | -0.00038 | 0.03963 | 0.01942 |
| **Canada** | Canada | -0.01093 | -0.01062 | -0.00556 | -0.00248 | -0.00001 | 0.00000 | 0.00004 | 0.00000 | 0.00002 | 0 | -0.00164 | 0.03934 | -0.02273 |
| **Namibia** | Namibia | -0.01244 | -0.00946 | -0.00398 | -0.03409 | 0.00000 | 0.00000 | -0.00001 | 0 | -0.00001 | 0 | -0.00017 | 0.05616 | -0.02077 |
| **Bangladesh** | Bangladesh | -0.01487 | -0.14132 | -0.16069 | -0.04555 | 0.00027 | -0.00009 | 0.00240 | 0 | 0.00069 | -0.00168 | -0.00066 | 0.35389 | -0.01648 |
| **Phoenix Group** | Kiribati | -0.02235 | -0.00034 | -0.00110 | -0.00422 | -0.00002 | 0 | 0 | 0 | 0 | -0.00010 | -0.00658 | 0.01499 | -0.02500 |
| **Belgium** | Belgium | -0.02587 | -0.04779 | -0.04396 | -0.01581 | 0.00026 | 0.00012 | -0.00355 | 0 | 0.00064 | 0 | -0.00164 | 0.09820 | -0.01016 |
| **Kerguelen Islands** | France | -0.02892 | 0.00112 | 0.00001 | 0.00000 | 0 | 0.00000 | 0 | 0 | 0 | 0 | 0.00001 | -0.03763 | 0.00754 |
| **North Korea** | North Korea | -0.03106 | -0.02090 | -0.01824 | -0.00878 | 0.00030 | -0.00017 | -0.00003 | 0 | 0.00002 | 0 | -0.00317 | 0.03895 | -0.01868 |
| **Eritrea** | Eritrea | -0.04317 | -0.00396 | -0.00261 | -0.00122 | -0.00183 | 0.00002 | 0.00006 | 0 | 0.00000 | 0.00000 | -0.00015 | -0.01116 | -0.02251 |
| **Tunisia** | Tunisia | -0.04385 | -0.09383 | -0.09685 | -0.04961 | 0.00021 | -0.00007 | -0.00024 | -0.00025 | -0.00056 | 0 | -0.01284 | 0.22843 | -0.01670 |
| **Kuwait** | Kuwait | -0.04554 | -0.03804 | -0.02796 | -0.01340 | 0.00171 | -0.00193 | -0.01819 | 0.00026 | -0.00466 | -0.01012 | -0.00090 | 0.06736 | 0.00188 |
| **Iran** | Iran | -0.05091 | -0.02165 | -0.01686 | -0.00819 | 0.00172 | 0.00008 | -0.00061 | -0.00038 | -0.00047 | -0.04838 | -0.00159 | 0.06058 | -0.01432 |
| **Guernsey** | United Kingdom | -0.05140 | -0.06031 | -0.05724 | -0.02584 | 0.00004 | -0.00032 | 0.00015 | 0 | 0.00002 | 0 | -0.01011 | 0.20194 | -0.09960 |
| **Greenland** | Denmark | -0.05359 | -0.01945 | -0.00536 | -0.00393 | 0.00017 | 0.00001 | 0.00000 | 0 | 0.00000 | 0 | -0.00023 | -0.04828 | 0.01208 |
| **Easter Island** | Chile | -0.05748 | 0.01410 | 0.00895 | 0.00951 | 0.00000 | 0.00000 | 0.00001 | 0 | 0.00000 | 0 | 0.01190 | -0.10851 | 0.00655 |
| **Estonia** | Estonia | -0.06215 | -0.00342 | -0.03439 | -0.03192 | -0.00324 | 0.00025 | -0.00081 | 0 | 0.00030 | 0 | -0.01768 | 0.06777 | -0.03752 |
| **Egypt** | Egypt | -0.06668 | -0.07702 | -0.09115 | -0.02587 | 0.00048 | -0.00011 | 0.00099 | -0.00049 | 0.00010 | -0.02835 | -0.00212 | 0.20604 | -0.04793 |
| **Argentina** | Argentina | -0.06810 | -0.07144 | -0.01772 | -0.01055 | 0.00002 | 0.00001 | 0.00000 | 0.00000 | 0.00004 | -0.00001 | -0.00013 | 0.00426 | 0.02744 |
| **Prince Edward Islands** | South Africa | -0.07698 | -0.00309 | -0.00009 | -0.00003 | 0 | 0 | -0.00003 | 0 | 0.00000 | 0 | -0.00010 | -0.05022 | -0.02343 |
| **Libya** | Libya | -0.08119 | -0.01750 | -0.03577 | -0.01105 | 0.00045 | -0.00003 | 0.00003 | 0.00003 | 0.00000 | -0.00025 | -0.00273 | 0.00631 | -0.02066 |
| **Norway** | Norway | -0.08625 | -0.01873 | -0.01556 | -0.00761 | 0.00024 | 0.00059 | 0.00019 | -0.00010 | 0.00000 | 0 | -0.01074 | -0.05870 | 0.01908 |
| **Ireland** | Ireland | -0.08825 | -0.07900 | -0.03668 | -0.01502 | 0.00036 | -0.00003 | 0.00144 | 0 | 0.00002 | 0 | -0.01572 | 0.09231 | -0.03553 |
| **Jarvis Island** | United States | -0.08829 | -0.00011 | -0.00014 | -0.00076 | 0.00000 | 0 | 0 | 0 | 0 | 0 | -0.00084 | -0.10942 | 0.02299 |
| **United Kingdom** | United Kingdom | -0.09450 | -0.05541 | -0.03788 | -0.01823 | -0.00023 | -0.00019 | 0.00069 | -0.00001 | 0.00013 | 0 | -0.02125 | 0.03436 | 0.00379 |
| **Sweden** | Sweden | -0.10210 | -0.01129 | -0.05896 | -0.03920 | 0.00140 | -0.00013 | -0.00232 | 0 | 0.00003 | 0 | -0.02804 | 0.10172 | -0.06281 |
| **United States** | United States | -0.11903 | -0.01055 | -0.00463 | -0.00470 | -0.00026 | -0.00016 | -0.00038 | 0.00000 | -0.00007 | -0.00054 | -0.00041 | -0.10482 | 0.00692 |
| **Falkland Islands** | United Kingdom | -0.12952 | -0.06454 | -0.00569 | -0.00333 | 0.00005 | 0.00001 | 0 | 0 | 0 | 0.00000 | -0.00003 | -0.08713 | 0.03104 |
| **Hawaii** | United States | -0.13413 | -0.00036 | -0.00070 | -0.00111 | -0.00001 | -0.00001 | 0.00010 | 0 | 0.00002 | -0.00016 | -0.00064 | -0.17992 | 0.04856 |
| **Finland** | Finland | -0.13657 | -0.00754 | -0.05007 | -0.03392 | 0.00051 | 0.00135 | 0.00048 | 0 | 0.00015 | 0 | -0.01316 | 0.02190 | -0.05558 |
| **Jan Mayen** | Norway | -0.13994 | -0.03787 | -0.00322 | -0.01531 | 0 | 0 | 0 | 0 | 0 | 0 | -0.00461 | -0.07555 | -0.00340 |
| **Marshall Islands** | Marshall Islands | -0.15421 | -0.00136 | -0.00403 | -0.00692 | 0.00002 | -0.00001 | 0 | 0 | 0 | 0 | -0.01145 | -0.13511 | 0.00464 |
| **Montenegro** | Montenegro | -0.15467 | 0.00724 | 0.00506 | 0.00360 | 0.00037 | 0.00098 | -0.02628 | 0 | -0.00155 | 0 | 0.00062 | -0.28897 | 0.14090 |
| **Howland Island and Baker Island** | United States | -0.17458 | -0.00025 | -0.00120 | -0.00347 | 0 | 0 | 0 | 0 | 0 | 0 | -0.00493 | -0.20033 | 0.03562 |
| **Johnston Atoll** | United States | -0.17688 | -0.00044 | -0.00039 | -0.00179 | 0.00005 | -0.00001 | 0 | 0 | 0 | 0 | -0.00268 | -0.31212 | 0.14051 |
| **Iceland** | Iceland | -0.18201 | -0.07257 | -0.04255 | -0.02983 | -0.00013 | 0.00002 | 0.00002 | 0 | 0.00000 | 0 | -0.01536 | 0.01877 | -0.04032 |
| **Germany** | Germany | -0.20714 | -0.11616 | -0.09384 | -0.04342 | -0.00037 | -0.00047 | -0.00270 | 0 | 0.00136 | 0 | -0.01180 | 0.07673 | -0.01525 |
| **United Arab Emirates** | United Arab Emirates | -0.22956 | -0.06835 | -0.06871 | -0.03827 | 0.00292 | -0.00016 | 0.00373 | -0.00006 | 0.00060 | -0.04863 | -0.00175 | -0.00911 | -0.00187 |
| **Netherlands** | Netherlands | -0.25262 | -0.14371 | -0.09677 | -0.04740 | 0.00143 | -0.00020 | -0.00250 | -0.00008 | 0.00071 | 0 | -0.00948 | 0.04993 | -0.00400 |
| **Denmark** | Denmark | -0.25289 | -0.13457 | -0.11858 | -0.05975 | 0.00023 | -0.00046 | -0.00205 | 0 | 0.00054 | 0 | -0.02271 | 0.09728 | -0.01094 |
| **Macquarie Island** | Australia | -0.25640 | -0.00004 | 0.00000 | 0.00000 | 0.00001 | 0 | 0.00000 | 0 | 0.00000 | 0 | 0.00000 | -0.27003 | 0.01365 |
| **Croatia** | Croatia | -0.28326 | -0.03957 | -0.02456 | -0.03259 | -0.00076 | 0.00001 | 0.00050 | 0 | 0.00013 | 0 | -0.00238 | -0.19528 | 0.00618 |
| **Iraq** | Iraq | -0.29371 | -0.08716 | -0.06539 | -0.02339 | 0.00125 | -0.00588 | -0.00650 | 0.00220 | -0.02083 | -0.00014 | -0.00024 | 0.12661 | -0.19853 |
| **Saint Pierre and Miquelon** | France | -0.30246 | -0.05483 | -0.04382 | -0.00931 | -0.00030 | -0.00010 | 0.00000 | 0 | 0.00000 | 0 | -0.00461 | 0.11876 | -0.30768 |
| **Gibraltar** | United Kingdom | -0.30522 | -0.02131 | -0.01798 | -0.01513 | 0.00065 | 0.00029 | -0.01043 | 0 | -0.00135 | 0 | -0.00419 | 0.23553 | -0.46988 |
| **Faeroe Islands** | Denmark | -0.31058 | -0.11835 | -0.04354 | -0.02256 | 0.00001 | 0.00008 | 0 | 0 | 0 | 0 | -0.03193 | -0.06933 | -0.02501 |
| **Kiribati** | Kiribati | -0.33717 | -0.00883 | -0.00632 | -0.01345 | -0.00004 | 0.00000 | 0 | 0 | 0 | -0.00003 | -0.01730 | -0.31269 | 0.02144 |
| **Bermuda** | United Kingdom | -0.36374 | -0.00023 | -0.00030 | -0.00118 | 0.00000 | 0.00000 | 0 | 0 | 0 | -0.00028 | -0.00044 | -0.35033 | -0.01098 |
| **Nauru** | Nauru | -0.36669 | -0.00146 | -0.00934 | -0.01423 | 0.00000 | 0.00000 | 0 | 0 | 0 | -0.00003 | -0.01475 | -0.35225 | 0.02536 |
| **Albania** | Albania | -0.37731 | -0.00584 | -0.00436 | -0.00215 | -0.00126 | 0.00074 | -0.00795 | 0 | -0.00104 | 0 | -0.00025 | -0.24806 | -0.11174 |
| **Slovenia** | Slovenia | -0.39932 | -0.12171 | -0.07954 | -0.10330 | 0.00184 | -0.00203 | -0.04680 | 0 | -0.01422 | 0 | -0.00632 | -0.03207 | 0 |
| **Crozet Islands** | France | -0.51315 | -0.00093 | -0.00007 | -0.00003 | 0 | 0.00000 | 0 | 0 | 0 | 0 | -0.00005 | -0.49858 | -0.01366 |
| **Alaska** | United States | -0.52029 | -0.02824 | -0.00778 | -0.00629 | 0.00003 | 0.00002 | 0.00000 | 0.00000 | 0.00001 | 0 | -0.00033 | -0.49204 | -0.01087 |
| **Palmyra Atoll** | United States | -0.61198 | -0.00044 | -0.00030 | -0.00089 | 0 | 0 | 0 | 0 | 0 | 0 | -0.00097 | -0.61095 | 0.00156 |
| **Pitcairn** | United Kingdom | -0.61611 | -0.00007 | -0.00017 | -0.00044 | -0.00002 | 0 | 0 | 0 | 0 | 0 | -0.00067 | -0.60840 | -0.00635 |
