# Supplementary material for: Spatial and temporal changes in cumulative human impacts on the world's ocean
Source: Nat Commun. 2015 Jul 14;6:7615. doi: 10.1038/ncomms8615 (PMC4510691; doi:10.1038/ncomms8615)
Supplement: Supplementary Data 3 — Average impact scores for each stressor and for cumulative impact in 2013 for each marine ecoregion of the world (MEOW; Spalding et al. 2007), in decreasing order of average cumulative impact. True zero values are indicated by zeros with no trailing decimals; very small values are zeros with several zero decimal values. [file ncomms8615-s4.doc]

## *Supplementary Data 3*

Average impact scores for each stressor and for cumulative impact in 2013 for each marine ecoregion of the world (MEOW; Spalding et al. 2007), in decreasing order of average cumulative impact. True zero values are indicated by zeros with no trailing decimals; very small values are zeros with several zero decimal values.

| **Suppl. Data 3:**  **2013 MEOW**  **Ecoregion** | **Province** | **Average cumulative impact score** | **Artisanal fishing** | **Demersal destructive fishing** | **Demersal nondestructive high bycatch fishing** | **Demersal nondestructive low bycatch fishing** | **Direct human impact** | **Inorganic pollution** | **Invasive species** | **Light pollution** | **Nutrient pollution** | **Ocean acidification** | **Ocean-based pollution** | **Oil rigs** | **Organic pollution** | **Pelagic high bycatch fishing** | **Pelagic low bycatch fishing** | **Sea level rise** | **Sea surface temperature** | **Shipping** | **UV** |
| --- | --- | --- | --- | --- | --- | --- | --- | --- | --- | --- | --- | --- | --- | --- | --- | --- | --- | --- | --- | --- | --- |
| **Southern China** | South China Sea | 5.3951 | 0.0021 | 0.6370 | 0.5967 | 0.1621 | 0.0345 | 0.0319 | 0.0551 | 0.0081 | 0.0380 | 0.7246 | 0.4512 | 0.0002 | 0.0045 | 0.0171 | 0.0037 | 0.3952 | 1.3210 | 0.4277 | 0.4943 |
| **East China Sea** | Warm Temperate Northwest Pacific | 5.3243 | 0.0010 | 0.5673 | 0.5004 | 0.1649 | 0.0310 | 0.0190 | 0.0277 | 0.0071 | 0.0214 | 0.7614 | 0.4125 | 0.0000 | 0.0031 | 0.0170 | 0.0104 | 0.2694 | 1.4743 | 0.4476 | 0.5998 |
| **Southern Norway** | Northern European Seas | 5.0881 | 0.0035 | 0.3062 | 0.3220 | 0.1318 | 0.0542 | 0.0129 | 0.0695 | 0.0115 | 0.0060 | 0.8768 | 0.3670 | 0.0006 | 0.0001 | 0 | 0.1687 | 0.1375 | 1.8895 | 0.3276 | 0.4565 |
| **Aegean Sea** | Mediterranean Sea | 4.9915 | 0.0030 | 0.1687 | 0.1049 | 0.1175 | 0.0517 | 0.0406 | 0.0671 | 0.0098 | 0.0292 | 1.0091 | 0.5227 | 0 | 0.0054 | 0 | 0.0242 | 0.1354 | 1.8223 | 0.4332 | 0.4879 |
| **North Sea** | Northern European Seas | 4.9006 | 0.0024 | 0.3572 | 0.2111 | 0.1051 | 0.0331 | 0.0321 | 0.1189 | 0.0074 | 0.0381 | 0.5987 | 0.4654 | 0.0003 | 0.0069 | 0 | 0.0972 | 0.3971 | 1.4339 | 0.4388 | 0.5731 |
| **Yellow Sea** | Cold Temperate Northwest Pacific | 4.8000 | 0.0010 | 0.4570 | 0.6078 | 0.2126 | 0.0423 | 0.0497 | 0.0650 | 0.0085 | 0.0696 | 0.3817 | 0.4410 | 0.0003 | 0.0146 | 0 | 0.0034 | 0.6450 | 0.9917 | 0.3764 | 0.4437 |
| **Celtic Seas** | Northern European Seas | 4.7981 | 0.0017 | 0.2489 | 0.1176 | 0.0542 | 0.0208 | 0.0117 | 0.0426 | 0.0034 | 0.0119 | 0.9386 | 0.3047 | 0 | 0.0015 | 0 | 0.0430 | 0.0998 | 1.8944 | 0.3485 | 0.6707 |
| **Northern Norway and Finnmark** | Northern European Seas | 4.7841 | 0.0013 | 0.2312 | 0.2500 | 0.1024 | 0.0295 | 0.0081 | 0.0338 | 0.0071 | 0.0006 | 0.9746 | 0.2981 | 0 | 0.0000 | 0 | 0.1300 | 0.1119 | 1.8702 | 0.2615 | 0.5172 |
| **Levantine Sea** | Mediterranean Sea | 4.7814 | 0.0006 | 0.0451 | 0.0602 | 0.0291 | 0.0136 | 0.0196 | 0.0264 | 0.0035 | 0.0204 | 1.1198 | 0.4822 | 0.0000 | 0.0049 | 0.0098 | 0.0085 | 0.0700 | 1.9475 | 0.3406 | 0.5926 |
| **Saharan Upwelling** | Lusitanian | 4.7532 | 0.0003 | 0.0956 | 0.1441 | 0.1854 | 0.0066 | 0.0090 | 0.0098 | 0.0008 | 0.0063 | 1.0359 | 0.4196 | 0 | 0.0014 | 0.0007 | 0.0078 | 0.0741 | 1.7701 | 0.3215 | 0.6701 |
| **South and West Iceland** | Northern European Seas | 4.7306 | 0.0008 | 0.2811 | 0.1681 | 0.1325 | 0.0060 | 0.0017 | 0.0116 | 0.0008 | 0.0018 | 0.9548 | 0.2466 | 0 | 0.0000 | 0 | 0.0338 | 0.0419 | 2.1193 | 0.2208 | 0.5200 |
| **South India and Sri Lanka** | West and South Indian Shelf | 4.6559 | 0.0009 | 0.1020 | 0.0847 | 0.0614 | 0.0090 | 0.0140 | 0.0080 | 0.0012 | 0.0156 | 1.0407 | 0.2235 | 0 | 0.0005 | 0.7153 | 0.0300 | 0.0702 | 1.5454 | 0.1399 | 0.5978 |
| **Faroe Plateau** | Northern European Seas | 4.6476 | 0.0003 | 0.2630 | 0.0953 | 0.0458 | 0.0040 | 0.0006 | 0.0092 | 0.0004 | 0 | 1.0820 | 0.2625 | 0 | 0 | 0 | 0.0662 | 0.0134 | 2.0093 | 0.2093 | 0.5880 |
| **Ionian Sea** | Mediterranean Sea | 4.6008 | 0.0017 | 0.0579 | 0.0482 | 0.0414 | 0.0175 | 0.0239 | 0.0211 | 0.0042 | 0.0140 | 1.1136 | 0.5523 | 0 | 0.0042 | 0 | 0.0155 | 0.0387 | 1.6455 | 0.4250 | 0.5861 |
| **Malacca Strait** | Sunda Shelf | 4.5570 | 0.0025 | 0.4947 | 0.3674 | 0.1921 | 0.0563 | 0.0531 | 0.1015 | 0.0078 | 0.0897 | 0.5470 | 0.3652 | 0.0001 | 0.0154 | 0.0013 | 0.0049 | 0.6255 | 1.0156 | 0.2717 | 0.3648 |
| **Alboran Sea** | Mediterranean Sea | 4.5501 | 0.0021 | 0.1083 | 0.1583 | 0.1202 | 0.0274 | 0.0539 | 0.0445 | 0.0071 | 0.0241 | 1.0132 | 0.5488 | 0 | 0.0034 | 0 | 0.0194 | 0.0511 | 1.4824 | 0.4588 | 0.4358 |
| **Eastern Philippines** | Western Coral Triangle | 4.5483 | 0.0158 | 0.1491 | 0.2766 | 0.1551 | 0.0384 | 0.0237 | 0.0219 | 0.0016 | 0.0127 | 1.1361 | 0.2237 | 0 | 0.0017 | 0.0017 | 0.0593 | 0.1069 | 1.6425 | 0.1342 | 0.5619 |
| **Black Sea** | Black Sea | 4.5394 | 0.0019 | 0.0128 | 0.0119 | 0.0579 | 0.0211 | 0.0375 | 0.0434 | 0.0033 | 0.0328 | 0.9712 | 0.4406 | 0.0001 | 0.0096 | 0.0001 | 0.0030 | 0.1815 | 1.8519 | 0.3384 | 0.5330 |
| **Central Kuroshio Current** | Warm Temperate Northwest Pacific | 4.5302 | 0.0022 | 0.1234 | 0.0826 | 0.0919 | 0.0252 | 0.0168 | 0.0499 | 0.0058 | 0.0107 | 1.0673 | 0.4107 | 0 | 0.0035 | 0.0000 | 0.0134 | 0.0678 | 1.7068 | 0.2925 | 0.5724 |
| **Eastern Caribbean** | Tropical Northwestern Atlantic | 4.5288 | 0.0007 | 0.0121 | 0.0152 | 0.0089 | 0.0046 | 0.0021 | 0.0064 | 0.0013 | 0.0002 | 1.1900 | 0.3198 | 0 | 0.0000 | 0.0021 | 0.0031 | 0.0167 | 2.1080 | 0.2023 | 0.6376 |
| **Sahelian Upwelling** | West African Transition | 4.5016 | 0.0003 | 0.0619 | 0.0820 | 0.0630 | 0.0068 | 0.0081 | 0.0082 | 0.0004 | 0.0027 | 1.0436 | 0.3317 | 0.0002 | 0.0004 | 0.0154 | 0.0043 | 0.0915 | 1.9436 | 0.2213 | 0.6218 |
| **Western India** | West and South Indian Shelf | 4.4671 | 0.0011 | 0.2734 | 0.1846 | 0.0882 | 0.0139 | 0.0305 | 0.0333 | 0.0024 | 0.0351 | 0.8899 | 0.3143 | 0.0010 | 0.0024 | 0 | 0.0050 | 0.1911 | 1.5663 | 0.2431 | 0.5982 |
| **Adriatic Sea** | Mediterranean Sea | 4.4477 | 0.0041 | 0.1630 | 0.0748 | 0.1096 | 0.0653 | 0.0789 | 0.1303 | 0.0141 | 0.0655 | 0.7167 | 0.4521 | 0 | 0.0196 | 0 | 0.0053 | 0.3831 | 1.2827 | 0.4292 | 0.4782 |
| **Southern Caribbean** | Tropical Northwestern Atlantic | 4.4274 | 0.0014 | 0.0369 | 0.0110 | 0.0348 | 0.0120 | 0.0106 | 0.0207 | 0.0022 | 0.0116 | 1.1217 | 0.3354 | 0.0000 | 0.0030 | 0.0005 | 0.0015 | 0.0652 | 1.9794 | 0.2422 | 0.5462 |
| **Cape Verde** | West African Transition | 4.3918 | 0.0001 | 0.0020 | 0.0008 | 0.0044 | 0.0015 | 0.0005 | 0.0006 | 0.0001 | 0.0001 | 1.1501 | 0.2529 | 0 | 0.0000 | 0 | 0.0048 | 0.0044 | 2.1068 | 0.1453 | 0.7189 |
| **Gulf of Tonkin** | South China Sea | 4.3837 | 0.0033 | 0.4392 | 0.4066 | 0.0985 | 0.0357 | 0.0348 | 0.0389 | 0.0048 | 0.0631 | 0.7925 | 0.2654 | 0.0004 | 0.0077 | 0.0121 | 0.0037 | 0.3395 | 1.1976 | 0.2340 | 0.4173 |
| **South European Atlantic Shelf** | Lusitanian | 4.3747 | 0.0010 | 0.0969 | 0.0619 | 0.0628 | 0.0115 | 0.0165 | 0.0233 | 0.0033 | 0.0123 | 1.0709 | 0.4526 | 0 | 0.0047 | 0 | 0.0187 | 0.0441 | 1.4734 | 0.3810 | 0.6449 |
| **Western Mediterranean** | Mediterranean Sea | 4.3740 | 0.0015 | 0.0544 | 0.0556 | 0.0551 | 0.0182 | 0.0337 | 0.0326 | 0.0049 | 0.0172 | 1.0904 | 0.5126 | 0 | 0.0049 | 0 | 0.0093 | 0.0381 | 1.4680 | 0.4293 | 0.5554 |
| **Azores Canaries Madeira** | Lusitanian | 4.3586 | 0.0000 | 0.0096 | 0.0076 | 0.0106 | 0.0017 | 0.0010 | 0.0006 | 0.0005 | 0.0007 | 1.1589 | 0.3720 | 0 | 0.0001 | 0.0001 | 0.0035 | 0.0031 | 1.7770 | 0.2395 | 0.7738 |
| **Gulf of Oman** | Somali/Arabian | 4.3236 | 0.0009 | 0.1122 | 0.0481 | 0.0320 | 0.0117 | 0.0098 | 0.0132 | 0.0022 | 0.0023 | 1.0031 | 0.4485 | 0.0001 | 0.0001 | 0.0620 | 0.0059 | 0.0890 | 1.6788 | 0.3186 | 0.4962 |
| **South Kuroshio** | South Kuroshio | 4.3141 | 0.0009 | 0.1084 | 0.1089 | 0.0644 | 0.0045 | 0.0014 | 0.0032 | 0.0007 | 0.0014 | 1.1652 | 0.3618 | 0 | 0.0002 | 0.0188 | 0.0262 | 0.0134 | 1.5229 | 0.2343 | 0.6793 |
| **Sunda Shelf/Java Sea** | Sunda Shelf | 4.3091 | 0.0039 | 0.5023 | 0.3659 | 0.2017 | 0.0149 | 0.0157 | 0.0113 | 0.0009 | 0.0211 | 0.6106 | 0.2234 | 0.0016 | 0.0035 | 0.0072 | 0.0348 | 0.5860 | 1.0809 | 0.1993 | 0.4280 |
| **Gulf of Guinea Upwelling** | Gulf of Guinea | 4.2886 | 0.0001 | 0.0601 | 0.0304 | 0.0565 | 0.0040 | 0.0096 | 0.0100 | 0.0005 | 0.0042 | 1.1005 | 0.2516 | 0.0003 | 0.0009 | 0.1079 | 0.0080 | 0.0545 | 1.8072 | 0.1533 | 0.6335 |
| **Southern Vietnam** | Sunda Shelf | 4.2847 | 0.0013 | 0.4817 | 0.4417 | 0.1532 | 0.0216 | 0.0244 | 0.0223 | 0.0027 | 0.0379 | 0.6102 | 0.3568 | 0.0032 | 0.0070 | 0.0024 | 0.0099 | 0.6246 | 0.9525 | 0.1995 | 0.3388 |
| **Ogasawara Islands** | Tropical Northwestern Pacific | 4.2822 | 0.0000 | 0.0414 | 0.0660 | 0.0527 | 0.0002 | 0.0000 | 0 | 0.0000 | 0 | 1.1883 | 0.2384 | 0 | 0 | 0.0067 | 0.0272 | 0.0007 | 1.7934 | 0.1414 | 0.7258 |
| **Western Caribbean** | Tropical Northwestern Atlantic | 4.2763 | 0.0069 | 0.0760 | 0.0473 | 0.0220 | 0.0156 | 0.0117 | 0.0165 | 0.0019 | 0.0082 | 1.1576 | 0.3167 | 0 | 0.0041 | 0.0159 | 0.0006 | 0.0746 | 1.7862 | 0.2217 | 0.5013 |
| **Palawan/North Borneo** | Western Coral Triangle | 4.2652 | 0.0068 | 0.2655 | 0.3206 | 0.1399 | 0.0336 | 0.0140 | 0.0231 | 0.0011 | 0.0131 | 0.9984 | 0.2017 | 0.0017 | 0.0019 | 0.0052 | 0.0343 | 0.2551 | 1.3609 | 0.1650 | 0.4354 |
| **Greater Antilles** | Tropical Northwestern Atlantic | 4.2645 | 0.0019 | 0.0297 | 0.0199 | 0.0104 | 0.0136 | 0.0137 | 0.0176 | 0.0016 | 0.0059 | 1.1589 | 0.3367 | 0 | 0.0010 | 0.0054 | 0.0008 | 0.0408 | 1.8663 | 0.2122 | 0.5391 |
| **South China Sea Oceanic Islands** | South China Sea | 4.2385 | 0 | 0.1300 | 0.1829 | 0.0894 | 0 | 0 | 0 | 0.0000 | 0 | 1.1091 | 0.3239 | 0 | 0 | 0.0101 | 0.0395 | 0.0127 | 1.5303 | 0.2267 | 0.5837 |
| **Northeastern Brazil** | Tropical Southwestern Atlantic | 4.2327 | 0.0006 | 0.1156 | 0.0495 | 0.0341 | 0.0060 | 0.0132 | 0.0136 | 0.0013 | 0.0176 | 1.0311 | 0.2989 | 0.0000 | 0.0019 | 0.0897 | 0.0097 | 0.0985 | 1.6985 | 0.1753 | 0.5822 |
| **Manning-Hawkesbury** | East Central Australian Shelf | 4.2285 | 0.0032 | 0.0266 | 0.0117 | 0.0070 | 0.0100 | 0.0092 | 0.0061 | 0.0020 | 0.0055 | 1.1325 | 0.3189 | 0 | 0.0007 | 0.0003 | 0.0018 | 0.0263 | 1.6999 | 0.2199 | 0.7516 |
| **Tunisian Plateau/Gulf of Sidra** | Mediterranean Sea | 4.2265 | 0.0012 | 0.0464 | 0.0665 | 0.0253 | 0.0108 | 0.0119 | 0.0303 | 0.0023 | 0.0071 | 1.0484 | 0.3079 | 0.0017 | 0.0010 | 0 | 0.0057 | 0.1061 | 1.7864 | 0.2376 | 0.5355 |
| **Gulf of Guinea Central** | Gulf of Guinea | 4.2240 | 0.0047 | 0.1297 | 0.1493 | 0.0655 | 0.0122 | 0.0188 | 0.0372 | 0.0015 | 0.0071 | 1.0219 | 0.2514 | 0.0530 | 0.0023 | 0.2647 | 0.0120 | 0.1204 | 1.4960 | 0.2038 | 0.3818 |
| **Fernando de Naronha and Atoll das Rocas** | Tropical Southwestern Atlantic | 4.2204 | 0.0000 | 0.0105 | 0.0100 | 0.0078 | 0.0001 | 0.0000 | 0 | 0.0000 | 0 | 1.0881 | 0.2407 | 0 | 0 | 0.0620 | 0.0072 | 0.0008 | 1.9683 | 0.1375 | 0.6876 |
| **Southwestern Caribbean** | Tropical Northwestern Atlantic | 4.1631 | 0.0022 | 0.0264 | 0.0208 | 0.0042 | 0.0073 | 0.0081 | 0.0090 | 0.0005 | 0.0059 | 1.0814 | 0.3178 | 0 | 0.0032 | 0.0003 | 0.0002 | 0.1109 | 1.8195 | 0.2276 | 0.5214 |
| **Gulf of Guinea West** | Gulf of Guinea | 4.1603 | 0.0003 | 0.0429 | 0.0604 | 0.0278 | 0.0088 | 0.0060 | 0.0097 | 0.0001 | 0.0023 | 1.0078 | 0.2557 | 0 | 0.0002 | 0.0232 | 0.0053 | 0.1812 | 1.7560 | 0.1595 | 0.6204 |
| **Natal** | Agulhas | 4.1570 | 0.0003 | 0.0142 | 0.0020 | 0.0166 | 0.0053 | 0.0106 | 0.0054 | 0.0006 | 0.0061 | 1.1580 | 0.2643 | 0 | 0.0010 | 0.0009 | 0.0022 | 0.0250 | 1.7449 | 0.1660 | 0.7380 |
| **Southeastern Brazil** | Warm Temperate Southwestern Atlantic | 4.1309 | 0.0011 | 0.0809 | 0.0515 | 0.0156 | 0.0121 | 0.0099 | 0.0294 | 0.0030 | 0.0059 | 1.0203 | 0.2941 | 0.0000 | 0.0004 | 0.0181 | 0.0126 | 0.0924 | 1.5563 | 0.2643 | 0.6694 |
| **Andaman Sea Coral Coast** | Andaman | 4.1045 | 0.0041 | 0.4439 | 0.3187 | 0.1457 | 0.0302 | 0.0098 | 0.0131 | 0.0020 | 0.0079 | 0.7871 | 0.0915 | 0.0000 | 0.0008 | 0.0012 | 0.0024 | 0.3927 | 1.3426 | 0.0782 | 0.4425 |
| **Mariana Islands** | Tropical Northwestern Pacific | 4.0999 | 0.0003 | 0.0045 | 0.0139 | 0.0325 | 0.0005 | 0.0003 | 0.0003 | 0.0001 | 0 | 1.1749 | 0.2124 | 0 | 0 | 0 | 0.0464 | 0.0013 | 1.7959 | 0.1258 | 0.6913 |
| **Gulf of Maine/Bay of Fundy** | Cold Temperate Northwest Atlantic | 4.0988 | 0.0053 | 0.2094 | 0.0915 | 0.0572 | 0.0262 | 0.0171 | 0.0504 | 0.0050 | 0.0071 | 0.7369 | 0.3239 | 0 | 0.0012 | 0 | 0.0281 | 0.1282 | 1.4871 | 0.3316 | 0.6067 |
| **Southern Grand Banks - South Newfoundland** | Cold Temperate Northwest Atlantic | 4.0946 | 0.0008 | 0.2065 | 0.1031 | 0.0337 | 0.0039 | 0.0011 | 0.0075 | 0.0005 | 0.0008 | 0.7391 | 0.2979 | 0.0003 | 0.0000 | 0 | 0.0132 | 0.0770 | 1.5076 | 0.3615 | 0.7472 |
| **Eastern India** | Bay of Bengal | 4.0879 | 0.0006 | 0.1724 | 0.1078 | 0.0675 | 0.0074 | 0.0269 | 0.0112 | 0.0011 | 0.0290 | 1.0068 | 0.1474 | 0.0002 | 0.0017 | 0.0337 | 0.0089 | 0.0556 | 1.7289 | 0.0833 | 0.6015 |
| **Halmahera** | Western Coral Triangle | 4.0845 | 0.0009 | 0.1329 | 0.1202 | 0.1448 | 0.0201 | 0.0050 | 0.0049 | 0.0002 | 0.0018 | 1.0601 | 0.1540 | 0 | 0.0000 | 0.0037 | 0.0522 | 0.1380 | 1.6107 | 0.0925 | 0.5519 |
| **Tweed-Moreton** | East Central Australian Shelf | 4.0777 | 0.0021 | 0.0219 | 0.0086 | 0.0130 | 0.0108 | 0.0069 | 0.0009 | 0.0016 | 0.0078 | 1.0963 | 0.2765 | 0 | 0.0007 | 0.0005 | 0.0136 | 0.0973 | 1.6373 | 0.1909 | 0.6975 |
| **Southeast Madagascar** | Western Indian Ocean | 4.0760 | 0.0006 | 0.0398 | 0.0081 | 0.0102 | 0.0049 | 0.0064 | 0.0018 | 0.0000 | 0.0004 | 1.1548 | 0.1672 | 0 | 0.0000 | 0 | 0.0074 | 0.0330 | 1.8450 | 0.0995 | 0.7020 |
| **Gulf of Thailand** | Sunda Shelf | 4.0682 | 0.0030 | 0.4138 | 0.3219 | 0.1447 | 0.0260 | 0.0254 | 0.0456 | 0.0041 | 0.0288 | 0.5212 | 0.2476 | 0.0006 | 0.0059 | 0.0001 | 0.0044 | 0.6221 | 1.0407 | 0.2005 | 0.4205 |
| **Northern and Central Red Sea** | Red Sea and Gulf of Aden | 4.0676 | 0.0019 | 0.1161 | 0.0792 | 0.0324 | 0.0233 | 0.0142 | 0.0402 | 0.0065 | 0.0035 | 1.1193 | 0.2985 | 0.0008 | 0.0002 | 0.0011 | 0.0004 | 0.1893 | 1.6474 | 0.2475 | 0.2696 |
| **Sao Pedro and Sao Paulo Islands** | Tropical Southwestern Atlantic | 4.0396 | 0 | 0.0039 | 0.0025 | 0.0027 | 0 | 0 | 0 | 0 | 0 | 1.1324 | 0.2113 | 0 | 0 | 0 | 0.0040 | 0 | 1.9025 | 0.1300 | 0.6504 |
| **Mascarene Islands** | Western Indian Ocean | 4.0262 | 0.0001 | 0.0027 | 0.0023 | 0.0058 | 0.0008 | 0.0009 | 0.0004 | 0.0003 | 0.0005 | 1.2115 | 0.1670 | 0 | 0.0003 | 0 | 0.0079 | 0.0024 | 1.8408 | 0.0997 | 0.6832 |
| **Puget Trough/Georgia Basin** | Cold Temperate Northeast Pacific | 4.0133 | 0.0504 | 0.0834 | 0.0183 | 0.1039 | 0.3432 | 0.1638 | 0.4577 | 0.0823 | 0.0531 | 0.4709 | 0.4683 | 0 | 0.0084 | 0 | 0.0012 | 0.2847 | 0.8115 | 0.4217 | 0.3275 |
| **Scotian Shelf** | Cold Temperate Northwest Atlantic | 4.0036 | 0.0009 | 0.1117 | 0.0718 | 0.0466 | 0.0069 | 0.0029 | 0.0147 | 0.0012 | 0.0004 | 0.8428 | 0.3601 | 0 | 0.0000 | 0 | 0.0394 | 0.0691 | 1.3614 | 0.3185 | 0.7596 |
| **Sulawesi Sea/Makassar Strait** | Western Coral Triangle | 4.0006 | 0.0024 | 0.2100 | 0.1586 | 0.1271 | 0.0129 | 0.0093 | 0.0107 | 0.0005 | 0.0083 | 0.9987 | 0.1828 | 0.0001 | 0.0006 | 0.0031 | 0.0484 | 0.1798 | 1.4073 | 0.1174 | 0.5273 |
| **Southern Gulf of Mexico** | Tropical Northwestern Atlantic | 3.9828 | 0.0004 | 0.0995 | 0.0641 | 0.0287 | 0.0048 | 0.0088 | 0.0117 | 0.0006 | 0.0110 | 1.0093 | 0.2623 | 0.0019 | 0.0039 | 0.0176 | 0.0011 | 0.1470 | 1.5408 | 0.1696 | 0.6021 |
| **Guianan** | North Brazil Shelf | 3.9793 | 0.0002 | 0.0518 | 0.0141 | 0.0136 | 0.0083 | 0.0072 | 0.0131 | 0.0008 | 0.0084 | 0.9363 | 0.1978 | 0.0002 | 0.0014 | 0.0079 | 0.0006 | 0.2525 | 1.7724 | 0.1354 | 0.5670 |
| **Sea of Japan/East Sea** | Cold Temperate Northwest Pacific | 3.9246 | 0.0012 | 0.1923 | 0.0825 | 0.0869 | 0.0146 | 0.0123 | 0.0243 | 0.0027 | 0.0083 | 0.8490 | 0.2692 | 0 | 0.0029 | 0 | 0.0044 | 0.0530 | 1.5866 | 0.1907 | 0.5527 |
| **Houtman** | West Central Australian Shelf | 3.9096 | 0.0002 | 0.0092 | 0.0162 | 0.0028 | 0.0034 | 0.0011 | 0.0051 | 0.0004 | 0.0057 | 1.0317 | 0.2715 | 0 | 0.0009 | 0.0010 | 0.0007 | 0.1358 | 1.5227 | 0.1812 | 0.7233 |
| **Western Arabian Sea** | Somali/Arabian | 3.9094 | 0.0001 | 0.0348 | 0.0128 | 0.0175 | 0.0042 | 0.0031 | 0.0015 | 0.0004 | 0.0008 | 1.0141 | 0.2943 | 0 | 0.0001 | 0.0039 | 0.0065 | 0.0419 | 1.7609 | 0.1877 | 0.5312 |
| **Bahamian** | Tropical Northwestern Atlantic | 3.9030 | 0.0009 | 0.0115 | 0.0164 | 0.0054 | 0.0053 | 0.0006 | 0.0016 | 0.0006 | 0.0002 | 1.1240 | 0.3709 | 0 | 0.0000 | 0.0027 | 0.0009 | 0.0604 | 1.4549 | 0.2405 | 0.6095 |
| **Ningaloo** | Northwest Australian Shelf | 3.8920 | 0.0000 | 0.0065 | 0.0050 | 0.0030 | 0.0010 | 0.0000 | 0.0013 | 0.0000 | 0.0000 | 1.0956 | 0.2333 | 0.0002 | 0.0000 | 0.0588 | 0.0015 | 0.0326 | 1.7159 | 0.1602 | 0.5810 |
| **Northern Grand Banks - Southern Labrador** | Arctic | 3.8919 | 0.0021 | 0.2468 | 0.1189 | 0.0297 | 0.0132 | 0.0032 | 0.0166 | 0.0020 | 0.0011 | 0.7120 | 0.1648 | 0 | 0.0001 | 0 | 0.0172 | 0.0851 | 1.7874 | 0.1508 | 0.5615 |
| **Gulf of Aden** | Red Sea and Gulf of Aden | 3.8917 | 0.0004 | 0.0541 | 0.0191 | 0.0233 | 0.0069 | 0.0062 | 0.0074 | 0.0003 | 0.0007 | 1.0528 | 0.2892 | 0 | 0.0000 | 0.0482 | 0.0129 | 0.0364 | 1.6331 | 0.1946 | 0.5108 |
| **Southern Cook/Austral Islands** | Southeast Polynesia | 3.8870 | 0.0000 | 0.0003 | 0.0002 | 0.0006 | 0.0002 | 0 | 0.0000 | 0.0000 | 0.0000 | 1.2262 | 0.0708 | 0 | 0.0000 | 0 | 0.0008 | 0.0007 | 1.8199 | 0.0399 | 0.7274 |
| **Tonga Islands** | Tropical Southwestern Pacific | 3.8845 | 0.0003 | 0.0016 | 0.0022 | 0.0015 | 0.0016 | 0.0000 | 0.0010 | 0.0000 | 0.0001 | 1.2278 | 0.0805 | 0 | 0.0000 | 0 | 0.0011 | 0.0078 | 1.8754 | 0.0500 | 0.6339 |
| **Fiji Islands** | Tropical Southwestern Pacific | 3.8786 | 0.0026 | 0.0053 | 0.0044 | 0.0033 | 0.0075 | 0.0012 | 0.0031 | 0.0002 | 0.0004 | 1.2169 | 0.1051 | 0 | 0.0000 | 0 | 0.0010 | 0.0491 | 1.8711 | 0.0658 | 0.5465 |
| **Northeastern Honshu** | Cold Temperate Northwest Pacific | 3.8644 | 0.0011 | 0.0989 | 0.0592 | 0.0742 | 0.0129 | 0.0142 | 0.0218 | 0.0034 | 0.0095 | 1.0381 | 0.3668 | 0 | 0.0042 | 0 | 0.0111 | 0.0339 | 1.2931 | 0.2599 | 0.5670 |
| **Samoa Islands** | Central Polynesia | 3.8621 | 0.0003 | 0.0136 | 0.0079 | 0.0043 | 0.0016 | 0 | 0.0008 | 0.0001 | 0.0000 | 1.2119 | 0.0713 | 0 | 0.0000 | 0 | 0.0049 | 0.0038 | 1.8872 | 0.0439 | 0.6115 |
| **Gulf of St. Lawrence - Eastern Scotian Shelf** | Cold Temperate Northwest Atlantic | 3.8603 | 0.0037 | 0.1329 | 0.1209 | 0.0504 | 0.0234 | 0.0110 | 0.0399 | 0.0055 | 0.0061 | 0.6035 | 0.2707 | 0 | 0.0009 | 0 | 0.0315 | 0.1678 | 1.6141 | 0.2431 | 0.5533 |
| **Lesser Sunda** | Western Coral Triangle | 3.8551 | 0.0063 | 0.1255 | 0.0932 | 0.0640 | 0.0214 | 0.0135 | 0.0046 | 0.0006 | 0.0117 | 1.0990 | 0.1739 | 0 | 0.0008 | 0.0020 | 0.0125 | 0.0474 | 1.4876 | 0.1090 | 0.5917 |
| **South Australian Gulfs** | Southwest Australian Shelf | 3.8528 | 0.0008 | 0.0321 | 0.0325 | 0.0058 | 0.0131 | 0.0026 | 0.0291 | 0.0007 | 0.0168 | 0.7757 | 0.1594 | 0 | 0.0016 | 0.0006 | 0.0005 | 0.3087 | 1.7451 | 0.1162 | 0.6277 |
| **Papua** | Western Coral Triangle | 3.8519 | 0.0010 | 0.1192 | 0.1213 | 0.1065 | 0.0165 | 0.0072 | 0.0053 | 0.0002 | 0.0025 | 1.0509 | 0.0829 | 0.0000 | 0.0001 | 0.0021 | 0.0515 | 0.1602 | 1.5100 | 0.0460 | 0.5756 |
| **North and East Iceland** | Arctic | 3.8421 | 0.0001 | 0.1575 | 0.0941 | 0.0980 | 0.0012 | 0.0001 | 0.0032 | 0.0001 | 0.0004 | 1.0050 | 0.1369 | 0 | 0.0000 | 0 | 0.0227 | 0.0092 | 1.7315 | 0.1148 | 0.4697 |
| **Solomon Sea** | Eastern Coral Triangle | 3.8388 | 0.0030 | 0.0118 | 0.0063 | 0.0671 | 0.0106 | 0.0030 | 0.0033 | 0.0000 | 0.0005 | 1.0955 | 0.1825 | 0 | 0.0001 | 0 | 0.1021 | 0.0937 | 1.6230 | 0.1162 | 0.5251 |
| **Lord Howe and Norfolk Islands** | Lord Howe and Norfolk Islands | 3.8378 | 0.0000 | 0.0069 | 0.0030 | 0.0010 | 0.0001 | 0 | 0.0003 | 0.0000 | 0 | 1.2303 | 0.1931 | 0 | 0 | 0 | 0.0003 | 0.0021 | 1.5721 | 0.1157 | 0.7130 |
| **Leeuwin** | Southwest Australian Shelf | 3.8312 | 0.0002 | 0.0094 | 0.0098 | 0.0025 | 0.0024 | 0.0009 | 0.0068 | 0.0003 | 0.0045 | 1.0094 | 0.1769 | 0 | 0.0008 | 0.0004 | 0.0008 | 0.0786 | 1.6568 | 0.1138 | 0.7598 |
| **Society Islands** | Southeast Polynesia | 3.8279 | 0.0008 | 0.0012 | 0.0001 | 0.0003 | 0.0019 | 0.0001 | 0.0006 | 0.0004 | 0.0000 | 1.2220 | 0.0852 | 0 | 0.0000 | 0 | 0.0004 | 0.0031 | 1.7897 | 0.0500 | 0.6729 |
| **Western Bassian** | Southeast Australian Shelf | 3.8270 | 0.0001 | 0.0195 | 0.0148 | 0.0029 | 0.0029 | 0.0015 | 0.0041 | 0.0001 | 0.0049 | 0.9449 | 0.1449 | 0 | 0.0012 | 0.0002 | 0.0006 | 0.1022 | 1.7820 | 0.0992 | 0.7044 |
| **Maldives** | Central Indian Ocean Islands | 3.8170 | 0.0012 | 0.0356 | 0.0205 | 0.0306 | 0.0042 | 0.0000 | 0.0003 | 0.0004 | 0.0000 | 1.1056 | 0.1740 | 0 | 0.0000 | 0.0001 | 0.0342 | 0.0240 | 1.6049 | 0.1133 | 0.6681 |
| **Vanuatu** | Tropical Southwestern Pacific | 3.8110 | 0.0008 | 0.0018 | 0.0017 | 0.0088 | 0.0029 | 0.0001 | 0.0004 | 0.0000 | 0.0000 | 1.2238 | 0.0921 | 0 | 0.0000 | 0.0001 | 0.0124 | 0.0058 | 1.7633 | 0.0550 | 0.6437 |
| **Angolan** | Gulf of Guinea | 3.8107 | 0.0005 | 0.1051 | 0.0664 | 0.0228 | 0.0053 | 0.0098 | 0.0075 | 0.0005 | 0.0009 | 1.0683 | 0.1587 | 0.0037 | 0.0001 | 0.0000 | 0.0016 | 0.0360 | 1.6498 | 0.1108 | 0.5670 |
| **Agulhas Bank** | Agulhas | 3.8016 | 0.0001 | 0.0215 | 0.0048 | 0.0741 | 0.0036 | 0.0034 | 0.0038 | 0.0006 | 0.0032 | 1.0861 | 0.1763 | 0.0001 | 0.0004 | 0 | 0.0006 | 0.0370 | 1.5036 | 0.1633 | 0.7217 |
| **Delagoa** | Western Indian Ocean | 3.8003 | 0.0006 | 0.0269 | 0.0026 | 0.0064 | 0.0068 | 0.0041 | 0.0047 | 0.0002 | 0.0025 | 1.1163 | 0.1598 | 0 | 0.0006 | 0.0016 | 0.0023 | 0.0435 | 1.7154 | 0.1038 | 0.6070 |
| **Northern Bay of Bengal** | Bay of Bengal | 3.7987 | 0.0019 | 0.2508 | 0.1899 | 0.0722 | 0.0216 | 0.0275 | 0.0074 | 0.0004 | 0.0261 | 0.8360 | 0.0423 | 0.0000 | 0.0039 | 0.0053 | 0.0044 | 0.2318 | 1.5248 | 0.0281 | 0.5369 |
| **Andaman and Nicobar Islands** | Andaman | 3.7965 | 0.0003 | 0.0984 | 0.0859 | 0.0406 | 0.0047 | 0.0007 | 0.0003 | 0.0001 | 0.0014 | 1.0532 | 0.0860 | 0 | 0.0000 | 0.0132 | 0.0088 | 0.0349 | 1.6756 | 0.0528 | 0.6417 |
| **Western and Northern Madagascar** | Western Indian Ocean | 3.7938 | 0.0009 | 0.0381 | 0.0101 | 0.0119 | 0.0076 | 0.0060 | 0.0041 | 0.0001 | 0.0007 | 1.0986 | 0.0998 | 0 | 0.0000 | 0.0009 | 0.0084 | 0.0525 | 1.7375 | 0.0567 | 0.6652 |
| **West Caroline Islands** | Tropical Northwestern Pacific | 3.7886 | 0.0004 | 0.0027 | 0.0080 | 0.0100 | 0.0006 | 0.0000 | 0.0006 | 0.0000 | 0.0000 | 1.1613 | 0.1423 | 0 | 0.0000 | 0.0002 | 0.0112 | 0.0057 | 1.6455 | 0.0792 | 0.7208 |
| **Cocos-Keeling/Christmas Island** | Java Transitional | 3.7812 | 0.0000 | 0.0019 | 0.0045 | 0.0044 | 0.0002 | 0.0000 | 0.0000 | 0.0000 | 0 | 1.1237 | 0.1273 | 0 | 0 | 0.0000 | 0.0043 | 0.0008 | 1.6753 | 0.0709 | 0.7678 |
| **Shark Bay** | West Central Australian Shelf | 3.7738 | 0.0000 | 0.0109 | 0.0103 | 0.0032 | 0.0029 | 0.0002 | 0.0057 | 0.0001 | 0.0006 | 0.9865 | 0.2233 | 0 | 0.0000 | 0.0065 | 0.0008 | 0.1687 | 1.5276 | 0.1833 | 0.6511 |
| **Cape Howe** | Southeast Australian Shelf | 3.7650 | 0.0008 | 0.0197 | 0.0141 | 0.0052 | 0.0083 | 0.0026 | 0.0047 | 0.0005 | 0.0044 | 0.9895 | 0.2463 | 0.0003 | 0.0004 | 0.0003 | 0.0007 | 0.1228 | 1.5619 | 0.1746 | 0.6147 |
| **Baltic Sea** | Northern European Seas | 3.7416 | 0.0034 | 0.0151 | 0.1344 | 0.1125 | 0.0704 | 0.0557 | 0.2183 | 0.0129 | 0.0483 | 0.0124 | 0.4638 | 0 | 0.0055 | 0 | 0.0475 | 0.7176 | 1.0641 | 0.3721 | 0.4130 |
| **Eastern Brazil** | Tropical Southwestern Atlantic | 3.7299 | 0.0020 | 0.0795 | 0.0390 | 0.0205 | 0.0054 | 0.0102 | 0.0092 | 0.0012 | 0.0093 | 1.1080 | 0.2407 | 0.0027 | 0.0010 | 0.0388 | 0.0178 | 0.0850 | 1.2108 | 0.1721 | 0.6793 |
| **Cargados Carajos/Tromelin Island** | Western Indian Ocean | 3.7292 | 0.0000 | 0.0465 | 0.0210 | 0.0191 | 0.0001 | 0 | 0 | 0 | 0 | 1.0890 | 0.0830 | 0 | 0 | 0.0039 | 0.0119 | 0.0231 | 1.7872 | 0.0497 | 0.5947 |
| **Exmouth to Broome** | Northwest Australian Shelf | 3.7276 | 0.0001 | 0.0589 | 0.0308 | 0.0119 | 0.0023 | 0.0003 | 0.0105 | 0.0002 | 0.0004 | 0.9261 | 0.1988 | 0.0002 | 0.0000 | 0.0104 | 0.0013 | 0.2047 | 1.5492 | 0.1770 | 0.5493 |
| **Amazonia** | North Brazil Shelf | 3.7200 | 0.0003 | 0.1025 | 0.0656 | 0.0318 | 0.0165 | 0.0106 | 0.0120 | 0.0007 | 0.0160 | 0.7389 | 0.2037 | 0 | 0.0023 | 0.0521 | 0.0045 | 0.4041 | 1.4485 | 0.1193 | 0.5015 |
| **Arabian (Persian) Gulf** | Somali/Arabian | 3.7175 | 0.0024 | 0.1346 | 0.1193 | 0.0646 | 0.0380 | 0.0280 | 0.1604 | 0.0132 | 0.0201 | 0.4908 | 0.3877 | 0.0173 | 0.0029 | 0.1569 | 0.0038 | 0.6184 | 0.8835 | 0.2743 | 0.3155 |
| **Gulf of Guinea Islands** | Gulf of Guinea | 3.7061 | 0.0001 | 0.0036 | 0.0030 | 0.0073 | 0.0008 | 0.0003 | 0.0005 | 0.0000 | 0 | 1.0376 | 0.1593 | 0 | 0 | 0.0741 | 0.0062 | 0.0031 | 1.6380 | 0.0918 | 0.6808 |
| **Southern Java** | Java Transitional | 3.7036 | 0.0061 | 0.0778 | 0.0641 | 0.0448 | 0.0091 | 0.0162 | 0.0098 | 0.0006 | 0.0113 | 1.0760 | 0.0958 | 0.0005 | 0.0021 | 0.0012 | 0.0079 | 0.0597 | 1.5138 | 0.0604 | 0.6501 |
| **Northern Gulf of Mexico** | Warm Temperate Northwest Atlantic | 3.6964 | 0.0003 | 0.0646 | 0.0263 | 0.0611 | 0.0133 | 0.0191 | 0.0340 | 0.0032 | 0.0248 | 0.8266 | 0.3038 | 0.0004 | 0.0054 | 0.0055 | 0.0004 | 0.2869 | 1.3707 | 0.1937 | 0.4654 |
| **Bermuda** | Tropical Northwestern Atlantic | 3.6902 | 0.0002 | 0.0002 | 0.0003 | 0.0008 | 0.0005 | 0.0001 | 0.0025 | 0.0002 | 0 | 1.2046 | 0.3410 | 0 | 0 | 0.0001 | 0.0004 | 0.0019 | 1.1157 | 0.1996 | 0.8221 |
| **Southeast Papua New Guinea** | Eastern Coral Triangle | 3.6778 | 0.0015 | 0.0039 | 0.0033 | 0.0626 | 0.0055 | 0.0024 | 0.0019 | 0.0001 | 0.0008 | 1.1183 | 0.1879 | 0 | 0.0000 | 0.0005 | 0.0919 | 0.0291 | 1.5062 | 0.1109 | 0.5544 |
| **Solomon Archipelago** | Eastern Coral Triangle | 3.6715 | 0.0018 | 0.0064 | 0.0066 | 0.0445 | 0.0065 | 0.0009 | 0.0011 | 0.0000 | 0.0001 | 1.1062 | 0.0787 | 0 | 0.0000 | 0.0004 | 0.0638 | 0.0220 | 1.7040 | 0.0463 | 0.5852 |
| **Bight of Sofala/Swamp Coast** | Western Indian Ocean | 3.6683 | 0.0003 | 0.0438 | 0.0036 | 0.0031 | 0.0083 | 0.0149 | 0.0079 | 0.0001 | 0.0062 | 0.9846 | 0.1368 | 0 | 0.0013 | 0 | 0.0020 | 0.1893 | 1.6561 | 0.0770 | 0.5408 |
| **Carolinian** | Warm Temperate Northwest Atlantic | 3.6555 | 0.0004 | 0.0644 | 0.0168 | 0.0215 | 0.0068 | 0.0149 | 0.0244 | 0.0016 | 0.0137 | 0.9938 | 0.3985 | 0 | 0.0017 | 0.0025 | 0.0015 | 0.1531 | 1.1729 | 0.2695 | 0.5012 |
| **Rio Grande** | Warm Temperate Southwestern Atlantic | 3.6513 | 0.0003 | 0.0605 | 0.0281 | 0.0120 | 0.0084 | 0.0046 | 0.0122 | 0.0012 | 0.0124 | 0.9435 | 0.1532 | 0 | 0.0021 | 0.0066 | 0.0063 | 0.1396 | 1.5171 | 0.1399 | 0.6097 |
| **East African Coral Coast** | Western Indian Ocean | 3.6487 | 0.0021 | 0.0495 | 0.0448 | 0.0124 | 0.0147 | 0.0163 | 0.0097 | 0.0006 | 0.0064 | 1.0407 | 0.0996 | 0 | 0.0007 | 0.0074 | 0.0029 | 0.0668 | 1.7014 | 0.0548 | 0.5275 |
| **Bismarck Sea** | Eastern Coral Triangle | 3.6461 | 0.0024 | 0.0122 | 0.0085 | 0.0536 | 0.0104 | 0.0034 | 0.0020 | 0.0001 | 0.0003 | 1.0859 | 0.1318 | 0 | 0.0001 | 0 | 0.0785 | 0.0342 | 1.6127 | 0.0848 | 0.5302 |
| **Tristan Gough** | Tristan Gough | 3.6456 | 0.0000 | 0.0001 | 0.0007 | 0.0009 | 0.0000 | 0.0000 | 0.0000 | 0 | 0 | 0.9739 | 0.0273 | 0 | 0 | 0.0000 | 0.0004 | 0.0026 | 1.8703 | 0.0157 | 0.7538 |
| **Chagos** | Central Indian Ocean Islands | 3.6412 | 0 | 0.0007 | 0.0007 | 0.0038 | 0 | 0 | 0.0003 | 0.0001 | 0 | 1.1069 | 0.1126 | 0 | 0 | 0.0000 | 0.0055 | 0.0347 | 1.6681 | 0.0626 | 0.6452 |
| **Namaqua** | Benguela | 3.6383 | 0.0001 | 0.0189 | 0.0086 | 0.1103 | 0.0032 | 0.0037 | 0.0035 | 0.0004 | 0.0025 | 1.0585 | 0.2101 | 0 | 0.0009 | 0 | 0.0003 | 0.0216 | 1.3596 | 0.1593 | 0.6797 |
| **St. Helena and Ascension Islands** | St. Helena and Ascension Islands | 3.6297 | 0.0000 | 0.0002 | 0.0000 | 0.0008 | 0.0001 | 0.0000 | 0.0002 | 0.0000 | 0 | 1.0873 | 0.0485 | 0 | 0 | 0.0000 | 0.0008 | 0.0005 | 1.7072 | 0.0261 | 0.7581 |
| **Great Australian Bight** | Southwest Australian Shelf | 3.6254 | 0.0000 | 0.0306 | 0.0355 | 0.0060 | 0.0006 | 0.0000 | 0.0032 | 0.0000 | 0.0008 | 0.8755 | 0.0847 | 0 | 0.0000 | 0.0009 | 0.0008 | 0.2102 | 1.7814 | 0.0587 | 0.5410 |
| **Virginian** | Cold Temperate Northwest Atlantic | 3.6241 | 0.0016 | 0.1744 | 0.0573 | 0.0479 | 0.0410 | 0.0220 | 0.0638 | 0.0097 | 0.0179 | 0.7531 | 0.3804 | 0 | 0.0040 | 0 | 0.0064 | 0.2579 | 0.9440 | 0.2834 | 0.5720 |
| **Three Kings-North Cape** | Northern New Zealand | 3.6221 | 0.0000 | 0.0231 | 0.0299 | 0.0032 | 0.0003 | 0.0000 | 0 | 0 | 0.0000 | 1.2038 | 0.2065 | 0 | 0.0000 | 0.0003 | 0.0007 | 0.0037 | 1.3553 | 0.1341 | 0.6616 |
| **Banda Sea** | Western Coral Triangle | 3.6146 | 0.0029 | 0.1701 | 0.1159 | 0.1088 | 0.0197 | 0.0078 | 0.0042 | 0.0002 | 0.0069 | 1.0812 | 0.0860 | 0.0000 | 0.0004 | 0.0033 | 0.0524 | 0.0957 | 1.2562 | 0.0518 | 0.5582 |
| **Namib** | Benguela | 3.6051 | 0.0001 | 0.0269 | 0.0172 | 0.0685 | 0.0009 | 0.0026 | 0.0014 | 0.0001 | 0.0003 | 1.0002 | 0.1521 | 0 | 0.0000 | 0 | 0.0002 | 0.0216 | 1.6098 | 0.1151 | 0.5921 |
| **Chiapas-Nicaragua** | Tropical East Pacific | 3.5962 | 0.0013 | 0.0609 | 0.0296 | 0.0203 | 0.0072 | 0.0135 | 0.0185 | 0.0006 | 0.0122 | 0.9478 | 0.2427 | 0 | 0.0044 | 0.0213 | 0.0153 | 0.0584 | 1.3310 | 0.1492 | 0.6680 |
| **Seychelles** | Western Indian Ocean | 3.5961 | 0.0001 | 0.0025 | 0.0009 | 0.0087 | 0.0004 | 0.0000 | 0.0007 | 0.0001 | 0.0000 | 1.0972 | 0.0808 | 0 | 0.0000 | 0.0075 | 0.0120 | 0.0195 | 1.6963 | 0.0479 | 0.6217 |
| **Floridian** | Tropical Northwestern Atlantic | 3.5844 | 0.0041 | 0.1073 | 0.0539 | 0.0490 | 0.0257 | 0.0262 | 0.0376 | 0.0083 | 0.0169 | 0.7689 | 0.3281 | 0 | 0.0020 | 0.0071 | 0.0005 | 0.4091 | 1.0068 | 0.2957 | 0.4432 |
| **Kermadec Island** | Northern New Zealand | 3.5747 | 0.0000 | 0.0126 | 0.0048 | 0.0025 | 0 | 0 | 0 | 0 | 0 | 1.1805 | 0.0825 | 0 | 0 | 0 | 0.0004 | 0.0003 | 1.4964 | 0.0462 | 0.7515 |
| **Clipperton** | Tropical East Pacific | 3.5656 | 0 | 0.0003 | 0.0022 | 0.0014 | 0 | 0 | 0 | 0 | 0 | 1.0736 | 0.1426 | 0 | 0 | 0.0080 | 0.0021 | 0.0000 | 1.4841 | 0.0787 | 0.7727 |
| **Gulf of Guinea South** | Gulf of Guinea | 3.5358 | 0.0004 | 0.0474 | 0.0559 | 0.0193 | 0.0051 | 0.0116 | 0.0213 | 0.0009 | 0.0025 | 0.9799 | 0.1655 | 0.0190 | 0.0006 | 0.0614 | 0.0024 | 0.0763 | 1.4414 | 0.1174 | 0.5119 |
| **Coral Sea** | Tropical Southwestern Pacific | 3.5262 | 0 | 0.0030 | 0.0009 | 0.0320 | 0 | 0 | 0 | 0 | 0 | 1.1443 | 0.1840 | 0 | 0 | 0.0023 | 0.0430 | 0.0120 | 1.4265 | 0.1219 | 0.5562 |
| **Central Somali Coast** | Somali/Arabian | 3.5249 | 0.0001 | 0.0126 | 0.0047 | 0.0039 | 0.0039 | 0.0046 | 0 | 0 | 0 | 1.0330 | 0.0929 | 0 | 0 | 0.0007 | 0.0034 | 0.0331 | 1.6660 | 0.0518 | 0.6182 |
| **Mexican Tropical Pacific** | Tropical East Pacific | 3.5147 | 0.0007 | 0.0351 | 0.0116 | 0.0089 | 0.0028 | 0.0067 | 0.0018 | 0.0003 | 0.0034 | 1.0814 | 0.3121 | 0 | 0.0017 | 0.0204 | 0.0067 | 0.0060 | 1.1401 | 0.1946 | 0.6822 |
| **Central Chile** | Warm Temperate Southeastern Pacific | 3.5138 | 0.0011 | 0.0156 | 0.0425 | 0.1518 | 0.0042 | 0.0070 | 0.0032 | 0.0004 | 0.0021 | 1.0336 | 0.2441 | 0 | 0.0005 | 0 | 0.0004 | 0.0057 | 1.0520 | 0.1431 | 0.8077 |
| **West Greenland Shelf** | Arctic | 3.5094 | 0.0016 | 0.1872 | 0.0341 | 0.0040 | 0.0051 | 0 | 0.0167 | 0.0002 | 0 | 0.7346 | 0.1136 | 0 | 0 | 0 | 0.0000 | 0.0991 | 1.6887 | 0.0821 | 0.5811 |
| **New Caledonia** | Tropical Southwestern Pacific | 3.5061 | 0.0015 | 0.0029 | 0.0020 | 0.0021 | 0.0029 | 0.0001 | 0.0017 | 0.0001 | 0.0001 | 1.2122 | 0.1405 | 0 | 0.0000 | 0 | 0.0021 | 0.0390 | 1.4183 | 0.0907 | 0.5913 |
| **Hawaii** | Hawaii | 3.5038 | 0.0002 | 0.0014 | 0.0013 | 0.0041 | 0.0006 | 0.0005 | 0.0006 | 0.0001 | 0.0005 | 1.1776 | 0.1842 | 0 | 0.0001 | 0.0017 | 0.0024 | 0.0023 | 1.3065 | 0.1078 | 0.7127 |
| **Araucanian** | Warm Temperate Southeastern Pacific | 3.4981 | 0.0021 | 0.0243 | 0.0312 | 0.1174 | 0.0056 | 0.0102 | 0.0051 | 0.0005 | 0.0097 | 0.9864 | 0.1676 | 0 | 0.0048 | 0 | 0.0001 | 0.0157 | 1.2508 | 0.1125 | 0.7556 |
| **Aleutian Islands** | Cold Temperate Northeast Pacific | 3.4838 | 0.0000 | 0.0253 | 0.0040 | 0.0103 | 0.0004 | 0.0003 | 0.0015 | 0.0000 | 0.0004 | 0.6968 | 0.2832 | 0 | 0.0000 | 0 | 0.0003 | 0.0084 | 1.5257 | 0.1722 | 0.7598 |
| **Northern Monsoon Current Coast** | Western Indian Ocean | 3.4832 | 0.0005 | 0.0108 | 0.0059 | 0.0039 | 0.0057 | 0.0054 | 0.0057 | 0.0000 | 0.0032 | 1.0599 | 0.0357 | 0 | 0.0005 | 0.0071 | 0.0036 | 0.0287 | 1.7195 | 0.0169 | 0.5769 |
| **Kamchatka Shelf and Coast** | Cold Temperate Northwest Pacific | 3.4748 | 0.0001 | 0.0691 | 0.0167 | 0.0595 | 0.0023 | 0.0006 | 0.0005 | 0.0001 | 0.0002 | 0.6715 | 0.1707 | 0 | 0.0000 | 0 | 0.0000 | 0.0226 | 1.6673 | 0.1039 | 0.6976 |
| **Northeast Sulawesi** | Western Coral Triangle | 3.4702 | 0.0037 | 0.3186 | 0.2559 | 0.1979 | 0.0368 | 0.0251 | 0.0166 | 0.0006 | 0.0350 | 0.8980 | 0.0127 | 0 | 0.0022 | 0.0034 | 0.0374 | 0.3326 | 1.1043 | 0.0029 | 0.1993 |
| **Gilbert/Ellis Islands** | Marshall, Gilbert and Ellis Islands | 3.4374 | 0.0003 | 0.0067 | 0.0070 | 0.0124 | 0.0008 | 0 | 0.0002 | 0.0000 | 0 | 1.1171 | 0.0296 | 0 | 0 | 0.0000 | 0.0131 | 0.0042 | 1.5574 | 0.0162 | 0.6728 |
| **Easter Island** | Easter Island | 3.4348 | 0.0000 | 0.0248 | 0.0141 | 0.0139 | 0.0001 | 0 | 0 | 0.0000 | 0.0001 | 1.0681 | 0.0210 | 0 | 0.0000 | 0 | 0.0138 | 0.0002 | 1.5479 | 0.0121 | 0.7189 |
| **Juan Fernandez and Desventuradas** | Juan Fernandez and Desventuradas | 3.3700 | 0 | 0.0056 | 0.0351 | 0.0620 | 0 | 0 | 0 | 0.0000 | 0 | 1.0456 | 0.0723 | 0 | 0 | 0 | 0.0009 | 0.0003 | 1.2961 | 0.0397 | 0.8123 |
| **East Caroline Islands** | Tropical Northwestern Pacific | 3.3648 | 0.0002 | 0.0021 | 0.0060 | 0.0109 | 0.0007 | 0.0000 | 0.0004 | 0.0000 | 0 | 1.1373 | 0.1068 | 0 | 0 | 0 | 0.0143 | 0.0083 | 1.3502 | 0.0615 | 0.6662 |
| **Western Sumatra** | Andaman | 3.3325 | 0.0033 | 0.1100 | 0.0978 | 0.0639 | 0.0105 | 0.0084 | 0.0032 | 0.0001 | 0.0070 | 1.0596 | 0.0470 | 0 | 0.0007 | 0.0025 | 0.0077 | 0.0494 | 1.2235 | 0.0287 | 0.6118 |
| **Marshall Islands** | Marshall, Gilbert and Ellis Islands | 3.3162 | 0.0005 | 0.0008 | 0.0026 | 0.0055 | 0.0013 | 0 | 0.0002 | 0.0000 | 0 | 1.1843 | 0.0807 | 0 | 0 | 0 | 0.0081 | 0.0089 | 1.3958 | 0.0502 | 0.5771 |
| **Cortezian** | Warm Temperate Northeast Pacific | 3.3018 | 0.0013 | 0.0822 | 0.0130 | 0.1258 | 0.0198 | 0.0203 | 0.0111 | 0.0017 | 0.0180 | 0.8908 | 0.1072 | 0 | 0.0084 | 0.0033 | 0.0045 | 0.1220 | 1.4046 | 0.0712 | 0.4138 |
| **South Georgia** | Scotia Sea | 3.2759 | 0 | 0.0015 | 0.0000 | 0.0008 | 0 | 0 | 0.0009 | 0 | 0 | 0.7838 | 0.0197 | 0 | 0 | 0 | 0.0003 | 0.0136 | 1.6664 | 0.0114 | 0.7786 |
| **Panama Bight** | Tropical East Pacific | 3.2739 | 0.0025 | 0.0499 | 0.0064 | 0.0563 | 0.0075 | 0.0063 | 0.0086 | 0.0004 | 0.0047 | 0.8800 | 0.1815 | 0 | 0.0020 | 0.0005 | 0.0041 | 0.0299 | 1.2609 | 0.1171 | 0.6588 |
| **Phoenix/Tokelau/Northern Cook Islands** | Central Polynesia | 3.2567 | 0.0000 | 0.0005 | 0.0011 | 0.0042 | 0.0001 | 0 | 0.0000 | 0 | 0 | 1.1102 | 0.0445 | 0 | 0 | 0.0000 | 0.0055 | 0.0005 | 1.3901 | 0.0241 | 0.6757 |
| **Bassian** | Southeast Australian Shelf | 3.2533 | 0.0010 | 0.0151 | 0.0084 | 0.0025 | 0.0096 | 0.0026 | 0.0159 | 0.0009 | 0.0042 | 0.9616 | 0.0845 | 0.0000 | 0.0004 | 0.0001 | 0.0004 | 0.0699 | 1.2964 | 0.0715 | 0.7142 |
| **Oregon, Washington, Vancouver Coast and Shelf** | Cold Temperate Northeast Pacific | 3.2504 | 0.0005 | 0.0359 | 0.0066 | 0.0568 | 0.0091 | 0.0052 | 0.0138 | 0.0012 | 0.0047 | 0.9288 | 0.3961 | 0 | 0.0015 | 0 | 0.0014 | 0.0253 | 0.7530 | 0.3037 | 0.7111 |
| **Oyashio Current** | Cold Temperate Northwest Pacific | 3.2409 | 0.0001 | 0.1120 | 0.0349 | 0.0534 | 0.0027 | 0.0009 | 0.0012 | 0.0002 | 0.0014 | 0.7751 | 0.2992 | 0 | 0.0005 | 0 | 0.0046 | 0.0159 | 1.0187 | 0.1916 | 0.7304 |
| **Magdalena Transition** | Warm Temperate Northeast Pacific | 3.2312 | 0.0002 | 0.0303 | 0.0110 | 0.1028 | 0.0048 | 0.0036 | 0 | 0.0001 | 0.0020 | 0.9448 | 0.3118 | 0 | 0.0007 | 0.0092 | 0.0042 | 0.0286 | 0.9563 | 0.2112 | 0.6133 |
| **Chiloense** | Magellanic | 3.2259 | 0.0016 | 0.1131 | 0.0398 | 0.0951 | 0.0242 | 0.0039 | 0.0025 | 0.0004 | 0.0031 | 0.8581 | 0.0553 | 0 | 0.0004 | 0 | 0.0000 | 0.0935 | 1.2799 | 0.0369 | 0.6417 |
| **Gulf of Alaska** | Cold Temperate Northeast Pacific | 3.2104 | 0.0001 | 0.0787 | 0.0110 | 0.0202 | 0.0028 | 0.0033 | 0.0133 | 0.0005 | 0.0018 | 0.7409 | 0.2683 | 0.0000 | 0.0001 | 0 | 0.0004 | 0.0440 | 1.0900 | 0.2011 | 0.7467 |
| **Southern Red Sea** | Red Sea and Gulf of Aden | 3.2024 | 0.0020 | 0.0578 | 0.0267 | 0.0180 | 0.0252 | 0.0174 | 0.0270 | 0.0016 | 0.0079 | 0.7814 | 0.2343 | 0 | 0.0003 | 0.0000 | 0.0007 | 0.4477 | 1.1314 | 0.1862 | 0.2500 |
| **Sea of Okhotsk** | Cold Temperate Northwest Pacific | 3.1985 | 0.0003 | 0.5173 | 0.0998 | 0.1123 | 0.0054 | 0.0016 | 0.0033 | 0.0005 | 0.0014 | 0.5810 | 0.1055 | 0.0000 | 0.0003 | 0 | 0.0003 | 0.1029 | 1.0267 | 0.0886 | 0.5583 |
| **Central and Southern Great Barrier Reef** | Northeast Australian Shelf | 3.1880 | 0.0028 | 0.0390 | 0.0188 | 0.0202 | 0.0156 | 0.0070 | 0.0379 | 0.0015 | 0.0137 | 0.6047 | 0.2564 | 0 | 0.0008 | 0.0016 | 0.0216 | 0.8736 | 0.8904 | 0.1704 | 0.2196 |
| **Northern California** | Cold Temperate Northeast Pacific | 3.1867 | 0.0005 | 0.0805 | 0.0592 | 0.0262 | 0.0057 | 0.0044 | 0.0097 | 0.0012 | 0.0033 | 0.9246 | 0.4726 | 0 | 0.0011 | 0 | 0.0005 | 0.0128 | 0.5629 | 0.3258 | 0.6974 |
| **Northern Labrador** | Arctic | 3.0957 | 0.0000 | 0.1745 | 0.0132 | 0.0137 | 0.0009 | 0.0000 | 0.0037 | 0.0002 | 0.0003 | 0.7113 | 0.0821 | 0 | 0.0000 | 0 | 0.0101 | 0.1305 | 1.3939 | 0.0690 | 0.5206 |
| **Patagonian Shelf** | Magellanic | 3.0812 | 0.0000 | 0.2876 | 0.0653 | 0.0237 | 0.0026 | 0.0005 | 0.0046 | 0.0002 | 0.0006 | 0.8170 | 0.0390 | 0.0000 | 0.0001 | 0.0000 | 0.0001 | 0.0714 | 0.9243 | 0.0477 | 0.7992 |
| **Western Galapagos Islands** | Galapagos | 3.0760 | 0.0000 | 0.0036 | 0.0076 | 0.0120 | 0.0003 | 0.0001 | 0 | 0.0000 | 0.0003 | 0.8946 | 0.1136 | 0 | 0.0001 | 0.0001 | 0.0095 | 0.0023 | 1.2709 | 0.0633 | 0.6988 |
| **Trindade and Martin Vaz Islands** | Tropical Southwestern Atlantic | 3.0557 | 0 | 0.0111 | 0.0109 | 0.0082 | 0 | 0 | 0 | 0 | 0 | 1.1600 | 0.0548 | 0 | 0 | 0.0193 | 0.0117 | 0.0001 | 0.9256 | 0.0292 | 0.8248 |
| **Nicoya** | Tropical East Pacific | 3.0440 | 0.0029 | 0.0675 | 0.0181 | 0.0435 | 0.0083 | 0.0068 | 0.0104 | 0.0007 | 0.0054 | 0.9379 | 0.2553 | 0 | 0.0028 | 0.0328 | 0.0040 | 0.0204 | 0.8074 | 0.1718 | 0.6519 |
| **Malvinas/Falklands** | Magellanic | 3.0428 | 0.0001 | 0.0923 | 0.0031 | 0.0034 | 0.0017 | 0 | 0.0013 | 0.0000 | 0 | 0.8609 | 0.0369 | 0 | 0 | 0.0000 | 0.0000 | 0.0598 | 1.3382 | 0.0316 | 0.6164 |
| **Uruguay-Buenos Aires Shelf** | Warm Temperate Southwestern Atlantic | 3.0357 | 0.0001 | 0.1817 | 0.0392 | 0.0290 | 0.0040 | 0.0029 | 0.0131 | 0.0005 | 0.0053 | 0.6460 | 0.1043 | 0 | 0.0009 | 0.0001 | 0.0007 | 0.2992 | 1.0714 | 0.0868 | 0.5532 |
| **Revillagigedos** | Tropical East Pacific | 3.0284 | 0 | 0.0068 | 0.0126 | 0.0046 | 0.0001 | 0 | 0 | 0.0000 | 0.0000 | 1.1162 | 0.1967 | 0 | 0.0000 | 0.0236 | 0.0072 | 0.0002 | 0.9750 | 0.1242 | 0.5613 |
| **Bonaparte Coast** | Sahul Shelf | 3.0203 | 0.0001 | 0.1333 | 0.0580 | 0.0330 | 0.0038 | 0.0012 | 0.0057 | 0.0002 | 0.0088 | 0.6829 | 0.1077 | 0.0002 | 0.0004 | 0.0060 | 0.0095 | 0.5119 | 0.8011 | 0.0991 | 0.5653 |
| **Rapa-Pitcairn** | Southeast Polynesia | 3.0042 | 0.0000 | 0.0000 | 0.0002 | 0.0004 | 0.0000 | 0 | 0 | 0.0000 | 0 | 1.2176 | 0.0724 | 0 | 0 | 0 | 0.0006 | 0.0002 | 0.9684 | 0.0420 | 0.7024 |
| **Southern California Bight** | Warm Temperate Northeast Pacific | 2.9919 | 0.0007 | 0.0284 | 0.0123 | 0.0728 | 0.0044 | 0.0070 | 0.0069 | 0.0008 | 0.0011 | 0.9611 | 0.3539 | 0 | 0.0002 | 0.0008 | 0.0040 | 0.0161 | 0.6544 | 0.2409 | 0.6284 |
| **East Greenland Shelf** | Arctic | 2.9700 | 0.0002 | 0.0697 | 0.0347 | 0.0574 | 0.0008 | 0 | 0.0006 | 0.0000 | 0 | 0.8339 | 0.0968 | 0 | 0 | 0 | 0.0020 | 0.0613 | 1.3988 | 0.0649 | 0.4496 |
| **North Patagonian Gulfs** | Magellanic | 2.9592 | 0.0001 | 0.2426 | 0.0457 | 0.0242 | 0.0058 | 0.0010 | 0.0082 | 0.0004 | 0.0010 | 0.7603 | 0.0451 | 0 | 0.0001 | 0.0000 | 0.0001 | 0.1939 | 0.8693 | 0.0512 | 0.7157 |
| **Arafura Sea** | Sahul Shelf | 2.9422 | 0.0002 | 0.3229 | 0.2189 | 0.1250 | 0.0103 | 0.0060 | 0 | 0.0000 | 0.0075 | 0.5201 | 0.0547 | 0 | 0.0003 | 0.0009 | 0.0342 | 0.6676 | 0.5098 | 0.0430 | 0.4238 |
| **Tuamotus** | Southeast Polynesia | 2.9402 | 0.0002 | 0.0007 | 0.0002 | 0.0006 | 0.0016 | 0 | 0.0001 | 0.0000 | 0 | 1.2185 | 0.0537 | 0 | 0 | 0 | 0.0008 | 0.0070 | 1.0275 | 0.0328 | 0.5964 |
| **Eastern Galapagos Islands** | Galapagos | 2.9322 | 0.0001 | 0.0195 | 0.0112 | 0.0249 | 0.0010 | 0.0002 | 0 | 0.0000 | 0.0008 | 0.8717 | 0.1189 | 0 | 0.0002 | 0.0023 | 0.0115 | 0.0045 | 1.1838 | 0.0741 | 0.6088 |
| **Northeastern New Zealand** | Northern New Zealand | 2.9160 | 0.0004 | 0.0200 | 0.0126 | 0.0051 | 0.0091 | 0.0029 | 0.0080 | 0.0007 | 0.0054 | 1.1017 | 0.1460 | 0 | 0.0004 | 0.0002 | 0.0005 | 0.0466 | 0.7990 | 0.0989 | 0.6621 |
| **North and East Barents Sea** | Arctic | 2.9134 | 0.0000 | 0.1576 | 0.1602 | 0.0414 | 0.0007 | 0.0003 | 0.0003 | 0.0000 | 0.0001 | 0.8394 | 0.0749 | 0 | 0.0000 | 0 | 0.0148 | 0.0549 | 1.2393 | 0.0719 | 0.4415 |
| **Channels and Fjords of Southern Chile** | Magellanic | 2.8932 | 0.0003 | 0.0723 | 0.0081 | 0.0057 | 0.0068 | 0.0014 | 0.0048 | 0.0001 | 0.0031 | 0.7770 | 0.0507 | 0.0000 | 0.0003 | 0 | 0.0000 | 0.0999 | 1.1725 | 0.0328 | 0.6802 |
| **Humboldtian** | Warm Temperate Southeastern Pacific | 2.8792 | 0.0004 | 0.0415 | 0.0320 | 0.1283 | 0.0026 | 0.0056 | 0.0023 | 0.0004 | 0.0018 | 1.0384 | 0.1852 | 0 | 0.0005 | 0.0021 | 0.0031 | 0.0020 | 0.5984 | 0.1061 | 0.7290 |
| **White Sea** | Arctic | 2.8724 | 0.0019 | 0.0569 | 0.1220 | 0.0719 | 0.0361 | 0.0227 | 0.0560 | 0.0026 | 0.0034 | 0.4020 | 0.0720 | 0 | 0.0002 | 0 | 0.0013 | 0.4520 | 1.1371 | 0.0491 | 0.4159 |
| **Central New Zealand** | Southern New Zealand | 2.7644 | 0.0005 | 0.0910 | 0.0180 | 0.0073 | 0.0084 | 0.0119 | 0.0083 | 0.0006 | 0.0093 | 1.0008 | 0.1223 | 0 | 0.0007 | 0.0004 | 0.0003 | 0.0537 | 0.8072 | 0.0985 | 0.5290 |
| **Guayaquil** | Tropical East Pacific | 2.7446 | 0.0005 | 0.0701 | 0.0191 | 0.0829 | 0.0080 | 0.0069 | 0.0116 | 0.0010 | 0.0075 | 0.8050 | 0.1428 | 0 | 0.0034 | 0.0031 | 0.0051 | 0.0191 | 0.8699 | 0.0914 | 0.5992 |
| **Eastern Bering Sea** | Arctic | 2.7402 | 0.0001 | 0.4273 | 0.0719 | 0.0450 | 0.0026 | 0.0018 | 0.0064 | 0.0002 | 0.0015 | 0.4425 | 0.1072 | 0 | 0.0002 | 0 | 0.0019 | 0.1800 | 0.7357 | 0.0963 | 0.6243 |
| **Amsterdam-St Paul** | Amsterdam-St Paul | 2.7200 | 0 | 0.0002 | 0.0001 | 0.0001 | 0 | 0 | 0 | 0 | 0 | 1.1392 | 0.0280 | 0 | 0 | 0 | 0.0002 | 0.0002 | 0.8721 | 0.0175 | 0.6625 |
| **Torres Strait Northern Great Barrier Reef** | Northeast Australian Shelf | 2.6774 | 0.0002 | 0.0294 | 0.0169 | 0.0224 | 0.0035 | 0.0006 | 0.0064 | 0.0001 | 0.0067 | 0.6550 | 0.1606 | 0 | 0.0002 | 0.0019 | 0.0191 | 0.7822 | 0.6037 | 0.0698 | 0.3013 |
| **Heard and Macdonald Islands** | Subantarctic Islands | 2.6710 | 0 | 0.0264 | 0 | 0.0000 | 0 | 0 | 0 | 0 | 0 | 0.7110 | 0.0091 | 0 | 0 | 0 | 0.0034 | 0.0061 | 1.3099 | 0.0059 | 0.5998 |
| **North American Pacific Fijordland** | Cold Temperate Northeast Pacific | 2.6620 | 0.0010 | 0.0530 | 0.0099 | 0.0191 | 0.0093 | 0.0010 | 0.0359 | 0.0006 | 0.0020 | 0.7888 | 0.2526 | 0 | 0.0001 | 0 | 0.0014 | 0.0629 | 0.6534 | 0.1861 | 0.6082 |
| **Central Peru** | Warm Temperate Southeastern Pacific | 2.6248 | 0.0003 | 0.0628 | 0.0178 | 0.1350 | 0.0041 | 0.0072 | 0.0053 | 0.0005 | 0.0042 | 0.8838 | 0.1630 | 0 | 0.0010 | 0.0031 | 0.0036 | 0.0249 | 0.5391 | 0.1100 | 0.6592 |
| **Gulf of Papua** | Sahul Shelf | 2.5198 | 0.0010 | 0.0266 | 0.0117 | 0.0331 | 0.0285 | 0.0181 | 0.0158 | 0.0000 | 0.0055 | 0.7179 | 0.0775 | 0 | 0.0004 | 0.0010 | 0.0326 | 0.5302 | 0.6067 | 0.0582 | 0.3668 |
| **Chatham Island** | Southern New Zealand | 2.5115 | 0.0000 | 0.0129 | 0.0024 | 0.0021 | 0.0002 | 0.0003 | 0 | 0 | 0.0001 | 1.0354 | 0.0393 | 0 | 0.0000 | 0.0000 | 0.0002 | 0.0101 | 0.7040 | 0.0250 | 0.6802 |
| **Northern Galapagos Islands** | Galapagos | 2.5079 | 0.0000 | 0.0109 | 0.0070 | 0.0175 | 0 | 0.0000 | 0 | 0 | 0.0000 | 0.9870 | 0.1139 | 0 | 0.0000 | 0.0038 | 0.0171 | 0.0001 | 0.6977 | 0.0741 | 0.5788 |
| **South Sandwich Islands** | Scotia Sea | 2.5037 | 0 | 0.0000 | 0 | 0.0000 | 0 | 0 | 0 | 0.0000 | 0 | 0.7116 | 0.0299 | 0 | 0 | 0 | 0 | 0.0014 | 0.9391 | 0.0165 | 0.8054 |
| **East Antarctic Enderby Land** | Continental High Antarctic | 2.5019 | 0 | 0.0001 | 0 | 0 | 0 | 0 | 0 | 0 | 0 | 0.6403 | 0.0307 | 0 | 0 | 0 | 0.0000 | 0.0240 | 1.2577 | 0.0168 | 0.6368 |
| **Arnhem Coast to Gulf of Carpenteria** | Sahul Shelf | 2.4428 | 0.0000 | 0.0648 | 0.0328 | 0.0244 | 0.0042 | 0.0015 | 0.0096 | 0.0001 | 0.0082 | 0.4872 | 0.0895 | 0 | 0.0003 | 0.0014 | 0.0170 | 0.7189 | 0.4444 | 0.0735 | 0.4686 |
| **Hudson Complex** | Arctic | 2.3444 | 0.0001 | 0.0005 | 0.0000 | 0.0000 | 0.0015 | 0.0013 | 0.0029 | 0.0002 | 0.0025 | 0.3226 | 0.0331 | 0 | 0.0006 | 0 | 0.0000 | 0.2798 | 1.0988 | 0.0295 | 0.5864 |
| **South Shetland Islands** | Scotia Sea | 2.3177 | 0 | 0.0011 | 0.0002 | 0.0294 | 0 | 0 | 0.0004 | 0 | 0 | 0.6850 | 0.0422 | 0 | 0 | 0 | 0 | 0.0142 | 0.8234 | 0.0253 | 0.7111 |
| **Cocos Islands** | Tropical East Pacific | 2.2688 | 0 | 0.0095 | 0.0075 | 0.0061 | 0 | 0 | 0 | 0 | 0 | 0.9278 | 0.1348 | 0 | 0 | 0.0394 | 0.0054 | 0.0001 | 0.3788 | 0.0814 | 0.6781 |
| **South Orkney Islands** | Scotia Sea | 2.2423 | 0 | 0.0000 | 0 | 0.0867 | 0 | 0 | 0.0017 | 0 | 0 | 0.7145 | 0.0287 | 0 | 0 | 0 | 0 | 0.0080 | 0.6844 | 0.0167 | 0.7068 |
| **Rio de la Plata** | Warm Temperate Southwestern Atlantic | 2.1458 | 0.0011 | 0.1441 | 0.0238 | 0.0076 | 0.0522 | 0.0719 | 0.1346 | 0.0071 | 0.0953 | 0.0174 | 0.1905 | 0 | 0.0480 | 0.0000 | 0.0000 | 0.8398 | 0.5084 | 0.0143 | 0.0016 |
| **Baffin Bay - Davis Strait** | Arctic | 2.1313 | 0.0000 | 0.0707 | 0.0041 | 0.0020 | 0.0007 | 0.0000 | 0.0013 | 0.0001 | 0.0000 | 0.6260 | 0.0565 | 0 | 0.0000 | 0 | 0.0001 | 0.0244 | 0.8716 | 0.0406 | 0.4504 |
| **Auckland Island** | Subantarctic New Zealand | 2.1203 | 0.0000 | 0.0778 | 0.0010 | 0.0021 | 0 | 0.0001 | 0 | 0 | 0.0001 | 0.8924 | 0.0179 | 0 | 0.0000 | 0 | 0.0001 | 0.0077 | 0.4170 | 0.0124 | 0.6918 |
| **South New Zealand** | Southern New Zealand | 2.1166 | 0.0002 | 0.0896 | 0.0081 | 0.0030 | 0.0043 | 0.0087 | 0.0032 | 0.0002 | 0.0026 | 0.9341 | 0.0857 | 0 | 0.0001 | 0.0002 | 0.0002 | 0.0371 | 0.2668 | 0.0552 | 0.6184 |
| **Prince Edward Islands** | Subantarctic Islands | 2.1059 | 0 | 0.0010 | 0.0000 | 0.0000 | 0 | 0 | 0 | 0 | 0.0004 | 0.8793 | 0.0223 | 0 | 0.0001 | 0 | 0.0000 | 0.0006 | 0.6003 | 0.0158 | 0.5862 |
| **Snares Island** | Southern New Zealand | 2.0939 | 0 | 0.1335 | 0.0058 | 0.0037 | 0 | 0 | 0 | 0 | 0 | 0.8971 | 0.0456 | 0 | 0 | 0.0001 | 0.0002 | 0.0432 | 0.2744 | 0.0322 | 0.6580 |
| **East Antarctic Dronning Maud Land** | Continental High Antarctic | 2.0708 | 0 | 0.0003 | 0 | 0 | 0 | 0 | 0 | 0 | 0 | 0.5868 | 0.0201 | 0 | 0 | 0 | 0.0000 | 0.0657 | 1.1205 | 0.0109 | 0.5097 |
| **Chukchi Sea** | Arctic | 2.0662 | 0.0000 | 0.0000 | 0.0000 | 0.0000 | 0.0014 | 0.0002 | 0.0025 | 0.0002 | 0.0008 | 0.4396 | 0.0283 | 0 | 0.0001 | 0 | 0 | 0.2143 | 1.0299 | 0.0224 | 0.4295 |
| **Peter the First Island** | Subantarctic Islands | 2.0582 | 0 | 0 | 0 | 0 | 0 | 0 | 0 | 0 | 0 | 0.6994 | 0.0102 | 0 | 0 | 0 | 0 | 0.0009 | 0.6379 | 0.0054 | 0.7205 |
| **Line Islands** | Central Polynesia | 2.0056 | 0.0000 | 0.0003 | 0.0003 | 0.0016 | 0.0003 | 0 | 0.0001 | 0.0000 | 0.0000 | 0.9928 | 0.0826 | 0 | 0.0000 | 0.0001 | 0.0019 | 0.0004 | 0.1475 | 0.0449 | 0.7328 |
| **Kerguelen Islands** | Subantarctic Islands | 1.9430 | 0 | 0.0308 | 0.0002 | 0.0001 | 0 | 0 | 0 | 0.0000 | 0 | 0.7488 | 0.0176 | 0 | 0 | 0 | 0.0002 | 0.0267 | 0.5141 | 0.0135 | 0.5920 |
| **Campbell Island** | Subantarctic New Zealand | 1.9293 | 0.0000 | 0.1631 | 0.0005 | 0.0038 | 0 | 0.0000 | 0 | 0 | 0.0000 | 0.8512 | 0.0350 | 0 | 0.0000 | 0 | 0.0001 | 0.0008 | 0.3391 | 0.0250 | 0.5107 |
| **Crozet Islands** | Subantarctic Islands | 1.9212 | 0 | 0.0015 | 0.0001 | 0.0000 | 0 | 0 | 0 | 0 | 0 | 0.8831 | 0.0147 | 0 | 0 | 0 | 0.0001 | 0.0035 | 0.4245 | 0.0104 | 0.5836 |
| **Marquesas** | Marquesas | 1.9094 | 0.0001 | 0.0003 | 0.0001 | 0.0007 | 0.0006 | 0.0001 | 0.0002 | 0.0000 | 0.0000 | 1.1208 | 0.0630 | 0 | 0.0000 | 0 | 0.0008 | 0.0003 | 0.0362 | 0.0350 | 0.6513 |
| **East Antarctic Wilkes Land** | Continental High Antarctic | 1.8940 | 0 | 0.0070 | 0 | 0.0003 | 0 | 0 | 0 | 0 | 0 | 0.6181 | 0.0189 | 0 | 0 | 0 | 0.0074 | 0.0538 | 0.7826 | 0.0107 | 0.5429 |
| **Bouvet Island** | Subantarctic Islands | 1.8877 | 0 | 0 | 0 | 0 | 0 | 0 | 0 | 0 | 0 | 0.7607 | 0.0190 | 0 | 0 | 0 | 0 | 0.0003 | 0.5202 | 0.0134 | 0.5740 |
| **Macquarie Island** | Subantarctic Islands | 1.8841 | 0.0000 | 0.0000 | 0.0000 | 0.0000 | 0.0002 | 0.0000 | 0 | 0 | 0.0000 | 0.8101 | 0.0127 | 0 | 0.0000 | 0 | 0.0000 | 0.0007 | 0.2281 | 0.0070 | 0.8252 |
| **Bounty and Antipodes Islands** | Subantarctic New Zealand | 1.8496 | 0.0000 | 0.0069 | 0.0012 | 0.0010 | 0 | 0.0000 | 0 | 0 | 0.0000 | 0.9240 | 0.0209 | 0 | 0.0000 | 0 | 0.0002 | 0.0003 | 0.2418 | 0.0123 | 0.6418 |
| **Antarctic Peninsula** | Scotia Sea | 1.6558 | 0 | 0.0000 | 0 | 0.1135 | 0 | 0 | 0.0009 | 0 | 0 | 0.5888 | 0.0312 | 0 | 0 | 0 | 0 | 0.1016 | 0.6019 | 0.0210 | 0.3680 |
| **Kara Sea** | Arctic | 1.5313 | 0.0000 | 0.0000 | 0.0000 | 0.0000 | 0.0041 | 0.0013 | 0.0014 | 0.0001 | 0.0005 | 0.4856 | 0.0177 | 0.0000 | 0.0001 | 0 | 0 | 0.2422 | 0.6628 | 0.0117 | 0.2824 |
| **Amundsen/Bellingshausen Sea** | Continental High Antarctic | 1.4575 | 0 | 0.0001 | 0 | 0.0000 | 0 | 0 | 0 | 0 | 0 | 0.5472 | 0.0066 | 0 | 0 | 0 | 0.0001 | 0.0322 | 0.9785 | 0.0040 | 0.3074 |
| **Lancaster Sound** | Arctic | 1.2368 | 0.0001 | 0.0019 | 0.0001 | 0.0000 | 0.0007 | 0 | 0.0005 | 0.0001 | 0.0001 | 0.3082 | 0.0272 | 0 | 0.0000 | 0 | 0 | 0.0085 | 0.7547 | 0.0186 | 0.2395 |
| **Beaufort Sea - continental coast and shelf** | Arctic | 1.1812 | 0.0000 | 0.0000 | 0.0000 | 0 | 0.0004 | 0.0000 | 0.0034 | 0.0005 | 0.0020 | 0.6329 | 0.0088 | 0.0000 | 0.0001 | 0 | 0 | 0.0826 | 0.5284 | 0.0046 | 0.1336 |
| **North Greenland** | Arctic | 1.1512 | 0.0000 | 0.0461 | 0.0034 | 0.0008 | 0.0001 | 0 | 0.0006 | 0 | 0.0000 | 0.7209 | 0.0113 | 0 | 0.0000 | 0 | 0 | 0.0063 | 0.7156 | 0.0092 | 0.1510 |
| **East Siberian Sea** | Arctic | 0.9663 | 0.0000 | 0.0000 | 0.0000 | 0 | 0.0006 | 0.0005 | 0.0010 | 0.0000 | 0.0000 | 0.3055 | 0.0016 | 0 | 0.0000 | 0 | 0 | 0.0001 | 0.3459 | 0.0012 | 0.3346 |
| **Beaufort-Amundsen-Viscount Melville-Queen Maud** | Arctic | 0.8387 | 0.0001 | 0.0001 | 0.0000 | 0 | 0.0005 | 0.0000 | 0.0063 | 0.0001 | 0.0003 | 0.3706 | 0.0064 | 0 | 0.0000 | 0 | 0 | 0.0292 | 0.4284 | 0.0024 | 0.1508 |
| **Weddell Sea** | Continental High Antarctic | 0.7388 | 0 | 0 | 0 | 0.0005 | 0 | 0 | 0 | 0 | 0 | 0.4128 | 0.0120 | 0 | 0 | 0 | 0 | 0.0157 | 0.4749 | 0.0068 | 0.1607 |
| **Laptev Sea** | Arctic | 0.6509 | 0.0000 | 0 | 0 | 0 | 0.0015 | 0.0013 | 0.0005 | 0.0000 | 0.0006 | 0.2291 | 0.0006 | 0 | 0.0001 | 0 | 0 | 0 | 0.2528 | 0 | 0.1956 |
| **High Arctic Archipelago** | Arctic | 0.5430 | 0.0001 | 0.0001 | 0.0000 | 0.0000 | 0 | 0.0000 | 0 | 0 | 0.0001 | 0.4323 | 0.0008 | 0 | 0.0000 | 0 | 0 | 0.0007 | 0.3203 | 0.0007 | 0.0407 |
| **Ross Sea** | Continental High Antarctic | 0.4586 | 0 | 0.0025 | 0 | 0.0001 | 0 | 0 | 0.0004 | 0 | 0 | 0.2488 | 0.0040 | 0 | 0 | 0 | 0.0031 | 0 | 0.5806 | 0.0025 | 0.0728 |
