# Supplementary material for: Spatial and temporal changes in cumulative human impacts on the world's ocean
Source: Nat Commun. 2015 Jul 14;6:7615. doi: 10.1038/ncomms8615 (PMC4510691; doi:10.1038/ncomms8615)
Supplement: Supplementary Data 4 — Average difference in impact scores for each stressor and for cumulative impact between 2013 and 2008 for each marine ecoregion of the world (MEOW; Spalding et al. 2007). Differences could only be calculated for the 12 (of 19) stressor layers that had data for both time. True zero values are indicated by zeros with no trailing decimals; very small values are zeros with several zero decimal values. [file ncomms8615-s5.doc]

## *Supplementary Data 4*

Average difference in impact scores for each stressor and for cumulative impact between 2013 and 2008 for each marine ecoregion of the world (MEOW; Spalding et al. 2007). Differences could only be calculated for the 12 (of 19) stressor layers that had data for both time. True zero values are indicated by zeros with no trailing decimals; very small values are zeros with several zero decimal values.

| **Suppl. Data 4: 2013 minus 2008 MEOW**  **Ecoregion** | **Province** | **Average cumulative impact score** | **Demersal destructive fishing** | **Demersal nondestructive high bycatch fishing** | **Demersal nondestructive low bycatch fishing** | **Direct human impact** | **Light pollution** | **Nutrient pollution** | **Oil rigs** | **Organic pollution** | **Pelagic high bycatch fishing** | **Pelagic low bycatch fishing** | **Sea surface temperature** | **UV** |
| --- | --- | --- | --- | --- | --- | --- | --- | --- | --- | --- | --- | --- | --- | --- |
| **East African Coral Coast** | Western Indian Ocean | 1.53935 | -0.01875 | -0.01878 | -0.00669 | 0.00022 | -0.00006 | 0.00119 | 0 | 0.00003 | -0.00576 | -0.00170 | 1.57053 | 0.02798 |
| **Southeast Papua New Guinea** | Eastern Coral Triangle | 1.48803 | 0.00074 | 0.00060 | 0.00912 | 0.00007 | 0.00001 | -0.00059 | 0 | -0.00004 | 0.00007 | 0.01436 | 1.49468 | -0.02765 |
| **Ningaloo** | Northwest Australian Shelf | 1.43703 | -0.00021 | -0.00016 | -0.00010 | 0.00012 | 0.00000 | -0.00009 | 0.00018 | 0.00000 | -0.00197 | -0.00005 | 1.44679 | -0.00406 |
| **Solomon Sea** | Eastern Coral Triangle | 1.41293 | 0.00218 | 0.00118 | 0.01045 | 0.00023 | 0.00000 | -0.00005 | 0 | -0.00001 | 0 | 0.01722 | 1.44496 | -0.05866 |
| **Lesser Sunda** | Western Coral Triangle | 1.39979 | -0.00541 | -0.00455 | -0.00279 | 0.00006 | -0.00006 | 0.00104 | 0 | 0.00008 | -0.00013 | -0.00076 | 1.41134 | 0.01032 |
| **Exmouth to Broome** | Northwest Australian Shelf | 1.37901 | -0.00159 | -0.00094 | -0.00038 | 0.00012 | 0.00002 | 0.00007 | -0.00002 | 0.00000 | -0.00036 | -0.00005 | 1.37701 | 0.00930 |
| **Seychelles** | Western Indian Ocean | 1.37881 | -0.00186 | -0.00069 | -0.00636 | 0.00000 | -0.00001 | 0.00000 | 0 | 0.00000 | -0.00587 | -0.00911 | 1.38171 | 0.02115 |
| **Mascarene Islands** | Western Indian Ocean | 1.37139 | -0.00289 | -0.00245 | -0.00711 | 0.00001 | 0.00000 | -0.00010 | 0 | 0.00000 | 0 | -0.01081 | 1.38744 | 0.00760 |
| **Southern Java** | Java Transitional | 1.36669 | -0.00739 | -0.00679 | -0.00434 | 0.00006 | 0.00001 | 0.00062 | -0.00015 | 0.00016 | -0.00018 | -0.00115 | 1.34947 | 0.03971 |
| **Coral Sea** | Tropical Southwestern Pacific | 1.27518 | -0.00010 | -0.00004 | -0.00150 | 0 | 0 | 0 | 0 | 0 | -0.00008 | -0.00212 | 1.25544 | 0.02357 |
| **Fernando de Naronha and Atoll das Rocas** | Tropical Southwestern Atlantic | 1.23758 | 0.00031 | 0.00036 | 0.00028 | 0.00014 | 0.00000 | 0 | 0 | 0 | 0.00246 | 0.00028 | 1.22648 | 0.00737 |
| **Western Galapagos Islands** | Galapagos | 1.23101 | 0.00052 | 0.00113 | 0.00176 | -0.00044 | 0.00001 | 0.00015 | 0 | 0.00002 | 0.00002 | 0.00157 | 1.23727 | -0.00999 |
| **St. Helena and Ascension Islands** | St. Helena and Ascension Islands | 1.21515 | -0.00007 | -0.00002 | -0.00025 | 0.00000 | 0.00000 | 0 | 0 | 0 | -0.00001 | -0.00020 | 1.22927 | -0.01349 |
| **Shark Bay** | West Central Australian Shelf | 1.20018 | -0.00036 | -0.00033 | -0.00011 | -0.00001 | 0.00000 | -0.00003 | 0 | -0.00001 | -0.00022 | -0.00003 | 1.20782 | -0.00030 |
| **Western and Northern Madagascar** | Western Indian Ocean | 1.19949 | -0.01946 | -0.00636 | -0.00800 | 0.00012 | -0.00002 | 0.00002 | 0 | 0.00000 | -0.00056 | -0.00644 | 1.21245 | 0.03140 |
| **Northeastern Brazil** | Tropical Southwestern Atlantic | 1.18291 | 0.00287 | 0.00164 | 0.00109 | 0.00022 | 0.00000 | 0.00135 | 0.00003 | 0.00024 | 0.00366 | 0.00039 | 1.19314 | -0.01840 |
| **Southeast Madagascar** | Western Indian Ocean | 1.17368 | -0.02049 | -0.00577 | -0.00743 | 0.00007 | 0.00001 | 0.00003 | 0 | 0.00000 | 0 | -0.00610 | 1.23172 | -0.01498 |
| **Clipperton** | Tropical East Pacific | 1.17327 | -0.00019 | -0.00162 | -0.00103 | 0 | 0 | 0 | 0 | 0 | -0.00596 | -0.00156 | 1.17507 | 0.00855 |
| **Cargados Carajos/Tromelin Island** | Western Indian Ocean | 1.14377 | -0.01729 | -0.01001 | -0.01249 | 0.00000 | 0 | 0 | 0 | 0 | -0.00249 | -0.01242 | 1.23554 | -0.03707 |
| **Halmahera** | Western Coral Triangle | 1.14105 | -0.00501 | -0.00515 | -0.00447 | 0.00026 | 0.00000 | 0.00037 | 0 | 0.00001 | -0.00020 | -0.00266 | 1.17709 | -0.01236 |
| **Panama Bight** | Tropical East Pacific | 1.10296 | -0.00397 | -0.00023 | -0.00033 | 0.00016 | -0.00001 | 0.00030 | 0 | -0.00053 | -0.00017 | -0.00039 | 1.05677 | 0.05431 |
| **Banda Sea** | Western Coral Triangle | 1.09558 | -0.00598 | -0.00506 | -0.00415 | 0.00005 | 0.00000 | 0.00027 | 0.00002 | 0.00001 | -0.00020 | -0.00283 | 1.11108 | 0.00871 |
| **Eastern Galapagos Islands** | Galapagos | 1.09194 | 0.00233 | 0.00165 | 0.00309 | -0.00081 | 0.00000 | 0.00048 | 0 | 0.00008 | 0.00038 | 0.00194 | 1.10573 | -0.02177 |
| **New Caledonia** | Tropical Southwestern Pacific | 1.03411 | -0.00483 | -0.00308 | -0.00323 | 0.00003 | 0.00001 | -0.00005 | 0 | -0.00001 | 0 | -0.00295 | 1.02892 | 0.02025 |
| **Gulf of Guinea Islands** | Gulf of Guinea | 1.03073 | 0.00017 | 0.00013 | 0.00009 | -0.00003 | 0.00000 | -0.00004 | 0 | -0.00001 | 0.00310 | -0.00004 | 1.03638 | -0.00871 |
| **Sulawesi Sea/Makassar Strait** | Western Coral Triangle | 1.02286 | -0.00746 | -0.00614 | -0.00425 | 0.00034 | -0.00006 | 0.00124 | -0.00001 | 0.00012 | -0.00017 | -0.00227 | 1.05074 | -0.00556 |
| **Sao Pedro and Sao Paulo Islands** | Tropical Southwestern Atlantic | 1.00584 | -0.00057 | -0.00013 | -0.00019 | 0 | 0 | 0 | 0 | 0 | 0 | -0.00019 | 1.00173 | 0.00520 |
| **Houtman** | West Central Australian Shelf | 1.00415 | -0.00030 | -0.00049 | -0.00009 | 0.00022 | 0.00001 | -0.00042 | 0 | -0.00008 | -0.00003 | -0.00003 | 0.99725 | 0.01017 |
| **Tristan Gough** | Tristan Gough | 0.98237 | -0.00005 | -0.00009 | -0.00046 | -0.00002 | 0.00000 | 0 | 0 | 0 | -0.00001 | -0.00018 | 0.98967 | -0.00644 |
| **Western Sumatra** | Andaman | 0.95702 | -0.00958 | -0.00937 | -0.00578 | 0.00007 | -0.00002 | 0.00086 | 0 | 0.00012 | -0.00038 | -0.00117 | 0.97325 | 0.01124 |
| **Northeast Sulawesi** | Western Coral Triangle | 0.94821 | -0.01150 | -0.01013 | -0.00642 | -0.00239 | 0.00006 | 0.00915 | 0 | 0.00082 | -0.00020 | -0.00206 | 0.99332 | -0.01083 |
| **Gulf of Guinea Upwelling** | Gulf of Guinea | 0.94472 | -0.01388 | -0.00818 | -0.01083 | 0.00012 | 0.00002 | 0.00031 | 0.00014 | 0.00007 | -0.02616 | -0.00296 | 1.00461 | 0.00388 |
| **Gulf of Guinea Central** | Gulf of Guinea | 0.94398 | -0.00040 | -0.00056 | -0.00061 | 0.00040 | -0.00005 | -0.00047 | 0.00364 | 0.00005 | 0.00208 | 0.00004 | 0.96023 | -0.01441 |
| **Angolan** | Gulf of Guinea | 0.91206 | -0.00843 | -0.00628 | -0.00238 | 0.00060 | 0.00006 | 0.00012 | 0.00156 | 0.00002 | -0.00001 | -0.00022 | 0.94519 | -0.01577 |
| **Magdalena Transition** | Warm Temperate Northeast Pacific | 0.90401 | -0.00114 | -0.00047 | -0.00247 | 0.00014 | 0.00002 | 0.00091 | 0 | 0.00045 | -0.00041 | -0.00019 | 0.92057 | -0.00988 |
| **West Caroline Islands** | Tropical Northwestern Pacific | 0.88026 | -0.00003 | -0.00010 | -0.00016 | -0.00003 | -0.00001 | 0.00000 | 0 | 0.00000 | 0.00000 | -0.00024 | 0.88814 | -0.00724 |
| **Juan Fernandez and Desventuradas** | Juan Fernandez and Desventuradas | 0.87658 | -0.00366 | -0.02650 | -0.03725 | 0 | 0.00000 | 0 | 0 | 0 | 0 | -0.00081 | 0.92715 | 0.01772 |
| **Papua** | Western Coral Triangle | 0.86375 | -0.00423 | -0.00492 | -0.00363 | 0.00144 | 0.00002 | 0.00033 | 0.00000 | 0.00004 | -0.00012 | -0.00217 | 0.87676 | 0.00433 |
| **Palawan/North Borneo** | Western Coral Triangle | 0.85006 | -0.01819 | -0.01615 | -0.00817 | 0.00079 | -0.00007 | 0.00046 | 0.00051 | 0.00027 | -0.00082 | -0.00175 | 0.92988 | -0.02839 |
| **Bight of Sofala/Swamp Coast** | Western Indian Ocean | 0.84937 | -0.00982 | -0.00100 | -0.00127 | 0.00004 | 0.00001 | 0.00117 | 0 | -0.00008 | 0 | -0.00112 | 0.83115 | 0.03419 |
| **Delagoa** | Western Indian Ocean | 0.83343 | -0.00642 | -0.00073 | -0.00182 | 0.00004 | 0.00002 | 0.00029 | 0 | 0.00003 | -0.00047 | -0.00088 | 0.79688 | 0.04869 |
| **Chiapas-Nicaragua** | Tropical East Pacific | 0.82335 | 0.00550 | 0.00261 | 0.00188 | -0.00001 | -0.00003 | -0.00136 | 0 | 0.00081 | 0.00388 | 0.00144 | 0.81402 | -0.00174 |
| **Manning-Hawkesbury** | East Central Australian Shelf | 0.81978 | -0.00076 | -0.00036 | -0.00022 | 0.00020 | 0.00007 | 0.00015 | 0 | 0.00010 | -0.00001 | -0.00006 | 0.76641 | 0.05631 |
| **South China Sea Oceanic Islands** | South China Sea | 0.78747 | -0.00882 | -0.01473 | -0.00777 | 0 | 0.00000 | 0 | 0 | 0 | -0.00139 | -0.00503 | 0.83388 | -0.00867 |
| **Nicoya** | Tropical East Pacific | 0.78508 | 0.01673 | 0.00578 | 0.00670 | 0.00004 | 0.00000 | -0.00071 | 0 | 0.00107 | 0.01204 | 0.00122 | 0.68981 | 0.05571 |
| **Three Kings-North Cape** | Northern New Zealand | 0.78189 | -0.01477 | -0.01959 | -0.00218 | -0.00002 | 0 | -0.00003 | 0 | 0.00000 | -0.00021 | -0.00049 | 0.84082 | -0.02141 |
| **Eastern Philippines** | Western Coral Triangle | 0.77370 | 0.00084 | 0.00120 | 0.00058 | 0.00083 | -0.00013 | -0.00166 | 0 | -0.00017 | -0.00002 | 0.00014 | 0.80515 | -0.02589 |
| **Gulf of Guinea South** | Gulf of Guinea | 0.77243 | -0.00426 | -0.00396 | -0.00221 | 0.00034 | -0.00004 | 0.00031 | -0.00076 | 0.00010 | -0.00436 | -0.00044 | 0.76589 | 0.02418 |
| **Bonaparte Coast** | Sahul Shelf | 0.76931 | -0.00358 | -0.00176 | -0.00100 | 0.00040 | 0.00002 | -0.00142 | 0.00000 | -0.00013 | -0.00022 | -0.00038 | 0.77894 | 0.00602 |
| **Chagos** | Central Indian Ocean Islands | 0.75940 | -0.00093 | -0.00082 | -0.00441 | 0 | -0.00002 | 0 | 0 | 0 | 0.00000 | -0.00677 | 0.79721 | -0.02486 |
| **Bismarck Sea** | Eastern Coral Triangle | 0.74734 | 0.00226 | 0.00152 | 0.00886 | 0.00025 | 0.00000 | 0.00001 | 0 | 0.00002 | 0 | 0.01399 | 0.75729 | -0.03448 |
| **South India and Sri Lanka** | West and South Indian Shelf | 0.73607 | -0.00305 | -0.00280 | -0.00191 | 0.00003 | -0.00003 | 0.00111 | 0 | -0.00013 | -0.00661 | -0.00088 | 0.76838 | -0.01591 |
| **Central Somali Coast** | Somali/Arabian | 0.70761 | -0.00566 | -0.00224 | -0.00182 | 0.00023 | 0.00000 | 0 | 0 | 0 | -0.00035 | -0.00162 | 0.65623 | 0.06441 |
| **Cocos-Keeling/Christmas Island** | Java Transitional | 0.70638 | 0.00007 | 0.00009 | 0.00008 | 0.00004 | 0.00001 | 0 | 0 | 0 | 0.00000 | 0.00015 | 0.65866 | 0.04729 |
| **Malacca Strait** | Sunda Shelf | 0.69323 | -0.03484 | -0.02994 | -0.01561 | 0.00152 | -0.00016 | 0.00539 | -0.00011 | 0.00153 | -0.00023 | -0.00095 | 0.76251 | 0.01876 |
| **South Georgia** | Scotia Sea | 0.69129 | -0.00150 | 0.00000 | -0.00062 | 0 | 0.00000 | 0 | 0 | 0 | 0 | -0.00033 | 0.70347 | -0.00929 |
| **Northern Monsoon Current Coast** | Western Indian Ocean | 0.68323 | -0.00526 | -0.00292 | -0.00212 | 0.00009 | 0.00000 | 0.00085 | 0 | 0.00021 | -0.00458 | -0.00198 | 0.77006 | -0.06816 |
| **Northern Galapagos Islands** | Galapagos | 0.66711 | 0.00109 | 0.00108 | 0.00250 | 0.00000 | 0 | 0.00000 | 0 | 0.00000 | 0.00067 | 0.00290 | 0.64672 | 0.01214 |
| **Trindade and Martin Vaz Islands** | Tropical Southwestern Atlantic | 0.66653 | -0.00177 | -0.00113 | -0.00117 | 0 | 0 | 0 | 0 | 0 | -0.00381 | -0.00153 | 0.67453 | 0.00142 |
| **Oyashio Current** | Cold Temperate Northwest Pacific | 0.66388 | 0.01101 | 0.00268 | 0.00453 | -0.00005 | -0.00002 | 0.00015 | 0 | 0.00000 | 0 | -0.00032 | 0.61468 | 0.03243 |
| **Guayaquil** | Tropical East Pacific | 0.65875 | -0.00296 | -0.00224 | -0.00523 | 0.00003 | 0.00000 | 0.00018 | 0 | -0.00039 | -0.00079 | -0.00039 | 0.70890 | -0.03674 |
| **Western India** | West and South Indian Shelf | 0.65710 | -0.01925 | -0.01507 | -0.00729 | 0.00023 | 0.00013 | 0.00376 | 0.00004 | -0.00003 | 0 | -0.00074 | 0.68243 | 0.01576 |
| **Andaman Sea Coral Coast** | Andaman | 0.65598 | -0.05323 | -0.04425 | -0.02069 | 0.00082 | 0.00017 | -0.00109 | -0.00006 | -0.00017 | -0.00043 | -0.00082 | 0.76212 | 0.01938 |
| **Maldives** | Central Indian Ocean Islands | 0.64970 | -0.00793 | -0.00276 | -0.01087 | 0.00000 | 0.00005 | 0.00000 | 0 | 0.00000 | -0.00006 | -0.01654 | 0.66863 | 0.01919 |
| **Southern Cook/Austral Islands** | Southeast Polynesia | 0.64680 | -0.00035 | -0.00016 | -0.00052 | -0.00002 | 0.00000 | 0.00000 | 0 | 0.00000 | 0 | -0.00076 | 0.64829 | 0.00035 |
| **South European Atlantic Shelf** | Lusitanian | 0.64493 | -0.01206 | -0.00862 | -0.00658 | 0.00005 | -0.00005 | -0.00110 | 0 | -0.00039 | 0 | -0.00368 | 0.69452 | -0.01478 |
| **Lord Howe and Norfolk Islands** | Lord Howe and Norfolk Islands | 0.62738 | -0.00539 | -0.00285 | -0.00084 | 0.00007 | 0.00000 | 0 | 0 | 0 | 0 | -0.00020 | 0.65556 | -0.01894 |
| **Eastern Brazil** | Tropical Southwestern Atlantic | 0.62573 | -0.00462 | -0.00159 | -0.00215 | 0.00012 | 0.00006 | 0.00050 | 0.00067 | -0.00005 | -0.00669 | -0.00146 | 0.62114 | 0.02113 |
| **Torres Strait Northern Great Barrier Reef** | Northeast Australian Shelf | 0.60563 | -0.00090 | -0.00053 | -0.00070 | -0.00017 | -0.00003 | 0.00049 | 0 | 0.00002 | -0.00006 | -0.00062 | 0.60105 | 0.00980 |
| **Gulf of Papua** | Sahul Shelf | 0.58548 | 0.00472 | 0.00202 | 0.00517 | 0.00045 | -0.00002 | -0.00223 | 0 | -0.00016 | 0.00011 | 0.00518 | 0.60363 | -0.02172 |
| **Revillagigedos** | Tropical East Pacific | 0.57562 | -0.00022 | -0.00055 | -0.00021 | 0.00014 | 0.00000 | 0.00000 | 0 | 0.00000 | -0.00116 | -0.00034 | 0.57430 | 0.00372 |
| **Leeuwin** | Southwest Australian Shelf | 0.56600 | -0.00030 | -0.00030 | -0.00008 | 0.00006 | 0.00000 | -0.00008 | 0 | 0.00000 | -0.00001 | -0.00003 | 0.53597 | 0.03176 |
| **Southern Vietnam** | Sunda Shelf | 0.56429 | -0.04009 | -0.04435 | -0.01709 | 0.00034 | 0.00010 | -0.00093 | -0.00020 | -0.00004 | -0.00046 | -0.00183 | 0.67320 | 0.00039 |
| **Eastern India** | Bay of Bengal | 0.56330 | -0.00899 | -0.00742 | -0.00435 | -0.00003 | 0.00007 | 0.00218 | 0.00000 | -0.00025 | -0.00383 | -0.00085 | 0.57237 | 0.01570 |
| **Chiloense** | Magellanic | 0.56209 | -0.01843 | -0.00862 | -0.01374 | -0.00075 | 0.00001 | -0.00018 | 0 | 0.00001 | 0 | -0.00001 | 0.68525 | -0.06870 |
| **Andaman and Nicobar Islands** | Andaman | 0.55219 | 0.00050 | 0.00089 | 0.00045 | -0.00010 | 0.00000 | 0.00024 | 0 | 0.00000 | -0.00008 | 0.00011 | 0.54934 | 0.00160 |
| **Tweed-Moreton** | East Central Australian Shelf | 0.54597 | -0.00062 | -0.00026 | -0.00041 | -0.00013 | 0.00003 | -0.00071 | 0 | -0.00002 | -0.00002 | -0.00044 | 0.49114 | 0.05933 |
| **Sunda Shelf/Java Sea** | Sunda Shelf | 0.53761 | -0.05264 | -0.04675 | -0.02392 | 0.00019 | -0.00006 | 0.00288 | 0.00016 | 0.00069 | -0.00110 | -0.00579 | 0.66626 | 0.00012 |
| **Northern Bay of Bengal** | Bay of Bengal | 0.53281 | -0.03898 | -0.03994 | -0.01340 | -0.00001 | 0.00002 | 0.00058 | 0.00000 | 0.00043 | -0.00091 | -0.00066 | 0.65503 | -0.02398 |
| **Gulf of Guinea West** | Gulf of Guinea | 0.51419 | 0.00110 | 0.00024 | -0.00020 | 0.00008 | 0.00001 | 0.00032 | 0 | 0.00007 | -0.00260 | -0.00017 | 0.51286 | 0.00463 |
| **Solomon Archipelago** | Eastern Coral Triangle | 0.50798 | -0.00526 | -0.00185 | -0.01410 | 0.00012 | 0.00000 | 0.00003 | 0 | 0.00000 | -0.00060 | -0.02176 | 0.55188 | 0.00051 |
| **Great Australian Bight** | Southwest Australian Shelf | 0.50499 | -0.00097 | -0.00108 | -0.00020 | 0.00005 | 0.00000 | -0.00021 | 0 | -0.00002 | -0.00003 | -0.00003 | 0.58824 | -0.07930 |
| **Arafura Sea** | Sahul Shelf | 0.49783 | -0.00815 | -0.00634 | -0.00339 | 0.00057 | 0.00000 | -0.00127 | 0 | -0.00007 | -0.00003 | -0.00115 | 0.47800 | 0.04243 |
| **Kermadec Island** | Northern New Zealand | 0.48963 | -0.00664 | -0.00255 | -0.00137 | -0.00008 | 0 | 0 | 0 | 0 | 0 | -0.00024 | 0.40965 | 0.09168 |
| **Mexican Tropical Pacific** | Tropical East Pacific | 0.48711 | -0.00113 | -0.00050 | -0.00038 | 0.00000 | 0.00002 | -0.00028 | 0 | 0.00044 | -0.00096 | -0.00030 | 0.47156 | 0.01943 |
| **South Shetland Islands** | Scotia Sea | 0.47681 | -0.00144 | -0.00026 | 0.00655 | 0 | 0 | 0 | 0 | 0 | 0 | 0 | 0.49635 | -0.01564 |
| **Bouvet Island** | Subantarctic Islands | 0.47232 | 0 | 0 | 0 | 0 | 0 | 0 | 0 | 0 | 0 | 0 | 0.48037 | -0.00805 |
| **Greater Antilles** | Tropical Northwestern Atlantic | 0.46316 | -0.01151 | -0.00862 | -0.00483 | -0.00012 | -0.00010 | 0.00057 | 0 | 0.00032 | -0.00337 | -0.00057 | 0.43233 | 0.06161 |
| **South Kuroshio** | South Kuroshio | 0.45555 | -0.00589 | -0.00727 | -0.00447 | -0.00001 | -0.00008 | -0.00006 | 0 | -0.00001 | -0.00230 | -0.00248 | 0.47552 | 0.00319 |
| **Gulf of Aden** | Red Sea and Gulf of Aden | 0.44912 | -0.00568 | -0.00182 | -0.00194 | 0.00074 | 0.00004 | 0.00035 | 0 | -0.00004 | -0.00358 | -0.00113 | 0.50004 | -0.03634 |
| **Western Caribbean** | Tropical Northwestern Atlantic | 0.44464 | -0.00615 | -0.00386 | -0.00183 | 0.00067 | -0.00001 | -0.00061 | 0 | 0.00102 | -0.00185 | -0.00008 | 0.41423 | 0.04506 |
| **Central and Southern Great Barrier Reef** | Northeast Australian Shelf | 0.43949 | -0.00121 | -0.00060 | -0.00065 | 0.00041 | -0.00001 | 0.00018 | 0 | 0.00007 | -0.00005 | -0.00072 | 0.43424 | 0.01160 |
| **Arnhem Coast to Gulf of Carpenteria** | Sahul Shelf | 0.43049 | -0.00179 | -0.00095 | -0.00072 | 0.00040 | 0.00000 | 0.00089 | 0 | 0.00007 | -0.00005 | -0.00060 | 0.41407 | 0.02251 |
| **Mariana Islands** | Tropical Northwestern Pacific | 0.42480 | 0.00187 | 0.00503 | 0.01151 | -0.00002 | -0.00002 | 0 | 0 | 0 | 0 | 0.01778 | 0.38068 | 0.00807 |
| **Central Peru** | Warm Temperate Southeastern Pacific | 0.41460 | -0.01723 | -0.00655 | -0.01853 | 0.00007 | 0.00003 | 0.00035 | 0 | 0.00012 | -0.00126 | -0.00147 | 0.50989 | -0.05046 |
| **Cocos Islands** | Tropical East Pacific | 0.40427 | 0.00312 | 0.00248 | 0.00203 | 0 | 0 | 0 | 0 | 0 | 0.01454 | 0.00191 | 0.37575 | 0.00445 |
| **Gulf of Tonkin** | South China Sea | 0.39487 | -0.02912 | -0.02762 | -0.00918 | 0.00043 | -0.00030 | -0.00152 | -0.00006 | -0.00067 | -0.00186 | -0.00059 | 0.45394 | 0.01576 |
| **Humboldtian** | Warm Temperate Southeastern Pacific | 0.39058 | -0.00985 | -0.00896 | -0.01850 | 0.00004 | 0.00004 | 0.00026 | 0 | 0.00010 | -0.00083 | -0.00119 | 0.42610 | 0.00379 |
| **Amsterdam-St Paul** | Amsterdam-St Paul | 0.38399 | -0.00015 | -0.00010 | -0.00008 | 0 | 0.00000 | 0 | 0 | 0 | 0 | -0.00016 | 0.37879 | 0.00571 |
| **Sea of Okhotsk** | Cold Temperate Northwest Pacific | 0.38320 | 0.06777 | 0.02007 | 0.02018 | 0.00016 | 0.00006 | 0.00013 | -0.00002 | -0.00004 | 0 | -0.00002 | 0.23179 | 0.04473 |
| **Saharan Upwelling** | Lusitanian | 0.38279 | 0.00036 | 0.00133 | -0.00003 | -0.00003 | -0.00001 | -0.00073 | 0 | -0.00080 | 0.00006 | 0.00003 | 0.36656 | 0.01725 |
| **Cortezian** | Warm Temperate Northeast Pacific | 0.37832 | -0.00297 | -0.00054 | -0.00324 | 0.00094 | 0.00004 | 0.00014 | 0 | 0.00250 | -0.00015 | -0.00020 | 0.39633 | -0.00970 |
| **Amazonia** | North Brazil Shelf | 0.37014 | 0.00315 | 0.00220 | 0.00109 | -0.00018 | 0.00003 | 0.00011 | 0 | 0.00001 | 0.00212 | 0.00018 | 0.35197 | 0.01204 |
| **Vanuatu** | Tropical Southwestern Pacific | 0.36450 | -0.00233 | -0.00235 | -0.01258 | 0.00002 | 0.00000 | 0.00001 | 0 | 0.00000 | -0.00020 | -0.01973 | 0.40595 | -0.00384 |
| **South Sandwich Islands** | Scotia Sea | 0.36427 | -0.00001 | 0 | 0.00000 | 0 | 0.00000 | 0 | 0 | 0 | 0 | 0 | 0.32865 | 0.03571 |
| **Central Chile** | Warm Temperate Southeastern Pacific | 0.36393 | -0.00358 | -0.00976 | -0.01831 | 0.00006 | 0.00002 | -0.00024 | 0 | -0.00001 | 0 | -0.00010 | 0.33008 | 0.06620 |
| **Southwestern Caribbean** | Tropical Northwestern Atlantic | 0.35685 | -0.00284 | -0.00340 | -0.00128 | 0.00008 | -0.00002 | -0.00027 | 0 | -0.00018 | 0.00008 | -0.00003 | 0.36714 | -0.00173 |
| **Araucanian** | Warm Temperate Southeastern Pacific | 0.34935 | -0.00541 | -0.00732 | -0.01494 | 0.00004 | 0.00001 | 0.00067 | 0 | 0.00085 | 0 | -0.00003 | 0.36742 | 0.00853 |
| **Northeastern New Zealand** | Northern New Zealand | 0.34144 | -0.01242 | -0.00804 | -0.00335 | 0.00018 | -0.00007 | -0.00112 | 0 | -0.00079 | -0.00013 | -0.00036 | 0.33718 | 0.03188 |
| **East Caroline Islands** | Tropical Northwestern Pacific | 0.33883 | -0.00015 | -0.00038 | -0.00072 | 0.00003 | 0.00000 | 0 | 0 | 0 | 0 | -0.00110 | 0.32855 | 0.01262 |
| **Peter the First Island** | Subantarctic Islands | 0.32192 | 0 | 0 | 0 | 0 | 0 | 0 | 0 | 0 | 0 | 0 | 0.40462 | -0.07242 |
| **Southern California Bight** | Warm Temperate Northeast Pacific | 0.31699 | -0.00112 | -0.00052 | -0.00190 | 0.00004 | -0.00003 | -0.00024 | 0 | -0.00002 | -0.00004 | -0.00018 | 0.31988 | 0.00228 |
| **Rio de la Plata** | Warm Temperate Southwestern Atlantic | 0.30429 | -0.00958 | -0.00288 | -0.00114 | 0.00011 | 0.00016 | 0.00063 | 0 | 0.00314 | 0.00000 | 0.00000 | 0.31972 | 0.00162 |
| **East Antarctic Enderby Land** | Continental High Antarctic | 0.29759 | 0.00004 | 0 | 0 | 0 | 0 | 0 | 0 | 0 | 0 | 0.00001 | 0.29421 | 0.02782 |
| **Gulf of Thailand** | Sunda Shelf | 0.29335 | -0.10365 | -0.09100 | -0.04336 | 0.00107 | -0.00052 | -0.00075 | -0.00009 | -0.00059 | -0.00004 | -0.00260 | 0.53901 | 0.00038 |
| **Eastern Caribbean** | Tropical Northwestern Atlantic | 0.28470 | -0.00402 | -0.00467 | -0.00363 | 0.00009 | -0.00007 | -0.00005 | 0 | 0.00000 | -0.00109 | -0.00169 | 0.26495 | 0.03519 |
| **Society Islands** | Southeast Polynesia | 0.28255 | -0.00189 | -0.00021 | -0.00043 | 0.00003 | 0.00001 | -0.00001 | 0 | 0.00000 | 0 | -0.00066 | 0.27745 | 0.00839 |
| **Fiji Islands** | Tropical Southwestern Pacific | 0.27518 | -0.00869 | -0.00733 | -0.00527 | -0.00011 | -0.00003 | -0.00011 | 0 | -0.00001 | 0 | -0.00161 | 0.32277 | -0.02357 |
| **Western Arabian Sea** | Somali/Arabian | 0.27468 | -0.00176 | -0.00080 | -0.00106 | 0.00046 | 0.00006 | -0.00006 | 0 | -0.00006 | -0.00016 | -0.00044 | 0.32562 | -0.04595 |
| **South Australian Gulfs** | Southwest Australian Shelf | 0.26952 | -0.00100 | -0.00099 | -0.00019 | 0.00040 | -0.00001 | -0.00014 | 0 | 0.00002 | -0.00002 | -0.00002 | 0.27553 | -0.00153 |
| **Southern Caribbean** | Tropical Northwestern Atlantic | 0.26919 | -0.02884 | -0.01126 | -0.02398 | 0.00034 | -0.00007 | 0.00221 | 0.00003 | 0.00038 | -0.00044 | -0.00187 | 0.32626 | 0.00790 |
| **Tuamotus** | Southeast Polynesia | 0.25581 | -0.00112 | -0.00028 | -0.00077 | 0.00005 | 0.00000 | 0 | 0 | 0 | 0 | -0.00115 | 0.27915 | -0.02007 |
| **Tonga Islands** | Tropical Southwestern Pacific | 0.25120 | -0.00103 | -0.00125 | -0.00081 | -0.00006 | -0.00001 | 0.00001 | 0 | 0.00000 | 0 | -0.00059 | 0.26821 | -0.01321 |
| **Guianan** | North Brazil Shelf | 0.24480 | -0.02165 | -0.00869 | -0.00810 | 0.00005 | 0.00001 | 0.00154 | -0.00001 | 0.00008 | -0.00419 | -0.00032 | 0.28923 | -0.00158 |
| **Virginian** | Cold Temperate Northwest Atlantic | 0.23581 | -0.02162 | -0.00951 | -0.00657 | -0.00058 | -0.00061 | -0.00096 | 0 | -0.00016 | 0 | -0.00131 | 0.27533 | 0.00552 |
| **Western Mediterranean** | Mediterranean Sea | 0.23090 | -0.01614 | -0.01642 | -0.01383 | 0.00006 | -0.00007 | -0.00168 | 0 | -0.00039 | 0 | -0.00319 | 0.25045 | 0.03337 |
| **Carolinian** | Warm Temperate Northwest Atlantic | 0.22735 | -0.00931 | -0.00309 | -0.00350 | -0.00014 | -0.00014 | -0.00037 | 0 | -0.00010 | -0.00052 | -0.00032 | 0.24850 | -0.00287 |
| **Natal** | Agulhas | 0.22590 | -0.00454 | -0.00065 | -0.00445 | 0.00006 | -0.00001 | 0.00044 | 0 | 0.00019 | -0.00033 | -0.00076 | 0.13607 | 0.10023 |
| **Southern Grand Banks - South Newfoundland** | Cold Temperate Northwest Atlantic | 0.22344 | -0.03945 | -0.02155 | -0.00799 | -0.00006 | -0.00001 | 0.00047 | -0.00001 | 0.00003 | 0 | -0.00377 | 0.37474 | -0.07719 |
| **Cape Verde** | West African Transition | 0.22274 | -0.00123 | -0.00049 | -0.00277 | 0.00001 | -0.00001 | 0.00001 | 0 | 0.00000 | 0 | -0.00314 | 0.21549 | 0.01503 |
| **Gulf of St. Lawrence - Eastern Scotian Shelf** | Cold Temperate Northwest Atlantic | 0.19929 | -0.02422 | -0.02222 | -0.00910 | -0.00021 | -0.00014 | -0.00020 | 0 | 0.00012 | 0 | -0.00697 | 0.27044 | -0.00514 |
| **Alboran Sea** | Mediterranean Sea | 0.19537 | -0.01944 | -0.02872 | -0.01788 | 0.00028 | 0.00009 | 0.00051 | 0 | -0.00042 | 0 | -0.00369 | 0.30292 | -0.03651 |
| **Channels and Fjords of Southern Chile** | Magellanic | 0.18956 | -0.01493 | -0.00200 | -0.00145 | -0.00325 | -0.00002 | 0.00018 | 0.00000 | -0.00008 | 0 | 0.00000 | 0.22298 | -0.00753 |
| **Kamchatka Shelf and Coast** | Cold Temperate Northwest Pacific | 0.18923 | 0.01379 | 0.00389 | 0.01260 | 0.00005 | 0.00001 | 0.00013 | 0 | 0.00001 | 0 | 0.00000 | 0.14765 | 0.01183 |
| **Western Bassian** | Southeast Australian Shelf | 0.18736 | -0.00060 | -0.00046 | -0.00010 | 0.00007 | 0.00001 | 0.00000 | 0 | -0.00008 | -0.00001 | -0.00002 | 0.21159 | -0.02264 |
| **Southern Gulf of Mexico** | Tropical Northwestern Atlantic | 0.18540 | -0.00309 | -0.00227 | -0.00170 | 0.00009 | -0.00002 | -0.00105 | 0.00002 | 0.00077 | -0.00189 | -0.00020 | 0.17289 | 0.02211 |
| **Gulf of Oman** | Somali/Arabian | 0.18381 | -0.01986 | -0.01117 | -0.00674 | 0.00038 | 0.00005 | -0.00049 | -0.00002 | -0.00007 | -0.01362 | -0.00128 | 0.23968 | -0.00149 |
| **Aegean Sea** | Mediterranean Sea | 0.18080 | 0.04765 | 0.03091 | 0.03014 | -0.00001 | -0.00048 | -0.00203 | 0 | 0.00000 | 0 | 0.00797 | 0.07711 | -0.00874 |
| **Southern China** | South China Sea | 0.18071 | -0.03294 | -0.03090 | -0.01174 | 0.00007 | -0.00013 | 0.00044 | -0.00021 | -0.00017 | -0.00234 | -0.00051 | 0.24319 | 0.01778 |
| **Heard and Macdonald Islands** | Subantarctic Islands | 0.17847 | -0.01083 | 0 | 0.00000 | 0 | 0.00000 | 0 | 0 | 0 | 0 | -0.00148 | 0.17349 | 0.01736 |
| **Ogasawara Islands** | Tropical Northwestern Pacific | 0.16704 | -0.00372 | -0.00699 | -0.00536 | 0.00000 | 0.00000 | 0 | 0 | 0 | -0.00082 | -0.00335 | 0.17346 | 0.01383 |
| **Hudson Complex** | Arctic | 0.16386 | -0.00011 | -0.00001 | 0.00000 | 0.00009 | 0.00003 | 0.00008 | 0 | 0.00004 | 0 | 0.00000 | 0.20294 | -0.03634 |
| **Rio Grande** | Warm Temperate Southwestern Atlantic | 0.15837 | 0.00209 | 0.00105 | 0.00048 | -0.00135 | 0.00007 | 0.00061 | 0 | 0.00005 | 0.00027 | 0.00026 | 0.13635 | 0.01905 |
| **Antarctic Peninsula** | Scotia Sea | 0.15186 | 0.00000 | 0 | 0.01980 | 0 | 0 | 0 | 0 | 0 | 0 | 0 | 0.25008 | -0.04690 |
| **Samoa Islands** | Central Polynesia | 0.14645 | 0.00486 | 0.00251 | 0.00252 | 0.00001 | -0.00003 | 0.00000 | 0 | 0.00000 | 0 | 0.00352 | 0.14291 | -0.00979 |
| **Agulhas Bank** | Agulhas | 0.13841 | -0.00680 | -0.00152 | -0.01465 | 0.00007 | -0.00003 | -0.00029 | -0.00008 | -0.00003 | 0 | -0.00021 | 0.10213 | 0.06001 |
| **Black Sea** | Black Sea | 0.13174 | -0.02244 | -0.01930 | -0.05392 | -0.00024 | 0.00006 | 0.00254 | -0.00011 | 0.00189 | -0.00019 | -0.00590 | 0.24389 | -0.01291 |
| **Sahelian Upwelling** | West African Transition | 0.12518 | -0.01047 | -0.00991 | -0.00791 | 0.00011 | 0.00002 | -0.00061 | 0.00017 | -0.00013 | -0.00560 | -0.00121 | 0.16818 | -0.00698 |
| **Azores Canaries Madeira** | Lusitanian | 0.12097 | -0.00103 | -0.00036 | -0.00068 | 0.00001 | -0.00001 | -0.00021 | 0 | -0.00002 | -0.00001 | -0.00033 | 0.11017 | 0.01354 |
| **East Antarctic Wilkes Land** | Continental High Antarctic | 0.11899 | 0.00446 | 0 | 0.00018 | 0 | 0 | 0 | 0 | 0 | 0 | 0.00470 | 0.11851 | 0.01351 |
| **Baffin Bay - Davis Strait** | Arctic | 0.11771 | -0.01485 | -0.00103 | -0.00051 | -0.00001 | 0.00000 | 0.00000 | 0 | 0.00000 | 0 | -0.00002 | 0.10376 | 0.03243 |
| **Namib** | Benguela | 0.11629 | -0.00927 | -0.00405 | -0.02624 | 0.00001 | 0.00001 | 0.00002 | 0 | 0.00001 | 0 | -0.00012 | 0.19666 | -0.04022 |
| **Rapa-Pitcairn** | Southeast Polynesia | 0.11618 | -0.00004 | -0.00014 | -0.00030 | -0.00001 | 0.00000 | 0 | 0 | 0 | 0 | -0.00043 | 0.13273 | -0.01563 |
| **Bahamian** | Tropical Northwestern Atlantic | 0.10988 | -0.00540 | -0.00782 | -0.00273 | 0.00010 | -0.00004 | -0.00142 | 0 | -0.00011 | -0.00148 | -0.00060 | 0.07407 | 0.05549 |
| **Northern Labrador** | Arctic | 0.10739 | -0.03069 | -0.00313 | -0.00300 | 0.00000 | 0.00003 | 0.00026 | 0 | 0.00001 | 0 | -0.00241 | 0.12919 | 0.01979 |
| **Northern and Central Red Sea** | Red Sea and Gulf of Aden | 0.09370 | -0.07354 | -0.05686 | -0.02354 | 0.00090 | -0.00002 | -0.00096 | -0.00050 | -0.00010 | -0.00126 | -0.00025 | 0.23207 | 0.02111 |
| **Phoenix/Tokelau/Northern Cook Islands** | Central Polynesia | 0.08890 | -0.00008 | -0.00036 | -0.00132 | -0.00003 | 0.00000 | 0 | 0 | 0 | -0.00003 | -0.00206 | 0.11318 | -0.02040 |
| **Floridian** | Tropical Northwestern Atlantic | 0.08820 | -0.01974 | -0.01168 | -0.00666 | -0.00082 | -0.00021 | 0.00057 | 0 | 0.00017 | -0.00181 | -0.00012 | 0.13477 | -0.00551 |
| **Northern Gulf of Mexico** | Warm Temperate Northwest Atlantic | 0.08732 | -0.01149 | -0.00474 | -0.00829 | -0.00009 | -0.00010 | -0.00058 | 0.00002 | 0.00011 | -0.00099 | -0.00010 | 0.11984 | -0.00548 |
| **Gulf of Maine/Bay of Fundy** | Cold Temperate Northwest Atlantic | 0.08295 | -0.02932 | -0.01531 | -0.00871 | -0.00005 | -0.00021 | -0.00052 | 0 | -0.00005 | 0 | -0.00575 | 0.23240 | -0.08736 |
| **Bounty and Antipodes Islands** | Subantarctic New Zealand | 0.07577 | -0.00473 | -0.00083 | -0.00074 | -0.00011 | 0 | 0.00000 | 0 | 0.00000 | 0 | -0.00012 | 0.05217 | 0.03032 |
| **Chukchi Sea** | Arctic | 0.06466 | -0.00004 | -0.00001 | 0.00000 | 0.00001 | 0.00000 | 0.00045 | 0 | 0.00003 | 0 | 0 | -0.02217 | 0.08422 |
| **Southeastern Brazil** | Warm Temperate Southwestern Atlantic | 0.05420 | 0.00252 | 0.00172 | 0.00059 | 0.00018 | 0.00018 | 0.00076 | -0.00009 | 0.00004 | 0.00074 | 0.00050 | -0.04045 | 0.08735 |
| **East China Sea** | Warm Temperate Northwest Pacific | 0.05370 | -0.03269 | -0.02972 | -0.01196 | -0.00013 | 0.00009 | 0.00101 | 0.00000 | 0.00020 | -0.00226 | -0.00133 | 0.09502 | 0.03620 |
| **Auckland Island** | Subantarctic New Zealand | 0.04930 | -0.03813 | -0.00073 | -0.00150 | -0.00112 | 0 | -0.00002 | 0 | 0.00000 | 0 | -0.00006 | 0.07906 | 0.01185 |
| **Uruguay-Buenos Aires Shelf** | Warm Temperate Southwestern Atlantic | 0.04256 | -0.04803 | -0.01506 | -0.01036 | 0.00010 | 0.00002 | -0.00010 | 0 | 0.00012 | 0.00002 | -0.00016 | 0.12725 | -0.01093 |
| **Northeastern Honshu** | Cold Temperate Northwest Pacific | 0.04190 | -0.01241 | -0.00952 | -0.01047 | -0.00062 | -0.00014 | -0.00110 | 0 | -0.00053 | 0 | -0.00221 | 0.08702 | -0.00779 |
| **Lancaster Sound** | Arctic | 0.04011 | -0.00041 | -0.00002 | -0.00001 | 0.00018 | 0.00001 | 0.00001 | 0 | 0.00000 | 0 | 0 | 0.07342 | -0.02108 |
| **Sea of Japan/East Sea** | Cold Temperate Northwest Pacific | 0.03702 | -0.00413 | -0.00645 | -0.00113 | -0.00013 | -0.00015 | -0.00049 | 0 | -0.00013 | 0 | -0.00070 | 0.06426 | -0.01354 |
| **Yellow Sea** | Cold Temperate Northwest Pacific | 0.03568 | -0.02884 | -0.03375 | -0.01388 | -0.00013 | 0.00024 | 0.00170 | -0.00013 | 0.00062 | 0 | -0.00043 | 0.04497 | 0.06582 |
| **Northern Grand Banks - Southern Labrador** | Arctic | 0.02084 | -0.03879 | -0.02246 | -0.00636 | -0.00017 | 0.00006 | 0.00016 | 0 | 0.00002 | 0 | -0.00418 | 0.15062 | -0.05633 |
| **West Greenland Shelf** | Arctic | 0.01623 | -0.03811 | -0.00956 | -0.00099 | 0.00037 | 0.00003 | 0 | 0 | 0 | 0 | -0.00001 | 0.05820 | 0.00764 |
| **Marquesas** | Marquesas | 0.01293 | -0.00042 | -0.00023 | -0.00111 | -0.00003 | 0.00000 | 0.00000 | 0 | 0.00000 | 0 | -0.00141 | 0.02891 | -0.01276 |
| **Beaufort-Amundsen-Viscount Melville-Queen Maud** | Arctic | 0.00662 | -0.00002 | 0.00000 | 0 | 0.00007 | 0.00001 | 0.00011 | 0 | 0.00001 | 0 | 0 | 0.08142 | -0.04514 |
| **North Patagonian Gulfs** | Magellanic | 0.00409 | -0.08594 | -0.02249 | -0.01191 | 0.00003 | 0.00003 | 0.00000 | 0 | 0.00001 | 0.00000 | -0.00006 | 0.01668 | 0.10785 |
| **Kerguelen Islands** | Subantarctic Islands | -0.00389 | 0.00088 | 0.00001 | 0.00000 | 0 | 0.00000 | 0 | 0 | 0 | 0 | -0.00001 | -0.01252 | 0.00774 |
| **Levantine Sea** | Mediterranean Sea | -0.01422 | -0.01658 | -0.03156 | -0.00477 | 0.00018 | -0.00002 | 0.00077 | 0.00000 | 0.00026 | -0.01434 | 0.00034 | 0.10671 | -0.05452 |
| **Amundsen/Bellingshausen Sea** | Continental High Antarctic | -0.01467 | 0.00009 | 0 | 0.00000 | 0 | 0 | 0 | 0 | 0 | 0 | 0.00009 | 0.06246 | -0.05066 |
| **Laptev Sea** | Arctic | -0.01738 | 0 | 0 | 0 | -0.00087 | 0.00000 | 0.00002 | 0 | 0.00001 | 0 | 0 | -0.03948 | 0.01804 |
| **South Orkney Islands** | Scotia Sea | -0.02063 | 0.00001 | 0 | 0.01809 | 0 | 0 | 0 | 0 | 0 | 0 | 0 | -0.04252 | 0.00347 |
| **Tunisian Plateau/Gulf of Sidra** | Mediterranean Sea | -0.02445 | -0.03047 | -0.04497 | -0.01674 | 0.00045 | -0.00004 | 0.00002 | -0.00004 | -0.00003 | 0 | -0.00403 | 0.08082 | -0.00917 |
| **White Sea** | Arctic | -0.03374 | -0.04877 | -0.09453 | -0.03941 | 0.00285 | 0.00009 | 0.00088 | 0 | 0.00003 | 0 | -0.00151 | 0.00695 | 0.13987 |
| **Central Kuroshio Current** | Warm Temperate Northwest Pacific | -0.03383 | -0.01250 | -0.01110 | -0.01111 | -0.00053 | -0.00024 | -0.00048 | 0 | -0.00010 | 0.00000 | -0.00240 | 0.01452 | -0.00979 |
| **Ross Sea** | Continental High Antarctic | -0.03736 | 0.00162 | 0 | 0.00005 | 0 | 0 | 0 | 0 | 0 | 0 | 0.00198 | -0.02964 | -0.03466 |
| **North Greenland** | Arctic | -0.04037 | -0.00926 | -0.00105 | -0.00025 | 0.00000 | 0 | 0.00002 | 0 | 0.00000 | 0 | 0 | -0.10090 | -0.00140 |
| **East Antarctic Dronning Maud Land** | Continental High Antarctic | -0.04144 | 0.00010 | 0 | 0 | 0 | 0 | 0 | 0 | 0 | 0 | 0.00001 | -0.10755 | 0.04266 |
| **Baltic Sea** | Northern European Seas | -0.04879 | -0.00826 | -0.05458 | -0.03664 | 0.00033 | 0.00025 | -0.00061 | 0 | 0.00090 | 0 | -0.02099 | 0.12106 | -0.04737 |
| **High Arctic Archipelago** | Arctic | -0.05270 | -0.00002 | 0.00000 | 0.00000 | 0 | 0 | 0.00001 | 0 | 0.00000 | 0 | 0 | -0.17691 | -0.01529 |
| **Cape Howe** | Southeast Australian Shelf | -0.05787 | -0.00060 | -0.00044 | -0.00017 | 0.00039 | 0.00000 | -0.00006 | 0.00005 | 0.00000 | -0.00001 | -0.00003 | 0.04225 | -0.09907 |
| **Beaufort Sea - continental coast and shelf** | Arctic | -0.06180 | -0.00001 | 0.0000 | 0 | -0.00009 | 0.00003 | 0.00069 | 0.00000 | 0.00007 | 0 | 0 | 0.21286 | -0.18824 |
| **Southern Red Sea** | Red Sea and Gulf of Aden | -0.06273 | -0.00854 | -0.00596 | -0.00379 | -0.00112 | 0.00022 | -0.00068 | 0 | -0.00057 | -0.00003 | -0.00017 | -0.02425 | -0.01813 |
| **Scotian Shelf** | Cold Temperate Northwest Atlantic | -0.06895 | -0.01935 | -0.01327 | -0.00769 | -0.00003 | -0.00021 | -0.00034 | 0 | -0.00001 | 0 | -0.00814 | 0.00303 | -0.02293 |
| **Easter Island** | Easter Island | -0.07138 | 0.01305 | 0.00789 | 0.00786 | 0.00000 | 0.00000 | 0.00001 | 0 | 0.00000 | 0 | 0.00836 | -0.11482 | 0.00625 |
| **Celtic Seas** | Northern European Seas | -0.07744 | -0.06656 | -0.03706 | -0.01622 | -0.00005 | -0.00012 | 0.00165 | 0 | 0.00014 | 0 | -0.01608 | 0.07458 | -0.01711 |
| **Arabian (Persian) Gulf** | Somali/Arabian | -0.07746 | -0.03136 | -0.02984 | -0.01597 | 0.00188 | 0.00007 | -0.00017 | -0.00066 | -0.00032 | -0.03867 | -0.00099 | 0.03154 | 0.00754 |
| **Southern Norway** | Northern European Seas | -0.07878 | -0.04282 | -0.04179 | -0.01851 | 0.00059 | 0.00190 | 0.00185 | -0.00025 | 0.00006 | 0 | -0.02717 | 0.01503 | 0.03277 |
| **Ionian Sea** | Mediterranean Sea | -0.08320 | 0.00090 | -0.00064 | 0.00158 | -0.00009 | -0.00001 | -0.00233 | 0 | -0.00043 | 0 | 0.00168 | 0.00999 | -0.09379 |
| **Namaqua** | Benguela | -0.09616 | -0.00808 | -0.00383 | -0.02884 | 0.00007 | -0.00001 | 0.00007 | 0 | -0.00002 | 0 | -0.00011 | -0.04748 | -0.00804 |
| **Central New Zealand** | Southern New Zealand | -0.10001 | -0.04568 | -0.01196 | -0.00489 | 0.00015 | -0.00003 | -0.00057 | 0 | -0.00120 | -0.00028 | -0.00020 | -0.05538 | 0.01975 |
| **Marshall Islands** | Marshall, Gilbert and Ellis Islands | -0.10043 | -0.00080 | -0.00184 | -0.00417 | 0.00002 | -0.00001 | 0 | 0 | 0 | 0 | -0.00707 | -0.10070 | 0.01414 |
| **Prince Edward Islands** | Subantarctic Islands | -0.11233 | -0.00204 | -0.00001 | -0.00001 | 0 | 0 | -0.00004 | 0 | -0.00001 | 0 | -0.00004 | -0.08544 | -0.02474 |
| **Puget Trough/Georgia Basin** | Cold Temperate Northeast Pacific | -0.11436 | -0.00998 | -0.00253 | -0.00752 | -0.00050 | 0.00041 | -0.00333 | 0 | -0.00040 | 0 | -0.00019 | -0.26875 | 0.13304 |
| **Weddell Sea** | Continental High Antarctic | -0.11522 | 0 | 0 | 0.00008 | 0 | 0 | 0 | 0 | 0 | 0 | 0 | -0.39340 | -0.00733 |
| **Snares Island** | Southern New Zealand | -0.12424 | -0.05980 | -0.00401 | -0.00264 | -0.00001 | 0 | 0 | 0 | 0 | -0.00010 | -0.00013 | -0.05541 | -0.00215 |
| **Malvinas/Falklands** | Magellanic | -0.12668 | -0.06800 | -0.00214 | -0.00334 | 0.00007 | 0.00001 | 0 | 0 | 0 | 0.00000 | -0.00001 | -0.08334 | 0.02990 |
| **Chatham Island** | Southern New Zealand | -0.12800 | -0.00852 | -0.00166 | -0.00147 | -0.00002 | 0.00000 | 0.00006 | 0 | 0.00000 | -0.00001 | -0.00018 | -0.08789 | -0.02840 |
| **Kara Sea** | Arctic | -0.13323 | -0.00020 | 0.00000 | -0.00007 | -0.00041 | 0.00002 | 0.00002 | 0.00000 | 0.00002 | 0 | 0 | -0.16935 | -0.00893 |
| **Northern Norway and Finnmark** | Northern European Seas | -0.14536 | -0.04481 | -0.04232 | -0.01901 | 0.00051 | 0.00180 | -0.00039 | 0 | 0.00000 | 0 | -0.02150 | -0.02524 | 0.00501 |
| **South and West Iceland** | Northern European Seas | -0.14692 | -0.08133 | -0.04880 | -0.03103 | -0.00025 | 0.00002 | 0.00004 | 0 | 0.00000 | 0 | -0.01582 | 0.12117 | -0.09029 |
| **Gilbert/Ellis Islands** | Marshall, Gilbert and Ellis Islands | -0.15359 | -0.00525 | -0.00542 | -0.00910 | -0.00003 | 0.00000 | 0 | 0 | 0 | -0.00008 | -0.01094 | -0.13009 | 0.00726 |
| **East Siberian Sea** | Arctic | -0.15578 | -0.00002 | 0.00000 | 0 | -0.00009 | 0.00000 | 0.00000 | 0 | 0.00000 | 0 | 0 | -0.22089 | 0.04903 |
| **Hawaii** | Hawaii | -0.16155 | -0.00030 | -0.00032 | -0.00080 | -0.00001 | -0.00001 | 0.00009 | 0 | 0.00002 | -0.00011 | -0.00068 | -0.22015 | 0.06061 |
| **North Sea** | Northern European Seas | -0.16930 | -0.08286 | -0.06221 | -0.03064 | 0.00025 | -0.00016 | -0.00127 | -0.00017 | 0.00016 | 0 | -0.02521 | 0.02934 | 0.00380 |
| **Aleutian Islands** | Cold Temperate Northeast Pacific | -0.17847 | -0.00775 | -0.00161 | -0.00301 | 0.00012 | 0.00001 | 0.00002 | 0 | 0.00000 | 0 | -0.00012 | -0.13234 | -0.03420 |
| **North and East Barents Sea** | Arctic | -0.18459 | -0.06152 | -0.05138 | -0.01543 | 0.00003 | 0.00000 | 0.00000 | 0 | 0.00000 | 0 | -0.00121 | -0.06509 | 0.00034 |
| **Line Islands** | Central Polynesia | -0.19104 | -0.00022 | -0.00019 | -0.00129 | 0.00001 | 0.00000 | 0.00000 | 0 | 0.00000 | -0.00011 | -0.00172 | -0.21223 | 0.02468 |
| **Bassian** | Southeast Australian Shelf | -0.19462 | -0.00047 | -0.00026 | -0.00008 | 0.00007 | 0.00001 | -0.00022 | 0.00000 | -0.00004 | 0.00000 | -0.00001 | -0.14659 | -0.04770 |
| **North and East Iceland** | Arctic | -0.20421 | -0.05677 | -0.02972 | -0.02897 | -0.00001 | 0.00002 | -0.00001 | 0 | 0.00000 | 0 | -0.01086 | -0.09326 | 0.01525 |
| **Macquarie Island** | Subantarctic Islands | -0.21165 | 0.00000 | 0.00000 | 0.00000 | 0.00002 | 0 | 0.00001 | 0 | 0.00000 | 0 | 0.00000 | -0.22987 | 0.01816 |
| **Campbell Island** | Subantarctic New Zealand | -0.22852 | -0.07461 | -0.00036 | -0.00270 | -0.00012 | 0 | 0.00000 | 0 | 0.00000 | 0 | -0.00006 | -0.14503 | -0.00567 |
| **South New Zealand** | Southern New Zealand | -0.23112 | -0.04133 | -0.00551 | -0.00208 | -0.00032 | -0.00002 | -0.00045 | 0 | -0.00034 | -0.00015 | -0.00015 | -0.15357 | -0.02798 |
| **East Greenland Shelf** | Arctic | -0.24699 | -0.01911 | -0.00970 | -0.01058 | 0.00029 | 0.00000 | 0 | 0 | 0 | 0 | -0.00070 | -0.27260 | 0.04573 |
| **Faroe Plateau** | Northern European Seas | -0.31021 | -0.11629 | -0.04955 | -0.02382 | 0.00001 | 0.00010 | 0 | 0 | 0 | 0 | -0.03645 | -0.05822 | -0.02606 |
| **Adriatic Sea** | Mediterranean Sea | -0.33396 | -0.03678 | -0.02177 | -0.02482 | -0.00029 | -0.00004 | -0.00620 | 0 | -0.00170 | 0 | -0.00189 | -0.21463 | -0.02999 |
| **Bermuda** | Tropical Northwestern Atlantic | -0.37306 | -0.00015 | -0.00017 | -0.00049 | 0.00000 | 0.00000 | 0 | 0 | 0 | -0.00004 | -0.00024 | -0.35834 | -0.01364 |
| **Patagonian Shelf** | Magellanic | -0.38024 | -0.09635 | -0.02737 | -0.01274 | -0.00008 | -0.00001 | 0.00011 | 0.00000 | 0.00000 | -0.00003 | -0.00010 | -0.26631 | 0.02186 |
| **Crozet Islands** | Subantarctic Islands | -0.52573 | -0.00060 | -0.00002 | -0.00001 | 0 | 0.00000 | 0 | 0 | 0 | 0 | -0.00002 | -0.51278 | -0.01251 |
| **Northern California** | Cold Temperate Northeast Pacific | -0.56552 | -0.00305 | -0.00229 | -0.00105 | -0.00013 | -0.00005 | 0.00000 | 0 | 0.00002 | 0 | -0.00002 | -0.58458 | 0.02380 |
| **Gulf of Alaska** | Cold Temperate Northeast Pacific | -0.80624 | -0.02500 | -0.00471 | -0.00783 | -0.00011 | 0.00004 | -0.00027 | -0.00001 | -0.00001 | 0 | -0.00020 | -0.81107 | 0.03334 |
| **Oregon, Washington, Vancouver Coast and Shelf** | Cold Temperate Northeast Pacific | -0.83502 | -0.00301 | -0.00073 | -0.00355 | -0.00015 | -0.00003 | -0.00040 | 0 | -0.00015 | 0 | -0.00019 | -0.88110 | 0.04934 |
| **Eastern Bering Sea** | Arctic | -0.85442 | -0.06561 | -0.01984 | -0.00927 | -0.00018 | 0.00001 | -0.00023 | 0 | 0.00001 | 0 | -0.00087 | -0.77722 | 0.01374 |
| **North American Pacific Fijordland** | Cold Temperate Northeast Pacific | -0.88403 | -0.01309 | -0.00267 | -0.00534 | -0.00018 | 0.00013 | -0.00035 | 0 | -0.00001 | 0 | -0.00037 | -0.90734 | 0.01266 |
