# Supplementary material for: Spatial and temporal changes in cumulative human impacts on the world's ocean
Source: Nat Commun. 2015 Jul 14;6:7615. doi: 10.1038/ncomms8615 (PMC4510691; doi:10.1038/ncomms8615)
Supplement: Supplementary Data 5 — Average impact scores for each stressor and for cumulative impact in 2013 for each FAO high seas region, in decreasing order of average cumulative impact. True zero values are indicated by zeros with no trailing decimals; very small values are zeros with several zero decimal values. [file ncomms8615-s6.doc]

## *Supplementary Data 5*

Average impact scores for each stressor and for cumulative impact in 2013 for each FAO high seas region, in decreasing order of average cumulative impact. True zero values are indicated by zeros with no trailing decimals; very small values are zeros with several zero decimal values.

| **Suppl. Data 5: FAO**  **High Seas region** | **Average cumulative impact score** | **Artisanal fishing** | **Demersal destructive fishing** | **Demersal nondestructive high bycatch fishing** | **Demersal nondestructive low bycatch fishing** | **Direct human impact** | **Inorganic pollution** | **Invasive species** | **Light pollution** | **Nutrient pollution** | **Ocean acidification** | **Ocean-based pollution** | **Oil rigs** | **Organic pollution** | **Pelagic high bycatch fishing** | **Pelagic low bycatch fishing** | **Sea level rise** | **Sea surface temperature** | **Shipping** | **UV** |
| --- | --- | --- | --- | --- | --- | --- | --- | --- | --- | --- | --- | --- | --- | --- | --- | --- | --- | --- | --- | --- |
| **Atlantic, Western-Central** | 4.2166 | 0 | 0.0034 | 0.0027 | 0.0092 | 0 | 0 | 0 | 0 | 0 | 1.2510 | 0.2501 | 0 | 0 | 0.0048 | 0.0054 | 0 | 1.7995 | 0.1474 | 0.7431 |
| **Atlantic, Northeast** | 4.2000 | 0 | 0.0171 | 0.0011 | 0.0027 | 0 | 0 | 0 | 0 | 0 | 1.1259 | 0.3098 | 0 | 0 | 0 | 0.0007 | 0.0000 | 1.8621 | 0.1961 | 0.7300 |
| **Pacific, Northwest** | 4.1766 | 0 | 0.0126 | 0.0635 | 0.0603 | 0 | 0 | 0 | 0 | 0 | 1.0901 | 0.2763 | 0 | 0 | 0.0001 | 0.0165 | 0 | 1.7209 | 0.1601 | 0.7769 |
| **Pacific, Western Central** | 4.1182 | 0 | 0.0196 | 0.1028 | 0.1461 | 0 | 0 | 0 | 0 | 0 | 1.1483 | 0.1325 | 0 | 0 | 0.0002 | 0.1984 | 0.0000 | 1.6024 | 0.0749 | 0.6936 |
| **Atlantic, Eastern Central** | 4.1043 | 0 | 0.0012 | 0.0010 | 0.0178 | 0 | 0 | 0 | 0 | 0 | 1.1680 | 0.1493 | 0 | 0 | 0.0042 | 0.0243 | 0.0000 | 1.9112 | 0.0855 | 0.7417 |
| **Atlantic, Northwest** | 3.8868 | 0 | 0.0166 | 0.0041 | 0.0025 | 0 | 0 | 0 | 0 | 0 | 1.1378 | 0.3439 | 0 | 0 | 0 | 0.0013 | 0.0016 | 1.3511 | 0.2097 | 0.8184 |
| **Indian Ocean, Western** | 3.6889 | 0 | 0.0095 | 0.0085 | 0.0171 | 0 | 0 | 0 | 0 | 0 | 1.1460 | 0.0872 | 0 | 0 | 0.0022 | 0.0238 | 0.0005 | 1.6015 | 0.0493 | 0.7433 |
| **Atlantic, Southwest** | 3.6342 | 0 | 0.0099 | 0.0037 | 0.0029 | 0 | 0 | 0 | 0 | 0 | 1.0923 | 0.0578 | 0 | 0 | 0.0049 | 0.0031 | 0.0001 | 1.6333 | 0.0315 | 0.7946 |
| **Pacific, Southwest** | 3.4396 | 0 | 0.0024 | 0.0022 | 0.0011 | 0 | 0 | 0 | 0 | 0 | 1.0185 | 0.0435 | 0 | 0 | 0.0000 | 0.0010 | 0.0000 | 1.5320 | 0.0240 | 0.8149 |
| **Pacific, Northeast** | 3.4203 | 0 | 0.0018 | 0.0023 | 0.0032 | 0 | 0 | 0 | 0 | 0 | 0.9290 | 0.3351 | 0 | 0 | 0 | 0.0026 | 0 | 1.1133 | 0.1970 | 0.8359 |
| **Indian Ocean, Eastern** | 3.2646 | 0 | 0.0036 | 0.0136 | 0.0129 | 0 | 0 | 0 | 0 | 0 | 1.0428 | 0.0571 | 0 | 0 | 0.0059 | 0.0151 | 0.0000 | 1.2957 | 0.0313 | 0.7866 |
| **Atlantic, Southeast** | 3.2373 | 0 | 0.0002 | 0.0002 | 0.0030 | 0 | 0 | 0 | 0 | 0 | 1.0300 | 0.0686 | 0 | 0 | 0.0000 | 0.0020 | 0.0001 | 1.3064 | 0.0379 | 0.7889 |
| **Pacific, Southeast** | 2.9156 | 0 | 0.0029 | 0.0311 | 0.0372 | 0 | 0 | 0 | 0 | 0 | 0.9613 | 0.0448 | 0 | 0 | 0.0006 | 0.0122 | 0.0000 | 1.0278 | 0.0244 | 0.7732 |
| **Pacific, Eastern Central** | 2.8132 | 0 | 0.0012 | 0.0058 | 0.0087 | 0 | 0 | 0 | 0 | 0 | 1.0827 | 0.1517 | 0 | 0 | 0.0055 | 0.0119 | 0 | 0.7274 | 0.0862 | 0.7321 |
| **Indian Ocean, Antarctic And Southern** | 2.4525 | 0 | 0.0007 | 0.0000 | 0.0000 | 0 | 0 | 0 | 0 | 0 | 0.7207 | 0.0164 | 0 | 0 | 0.0000 | 0.0000 | 0.0000 | 0.9065 | 0.0089 | 0.7991 |
| **Atlantic, Antarctic** | 2.2736 | 0 | 0.0011 | 0.0000 | 0.0049 | 0 | 0 | 0 | 0 | 0 | 0.7359 | 0.0152 | 0 | 0 | 0 | 0.0001 | 0 | 0.8302 | 0.0084 | 0.7202 |
| **Pacific, Antarctic** | 2.1857 | 0 | 0.0021 | 0 | 0.0000 | 0 | 0 | 0 | 0 | 0 | 0.7035 | 0.0110 | 0 | 0 | 0 | 0.0029 | 0.0000 | 0.7321 | 0.0060 | 0.7502 |
| **Arctic Sea** | 0.7431 | 0 | 0.0000 | 0.0000 | 0 | 0 | 0 | 0 | 0 | 0 | 0.7085 | 0.0014 | 0 | 0 | 0 | 0 | 0.0000 | 0.5933 | 0.0008 | 0.0149 |
