# Supplementary material for: Spatial and temporal changes in cumulative human impacts on the world's ocean
Source: Nat Commun. 2015 Jul 14;6:7615. doi: 10.1038/ncomms8615 (PMC4510691; doi:10.1038/ncomms8615)
Supplement: Supplementary Data 6 — Average difference in impact scores for each stressor and for cumulative impact between 2013 and 2008 for each FAO high seas region. Differences could only be calculated for the 12 (of 19) stressor layers that had data for both time. True zero values are indicated by zeros with no trailing decimals; very small values are zeros with several zero decimal values. [file ncomms8615-s7.doc]

## *Supplementary Data 6*

Average difference in impact scores for each stressor and for cumulative impact between 2013 and 2008 for each FAO high seas region. Differences could only be calculated for the 12 (of 19) stressor layers that had data for both time. True zero values are indicated by zeros with no trailing decimals; very small values are zeros with several zero decimal values.

| **Suppl. Data 6: 2013 minus 2008 FAO**  **High Seas region** | **Average cumulative impact scores** | **Demersal destructive fishing** | **Demersal nondestructive high bycatch fishing** | **Demersal nondestructive low bycatch fishing** | **Direct human impact** | **Light pollution** | **Nutrient pollution** | **Oil rigs** | **Organic pollution** | **Pelagic high bycatch fishing** | **Pelagic low bycatch fishing** | **Sea surface temperature** | **UV** |
| --- | --- | --- | --- | --- | --- | --- | --- | --- | --- | --- | --- | --- | --- |
| **Atlantic, Southeast** | 0.57803 | -0.00009 | -0.00011 | -0.00154 | 0 | 0 | 0 | 0 | 0 | 0.00000 | -0.00121 | 0.58758 | -0.00660 |
| **Atlantic, Eastern Central** | 0.48168 | 0.00006 | 0.00005 | 0.00084 | 0 | 0 | 0 | 0 | 0 | 0.00022 | 0.00119 | 0.47469 | 0.00464 |
| **Indian Ocean, Western** | 0.47253 | -0.00462 | -0.00435 | -0.00798 | 0 | 0 | 0 | 0 | 0 | -0.00122 | -0.01199 | 0.50342 | -0.00074 |
| **Pacific, Southeast** | 0.43075 | -0.00089 | -0.01005 | -0.01010 | 0 | 0 | 0 | 0 | 0 | -0.00023 | -0.00433 | 0.46893 | -0.01258 |
| **Atlantic, Southwest** | 0.41183 | -0.00955 | -0.00418 | -0.00374 | 0 | 0 | 0 | 0 | 0 | -0.00669 | -0.00428 | 0.43990 | 0.00037 |
| **Indian Ocean, Eastern** | 0.31458 | -0.00044 | -0.00161 | -0.00145 | 0 | 0 | 0 | 0 | 0 | -0.00072 | -0.00180 | 0.31226 | 0.00835 |
| **Pacific, Northwest** | 0.30702 | -0.00335 | -0.01593 | -0.01354 | 0 | 0 | 0 | 0 | 0 | -0.00003 | -0.00485 | 0.32369 | 0.02116 |
| **Pacific, Southwest** | 0.18765 | -0.00151 | -0.00161 | -0.00089 | 0 | 0 | 0 | 0 | 0 | 0.00000 | -0.00085 | 0.18968 | 0.00285 |
| **Atlantic, Antarctic** | 0.18012 | 0.00029 | 0.00000 | 0.00109 | 0 | 0 | 0 | 0 | 0 | 0 | 0.00001 | 0.19108 | -0.00260 |
| **Pacific, Western Central** | 0.14476 | -0.00133 | -0.00609 | -0.00623 | 0 | 0 | 0 | 0 | 0 | -0.00001 | -0.01077 | 0.16382 | 0.00543 |
| **Atlantic, Northeast** | 0.09167 | -0.02148 | -0.00189 | -0.00456 | 0 | 0 | 0 | 0 | 0 | 0 | -0.00144 | 0.12311 | 0.00093 |
| **Pacific, Eastern Central** | 0.06779 | -0.00061 | -0.00269 | -0.00422 | 0 | 0 | 0 | 0 | 0 | -0.00279 | -0.00593 | 0.07419 | 0.00984 |
| **Atlantic, Western-Central** | 0.04933 | -0.00201 | -0.00158 | -0.00518 | 0 | 0 | 0 | 0 | 0 | -0.00294 | -0.00328 | 0.03865 | 0.02568 |
| **Arctic Sea** | -0.00145 | -0.00014 | 0.00000 | 0 | 0 | 0 | 0 | 0 | 0 | 0 | 0 | -0.38303 | 0.00989 |
| **Indian Ocean, Antarctic And Southern** | -0.03278 | 0.00021 | 0.00000 | 0.00000 | 0 | 0 | 0 | 0 | 0 | 0.00000 | 0.00000 | -0.02180 | -0.01120 |
| **Pacific, Antarctic** | -0.07969 | 0.00133 | 0 | 0.00003 | 0 | 0 | 0 | 0 | 0 | 0 | 0.00183 | -0.07290 | -0.01217 |
| **Atlantic, Northwest** | -0.07987 | -0.00366 | -0.00132 | -0.00082 | 0 | 0 | 0 | 0 | 0 | 0 | -0.00048 | -0.06355 | -0.01003 |
| **Pacific, Northeast** | -0.21884 | 0.00005 | 0.00006 | 0.00009 | 0 | 0 | 0 | 0 | 0 | 0 | 0.00007 | -0.22001 | 0.00090 |
