# Supplementary material for: Spatial and temporal changes in cumulative human impacts on the world's ocean
Source: Nat Commun. 2015 Jul 14;6:7615. doi: 10.1038/ncomms8615 (PMC4510691; doi:10.1038/ncomms8615)
Supplement: Supplementary Data 7 — Average impact scores for each stressor and for cumulative impact in 2013 for each large marine ecosystem (LME), in decreasing order of average cumulative impact. True zero values are indicated by zeros with no trailing decimals; very small values are zeros with several zero decimal values. [file ncomms8615-s8.doc]

## *Supplementary Data 7*

Average impact scores for each stressor and for cumulative impact in 2013 for each large marine ecosystem (LME), in decreasing order of average cumulative impact. True zero values are indicated by zeros with no trailing decimals; very small values are zeros with several zero decimal values.

| **Suppl. Data 7: 2013 LME**  **LME** | **Average cumulative impact score** | **Artisanal fishing** | **Demersal destructive fishing** | **Demersal nondestructive high bycatch fishing** | **Demersal nondestructive low bycatch fishing** | **Direct human impact** | **Inorganic pollution** | **Invasive species** | **Light pollution** | **Nutrient pollution** | **Ocean acidification** | **Ocean-based pollution** | **Oil rigs** | **Organic pollution** | **Pelagic high bycatch fishing** | **Pelagic low bycatch fishing** | **Sea level rise** | **Sea surface temperature** | **Shipping** | **UV** |
| --- | --- | --- | --- | --- | --- | --- | --- | --- | --- | --- | --- | --- | --- | --- | --- | --- | --- | --- | --- | --- |
| **East China Sea** | 5.2771 | 0.0014 | 0.5644 | 0.4816 | 0.1671 | 0.0274 | 0.0178 | 0.0277 | 0.0055 | 0.0193 | 0.7854 | 0.4021 | 0.0000 | 0.0030 | 0.0210 | 0.0115 | 0.2520 | 1.4818 | 0.4295 | 0.5872 |
| **North Sea** | 4.9388 | 0.0019 | 0.3841 | 0.1987 | 0.1020 | 0.0274 | 0.0250 | 0.1007 | 0.0054 | 0.0289 | 0.6240 | 0.4724 | 0.0004 | 0.0041 | 0 | 0.0901 | 0.3649 | 1.4732 | 0.4548 | 0.5946 |
| **Faroe Plateau** | 4.8104 | 0.0004 | 0.3605 | 0.0438 | 0.0175 | 0.0063 | 0.0010 | 0.0163 | 0.0006 | 0 | 1.0793 | 0.2731 | 0 | 0 | 0 | 0.0119 | 0.0242 | 2.1704 | 0.2476 | 0.5604 |
| **Yellow Sea** | 4.7979 | 0.0009 | 0.4583 | 0.6100 | 0.2135 | 0.0353 | 0.0481 | 0.0630 | 0.0065 | 0.0676 | 0.3833 | 0.4427 | 0.0003 | 0.0141 | 0 | 0.0034 | 0.6415 | 0.9941 | 0.3778 | 0.4459 |
| **Celtic-Biscay Shelf** | 4.6649 | 0.0028 | 0.1976 | 0.1267 | 0.0615 | 0.0243 | 0.0188 | 0.0614 | 0.0042 | 0.0186 | 0.8806 | 0.3394 | 0 | 0.0046 | 0 | 0.0422 | 0.1379 | 1.6972 | 0.4057 | 0.6557 |
| **Canary Current** | 4.6440 | 0.0003 | 0.0718 | 0.1030 | 0.1182 | 0.0056 | 0.0077 | 0.0072 | 0.0006 | 0.0041 | 1.0516 | 0.4052 | 0.0000 | 0.0008 | 0.0052 | 0.0064 | 0.0730 | 1.8326 | 0.2906 | 0.6646 |
| **Iberian Coastal** | 4.5780 | 0.0011 | 0.1381 | 0.0890 | 0.0905 | 0.0153 | 0.0277 | 0.0315 | 0.0044 | 0.0180 | 1.0395 | 0.5060 | 0 | 0.0059 | 0 | 0.0265 | 0.0519 | 1.4890 | 0.4089 | 0.6411 |
| **Mediterranean Sea** | 4.5487 | 0.0016 | 0.0735 | 0.0676 | 0.0563 | 0.0204 | 0.0296 | 0.0380 | 0.0043 | 0.0196 | 1.0631 | 0.4812 | 0.0003 | 0.0049 | 0.0019 | 0.0115 | 0.0829 | 1.6705 | 0.3840 | 0.5488 |
| **Black Sea** | 4.5408 | 0.0018 | 0.0128 | 0.0119 | 0.0579 | 0.0166 | 0.0365 | 0.0412 | 0.0022 | 0.0321 | 0.9748 | 0.4420 | 0.0001 | 0.0094 | 0.0001 | 0.0030 | 0.1770 | 1.8563 | 0.3392 | 0.5350 |
| **South China Sea** | 4.4507 | 0.0012 | 0.3394 | 0.3173 | 0.1338 | 0.0115 | 0.0109 | 0.0146 | 0.0013 | 0.0160 | 0.8982 | 0.3069 | 0.0011 | 0.0022 | 0.0106 | 0.0304 | 0.2456 | 1.3512 | 0.2451 | 0.5172 |
| **Norwegian Sea** | 4.4286 | 0.0004 | 0.1412 | 0.1216 | 0.0612 | 0.0065 | 0.0014 | 0.0076 | 0.0011 | 0.0005 | 1.0722 | 0.2348 | 0.0001 | 0.0000 | 0 | 0.0810 | 0.0233 | 1.9140 | 0.1690 | 0.5985 |
| **Iceland Shelf and Sea** | 4.4107 | 0.0007 | 0.2419 | 0.1993 | 0.1535 | 0.0050 | 0.0014 | 0.0111 | 0.0006 | 0.0016 | 0.9522 | 0.2105 | 0 | 0.0000 | 0 | 0.0541 | 0.0381 | 1.8803 | 0.1995 | 0.4685 |
| **Kuroshio Current** | 4.3262 | 0.0014 | 0.1098 | 0.0882 | 0.0766 | 0.0109 | 0.0092 | 0.0237 | 0.0021 | 0.0061 | 1.1009 | 0.3947 | 0 | 0.0020 | 0.0108 | 0.0186 | 0.0345 | 1.5417 | 0.2710 | 0.6282 |
| **Sulu-Celebes Sea** | 4.3054 | 0.0141 | 0.2197 | 0.3044 | 0.1476 | 0.0355 | 0.0214 | 0.0216 | 0.0011 | 0.0130 | 1.0653 | 0.1633 | 0.0000 | 0.0016 | 0.0025 | 0.0510 | 0.1747 | 1.4852 | 0.1133 | 0.4817 |
| **Caribbean Sea** | 4.2290 | 0.0020 | 0.0329 | 0.0230 | 0.0145 | 0.0096 | 0.0097 | 0.0148 | 0.0012 | 0.0063 | 1.1122 | 0.3337 | 0.0000 | 0.0019 | 0.0033 | 0.0010 | 0.0775 | 1.8403 | 0.2259 | 0.5250 |
| **East Brazil Shelf** | 4.1414 | 0.0012 | 0.1074 | 0.0491 | 0.0302 | 0.0044 | 0.0133 | 0.0126 | 0.0007 | 0.0154 | 1.0462 | 0.3041 | 0.0007 | 0.0017 | 0.0740 | 0.0137 | 0.1030 | 1.5751 | 0.1928 | 0.5988 |
| **Arabian Sea** | 4.1312 | 0.0004 | 0.0849 | 0.0563 | 0.0406 | 0.0053 | 0.0085 | 0.0167 | 0.0010 | 0.0071 | 1.0018 | 0.3129 | 0.0012 | 0.0005 | 0.0219 | 0.0181 | 0.0841 | 1.6531 | 0.2084 | 0.6116 |
| **Guinea Current** | 4.0820 | 0.0010 | 0.0568 | 0.0623 | 0.0369 | 0.0040 | 0.0082 | 0.0140 | 0.0002 | 0.0029 | 1.0430 | 0.2255 | 0.0131 | 0.0007 | 0.1079 | 0.0084 | 0.0957 | 1.6840 | 0.1504 | 0.5702 |
| **East Central Australian Shelf** | 4.0792 | 0.0018 | 0.0191 | 0.0081 | 0.0128 | 0.0055 | 0.0054 | 0.0024 | 0.0006 | 0.0047 | 1.1295 | 0.2718 | 0 | 0.0005 | 0.0004 | 0.0128 | 0.0455 | 1.6572 | 0.1812 | 0.7225 |
| **Gulf of Thailand** | 4.0638 | 0.0022 | 0.4354 | 0.3459 | 0.1638 | 0.0203 | 0.0246 | 0.0398 | 0.0026 | 0.0352 | 0.4884 | 0.2544 | 0.0006 | 0.0069 | 0.0001 | 0.0043 | 0.6630 | 1.0060 | 0.1923 | 0.3839 |
| **Scotian Shelf** | 4.0411 | 0.0031 | 0.1750 | 0.1657 | 0.0761 | 0.0200 | 0.0090 | 0.0499 | 0.0041 | 0.0046 | 0.5557 | 0.2655 | 0 | 0.0003 | 0 | 0.0476 | 0.2324 | 1.5825 | 0.3046 | 0.5567 |
| **Bay of Bengal** | 4.0213 | 0.0009 | 0.1476 | 0.1178 | 0.0716 | 0.0090 | 0.0117 | 0.0075 | 0.0005 | 0.0138 | 0.9911 | 0.1243 | 0.0000 | 0.0014 | 0.1008 | 0.0290 | 0.1056 | 1.6019 | 0.0781 | 0.6127 |
| **Sea of Japan** | 3.9253 | 0.0012 | 0.1920 | 0.0826 | 0.0871 | 0.0118 | 0.0119 | 0.0230 | 0.0020 | 0.0081 | 0.8515 | 0.2698 | 0 | 0.0028 | 0 | 0.0044 | 0.0496 | 1.5889 | 0.1910 | 0.5543 |
| **Labrador - Newfoundland** | 3.9077 | 0.0021 | 0.2384 | 0.1129 | 0.0376 | 0.0102 | 0.0045 | 0.0156 | 0.0017 | 0.0021 | 0.6759 | 0.2221 | 0.0001 | 0.0003 | 0 | 0.0222 | 0.1019 | 1.6157 | 0.2460 | 0.6129 |
| **South Brazil Shelf** | 3.9070 | 0.0010 | 0.0831 | 0.0486 | 0.0164 | 0.0089 | 0.0088 | 0.0248 | 0.0016 | 0.0099 | 0.9477 | 0.2710 | 0.0018 | 0.0011 | 0.0140 | 0.0111 | 0.1338 | 1.4741 | 0.2528 | 0.6012 |
| **North Brazil Shelf** | 3.8800 | 0.0002 | 0.0750 | 0.0400 | 0.0228 | 0.0091 | 0.0073 | 0.0069 | 0.0004 | 0.0101 | 0.8585 | 0.2008 | 0.0001 | 0.0015 | 0.0278 | 0.0025 | 0.3049 | 1.6472 | 0.1282 | 0.5436 |
| **West Central Australian Shelf** | 3.8751 | 0.0001 | 0.0090 | 0.0117 | 0.0034 | 0.0020 | 0.0008 | 0.0066 | 0.0001 | 0.0030 | 1.0303 | 0.2409 | 0 | 0.0005 | 0.0289 | 0.0014 | 0.1218 | 1.5502 | 0.1698 | 0.6977 |
| **Agulhas Current** | 3.8436 | 0.0007 | 0.0396 | 0.0089 | 0.0170 | 0.0055 | 0.0080 | 0.0049 | 0.0002 | 0.0027 | 1.0958 | 0.1566 | 0.0000 | 0.0004 | 0.0033 | 0.0054 | 0.0617 | 1.6932 | 0.1013 | 0.6424 |
| **Gulf of Mexico** | 3.8248 | 0.0005 | 0.0828 | 0.0462 | 0.0427 | 0.0073 | 0.0124 | 0.0211 | 0.0016 | 0.0152 | 0.9196 | 0.2868 | 0.0011 | 0.0038 | 0.0116 | 0.0008 | 0.2228 | 1.4236 | 0.1928 | 0.5364 |
| **Indonesian Sea** | 3.8085 | 0.0060 | 0.2607 | 0.1806 | 0.1435 | 0.0220 | 0.0174 | 0.0133 | 0.0006 | 0.0156 | 0.9069 | 0.1398 | 0.0004 | 0.0018 | 0.0026 | 0.0405 | 0.3120 | 1.1959 | 0.0931 | 0.4624 |
| **Northeast U.S. Continental Shelf** | 3.8010 | 0.0045 | 0.2817 | 0.1144 | 0.0827 | 0.0448 | 0.0285 | 0.0863 | 0.0091 | 0.0189 | 0.5342 | 0.3009 | 0 | 0.0040 | 0 | 0.0233 | 0.3179 | 1.1698 | 0.3184 | 0.4783 |
| **South West Australian Shelf** | 3.7697 | 0.0002 | 0.0192 | 0.0209 | 0.0041 | 0.0025 | 0.0007 | 0.0076 | 0.0001 | 0.0048 | 0.9357 | 0.1377 | 0 | 0.0005 | 0.0005 | 0.0010 | 0.1465 | 1.7182 | 0.0908 | 0.6825 |
| **Baltic Sea** | 3.7576 | 0.0033 | 0.0198 | 0.1401 | 0.1123 | 0.0669 | 0.0562 | 0.2345 | 0.0101 | 0.0537 | 0.0126 | 0.4732 | 0 | 0.0059 | 0 | 0.0467 | 0.7262 | 1.0462 | 0.3668 | 0.4044 |
| **Benguela Current** | 3.6987 | 0.0002 | 0.0422 | 0.0251 | 0.0666 | 0.0021 | 0.0047 | 0.0041 | 0.0002 | 0.0013 | 1.0483 | 0.1700 | 0.0010 | 0.0003 | 0.0000 | 0.0008 | 0.0223 | 1.5483 | 0.1235 | 0.6398 |
| **Northwest Australian Shelf** | 3.6911 | 0.0001 | 0.0447 | 0.0248 | 0.0099 | 0.0018 | 0.0003 | 0.0069 | 0.0001 | 0.0013 | 0.9345 | 0.2053 | 0.0002 | 0.0001 | 0.0203 | 0.0019 | 0.2272 | 1.5005 | 0.1609 | 0.5550 |
| **Red Sea** | 3.6423 | 0.0019 | 0.0883 | 0.0531 | 0.0254 | 0.0201 | 0.0156 | 0.0354 | 0.0028 | 0.0055 | 0.9517 | 0.2691 | 0.0004 | 0.0003 | 0.0005 | 0.0006 | 0.3159 | 1.3900 | 0.2185 | 0.2607 |
| **Southeast Australian Shelf** | 3.5414 | 0.0004 | 0.0139 | 0.0097 | 0.0026 | 0.0040 | 0.0015 | 0.0071 | 0.0002 | 0.0033 | 0.9823 | 0.1317 | 0.0001 | 0.0005 | 0.0002 | 0.0006 | 0.0746 | 1.4999 | 0.0937 | 0.7182 |
| **Insular Pacific-Hawaiian** | 3.5221 | 0.0004 | 0.0029 | 0.0018 | 0.0056 | 0.0014 | 0.0013 | 0.0014 | 0.0003 | 0.0014 | 1.1960 | 0.1901 | 0 | 0.0002 | 0.0004 | 0.0011 | 0.0058 | 1.3281 | 0.1184 | 0.6667 |
| **West Bering Sea** | 3.4665 | 0.0001 | 0.0773 | 0.0174 | 0.0608 | 0.0023 | 0.0006 | 0.0005 | 0.0001 | 0.0003 | 0.6576 | 0.1441 | 0 | 0.0000 | 0 | 0.0000 | 0.0234 | 1.7086 | 0.0880 | 0.6917 |
| **Somali Coastal Current** | 3.4494 | 0.0010 | 0.0210 | 0.0234 | 0.0072 | 0.0074 | 0.0098 | 0.0050 | 0.0002 | 0.0034 | 1.0383 | 0.0476 | 0 | 0.0005 | 0.0073 | 0.0024 | 0.0498 | 1.6806 | 0.0251 | 0.5251 |
| **Northeast Australian Shelf** | 3.4217 | 0.0005 | 0.0123 | 0.0062 | 0.0309 | 0.0025 | 0.0012 | 0.0069 | 0.0002 | 0.0031 | 0.9905 | 0.1927 | 0 | 0.0001 | 0.0019 | 0.0402 | 0.2606 | 1.2648 | 0.1197 | 0.4895 |
| **Southeast U.S. Continental Shelf** | 3.4143 | 0.0029 | 0.0881 | 0.0276 | 0.0279 | 0.0167 | 0.0256 | 0.0385 | 0.0034 | 0.0200 | 0.8817 | 0.3854 | 0 | 0.0024 | 0.0034 | 0.0004 | 0.2245 | 1.0541 | 0.2839 | 0.3334 |
| **Pacific Central-American Coastal** | 3.3728 | 0.0015 | 0.0566 | 0.0157 | 0.0350 | 0.0049 | 0.0084 | 0.0084 | 0.0003 | 0.0066 | 0.9756 | 0.2489 | 0 | 0.0028 | 0.0156 | 0.0071 | 0.0270 | 1.1561 | 0.1595 | 0.6454 |
| **Gulf of California** | 3.2609 | 0.0011 | 0.0745 | 0.0121 | 0.1461 | 0.0155 | 0.0168 | 0.0108 | 0.0010 | 0.0149 | 0.8946 | 0.0974 | 0 | 0.0067 | 0.0027 | 0.0040 | 0.1206 | 1.4010 | 0.0652 | 0.3891 |
| **Oyashio Current** | 3.2158 | 0.0002 | 0.0739 | 0.0317 | 0.0434 | 0.0025 | 0.0014 | 0.0017 | 0.0002 | 0.0019 | 0.7851 | 0.3430 | 0 | 0.0008 | 0 | 0.0060 | 0.0212 | 0.9171 | 0.2227 | 0.7646 |
| **Barents Sea** | 3.1651 | 0.0002 | 0.1718 | 0.1782 | 0.0517 | 0.0046 | 0.0021 | 0.0069 | 0.0008 | 0.0002 | 0.8319 | 0.0983 | 0 | 0.0000 | 0 | 0.0255 | 0.0800 | 1.3017 | 0.0936 | 0.4571 |
| **Sea of Okhotsk** | 3.1569 | 0.0002 | 0.4469 | 0.0937 | 0.1039 | 0.0028 | 0.0011 | 0.0021 | 0.0002 | 0.0010 | 0.6244 | 0.1324 | 0.0000 | 0.0002 | 0 | 0.0005 | 0.0675 | 1.0242 | 0.1009 | 0.5584 |
| **East Bering Sea** | 3.1060 | 0.0000 | 0.2189 | 0.0368 | 0.0375 | 0.0007 | 0.0010 | 0.0032 | 0.0001 | 0.0007 | 0.5836 | 0.1696 | 0 | 0.0001 | 0 | 0.0014 | 0.0627 | 1.1400 | 0.1258 | 0.7277 |
| **Aleutian Islands** | 3.0676 | 0.0000 | 0.0863 | 0.0099 | 0.0213 | 0.0005 | 0.0006 | 0.0051 | 0.0000 | 0.0013 | 0.6487 | 0.2200 | 0 | 0.0001 | 0 | 0.0004 | 0.0323 | 1.3050 | 0.1457 | 0.6009 |
| **Humboldt Current** | 3.0537 | 0.0007 | 0.0495 | 0.0306 | 0.1019 | 0.0056 | 0.0052 | 0.0039 | 0.0003 | 0.0035 | 0.9523 | 0.1530 | 0.0000 | 0.0010 | 0.0014 | 0.0025 | 0.0380 | 0.8966 | 0.0932 | 0.7199 |
| **Patagonian Shelf** | 2.9912 | 0.0001 | 0.2206 | 0.0437 | 0.0211 | 0.0042 | 0.0030 | 0.0110 | 0.0003 | 0.0045 | 0.7118 | 0.0603 | 0.0000 | 0.0015 | 0.0000 | 0.0002 | 0.1970 | 0.9782 | 0.0565 | 0.6804 |
| **California Current** | 2.9589 | 0.0003 | 0.0259 | 0.0151 | 0.0393 | 0.0030 | 0.0033 | 0.0045 | 0.0005 | 0.0016 | 0.9682 | 0.3757 | 0 | 0.0005 | 0.0032 | 0.0052 | 0.0101 | 0.5524 | 0.2487 | 0.7026 |
| **Gulf of Alaska** | 2.9512 | 0.0008 | 0.0524 | 0.0086 | 0.0194 | 0.0064 | 0.0033 | 0.0191 | 0.0010 | 0.0018 | 0.8148 | 0.2799 | 0.0000 | 0.0002 | 0 | 0.0011 | 0.0378 | 0.8005 | 0.2028 | 0.7118 |
| **New Zealand Shelf** | 2.7737 | 0.0005 | 0.1043 | 0.0267 | 0.0095 | 0.0076 | 0.0114 | 0.0090 | 0.0004 | 0.0088 | 1.0099 | 0.1464 | 0 | 0.0006 | 0.0004 | 0.0004 | 0.0650 | 0.7444 | 0.1168 | 0.5144 |
| **Greenland Sea** | 2.6811 | 0.0001 | 0.0514 | 0.0226 | 0.0385 | 0.0003 | 0 | 0.0003 | 0.0000 | 0 | 0.9273 | 0.0621 | 0 | 0 | 0 | 0.0019 | 0.0285 | 1.3579 | 0.0459 | 0.3591 |
| **Canadian Eastern Arctic - West Greenland** | 2.5781 | 0.0007 | 0.1564 | 0.0185 | 0.0032 | 0.0022 | 0.0000 | 0.0075 | 0.0001 | 0.0000 | 0.6052 | 0.0735 | 0 | 0.0000 | 0 | 0.0002 | 0.0650 | 1.2093 | 0.0551 | 0.4298 |
| **North Australian Shelf** | 2.5556 | 0.0001 | 0.0682 | 0.0331 | 0.0229 | 0.0033 | 0.0014 | 0.0094 | 0.0001 | 0.0086 | 0.5358 | 0.0938 | 0 | 0.0003 | 0.0028 | 0.0134 | 0.6506 | 0.5166 | 0.0832 | 0.5150 |
| **Hudson Bay Complex** | 2.3537 | 0.0001 | 0.0005 | 0.0000 | 0.0000 | 0.0013 | 0.0013 | 0.0028 | 0.0001 | 0.0024 | 0.3245 | 0.0333 | 0 | 0.0005 | 0 | 0.0000 | 0.2754 | 1.1027 | 0.0296 | 0.5907 |
| **Northern Bering - Chukchi Seas** | 1.9368 | 0.0000 | 0.1142 | 0.0219 | 0.0147 | 0.0016 | 0.0006 | 0.0032 | 0.0002 | 0.0009 | 0.4674 | 0.0337 | 0 | 0.0001 | 0 | 0.0000 | 0.1743 | 0.9251 | 0.0256 | 0.3654 |
| **Kara Sea** | 1.5748 | 0.0000 | 0.0011 | 0.0000 | 0.0000 | 0.0030 | 0.0012 | 0.0013 | 0.0001 | 0.0004 | 0.4941 | 0.0185 | 0.0000 | 0.0001 | 0 | 0 | 0.2435 | 0.6584 | 0.0122 | 0.2982 |
| **East Siberian Sea** | 1.0204 | 0.0000 | 0.0000 | 0.0000 | 0 | 0.0005 | 0.0006 | 0.0013 | 0.0000 | 0.0000 | 0.3592 | 0.0014 | 0 | 0.0000 | 0 | 0 | 0 | 0.3072 | 0.0007 | 0.3746 |
| **Beaufort Sea** | 0.9386 | 0.0000 | 0.0001 | 0.0000 | 0 | 0.0002 | 0.0000 | 0.0037 | 0.0002 | 0.0007 | 0.5457 | 0.0058 | 0.0000 | 0.0000 | 0 | 0 | 0.0393 | 0.4755 | 0.0026 | 0.1102 |
| **Antarctica** | 0.9375 | 0 | 0.0024 | 0 | 0.0114 | 0 | 0 | 0.0002 | 0 | 0 | 0.4286 | 0.0089 | 0 | 0 | 0 | 0.0023 | 0.0594 | 0.7202 | 0.0061 | 0.1473 |
| **Central Arctic** | 0.7444 | 0 | 0.0004 | 0.0000 | 0.0000 | 0 | 0 | 0 | 0 | 0 | 0.7260 | 0.0023 | 0 | 0 | 0 | 0 | 0 | 0.7668 | 0.0013 | 0.0027 |
| **Laptev Sea** | 0.6417 | 0.0000 | 0 | 0 | 0 | 0.0008 | 0.0008 | 0.0003 | 0.0000 | 0.0004 | 0.2537 | 0.0004 | 0 | 0.0001 | 0 | 0 | 0 | 0.2530 |  | 0.1731 |
| **Canadian High Arctic - North Greenland** | 0.5805 | 0.0001 | 0.0002 | 0.0000 | 0.0000 | 0 | 0.0000 | 0 | 0 | 0.0001 | 0.4454 | 0.0013 | 0 | 0.0000 | 0 | 0 | 0.0008 | 0.3128 | 0.0012 | 0.0519 |
