# Supplementary material for: Spatial and temporal changes in cumulative human impacts on the world's ocean
Source: Nat Commun. 2015 Jul 14;6:7615. doi: 10.1038/ncomms8615 (PMC4510691; doi:10.1038/ncomms8615)
Supplement: Supplementary Data 8 — Average difference in impact scores for each stressor and for cumulative impact between 2013 and 2008 for each large marine ecosystem (LME). Differences could only be calculated for the 12 (of 19) stressor layers that had data for both time. True zero values are indicated by zeros with no trailing decimals; very small values are zeros with several zero decimal values. [file ncomms8615-s9.doc]

## *Supplementary Data 8*

Average difference in impact scores for each stressor and for cumulative impact between 2013 and 2008 for each large marine ecosystem (LME). Differences could only be calculated for the 12 (of 19) stressor layers that had data for both time. True zero values are indicated by zeros with no trailing decimals; very small values are zeros with several zero decimal values.

| **Suppl. Data 8: 2013 minus 2008 LME**  **LME** | **Average cumulative impact score** | **Demersal destructive fishing** | **Demersal nondestructive high bycatch fishing** | **Demersal nondestructive low bycatch fishing** | **Direct human impact** | **Light pollution** | **Nutrient pollution** | **Oil rigs** | **Organic pollution** | **Pelagic high bycatch fishing** | **Pelagic low bycatch fishing** | **Sea surface temperature** | **UV** |
| --- | --- | --- | --- | --- | --- | --- | --- | --- | --- | --- | --- | --- | --- |
| **Northwest Australian Shelf** | 1.32949 | -0.00122 | -0.00077 | -0.00032 | 0.00008 | 0.00002 | -0.00042 | 0.00002 | -0.00003 | -0.00069 | -0.00008 | 1.32449 | 0.01242 |
| **West Central Australian Shelf** | 1.08268 | -0.00029 | -0.00037 | -0.00011 | 0.00005 | 0.00000 | -0.00015 | 0 | -0.00003 | -0.00097 | -0.00005 | 1.08367 | 0.00314 |
| **East Brazil Shelf** | 1.06630 | 0.00278 | 0.00165 | 0.00099 | 0.00019 | 0.00002 | 0.00101 | 0.00033 | 0.00011 | 0.00297 | 0.00054 | 1.04856 | 0.00917 |
| **Northeast Australian Shelf** | 1.04552 | -0.00038 | -0.00020 | -0.00106 | 0.00004 | -0.00001 | 0.00010 | 0 | 0.00001 | -0.00006 | -0.00146 | 1.03828 | 0.01187 |
| **Sulu-Celebes Sea** | 1.01620 | -0.00407 | -0.00436 | -0.00252 | 0.00080 | -0.00015 | -0.00129 | 0.00000 | -0.00013 | -0.00009 | -0.00132 | 1.06054 | -0.02318 |
| **Indonesian Sea** | 0.98933 | -0.01374 | -0.01147 | -0.00736 | 0.00035 | -0.00003 | 0.00160 | 0.00000 | 0.00020 | -0.00017 | -0.00289 | 1.02289 | 0.00551 |
| **Somali Coastal Current** | 0.97707 | -0.01124 | -0.01064 | -0.00420 | 0.00022 | -0.00003 | 0.00043 | 0 | 0.00009 | -0.00532 | -0.00152 | 1.00699 | 0.00561 |
| **Agulhas Current** | 0.89335 | -0.01616 | -0.00464 | -0.00674 | 0.00006 | -0.00001 | 0.00032 | -0.00001 | 0.00001 | -0.00182 | -0.00356 | 0.88385 | 0.04416 |
| **Iberian Coastal** | 0.78893 | -0.01474 | -0.01065 | -0.00826 | 0.00008 | -0.00002 | -0.00173 | 0 | -0.00079 | 0 | -0.00510 | 0.85742 | -0.02355 |
| **Guinea Current** | 0.77431 | -0.00248 | -0.00170 | -0.00202 | 0.00010 | -0.00001 | 0.00007 | 0.00068 | 0.00004 | -0.00448 | -0.00043 | 0.78650 | -0.00038 |
| **East Central Australian Shelf** | 0.74681 | -0.00056 | -0.00025 | -0.00049 | 0.00000 | 0.00002 | -0.00019 | 0 | 0.00003 | -0.00001 | -0.00054 | 0.70594 | 0.04401 |
| **Pacific Central-American Coastal** | 0.72916 | 0.00192 | 0.00089 | 0.00091 | 0.00006 | 0.00000 | -0.00041 | 0 | 0.00029 | 0.00168 | 0.00021 | 0.70105 | 0.02420 |
| **South China Sea** | 0.59969 | -0.02705 | -0.02733 | -0.01285 | 0.00012 | -0.00003 | 0.00067 | 0.00010 | 0.00016 | -0.00156 | -0.00415 | 0.67903 | -0.00562 |
| **Oyashio Current** | 0.59495 | 0.00387 | 0.00096 | 0.00145 | -0.00005 | -0.00001 | 0.00011 | 0 | 0.00000 | 0 | -0.00033 | 0.55272 | 0.03722 |
| **Bay of Bengal** | 0.58681 | -0.01229 | -0.01187 | -0.00628 | 0.00009 | 0.00001 | 0.00077 | -0.00001 | 0.00007 | -0.00223 | -0.00265 | 0.62691 | -0.00413 |
| **Sea of Okhotsk** | 0.51545 | 0.05522 | 0.01600 | 0.01742 | 0.00006 | 0.00002 | 0.00013 | -0.00001 | -0.00002 | 0 | -0.00005 | 0.39570 | 0.03233 |
| **North Australian Shelf** | 0.50484 | -0.00171 | -0.00086 | -0.00063 | 0.00030 | 0.00000 | 0.00059 | 0 | 0.00004 | -0.00010 | -0.00044 | 0.49411 | 0.01656 |
| **South West Australian Shelf** | 0.47105 | -0.00061 | -0.00064 | -0.00014 | 0.00008 | 0.00000 | -0.00012 | 0 | 0.00000 | -0.00002 | -0.00003 | 0.48073 | -0.00711 |
| **Arabian Sea** | 0.41673 | -0.00904 | -0.00788 | -0.00740 | 0.00021 | 0.00003 | 0.00060 | -0.00004 | -0.00004 | -0.00394 | -0.00586 | 0.46073 | -0.00969 |
| **Caribbean Sea** | 0.36159 | -0.01238 | -0.00884 | -0.00756 | 0.00011 | -0.00005 | 0.00026 | 0.00001 | 0.00019 | -0.00139 | -0.00082 | 0.36536 | 0.02790 |
| **Gulf of California** | 0.36010 | -0.00279 | -0.00051 | -0.00375 | 0.00043 | 0.00000 | 0.00055 | 0 | 0.00230 | -0.00012 | -0.00018 | 0.37922 | -0.01142 |
| **Humboldt Current** | 0.31853 | -0.01044 | -0.00824 | -0.01500 | -0.00082 | 0.00001 | 0.00021 | 0.00000 | 0.00014 | -0.00054 | -0.00092 | 0.35843 | -0.00213 |
| **North Brazil Shelf** | 0.31291 | -0.00717 | -0.00223 | -0.00313 | 0.00000 | 0.00001 | 0.00032 | -0.00001 | -0.00003 | -0.00285 | -0.00022 | 0.32164 | 0.00792 |
| **Benguela Current** | 0.29655 | -0.00746 | -0.00401 | -0.01891 | 0.00013 | 0.00001 | 0.00008 | 0.00041 | 0.00001 | 0.00000 | -0.00024 | 0.34152 | -0.01449 |
| **Canary Current** | 0.27635 | -0.00351 | -0.00295 | -0.00304 | 0.00002 | -0.00001 | -0.00072 | 0.00005 | -0.00044 | -0.00170 | -0.00046 | 0.28227 | 0.00751 |
| **Gulf of Thailand** | 0.26081 | -0.11297 | -0.10246 | -0.04989 | 0.00095 | -0.00037 | -0.00006 | -0.00009 | -0.00016 | -0.00008 | -0.00279 | 0.53805 | -0.00618 |
| **Southeast U.S. Continental Shelf** | 0.17322 | -0.01330 | -0.00572 | -0.00490 | -0.00040 | -0.00022 | -0.00136 | 0 | -0.00018 | -0.00083 | -0.00009 | 0.19739 | 0.00389 |
| **Hudson Bay Complex** | 0.16584 | -0.00011 | -0.00001 | 0.00000 | 0.00007 | 0.00002 | 0.00007 | 0 | 0.00004 | 0 | 0.00000 | 0.20401 | -0.03613 |
| **Gulf of Mexico** | 0.13725 | -0.00794 | -0.00412 | -0.00478 | -0.00011 | -0.00005 | -0.00062 | 0.00002 | 0.00037 | -0.00159 | -0.00015 | 0.14828 | 0.00837 |
| **Black Sea** | 0.13207 | -0.02255 | -0.01939 | -0.05419 | -0.00019 | 0.00002 | 0.00243 | -0.00011 | 0.00183 | -0.00019 | -0.00593 | 0.24449 | -0.01296 |
| **Labrador - Newfoundland** | 0.13090 | -0.04165 | -0.02274 | -0.00812 | -0.00012 | 0.00002 | 0.00022 | -0.00001 | 0.00006 | 0 | -0.00546 | 0.25176 | -0.04076 |
| **Kuroshio Current** | 0.12531 | -0.00993 | -0.00997 | -0.00889 | -0.00025 | -0.00011 | -0.00039 | 0 | -0.00013 | -0.00133 | -0.00273 | 0.15070 | 0.00877 |
| **Scotian Shelf** | 0.10704 | -0.03095 | -0.02948 | -0.01315 | -0.00016 | -0.00021 | -0.00042 | 0 | 0.00004 | 0 | -0.01016 | 0.21224 | -0.01914 |
| **West Bering Sea** | 0.10304 | 0.01491 | 0.00383 | 0.01150 | 0.00006 | 0.00001 | 0.00014 | 0 | 0.00001 | 0 | 0.00000 | 0.06433 | 0.00851 |
| **South Brazil Shelf** | 0.10084 | 0.00296 | 0.00173 | 0.00068 | -0.00035 | 0.00010 | 0.00089 | 0.00010 | 0.00006 | 0.00056 | 0.00045 | 0.03771 | 0.05609 |
| **Canadian Eastern Arctic - West Greenland** | 0.06760 | -0.03096 | -0.00517 | -0.00080 | 0.00017 | 0.00001 | 0.00003 | 0 | 0.00000 | 0 | -0.00004 | 0.09691 | 0.01136 |
| **Mediterranean Sea** | 0.06230 | -0.00923 | -0.01625 | -0.00557 | 0.00008 | -0.00008 | -0.00126 | -0.00001 | -0.00024 | -0.00271 | -0.00050 | 0.11816 | -0.01929 |
| **Antarctica** | 0.05285 | 0.00153 | 0 | 0.00199 | 0 | 0 | 0 | 0 | 0 | 0 | 0.00146 | 0.11998 | 0.00275 |
| **East China Sea** | 0.04817 | -0.03266 | -0.02918 | -0.01215 | -0.00020 | 0.00003 | 0.00083 | 0.00000 | 0.00016 | -0.00273 | -0.00148 | 0.09658 | 0.02956 |
| **Sea of Japan** | 0.03708 | -0.00415 | -0.00647 | -0.00114 | -0.00012 | -0.00011 | -0.00048 | 0 | -0.00013 | 0 | -0.00071 | 0.06425 | -0.01358 |
| **Yellow Sea** | 0.03570 | -0.02900 | -0.03394 | -0.01397 | -0.00011 | 0.00016 | 0.00165 | -0.00013 | 0.00060 | 0 | -0.00043 | 0.04517 | 0.06609 |
| **Red Sea** | 0.01670 | -0.04113 | -0.03144 | -0.01369 | 0.00014 | 0.00006 | -0.00068 | -0.00025 | -0.00031 | -0.00064 | -0.00021 | 0.10382 | 0.00203 |
| **New Zealand Shelf** | 0.00779 | -0.05348 | -0.01780 | -0.00642 | 0.00004 | -0.00004 | -0.00086 | 0 | -0.00114 | -0.00032 | -0.00028 | 0.08759 | 0.00087 |
| **Central Arctic** | -0.00010 | -0.00046 | 0.00000 | 0.00000 | 0 | 0 | 0 | 0 | 0 | 0 | 0 | -0.11261 | 0.00205 |
| **Laptev Sea** | -0.00436 | 0 | 0 | 0 | -0.00051 | 0.00000 | 0.00001 | 0 | 0.00000 | 0 | 0 | -0.02928 | 0.02047 |
| **Northeast U.S. Continental Shelf** | -0.00703 | -0.03632 | -0.01903 | -0.01178 | -0.00039 | -0.00050 | -0.00098 | 0 | -0.00012 | 0 | -0.00468 | 0.11780 | -0.04934 |
| **Beaufort Sea** | -0.02214 | -0.00001 | 0.00000 | 0 | -0.00001 | 0.00001 | 0.00027 | 0.00000 | 0.00002 | 0 | 0 | 0.13494 | -0.08763 |
| **Celtic-Biscay Shelf** | -0.02240 | -0.05244 | -0.03735 | -0.01671 | -0.00002 | -0.00014 | 0.00106 | 0 | 0.00006 | 0 | -0.01542 | 0.11186 | -0.01237 |
| **Canadian High Arctic - North Greenland** | -0.05811 | -0.00006 | -0.00001 | -0.00001 | 0 | 0 | 0.00002 | 0 | 0.00000 | 0 | 0 | -0.14648 | -0.02073 |
| **Baltic Sea** | -0.05854 | -0.01133 | -0.05855 | -0.03759 | 0.00031 | 0.00009 | -0.00081 | 0 | 0.00091 | 0 | -0.02075 | 0.11926 | -0.04764 |
| **Southeast Australian Shelf** | -0.07098 | -0.00043 | -0.00030 | -0.00009 | 0.00006 | 0.00000 | -0.00009 | 0.00001 | -0.00003 | -0.00001 | -0.00002 | -0.02105 | -0.04906 |
| **Northern Bering - Chukchi Seas** | -0.09041 | -0.00774 | -0.00275 | 0.00012 | -0.00010 | 0.00001 | 0.00020 | 0 | 0.00002 | 0 | -0.00002 | -0.17110 | 0.05170 |
| **Kara Sea** | -0.14317 | -0.00307 | -0.00018 | -0.00008 | -0.00027 | 0.00002 | 0.00002 | 0 | 0.00001 | 0 | 0 | -0.16634 | -0.01304 |
| **Patagonian Shelf** | -0.14517 | -0.07885 | -0.01942 | -0.01023 | 0.00002 | 0.00001 | 0.00004 | 0.00000 | 0.00012 | -0.00001 | -0.00008 | -0.06816 | 0.03115 |
| **North Sea** | -0.15999 | -0.08428 | -0.05824 | -0.02932 | 0.00025 | 0.00002 | -0.00034 | -0.00024 | 0.00018 | 0 | -0.02322 | 0.02662 | 0.00883 |
| **Faroe Plateau** | -0.16358 | -0.15043 | -0.03076 | -0.01275 | 0.00001 | 0.00015 | 0 | 0 | 0 | 0 | -0.00880 | 0.07014 | -0.03104 |
| **Greenland Sea** | -0.17497 | -0.01669 | -0.00694 | -0.00819 | 0.00014 | 0.00000 | 0 | 0 | 0 | 0 | -0.00104 | -0.20059 | 0.02662 |
| **Insular Pacific-Hawaiian** | -0.17504 | -0.00053 | -0.00038 | -0.00064 | -0.00003 | -0.00002 | 0.00024 | 0 | 0.00006 | -0.00002 | -0.00025 | -0.21238 | 0.03877 |
| **Barents Sea** | -0.18116 | -0.06313 | -0.05620 | -0.01807 | 0.00019 | 0.00020 | -0.00001 | 0 | 0.00000 | 0 | -0.00315 | -0.05571 | 0.00872 |
| **Norwegian Sea** | -0.22389 | -0.05517 | -0.01993 | -0.01861 | 0.00004 | 0.00029 | 0.00014 | -0.00002 | 0.00000 | 0 | -0.01916 | -0.11507 | 0.00324 |
| **East Siberian Sea** | -0.22535 | -0.00002 | 0.00000 | 0 | -0.00011 | 0.00000 | 0.00001 | 0 | 0.00000 | 0 | 0 | -0.30759 | 0.05703 |
| **California Current** | -0.25496 | -0.00118 | -0.00086 | -0.00210 | -0.00001 | -0.00002 | -0.00006 | 0 | 0.00001 | -0.00016 | -0.00132 | -0.26849 | 0.01876 |
| **Iceland Shelf and Sea** | -0.27386 | -0.07831 | -0.06047 | -0.04085 | -0.00018 | 0.00002 | 0.00003 | 0 | 0.00000 | 0 | -0.02439 | -0.03097 | -0.03886 |
| **Aleutian Islands** | -0.48007 | -0.02560 | -0.00421 | -0.00720 | 0.00014 | 0.00001 | 0.00006 | 0 | 0.00001 | 0 | -0.00021 | -0.41229 | -0.03405 |
| **East Bering Sea** | -0.74419 | -0.04420 | -0.01342 | -0.01080 | 0.00007 | 0.00001 | -0.00016 | 0 | 0.00000 | 0 | -0.00067 | -0.66481 | -0.01251 |
| **Gulf of Alaska** | -0.94104 | -0.01482 | -0.00280 | -0.00530 | -0.00012 | 0.00005 | -0.00025 | 0.00000 | -0.00001 | 0 | -0.00022 | -0.96149 | 0.03128 |
